# Supplementary material for: A Polyol-Polyol Super-Carbon-Chain Compound Containing Thirty-Six Carbon Stereocenters from the Dinoflagellate Amphidinium gibbosum: Absolute Configuration and Multi-Segment Modification
Source: Mar Drugs. 2020 Nov 26;18(12):590. doi: 10.3390/md18120590 (PMC7759953; doi:10.3390/md18120590)

Supporting Information-I

Table of Contents:

Table S1. $^{3}J_{H,H}$ (Hz) of the 1,2,3,4-tetraol moiety in **1c** (CD_3OD) ········· S2

Table S2. ^{1}H (700 MHz) and ^{13}C (175 MHz) NMR data for the fragment **1a** ········· S3

Table S3. ^{1}H (700 Hz) and ^{13}C (175 Hz) NMR data for the fragment **1b** ········· S3-S4

Table S4. ^{1}H (700 MHz) and ^{13}C (175 MHz) NMR data for the fragment **1c** ········· S4-S5

Table S5. ^{1}H (400 MHz) NMR data for **1as/r** in CD_3OD ········· S6

Table S1. $^{3}J_{H,H}$ (Hz) of the 1,2,3,4-tetraol moiety in **1c** (CD_3OD).[1]

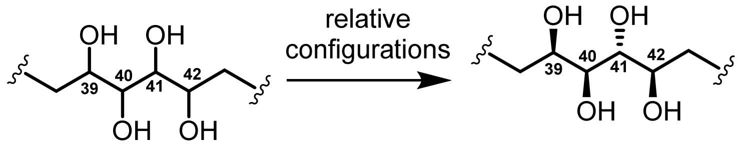

	**1c**	Kishi's universal NMR database							
		SSS	AAA	ASA	SAS	**SAA**	AAS	SSA	ASS
$^{3}J_{H\text{-}39,H\text{-}40}$	1.8	4.4	5.1	8.7	1.8	**2.0**	4.4	4.7	8.1
$^{3}J_{H\text{-}40,H\text{-}41}$	8.1	4.8	6.7	1.0	9.2	**8.5**	8.5	2.3	2.3
$^{3}J_{H\text{-}41,H\text{-}42}$	4.9	4.0	5.8	8.9	1.4	**4.4**	2.0	8.1	4.7
$\Sigma\|\Delta Hz\|$	N/A	6.8	5.6	18.0	4.6	**1.1**	5.9	11.9	12.3

A: *anti*; S: *syn*

Reference

[1]. Seike, I. Ghoshi, Y. Kishi, Attempts to assemble a universal NMR database without synthesis of NMR database compounds. *Org*. *Lett*. **2006**, *8*, 3861-3864.

Table S2. ^{1}H (700 MHz) and ^{13}C (175 MHz) NMR data for the fragment **1a** in CD_3OD (δ in ppm and J in Hz).

No.	δ_H	δ_C
1a	3.41 dd (11.2, 6.3)	67.4 CH_2
1b	3.46 dd (11.2, 4.2)	
2	3.54 m	73.5 CH
3a	1.39 m	31.8 CH_2
3b	1.51 m	
4a	1.32 m	34.8 CH_2
4b	1.36 m	
5	1.69 m	30.3 CH
6a	1.17 m	46.2 CH_2
6b	1.43 m	
7	3.76 m	68.1 CH
8a	1.28 m	43.2 CH_2
8b	1.48 m	
9	1.78 m	33.7 CH
10a	3.38 dd (10.5, 6.3)	68.0 CH_2
10b	3.44 dd (10.5, 5.6)	
71	0.91 d (7.0)	19.5 CH_3
72	0.94 d (7.0)	18.0 CH_3

Table S3. ^{1}H (700 Hz) and ^{13}C (175 Hz) NMR data for the fragment **1b** in CD_3OD (δ in ppm, J in Hz).

No.	δ_H	δ_C
13	3.74 m	68.6 CH
13'	3.73 m	68.9 CH
14a/14b	1.48 m/1.65 m	36.5 CH_2
14a'/14b'		36.7 CH_2
15a/15b	1.46 m/1.66 m	29.9 CH_2
15a'/15b'	1.38 m/1.73 m	30.2 CH_2
16	3.41 m	76.3 CH
16'	3.40 m	76.5 CH
17	3.69 m	72.8 CH
18a	1.50 m	41.3 CH_2
18b	1.67 m	
19	4.10 m	66.5 CH
20a	1.58 m	46.9 CH_2
20b	1.58 m	
21	4.11 m	66.44 CH
22a	1.58 m	46.9 CH_2
22b	1.58 m	

23	4.11 m	66.36 CH
24a	1.55 m	41.5 CH_2
24b	1.80 m	
25	3.89 ddd (9.8, 6.3, 2.1)	70.7 CH
26	3.37 t (6.3)	78.7 CH
27	3.85 ddd (9.1, 6.3, 2.8)	73.3 CH
28a	1.58 m	40.4 CH_2
28b	1.86 m	
29	3.97 m	69.9 CH
30a	1.32 m	42.6 CH_2
30b	1.56 m	
31	1.81 m	33.4 CH
32a	3.39 dd (10.5, 6.3)	68.0 CH_2
32b	3.47 dd (10.5, 5.6)	
74	1.16 d (6.3)	23.58 CH_3
74'		23.63 CH_3
75	0.96 d (6.3)	18.2 CH_3

Table S4. ^{1}H (700 MHz) and ^{13}C (175 MHz) NMR data for the fragment **1c** in CD_3OD (δ in ppm, J in Hz).

No.	δ_H	δ_C
33	3.75 m	68.49 CH
33'		68.54 CH
34a/34b	1.50 m/1.61 m	36.5 CH_2
34a'/34b'	1.49 m/1.63 m	36.7 CH_2
35a/35b	1.58 m/1.60 m	30.2 CH_2
35a'/35b'	1.47 m/1.68 m	30.4 CH_2
36	3.44 m	75.4 CH
37	3.68 m	73.6 CH
38a	1.78 m	37.3 CH_2
38b	1.82 m	
39	4.13 ddd (9.2, 5.3, 1.8)	70.3 CH
40	3.45 dd (8.1, 1.8)	74.6 CH
41	3.69 dd (8.1, 4.9)	75.6 CH
42	4.04 ddd (10.5, 4.9, 2.1)	70.0 CH
43a	1.77 m	34.8 CH_2
43b	1.98 m	
44	3.67 m	70.9 CH
45	3.03 t (8.4)	77.6 CH
46	3.74 m	70.3 CH
47a	1.70 m	37.6 CH_2
47b	1.88 m	
48	3.93 m	73.1 CH

49a	1.38 m	32.4 CH_2
49b	1.92 m	
50a	1.52 m	23.3 CH_2
50b		
51a	1.52 m	32.7 CH_2
51b	1.75 m	
52	3.41 m	76.4 CH
53	3.14 t (7.7)	75.2 CH
54	3.72 t (7.7)	75.1 CH
55	3.78 dd (7.7, 4.9)	73.8 CH
56	3.60 dd (9.1, 4.9)	75.7 CH
57	4.40 m	67.7 CH
58a	1.52 m	42.0 CH_2
58b	1.86 m	
59	4.10 m	67.1 CH
60a	1.67 m	42.6 CH_2
60b	1.84 m	
61	4.09 m	69.7 CH
62	3.25 dd (7.7, 1.4)	77.1 CH
63	3.83 ddd (9.8, 7.7, 2.8)	71.8 CH
64a	1.63 m	42.5 CH_2
64b	1.94 m	
65	4.11 m	68.8 CH
66a	1.55 m	41.7 CH_2
66b	1.59 m	
67	3.87 m	70.1 CH
68a	3.46 dd (11.2, 6.3)	67.9 CH_2
68b	3.49 dd (11.2, 4.2)	
76	1.17 d (6.3)	23.6 CH_3
76'		23.5 CH_3

Table S5. ^{1}H (400 MHz) NMR data for **1as** and **1ar** in CD_3OD (δ in ppm, J in Hz).

No.	**1as**	**1ar**
1a	4.34 dd (12.8, 6.0)	4.22 dd (12.4, 4.4)
1b	4.71 dd (12.8, 2.4)	4.55 dd (12.4, 2.4)
2	5.26 m	5.19 m
3a	1.49 m	1.50 m
3b	1.49 m	1.50 m
4a	0.95 m	1.05 m
4b	1.09 m	1.10 m
5	1.24 m	1.06 m
6a	1.11 m	1.10 m
6b	1.46 m	1.43 m
7	5.16 m	5.19 m
8a	1.48 m	1.56 m
8b	1.48 m	1.56 m
9	1.69 m	1.84 m
10a	4.13 dd (10.8, 4.8)	4.18 dd (10.8, 6.0)
10b	4.18 dd (10.8, 5.6)	4.29 dd (10.8, 5.2)
71	0.74 d (6.4)	0.73 d (5.2)
72	0.86 d (6.4)	0.96 d (6.8)

Supporting Information-II

Copies of High Performance Liquid Chromatogram, the UV spectrum, HR-ESI-MS for **1**; LR-ESI-MS for **1a**–**1c**, **1as**, and **1ar**; and 1D and 2D NMR spectra for **1**, **1a**–**1c**, **1as**, and **1ar**.

Table of Contents:

1. High Performance Liquid Chromatogram and the UV spectrum for compound **1**··············S5
2. HR-ESI-MS for compound **1** ··S6-S7
3. ^{1}H (700 MHz) NMR spectrum of compound **1** in CD_3OD·································S8-S14
4. ^{13}C (175 MHz) NMR spectrum of compound **1** in CD_3OD·······························S15-S21
5. DEPT135 (175 MHz) spectrum of compound **1** in CD_3OD ·······························S22-S26
6. ^{1}H–^{1}H COSY (700 MHz) spectrum of compound **1** in CD_3OD ·······················S27-S30
7. HSQC (700 MHz) spectrum of compound **1** in CD_3OD ···································S31-S34
8. Selective-HSQC (700 MHz) spectrum of compound **1** in CD_3OD·························S35-S41
9. HMBC (700 MHz) spectrum of compound **1** in CD_3OD ···································S42-S47
10. H2BC (700 MHz) spectrum of compound **1** in CD_3OD ···································S48-S53
11. NOESY (700 MHz) spectrum of compound **1** in CD_3OD ·······························S54-S56
12. ^{13}C (175 MHz) NMR spectrum of compound **1** in CD_3OH···································S57
13. Comparison of ^{13}C (175 MHz) NMR spectrum of compound **1** in CD_3OH with that in CD_3OD·······S58-S60
14. LR-ESI-MS for the fragment **1a**···S61
15. ^{1}H (700 MHz) NMR spectrum of the fragment **1a** in CD_3OD··························S62-S64
16. ^{13}C (175 MHz) NMR spectrum of the fragment **1a** in CD_3OD························S65-S66
17. DEPT135 (175 MHz) spectrum of the fragment **1a** in CD_3OD ························S67-S68
18. ^{1}H–^{1}H COSY (700 MHz) spectrum of the fragment **1a** in CD_3OD ·················S69-S72
19. HSQC (700 MHz) spectrum of the fragment **1a** in CD_3OD ····························S73-S75
20. HMBC (700 MHz) spectrum of the fragment **1a** in CD_3OD ····························S76-S77
21. 2D *J*RES (700 MHz) spectrum of the fragment **1a** in CD_3OD ··························S78-S81

21. HECADE (700 MHz) spectrum of the fragment **1a** in CD_3OD ······ S82-S86
22. LR-ESI-MS for the fragment **1b** ······ S87
23. ^1H (700 MHz) NMR spectrum of the fragment **1b** in CD_3OD ······ S88-S90
24. ^{13}C (175 MHz) NMR spectrum of the fragment **1b** in CD_3OD ······ S91-S93
25. DEPT135 (175 MHz) spectrum of the fragment **1b** in CD_3OD ······ S94-S96
26. ^1H–^1H COSY (700 MHz) spectrum of the fragment **1b** in CD_3OD ······ S97-S100
27. HSQC (700 MHz) spectrum of the fragment **1b** in CD_3OD ······ S101-S103
28. HMBC (700 MHz) spectrum of the fragment **1b** in CD_3OD ······ S104-S107
29. 2D *J*RES (700 MHz) spectrum of the fragment **1b** in CD_3OD ······ S108-S113
30. HETLOC (700 MHz) spectrum of the fragment **1b** in CD_3OD ······ S114-S122
31. HECADE (700 MHz) spectrum of the fragment **1b** in CD_3OD ······ S123-S130
32. LR-ESI-MS for the fragment **1c** ······ S131
33. ^1H (700 MHz) NMR spectrum of the fragment **1c** in CD_3OD ······ S132-S134
34. ^{13}C (175 MHz) NMR spectrum of the fragment **1c** in CD_3OD ······ S135-S137
35. DEPT135 (175 MHz) spectrum of the fragment **1c** in CD_3OD ······ S138-S140
36. ^1H–^1H COSY (700 MHz) spectrum of the fragment **1c** in CD_3OD ······ S141-S144
37. HSQC (700 MHz) spectrum of the fragment **1c** in CD_3OD ······ S145-S147
38. HMBC (700 MHz) spectrum of the fragment **1c** in CD_3OD ······ S148-S151
39. NOESY (700 MHz) spectrum of the fragment **1c** in CD_3OD ······ S152-S155
40. 2D *J*RES (700 MHz) spectrum of the fragment **1c** in CD_3OD ······ S156-S158

41. HECADE (700 MHz) spectrum of the fragment **1c** in CD_3OD ························ S159-S163
42. LR-ESI-MS for the fragment **1as**···S164
43. ^1H (400 MHz) NMR spectrum of the fragment **1as** in CD_3OD··························S165-S167
44. ^1H–^1H COSY (400 MHz) spectrum of the fragment **1as** in CD_3OD ····················S168-S171
45. LR-ESI-MS for the fragment **1ar**···S172
46. ^1H (400 MHz) NMR spectrum of the fragment **1ar** in CD_3OD··························S173-S175
47. ^1H–^1H COSY (400 MHz) spectrum of the fragment **1ar** in CD_3OD ····················S176-S179

High Performance Liquid Chromatogram and the UV spectrum for compound **1**

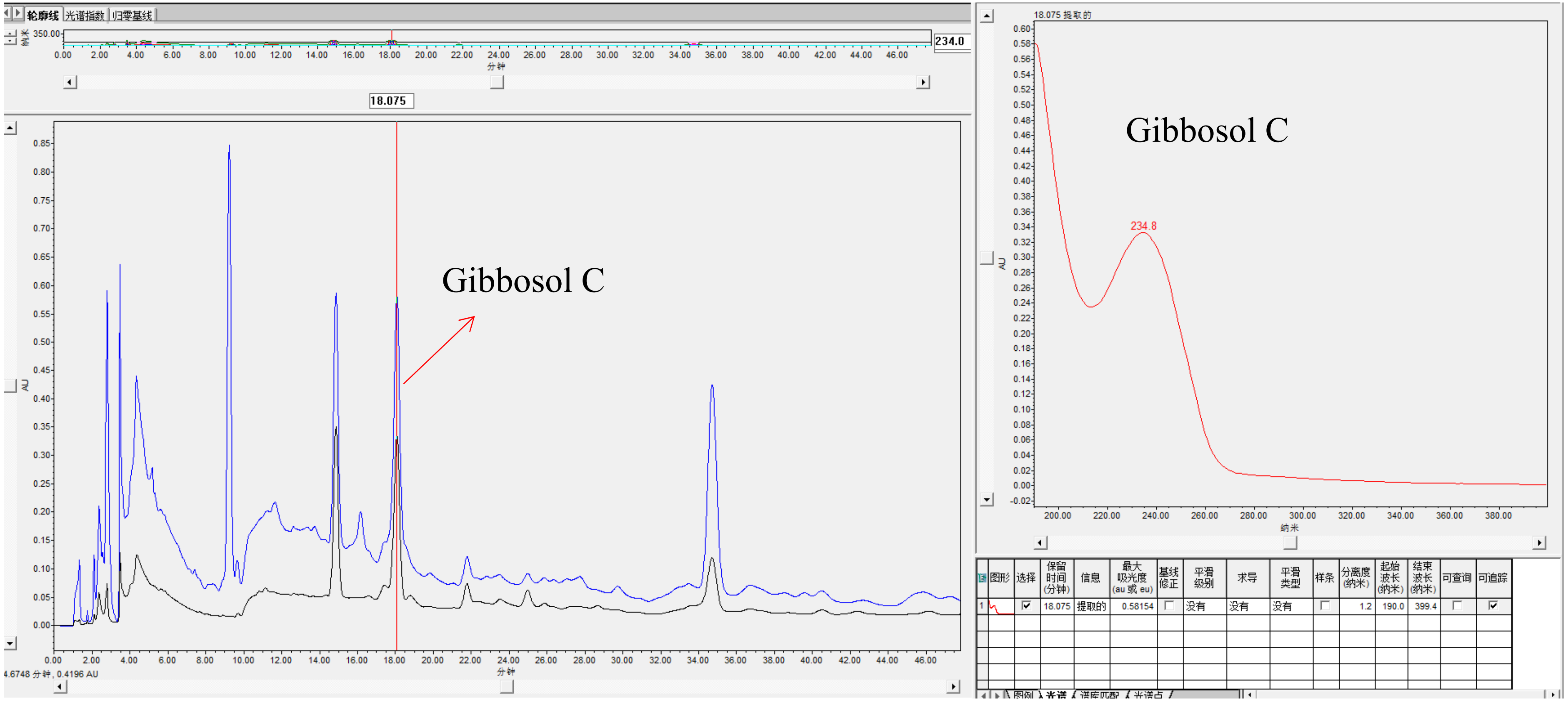

Mobile Phase: MeCN/H_2O, 21:79; Flow Rate: 1.0 mL/min

HR-ESI-MS for compound **1**

Mass Spectrum SmartFormula Report

Analysis Info

Analysis Name	D:\Data\MS\data\201905\liwanshan_40-2-2_pos_37_01_6600.d	Acquisition Date	5/6/2019 5:10:36 PM
Method	LC_Direct Infusion_pos_100-3000mz.m	Operator	SCSIO
Sample Name	liwanshan_40-2-2_pos	Instrument	maXis 255552.00029
Comment			

Acquisition Parameter

Source Type	ESI	Ion Polarity	Positive	Set Nebulizer	0.4 Bar
Focus	Active	Set Capillary	4500 V	Set Dry Heater	180 °C
Scan Begin	100 m/z	Set End Plate Offset	0 V	Set Dry Gas	4.0 l/min
Scan End	3500 m/z	Set Charging Voltage	0 V	Set Divert Valve	Waste
		Set Corona	0 nA	Set APCI Heater	0 °C

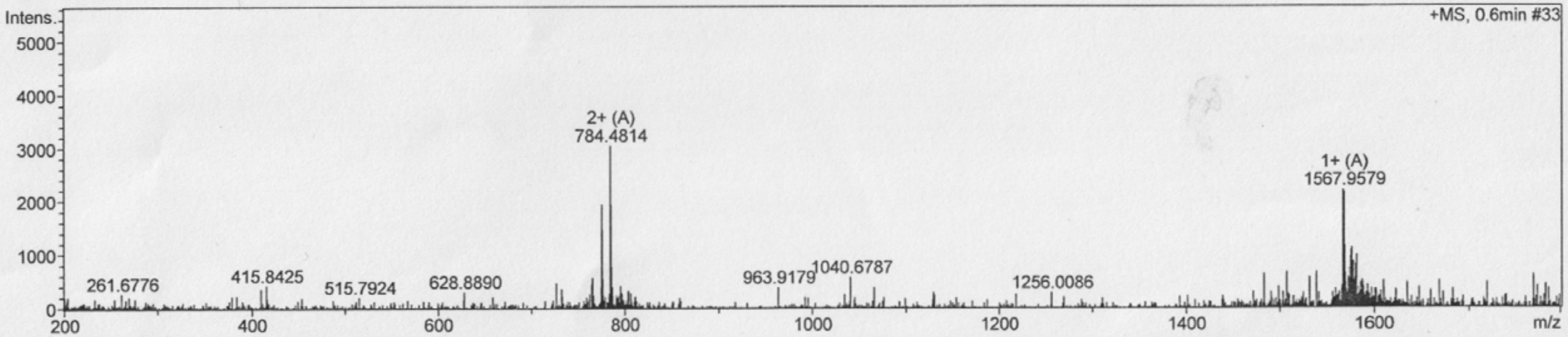

Meas. m/z	#	Ion Formula	Score	m/z	err [ppm]	err [mDa]	mSigma	rdb	e⁻ Conf	N-Rule
784.4814	1	C76H144O32	100.00	784.4815	0.1	0.1	118.4	5.0	even	ok
1567.9579	1	C76H143O32	100.00	1567.9557	1.4	2.2	79.1	5.5	even	ok

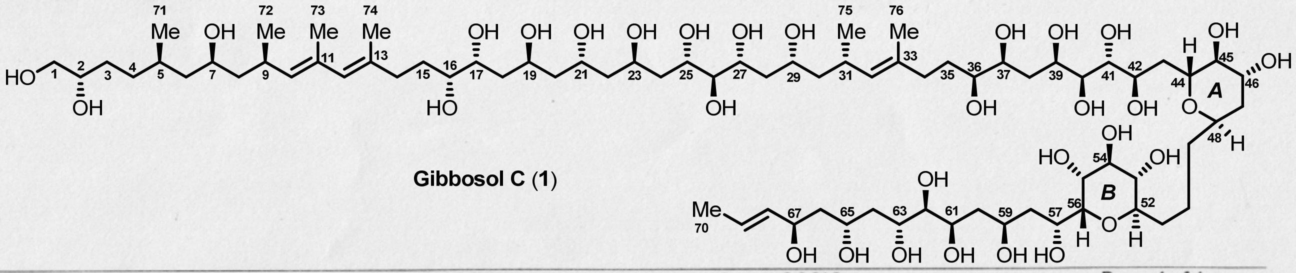

Gibbosol C (**1**)

HR-ESI-MS for compound **1**

Generic Display Report

Analysis Info

Analysis Name	D:\Data\MS\data\201905\liwanshan_40-2-2_pos_37_01_6600.d	Acquisition Date	5/6/2019 5:10:36 PM
Method	LC_Direct Infusion_pos_100-3000mz.m	Operator	SCSIO
Sample Name	liwanshan_40-2-2_pos	Instrument	maXis
Comment			

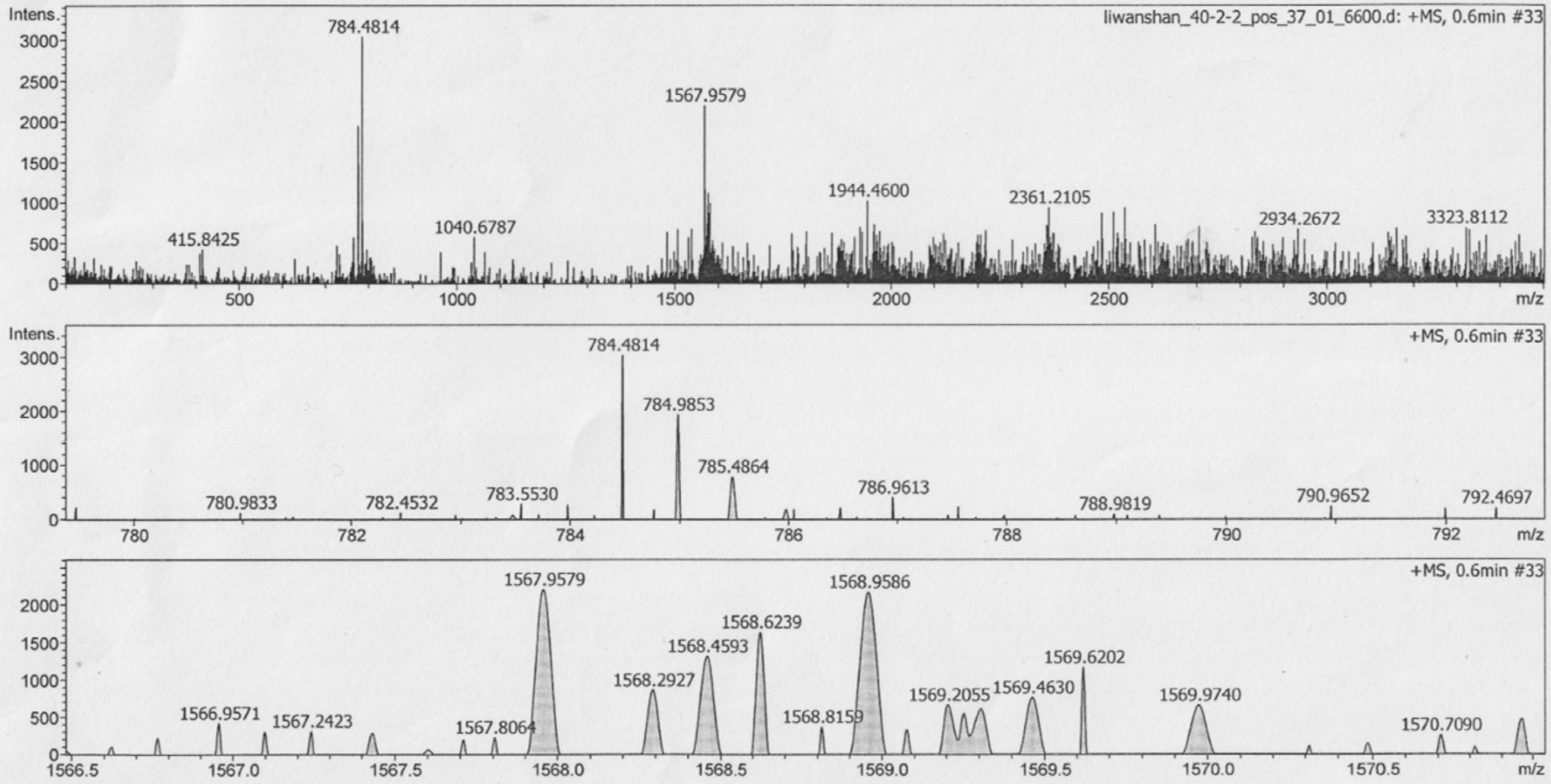

Bruker Compass DataAnalysis 4.1 printed: 5/6/2019 5:26:11 PM by: SCSIO Page 1 of 1

^{1}H (700 MHz) NMR spectrum of compound **1** in CD_3OD

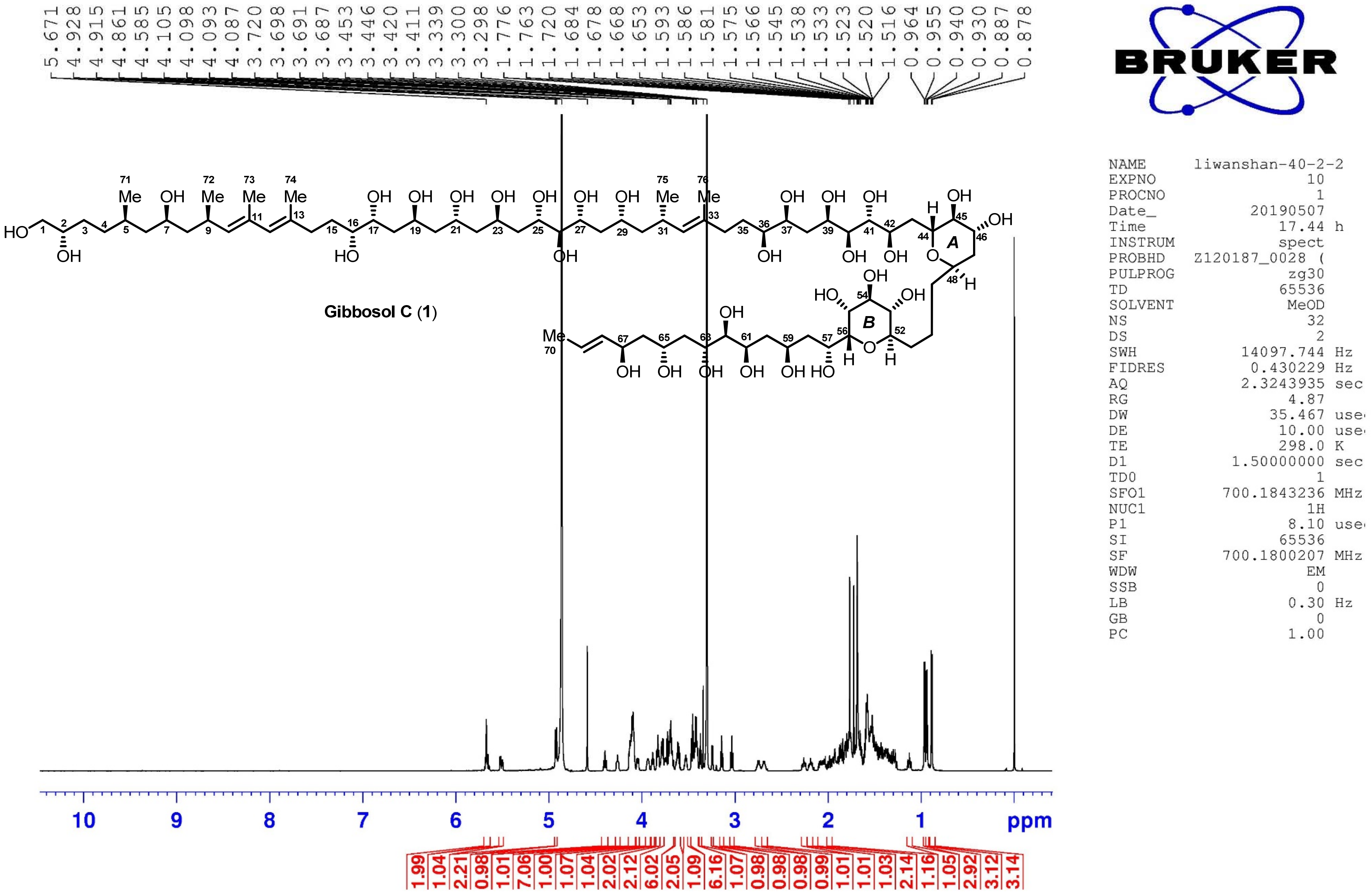

^{1}H (700 MHz) NMR spectrum of compound **1** in CD_3OD

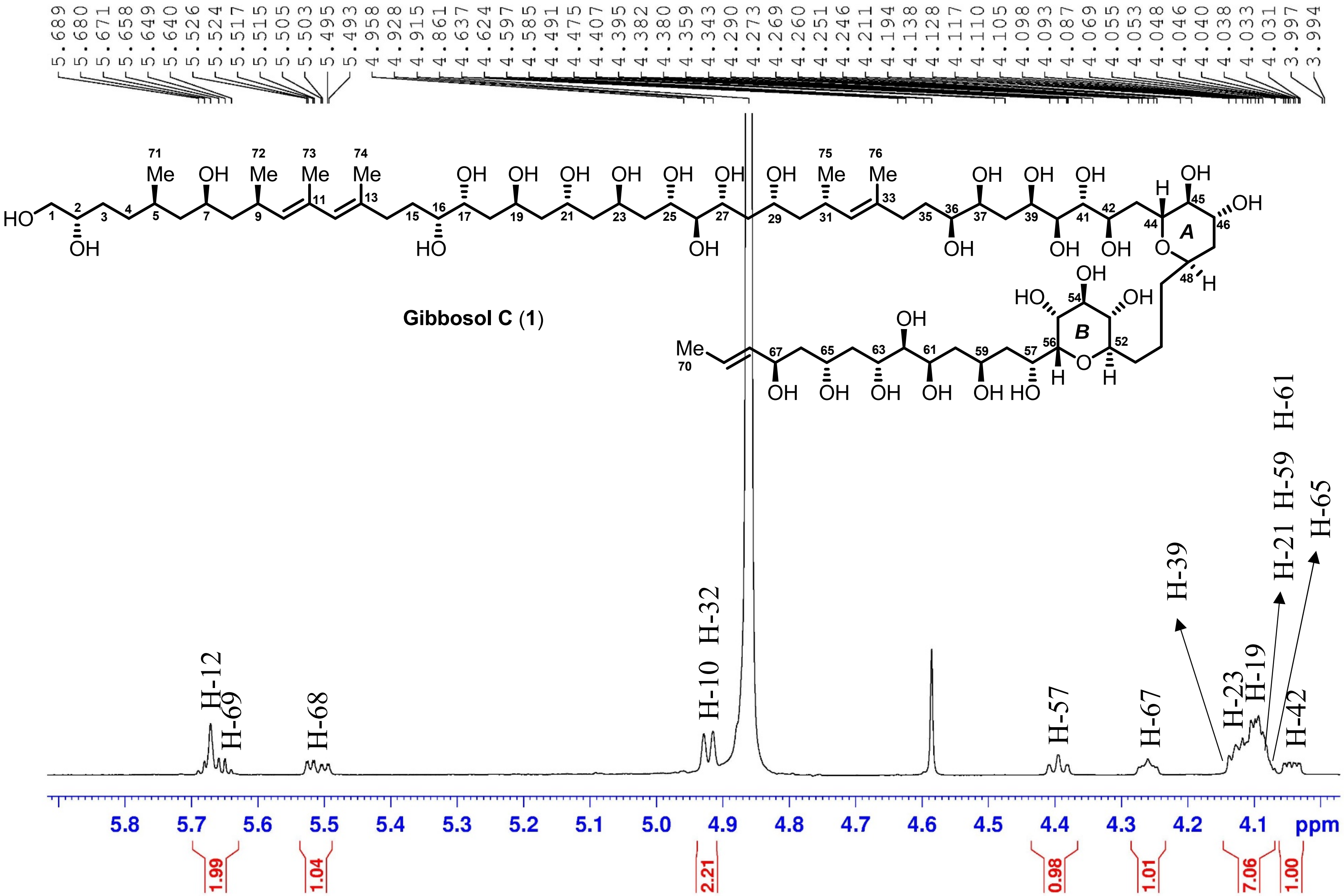

^{1}H (700 MHz) NMR spectrum of compound **1** in CD_3OD

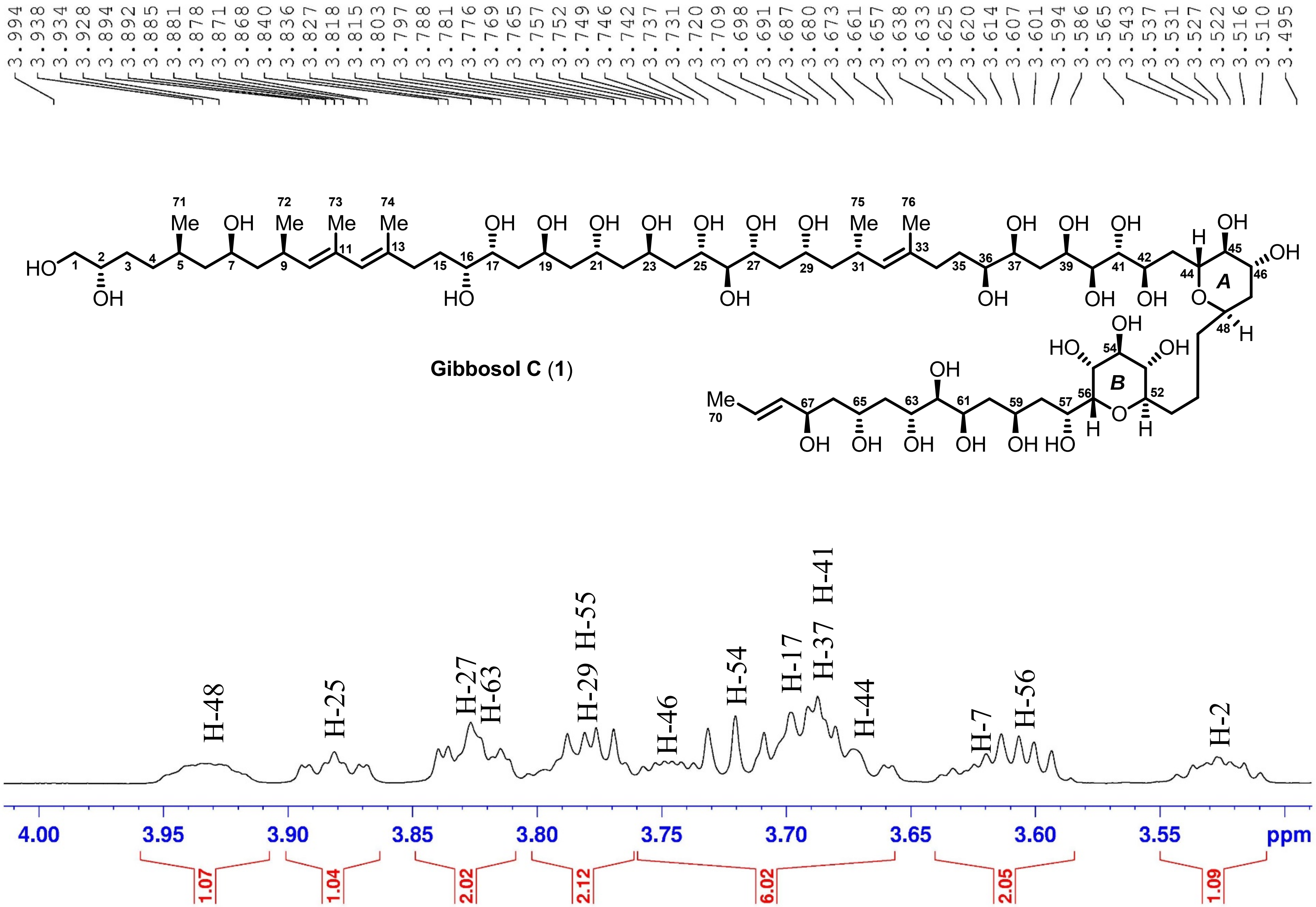

^{1}H (700 MHz) NMR spectrum of compound **1** in CD_3OD

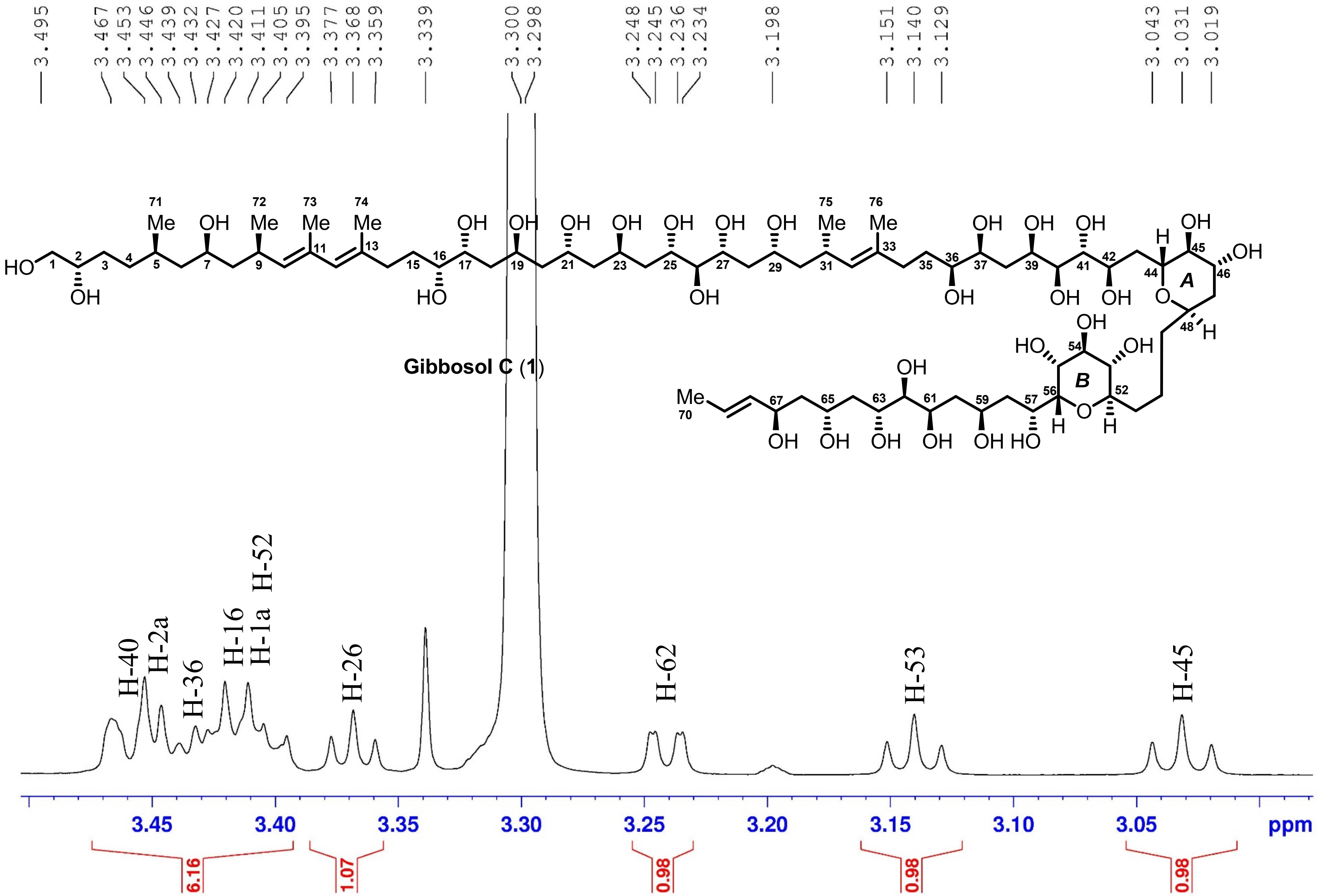

^{1}H (700 MHz) NMR spectrum of compound **1** in CD_3OD

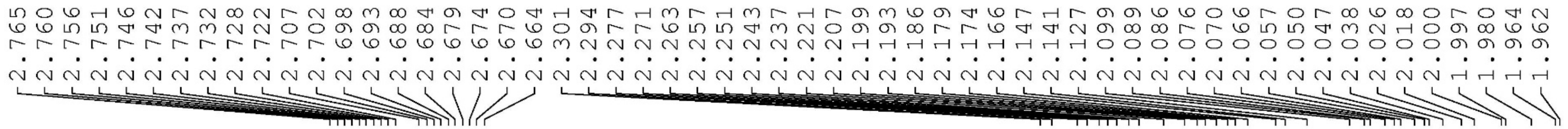

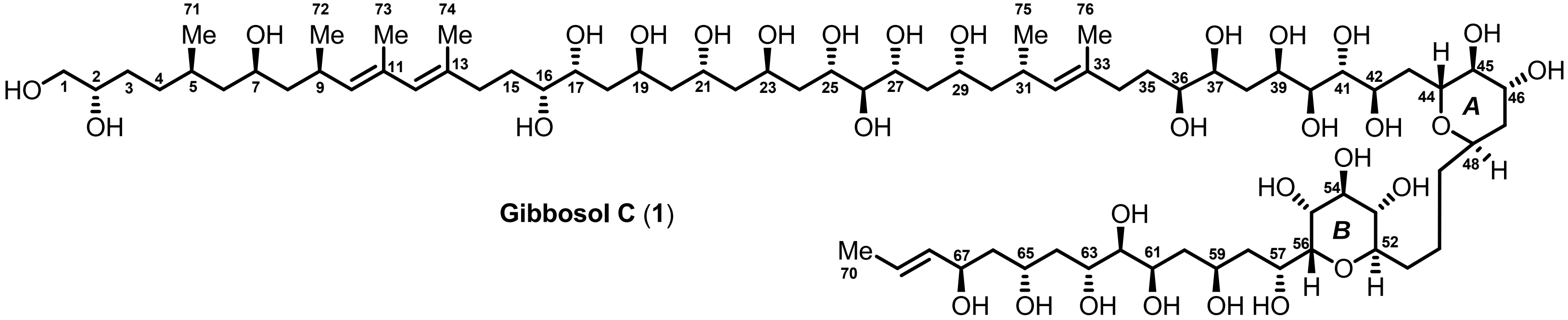

Gibbosol C (**1**)

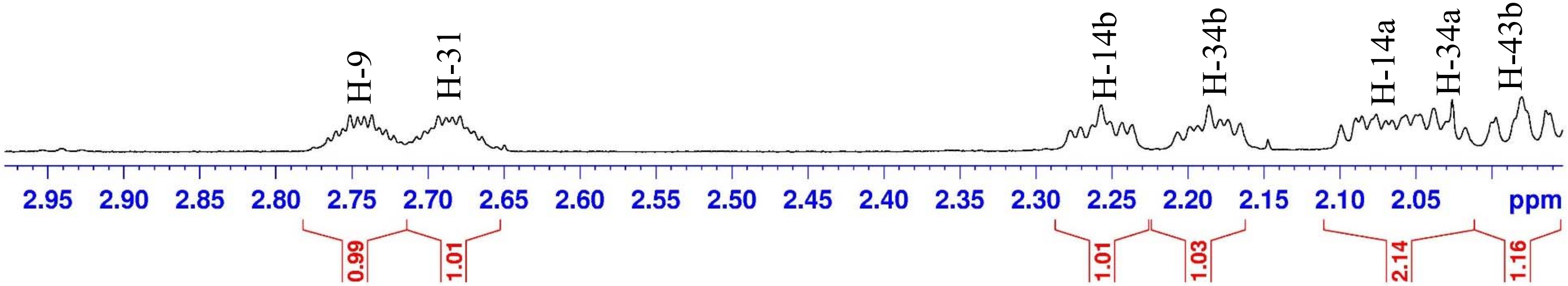

^{1}H (700 MHz) NMR spectrum of compound **1** in CD_3OD

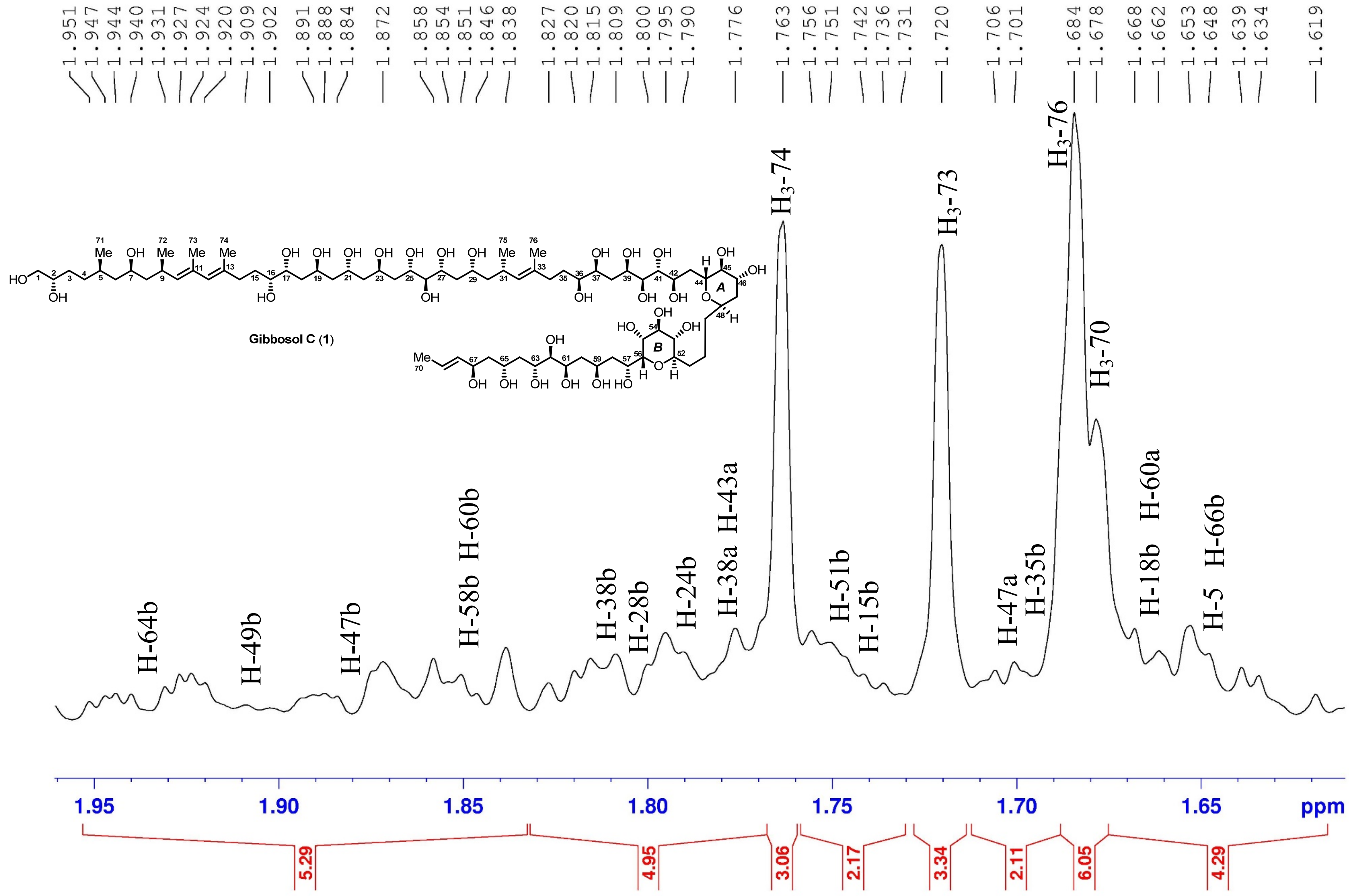

^{1}H (700 MHz) NMR spectrum of compound **1** in CD_3OD

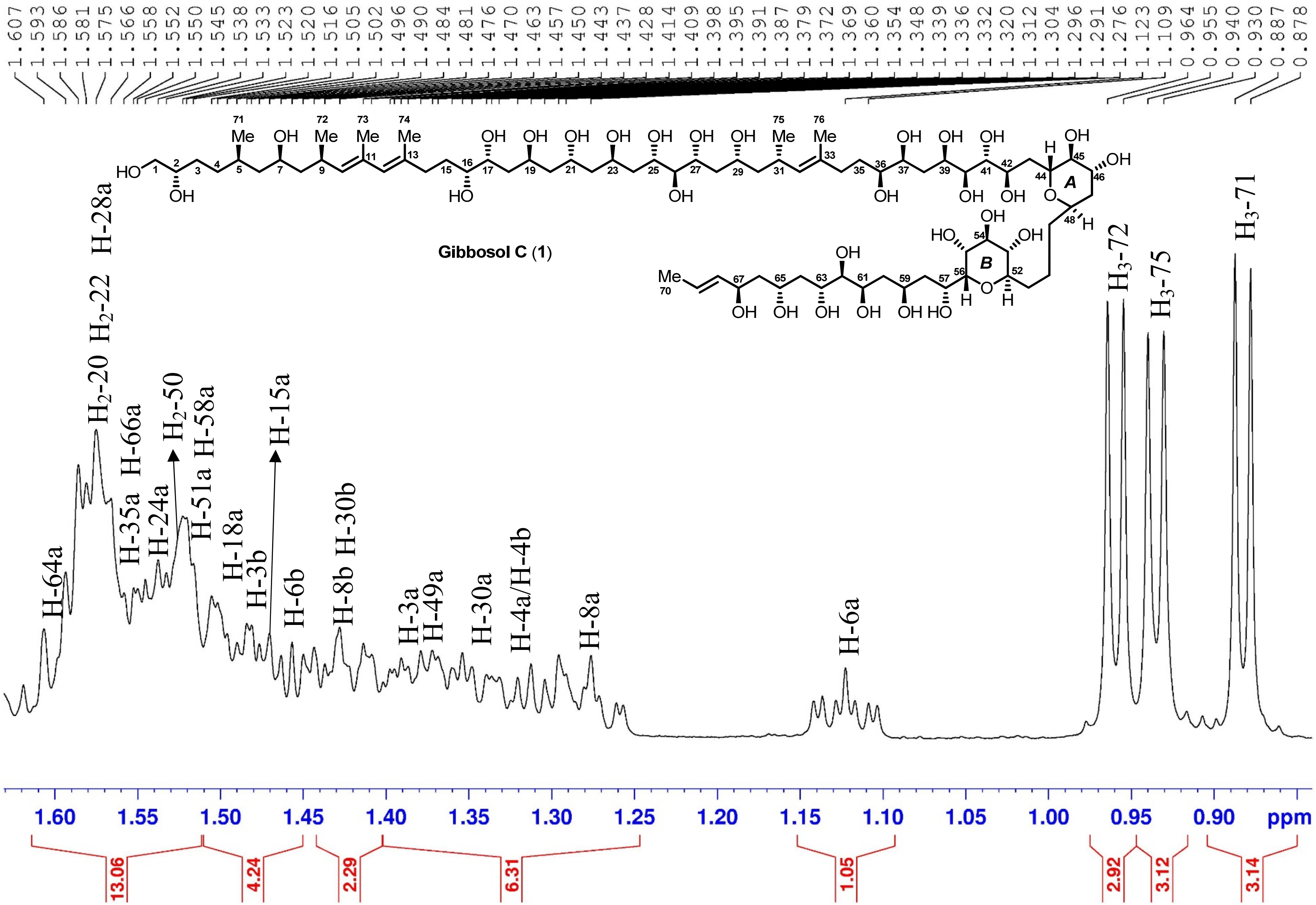

^{13}C (175 MHz) NMR spectrum of compound **1** in CD_3OD

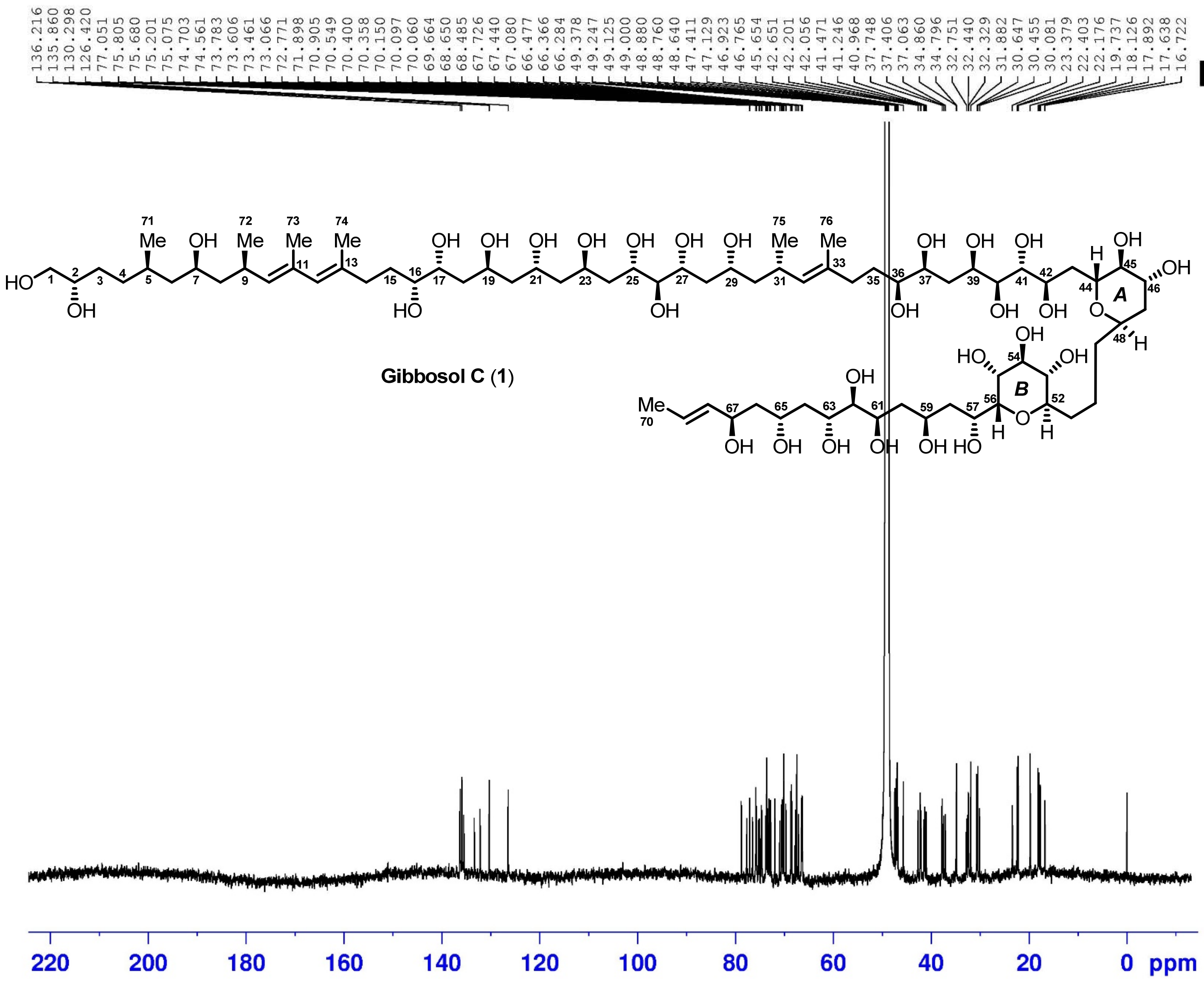

NAME	liwanshan-40-2-2
EXPNO	13
PROCNO	1
Date_	20190507
Time	23.49 h
INSTRUM	spect
PROBHD	Z120187_0028 (
PULPROG	zgpg30
TD	32768
SOLVENT	MeOD
NS	8000
DS	8
SWH	43859.648 H:
FIDRES	2.676980 H:
AQ	0.3736052 s‹
RG	181.26
DW	11.400 u:
DE	18.00 u:
TE	298.0 K
D1	1.00000000 s‹
D11	0.03000000 s‹
TD0	1
SFO1	176.0797677 MI
NUC1	13C
P1	11.90 u:
SI	32768
SF	176.0601526 MI
WDW	EM
SSB	0
LB	5.00 H:
GB	0
PC	1.40

^{13}C (175 MHz) NMR spectrum of compound **1** in CD_3OD

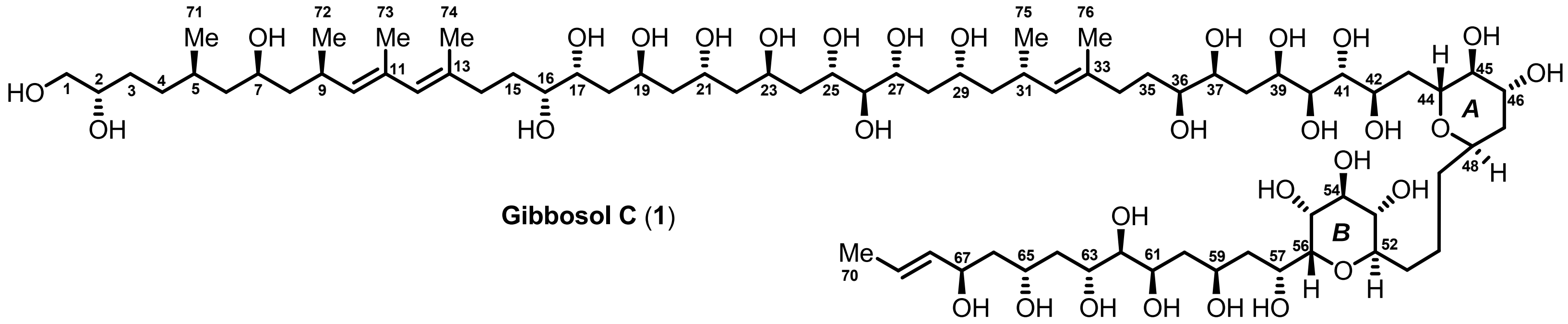

Gibbosol C (**1**)

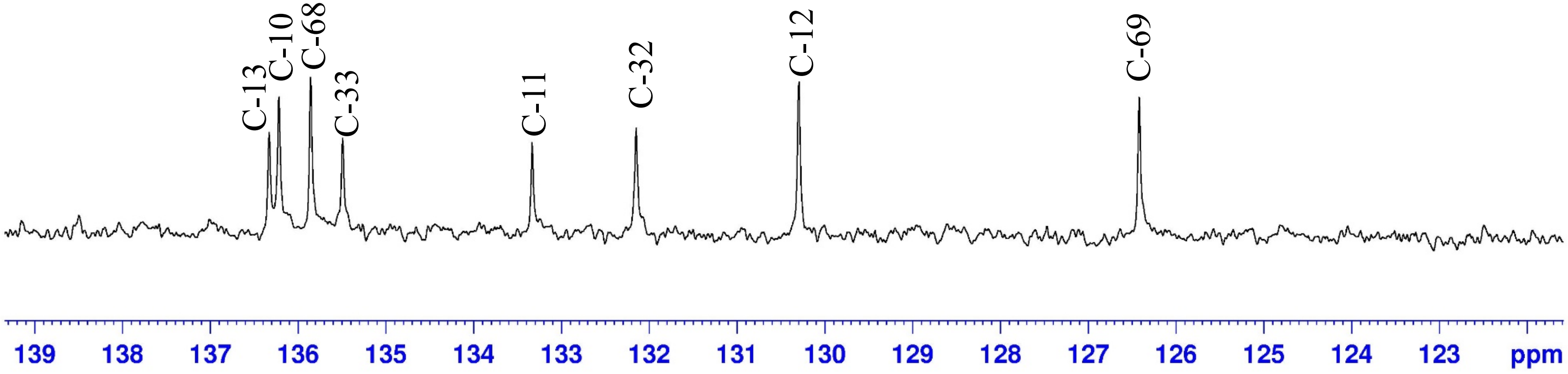

^{13}C (175 MHz) NMR spectrum of compound **1** in CD_3OD

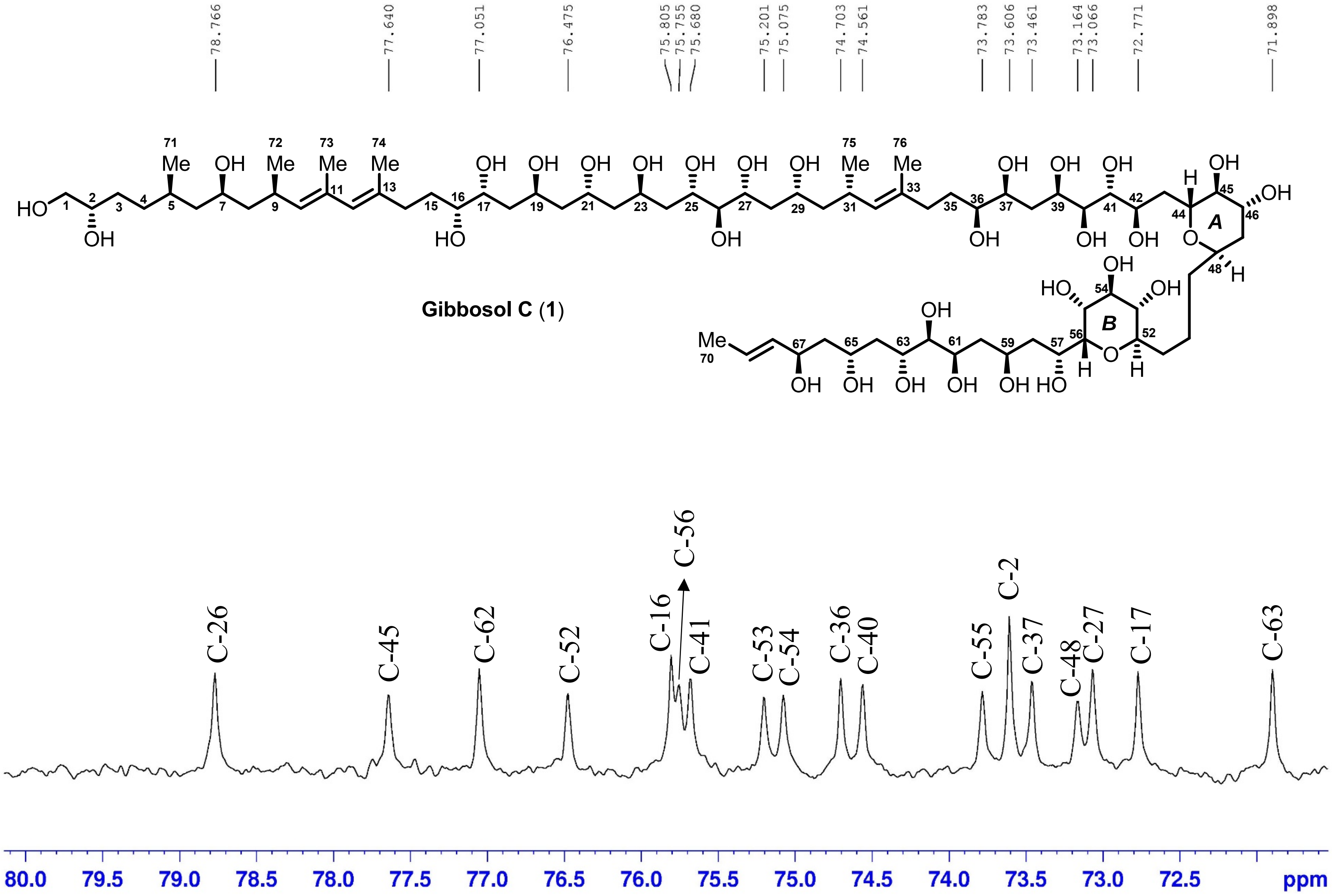

^{13}C (175 MHz) NMR spectrum of compound **1** in CD_3OD

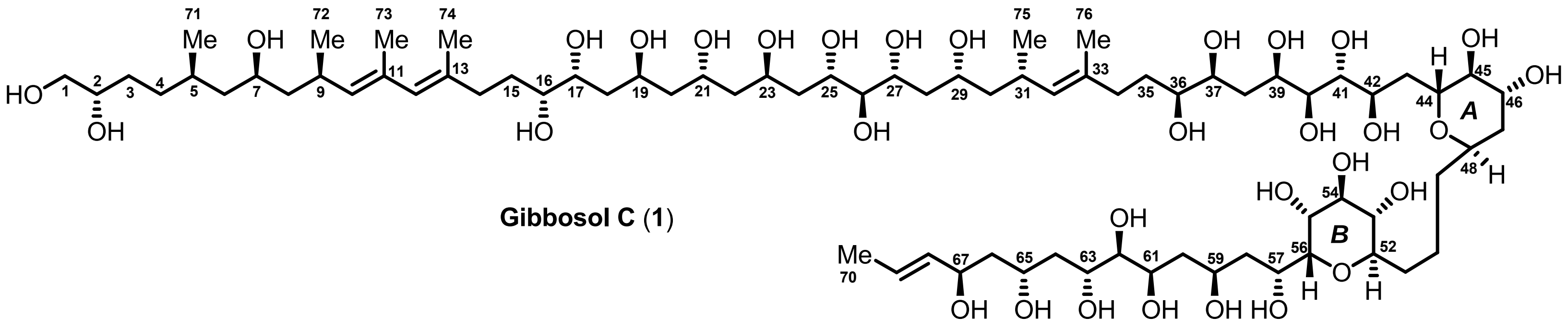

Gibbosol C (**1**)

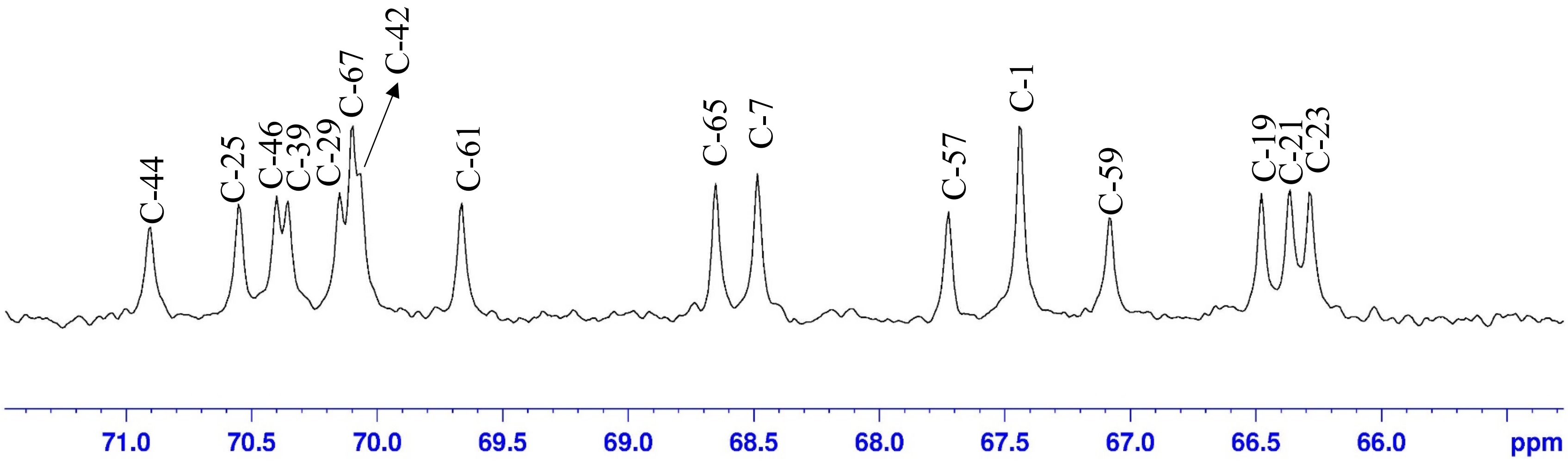

^{13}C (175 MHz) NMR spectrum of compound **1** in CD_3OD

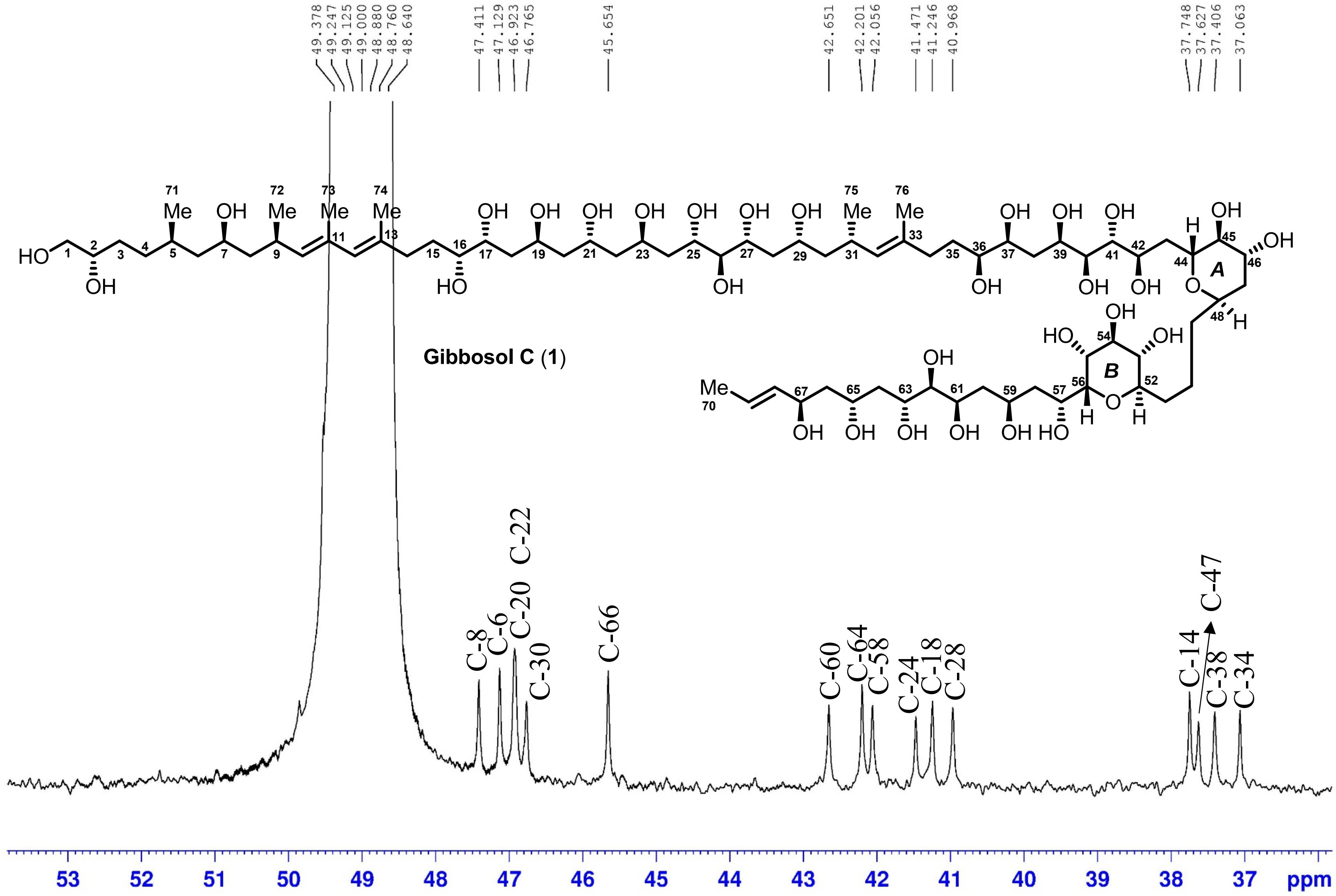

^{13}C (175 MHz) NMR spectrum of compound **1** in CD_3OD

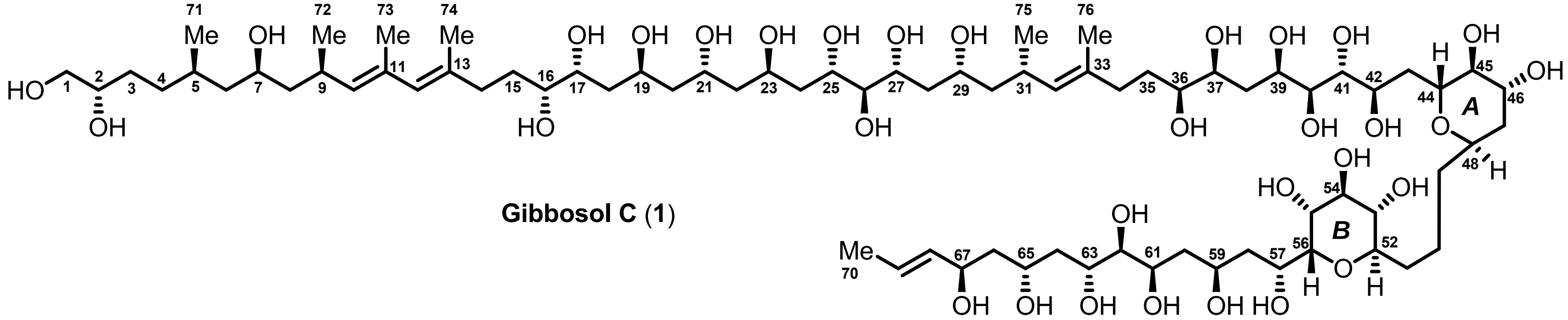

Gibbosol C (1)

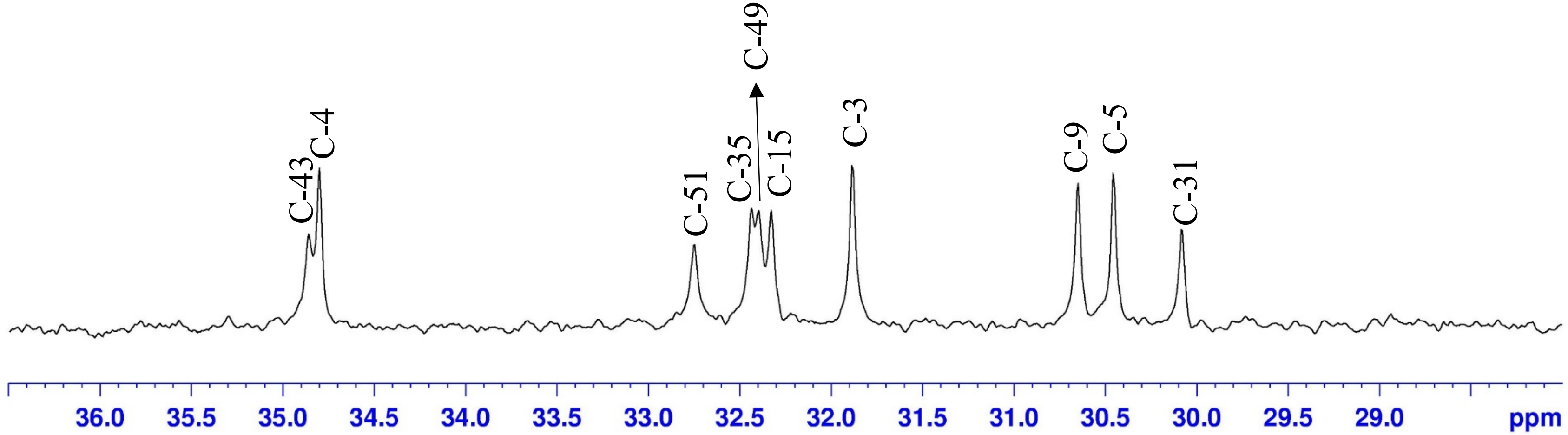

^{13}C (175 MHz) NMR spectrum of compound **1** in CD_3OD

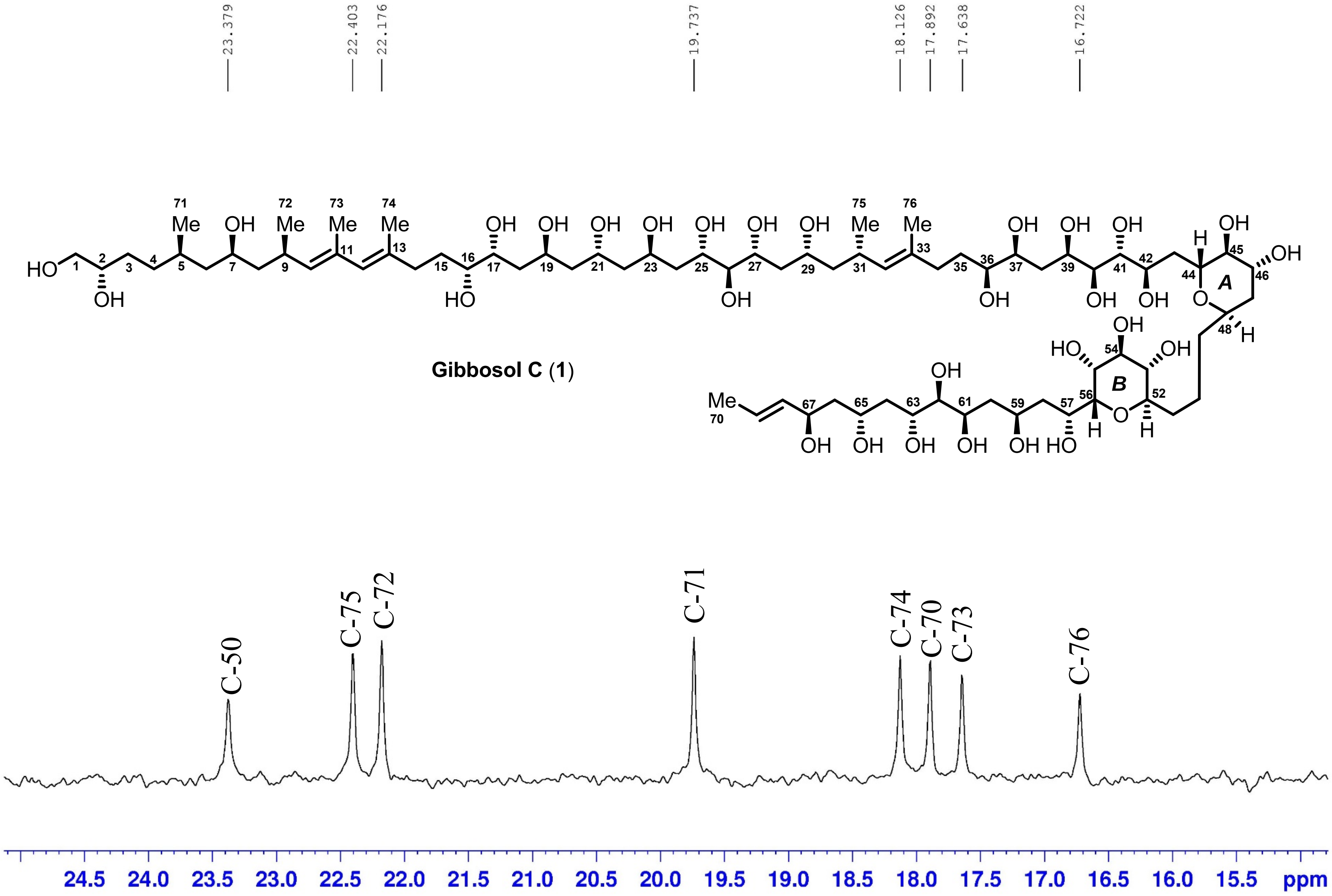

DEPT135 (175 MHz) spectrum of compound **1** in CD_3OD

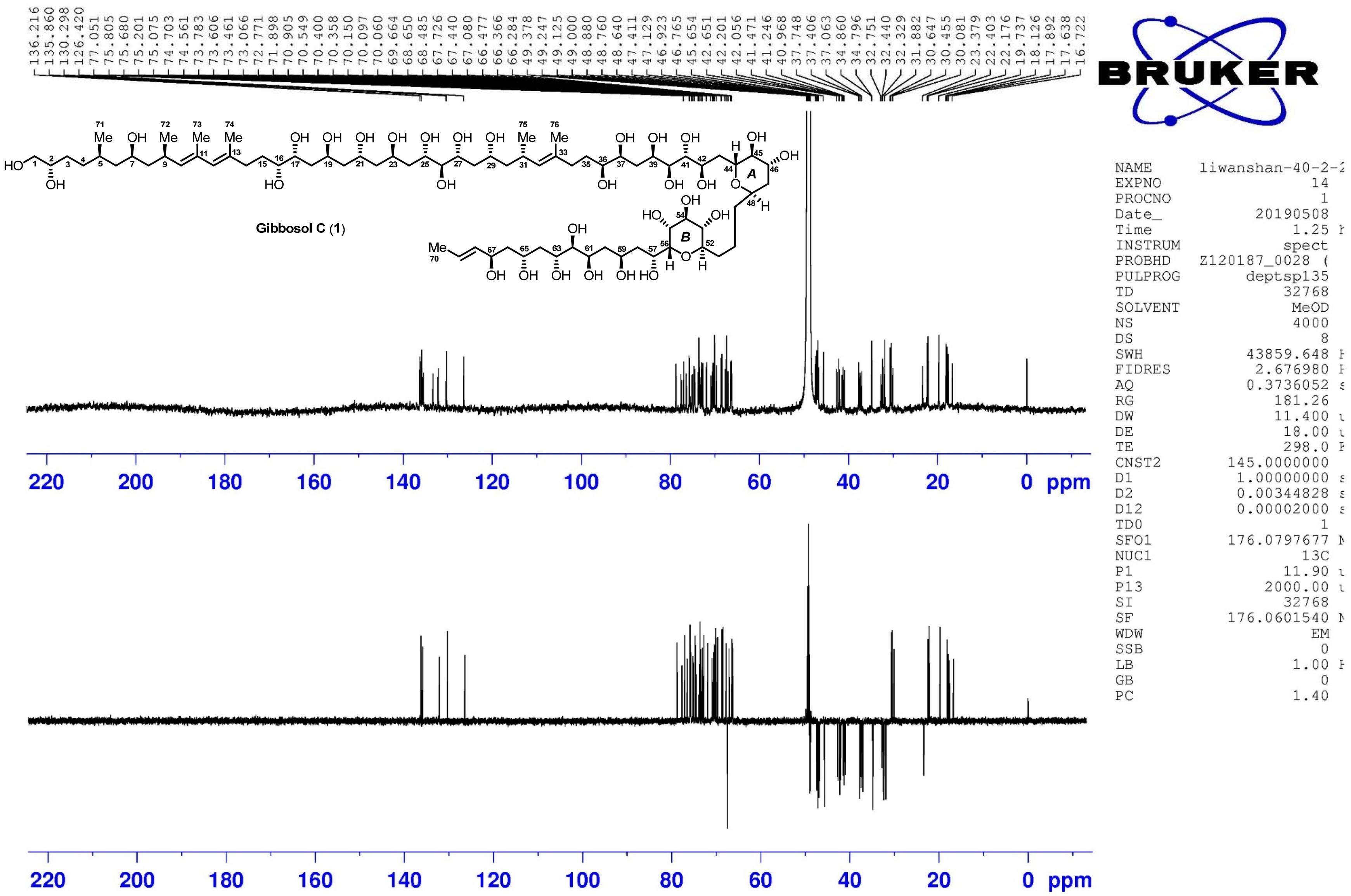

DEPT135 (175 MHz) spectrum of compound **1** in CD_3OD

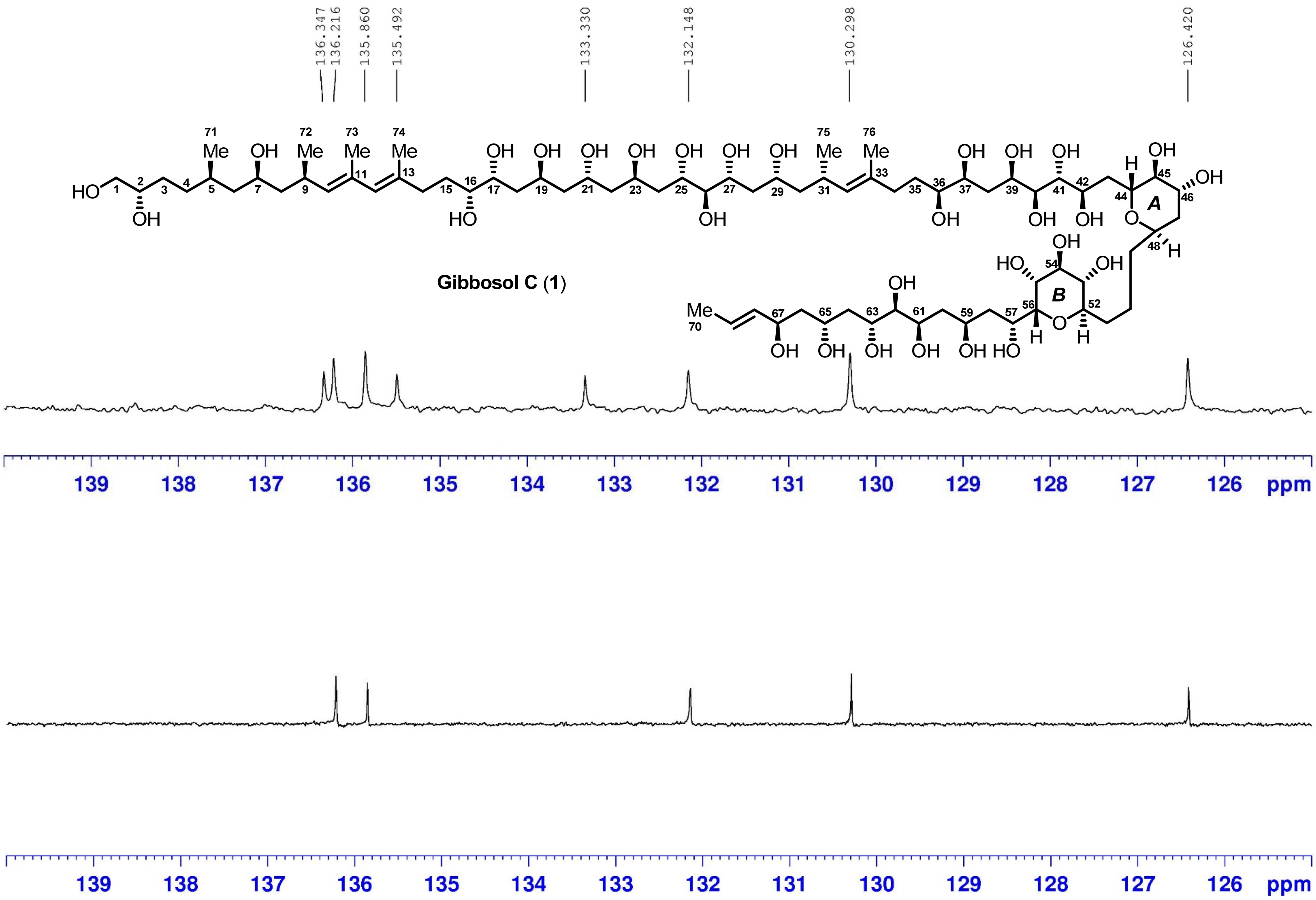

DEPT135 (175 MHz) spectrum of compound **1** in CD_3OD

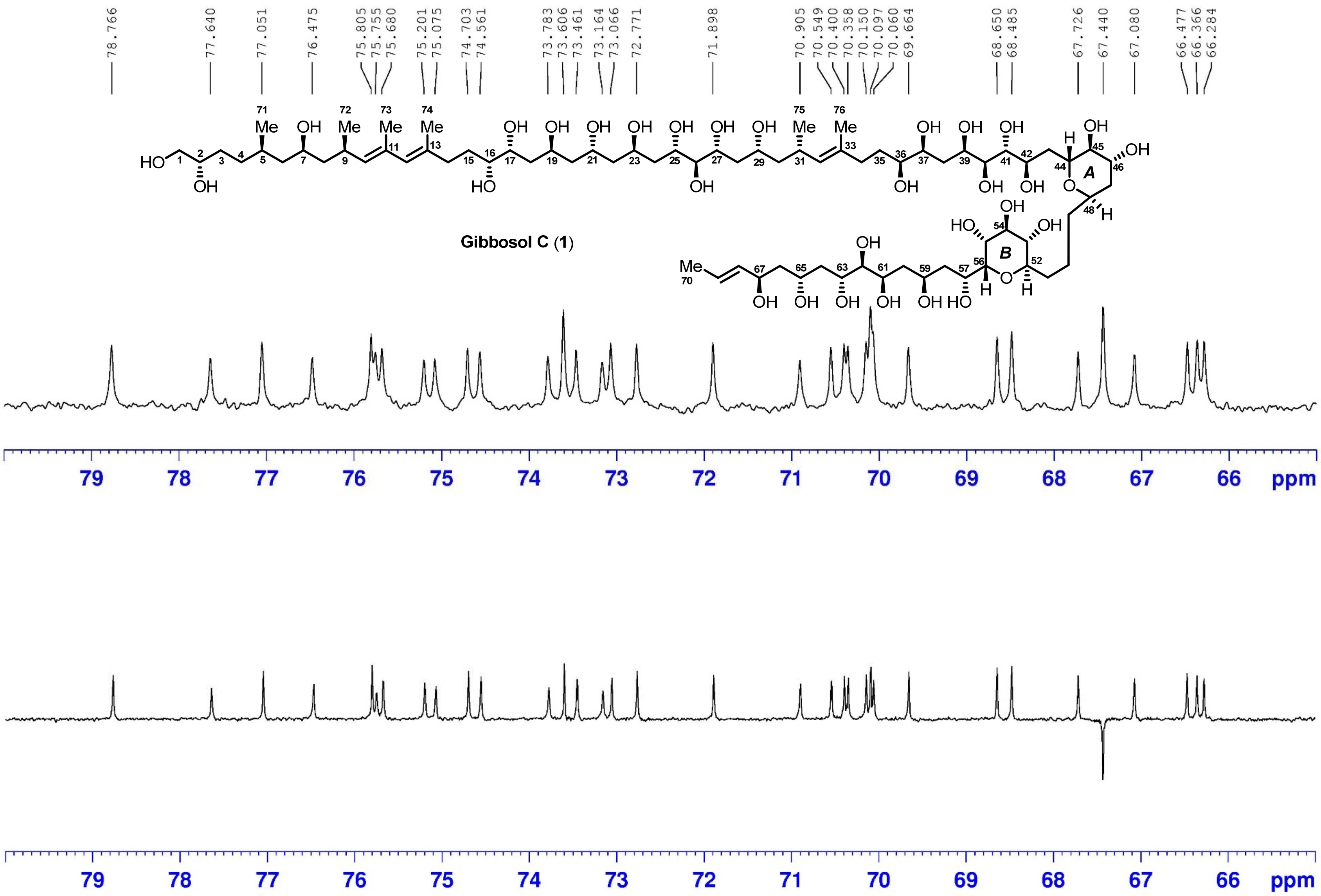

DEPT135 (175 MHz) spectrum of compound **1** in CD_3OD

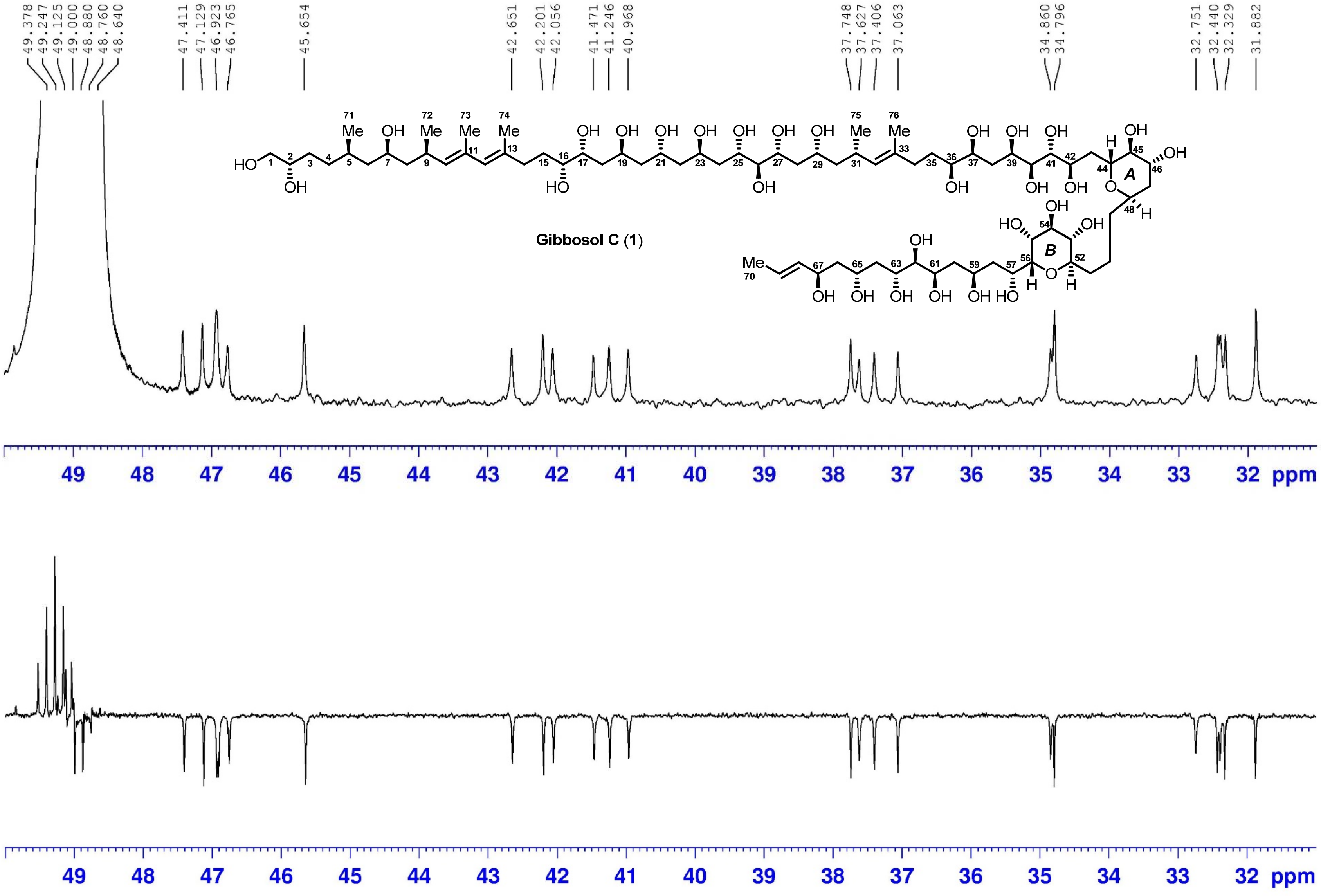

DEPT135 (175 MHz) spectrum of compound **1** in CD_3OD

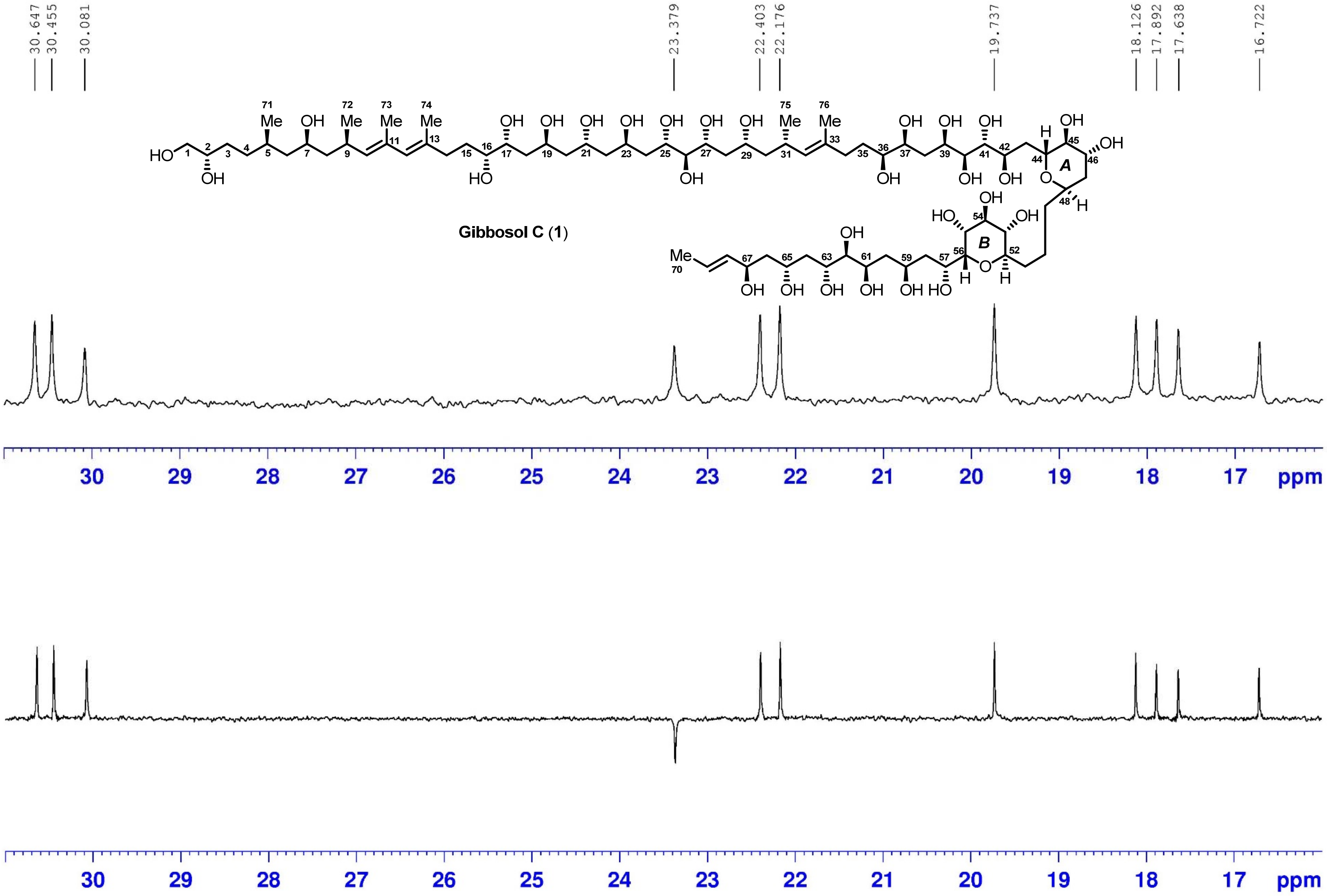

^{1}H–^{1}H COSY (700 MHz) spectrum of compound **1** in CD_3OD

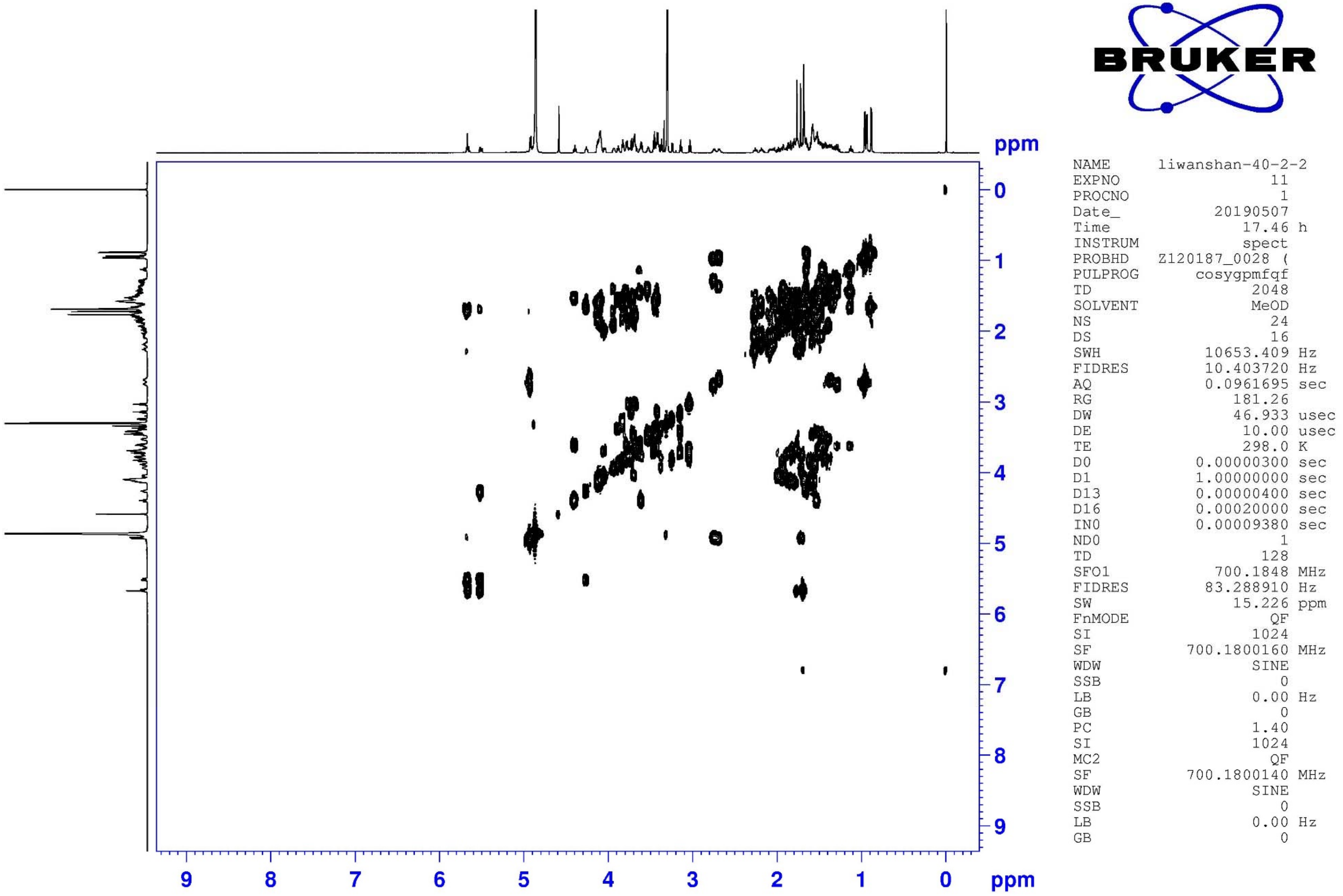

^1H–^1H COSY (700 MHz) spectrum of compound **1** in CD_3OD

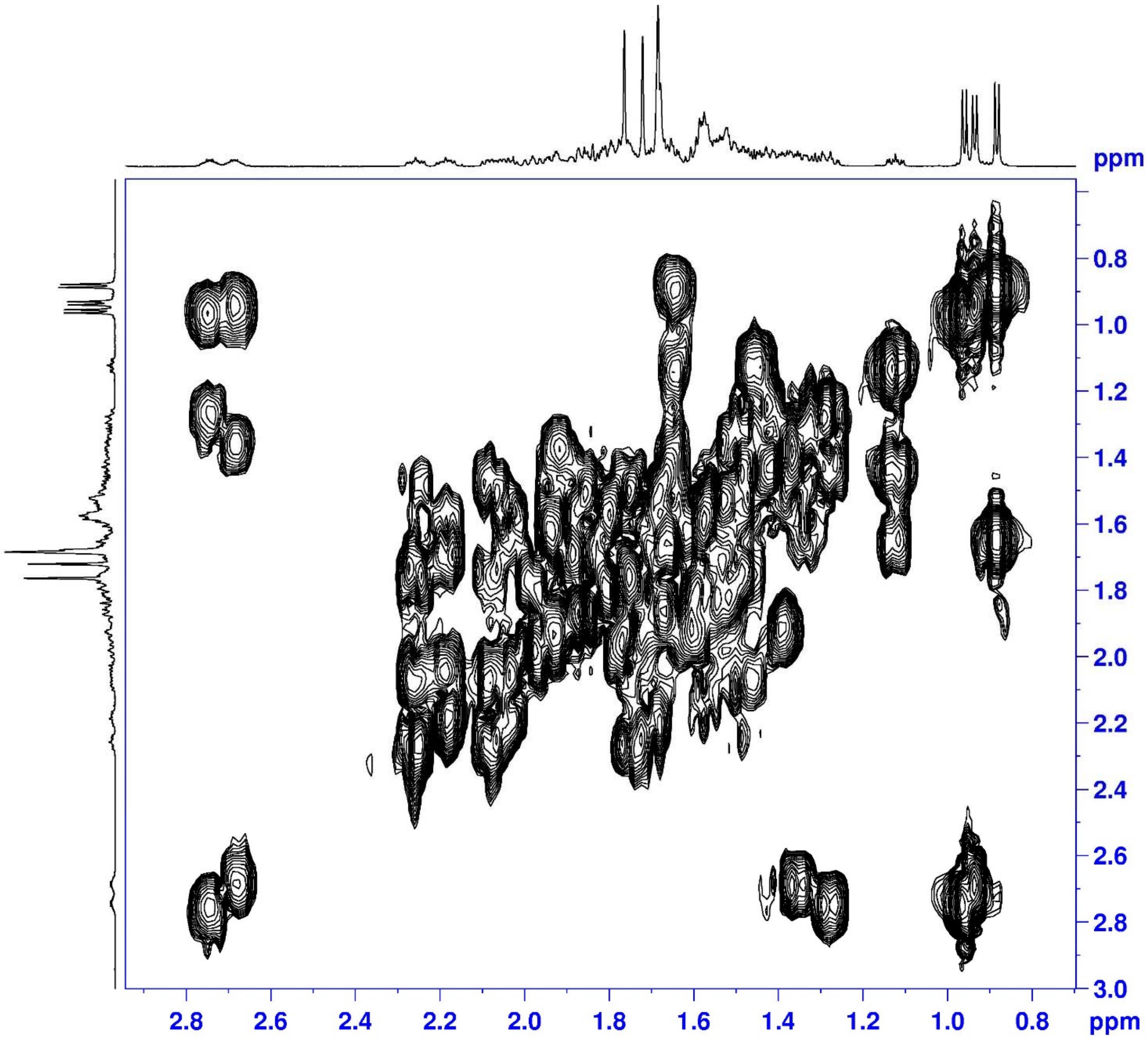

^{1}H–^{1}H COSY (700 MHz) spectrum of compound **1** in CD_3OD

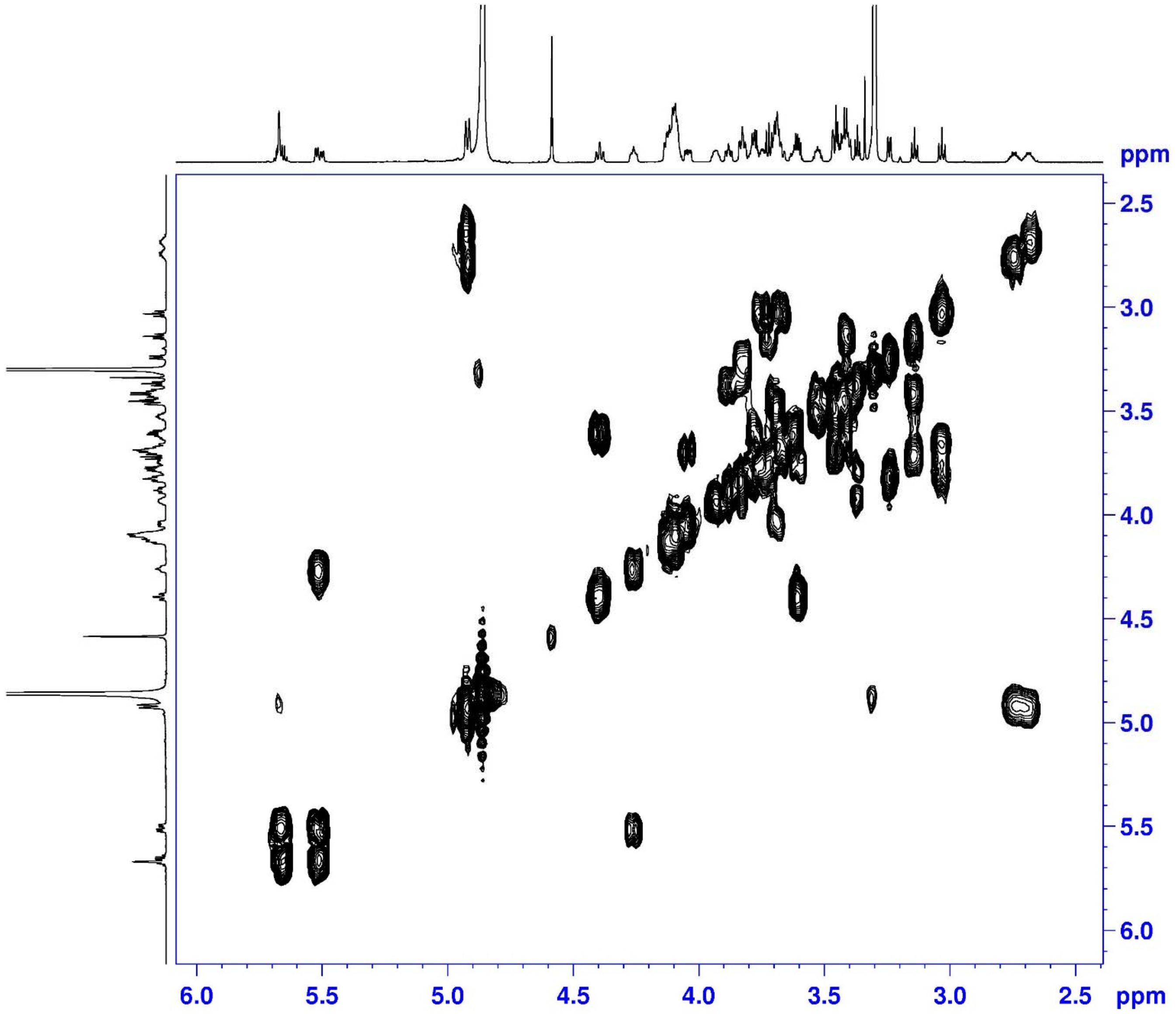

^1H–^1H COSY (700 MHz) spectrum of compound **1** in CD_3OD

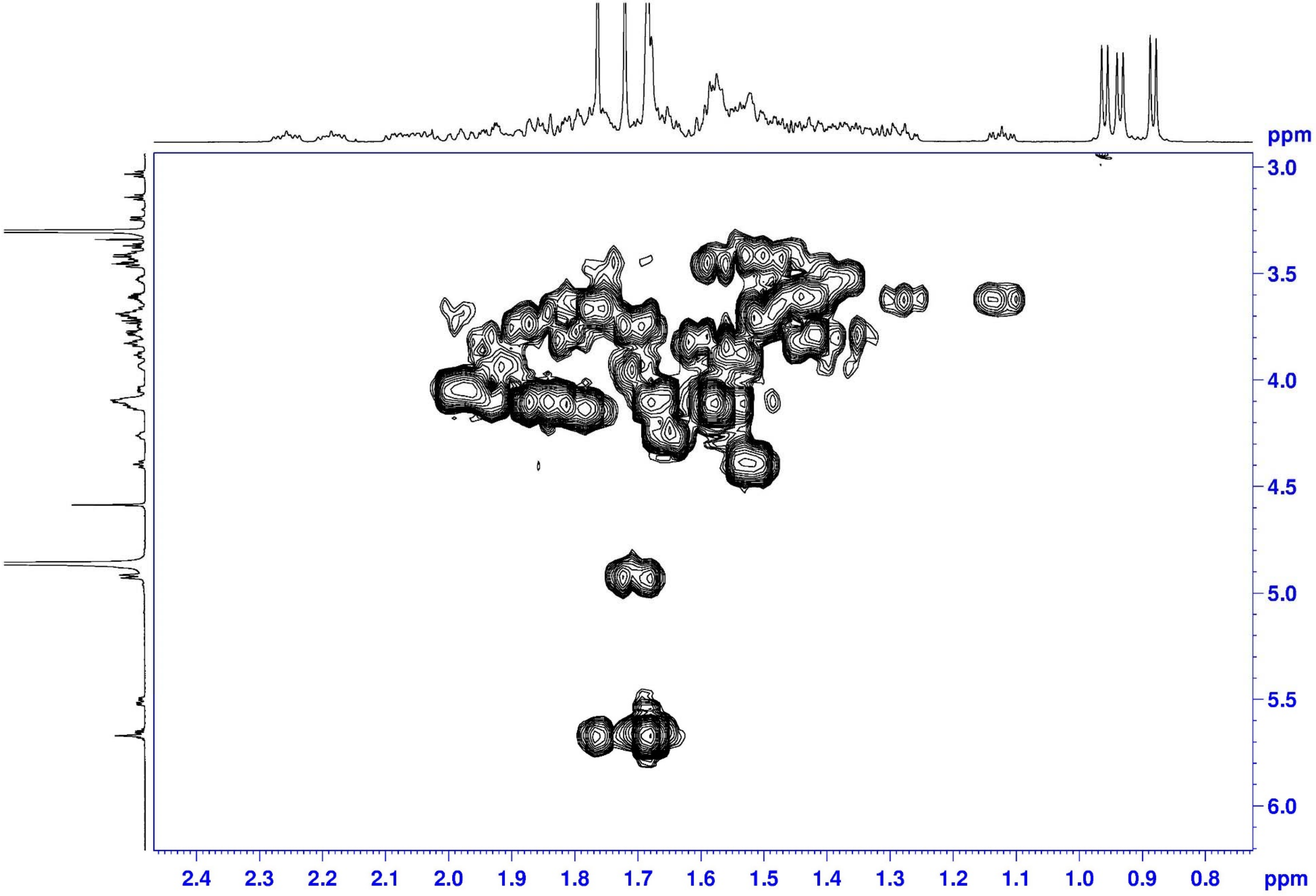

HSQC (700 MHz) spectrum of compound **1** in CD_3OD

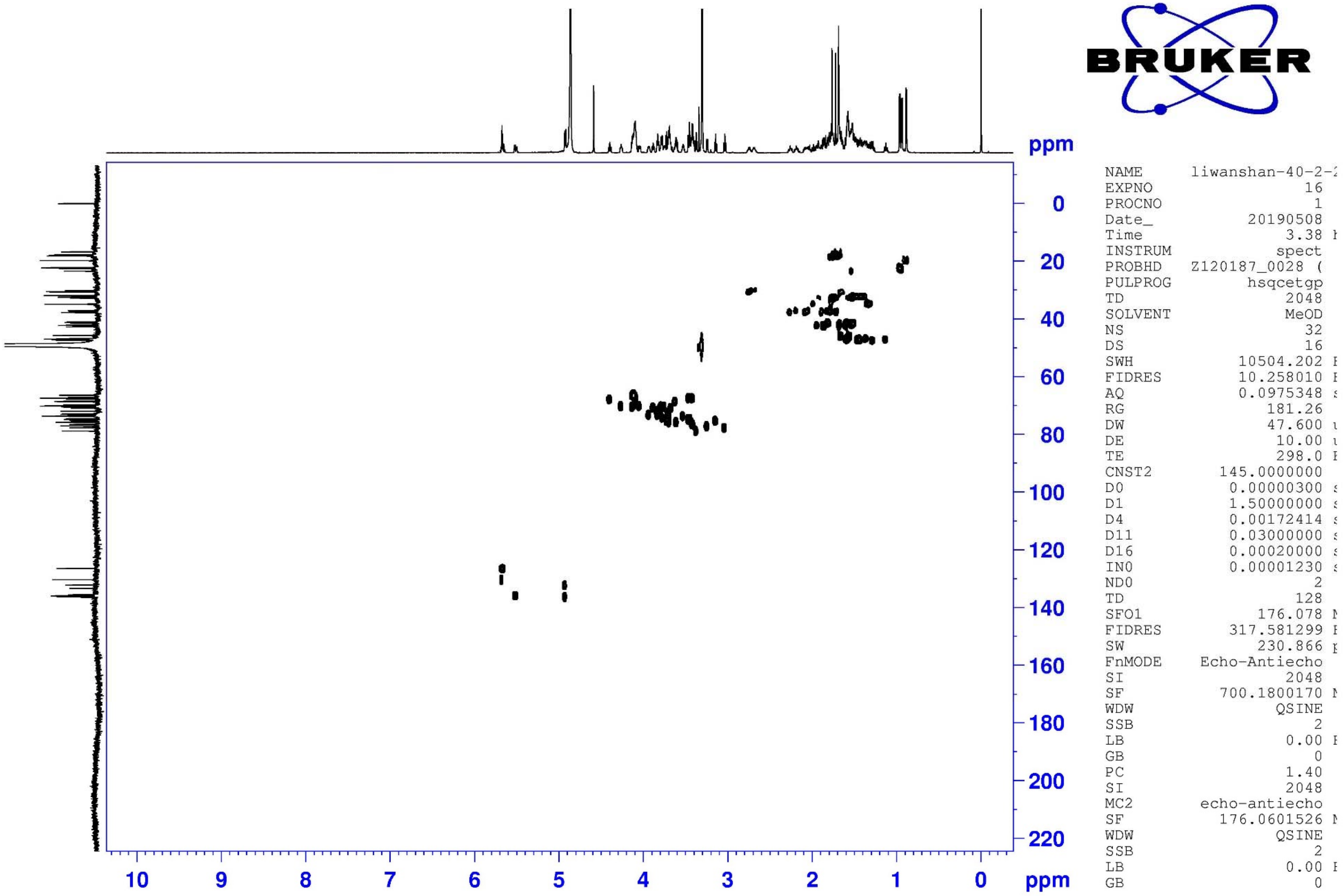

HSQC (700 MHz) spectrum of compound **1** in CD_3OD

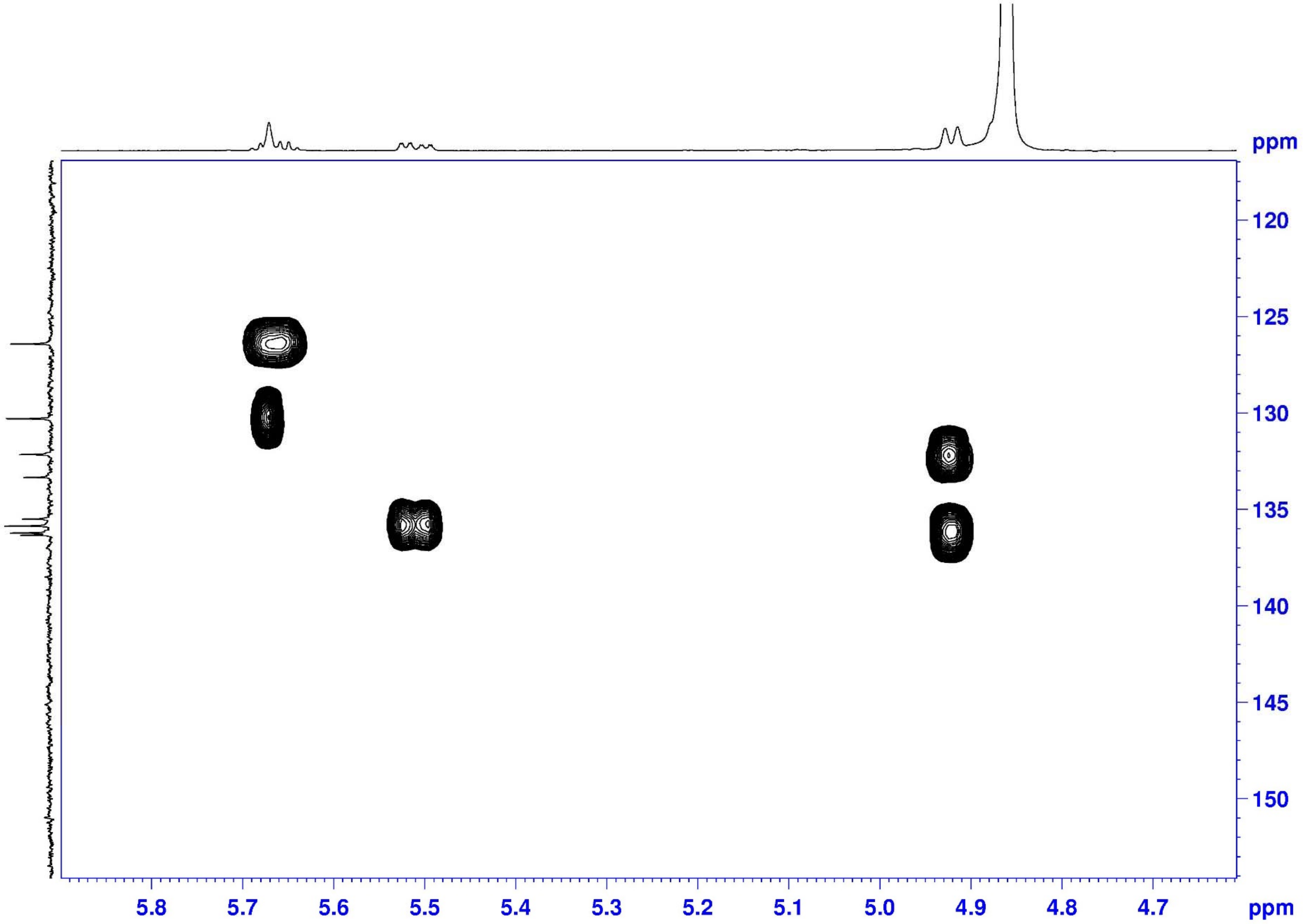

HSQC (700 MHz) spectrum of compound **1** in CD_3OD

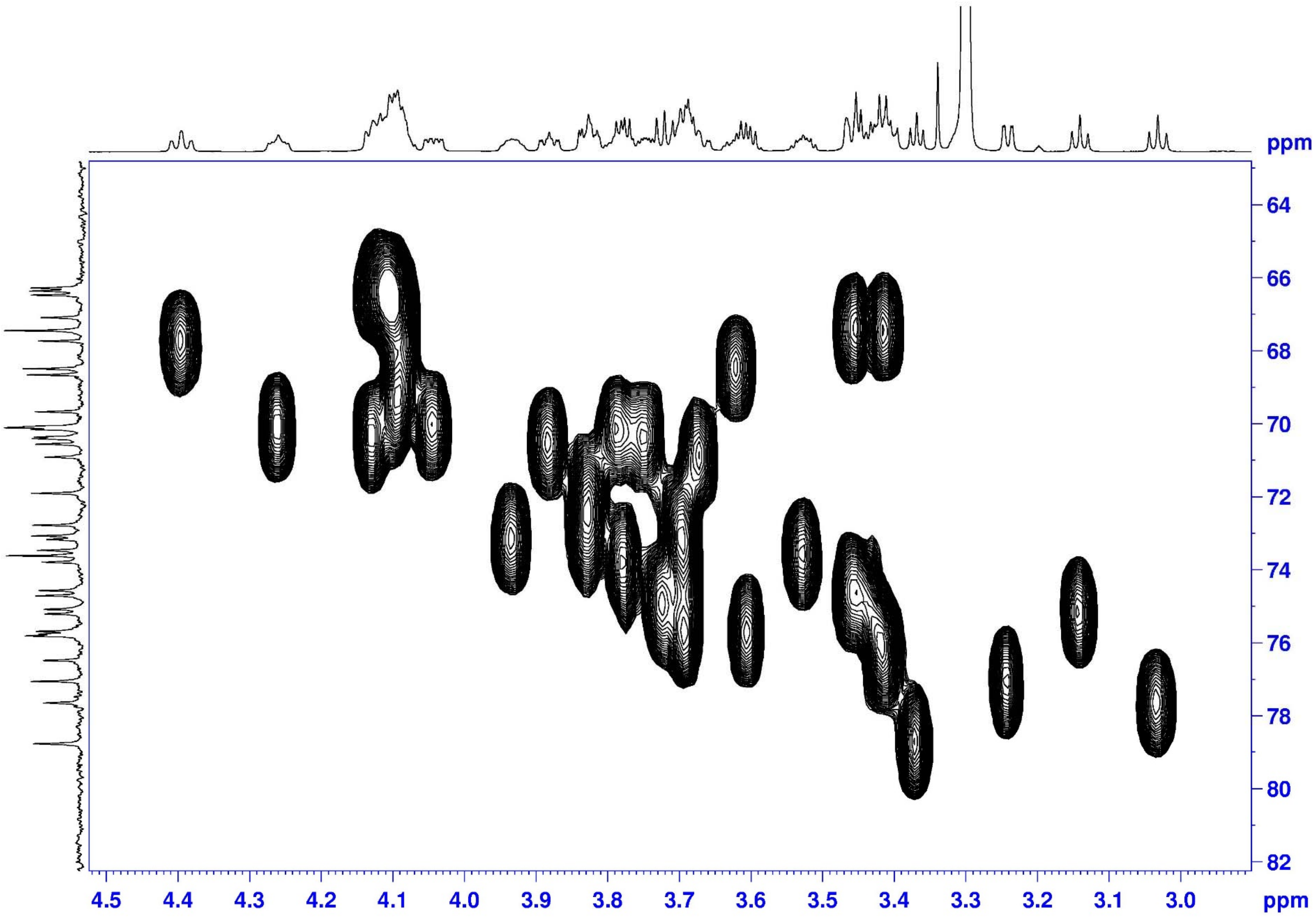

HSQC (700 MHz) spectrum of compound **1** in CD_3OD

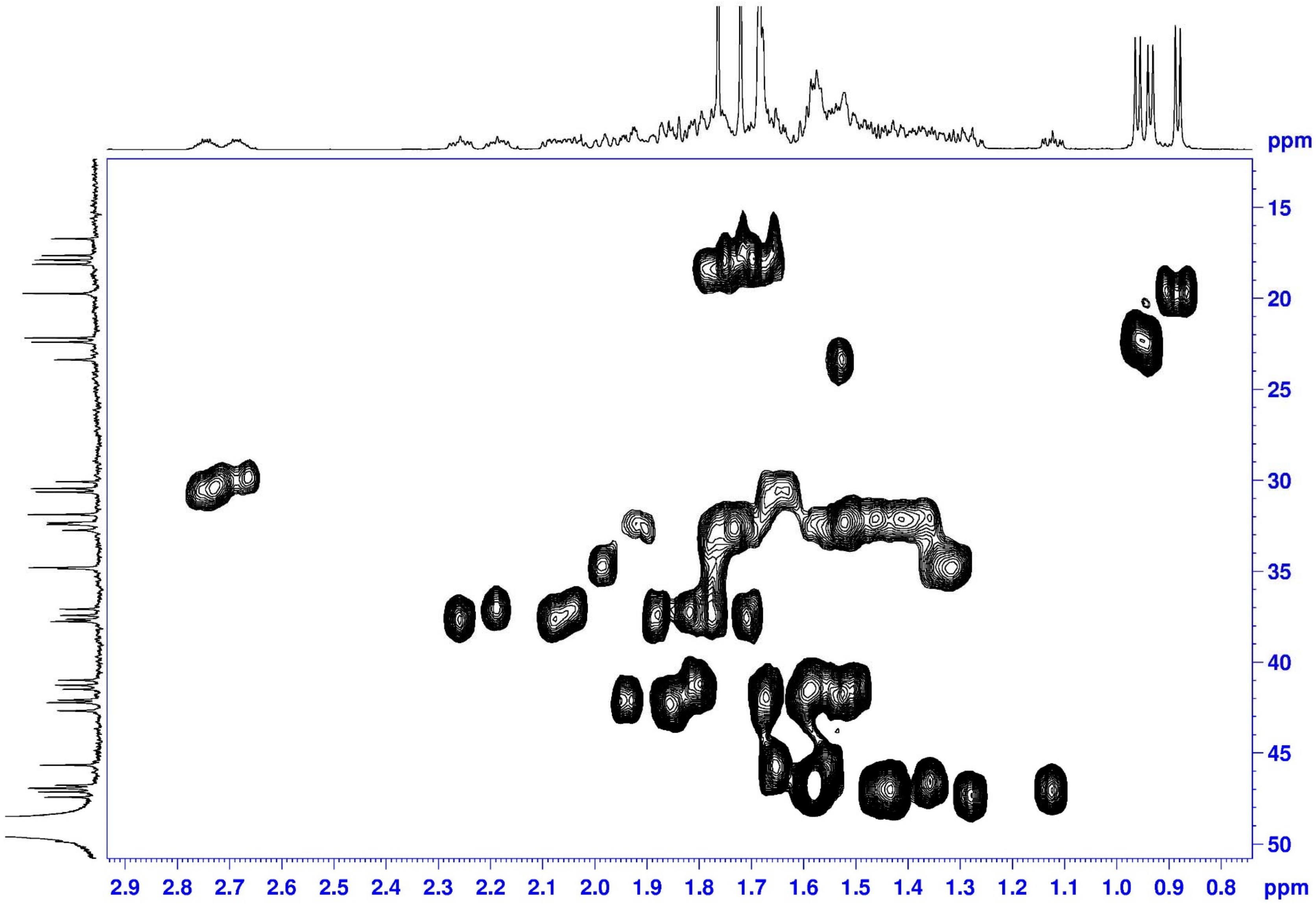

Selective-HSQC (700 MHz) spectrum of compound **1** in CD_3OD

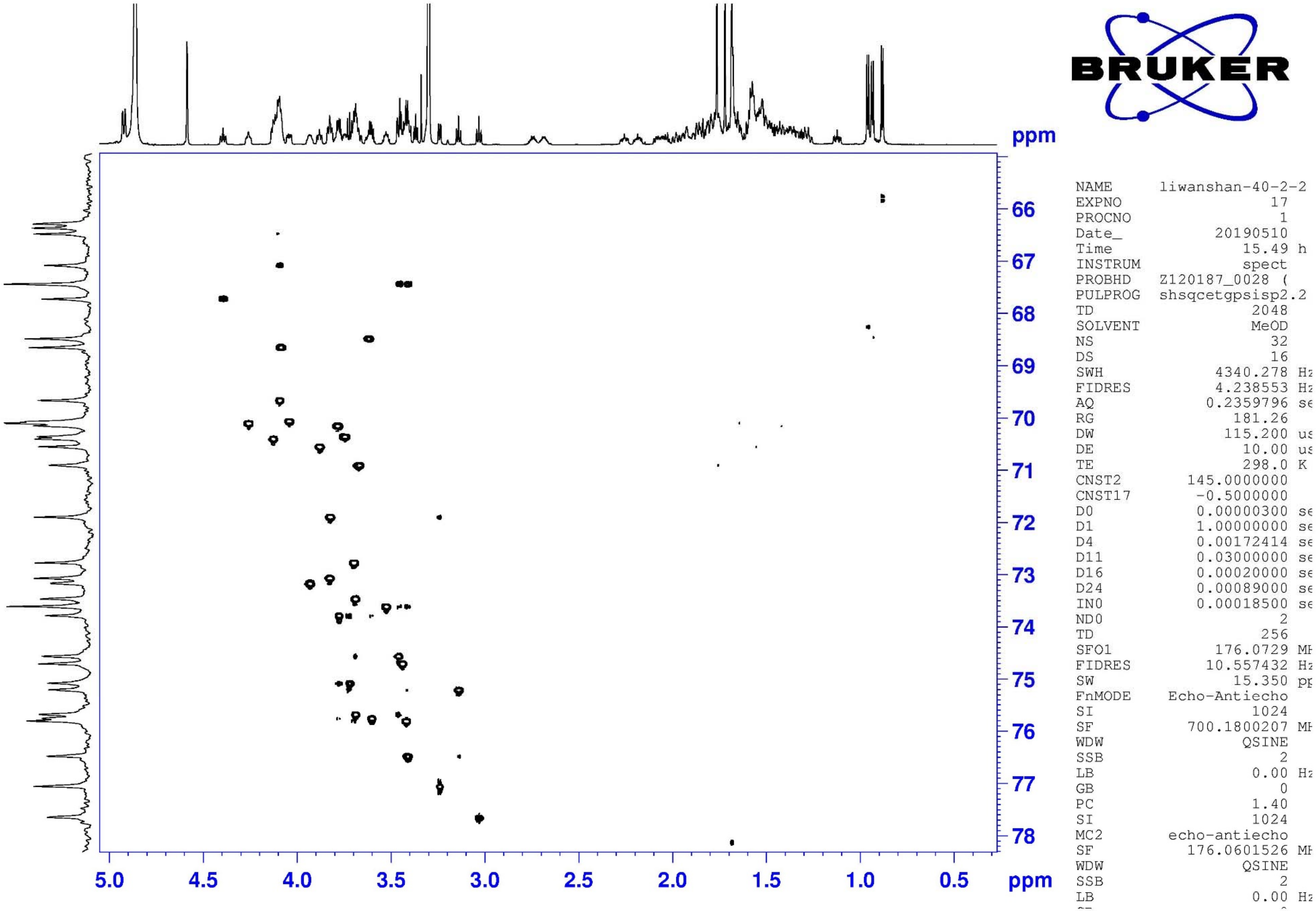

Selective-HSQC (700 MHz) spectrum of compound **1** in CD_3OD

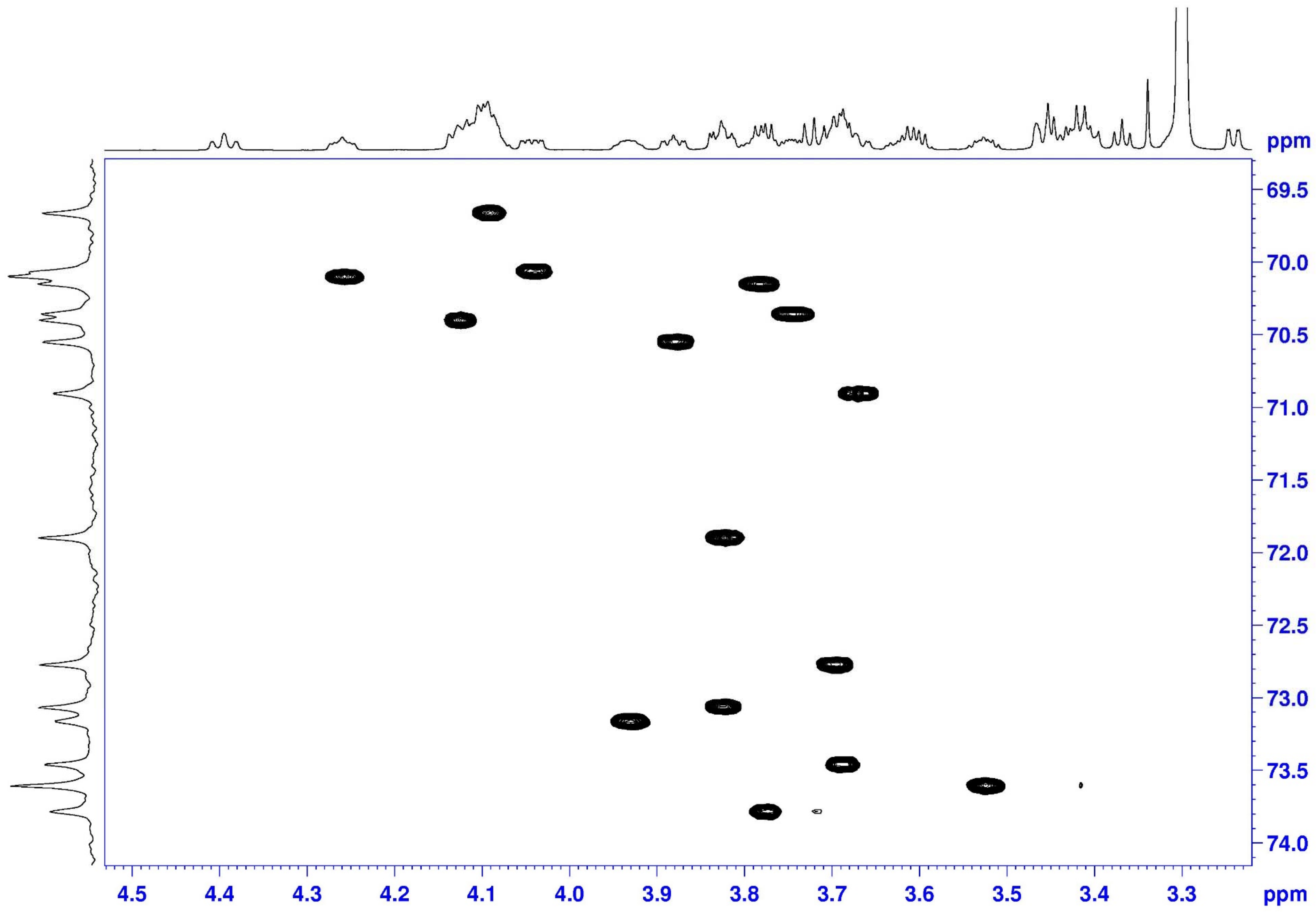

Selective-HSQC (700 MHz) spectrum of compound **1** in CD_3OD

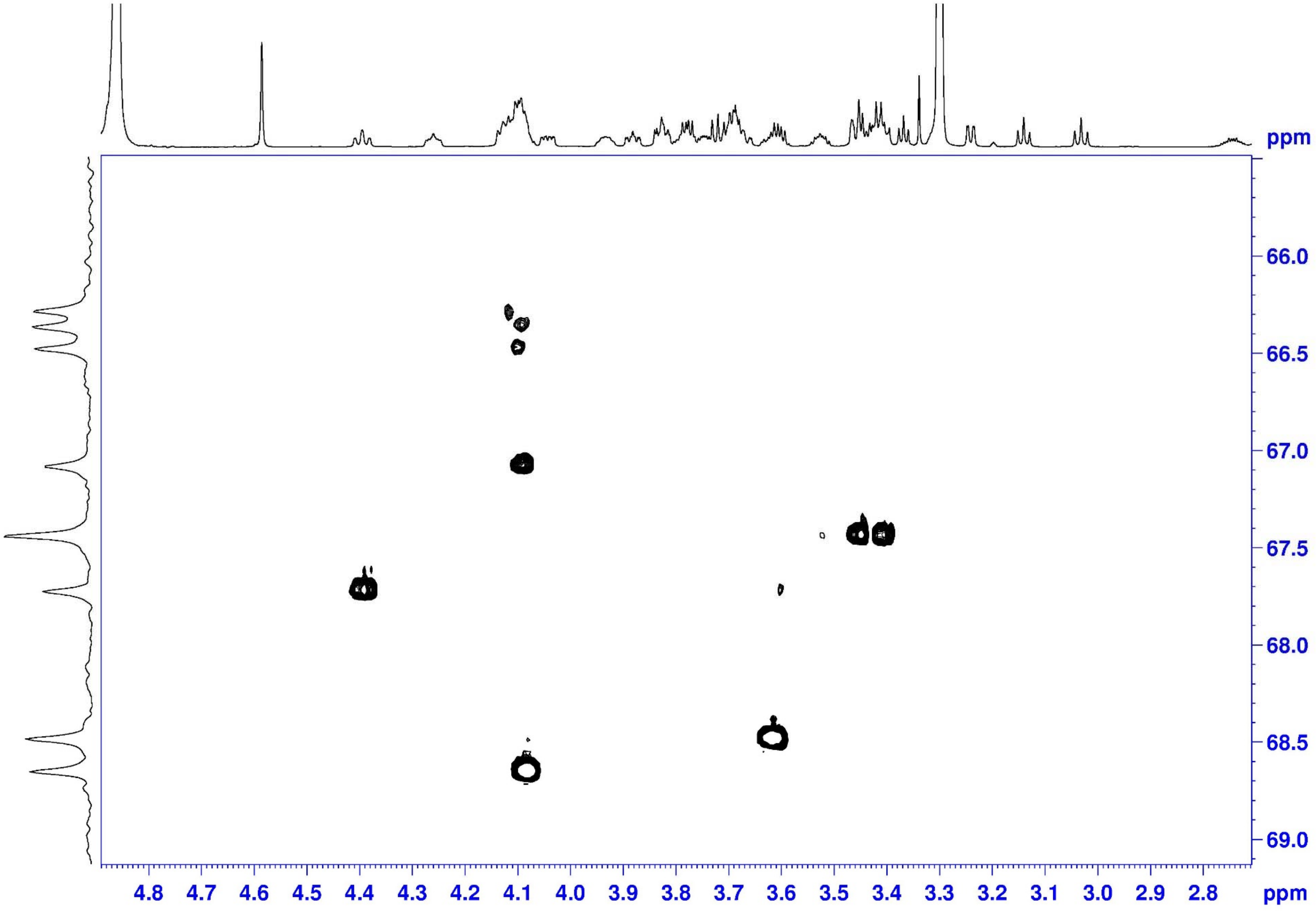

Selective-HSQC (700 MHz) spectrum of compound **1** in CD_3OD

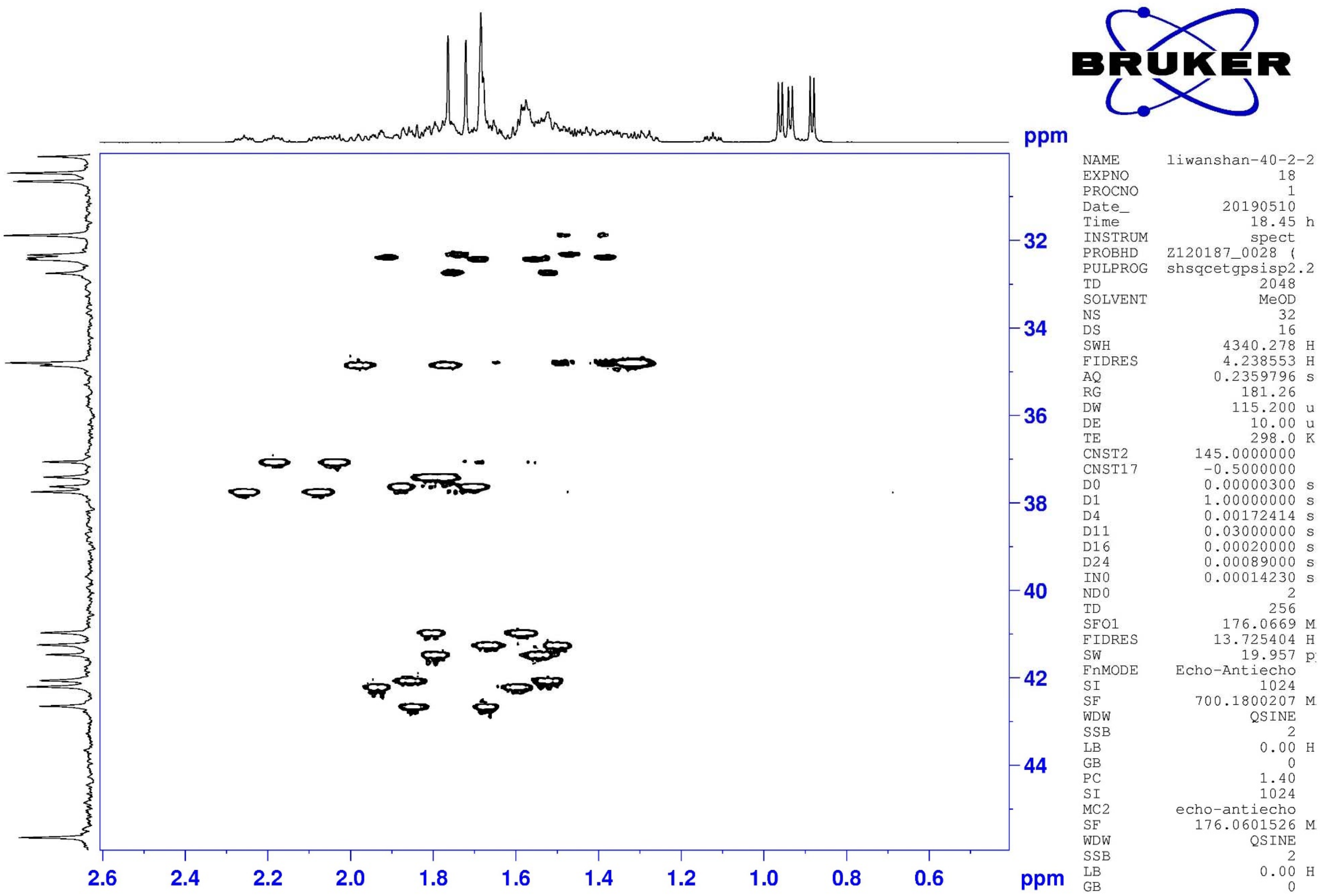

Selective-HSQC (700 MHz) spectrum of compound **1** in CD_3OD

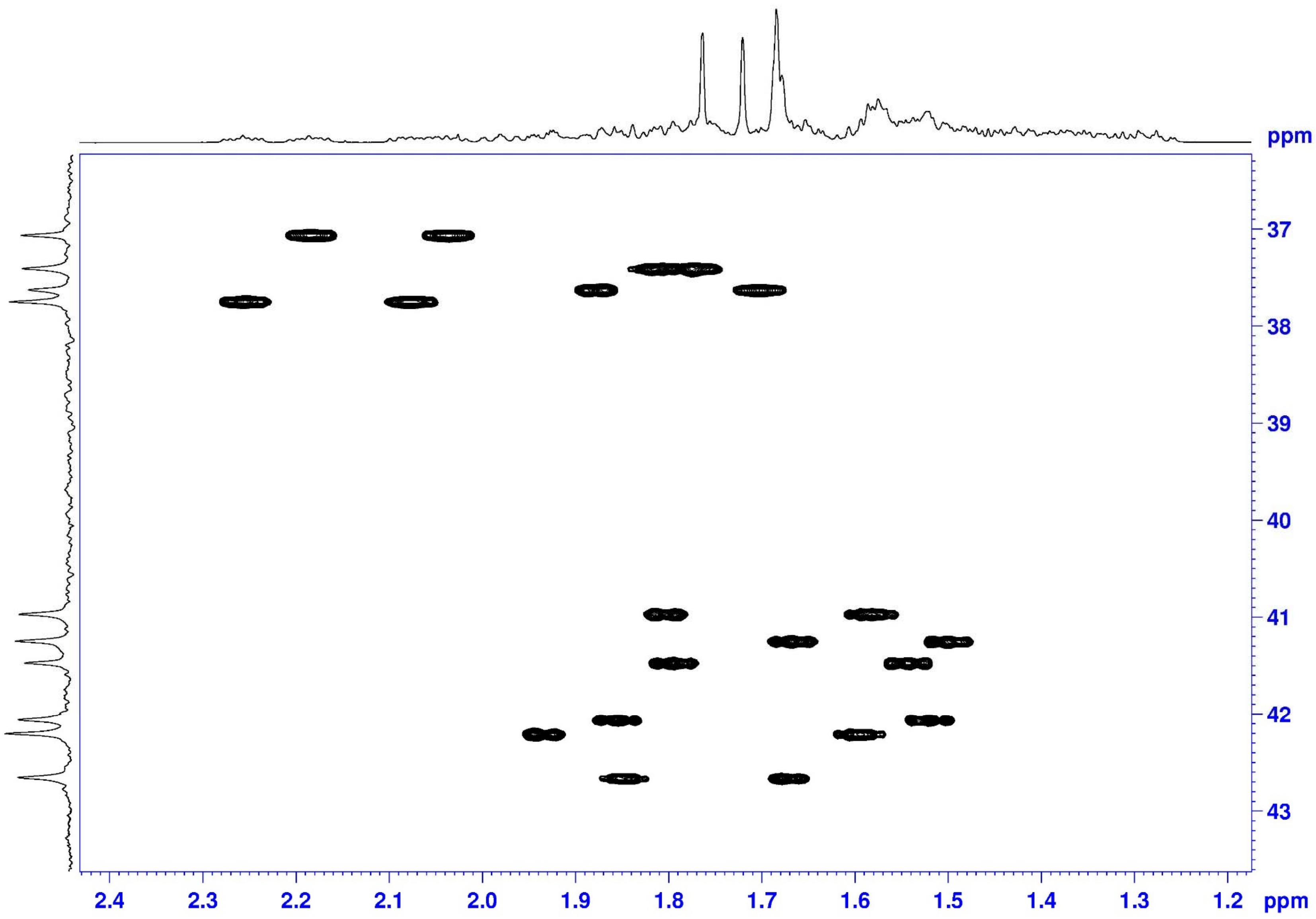

Selective-HSQC (700 MHz) spectrum of compound **1** in CD_3OD

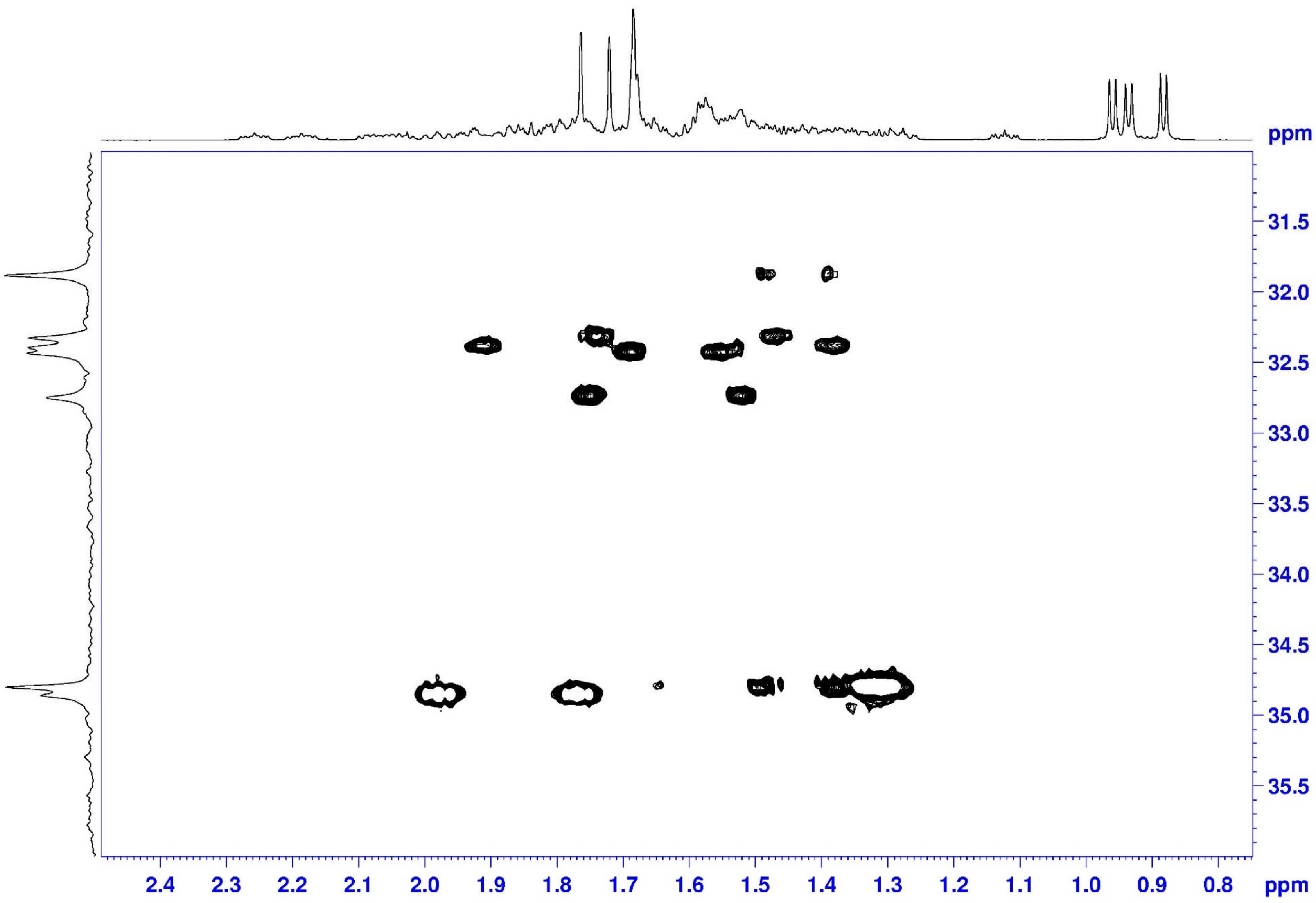

Selective-HSQC (700 MHz) spectrum of compound **1** in CD_3OD

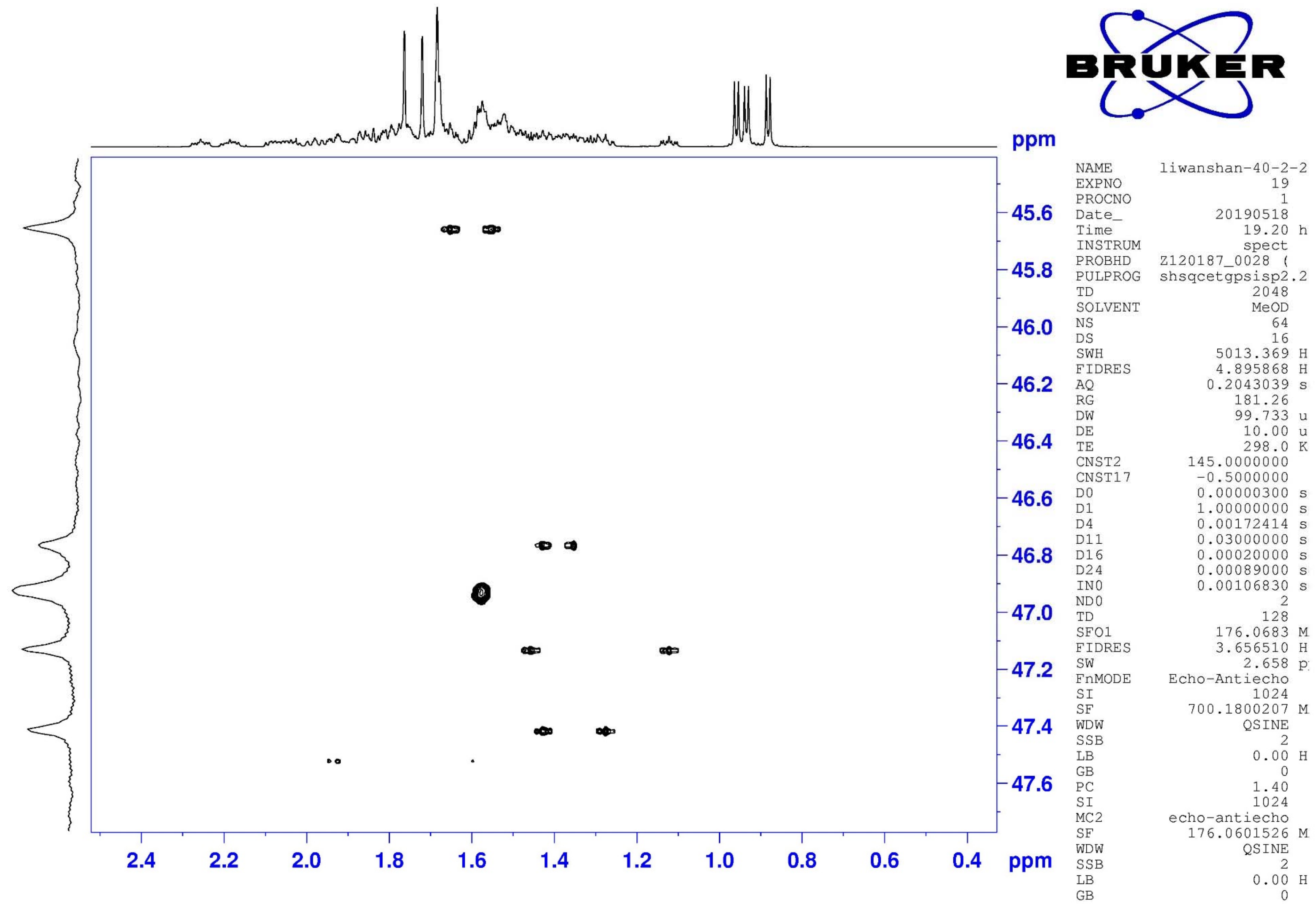

HMBC (700 MHz) spectrum of compound **1** in CD_3OD

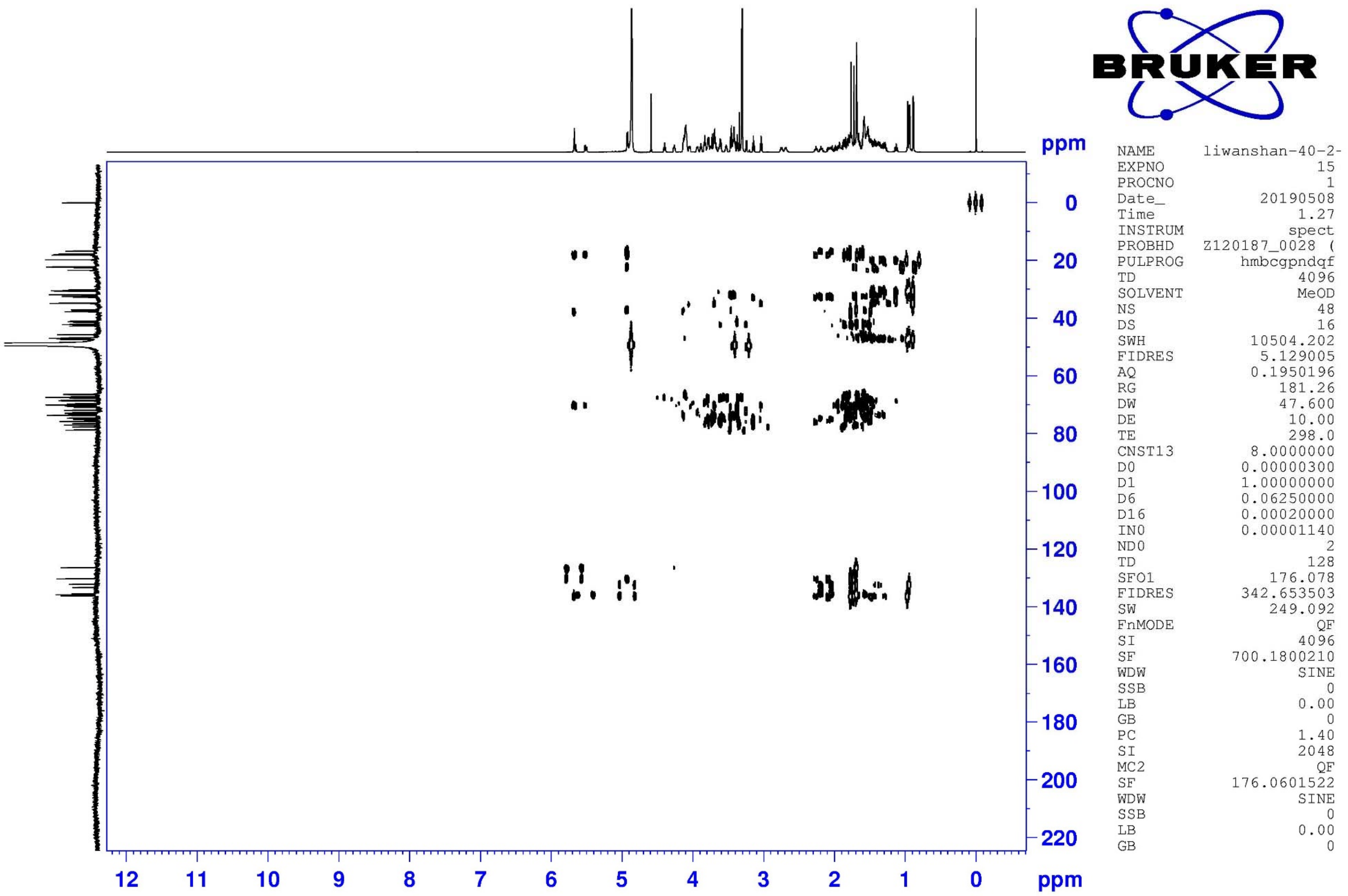

HMBC (700 MHz) spectrum of compound **1** in CD_3OD

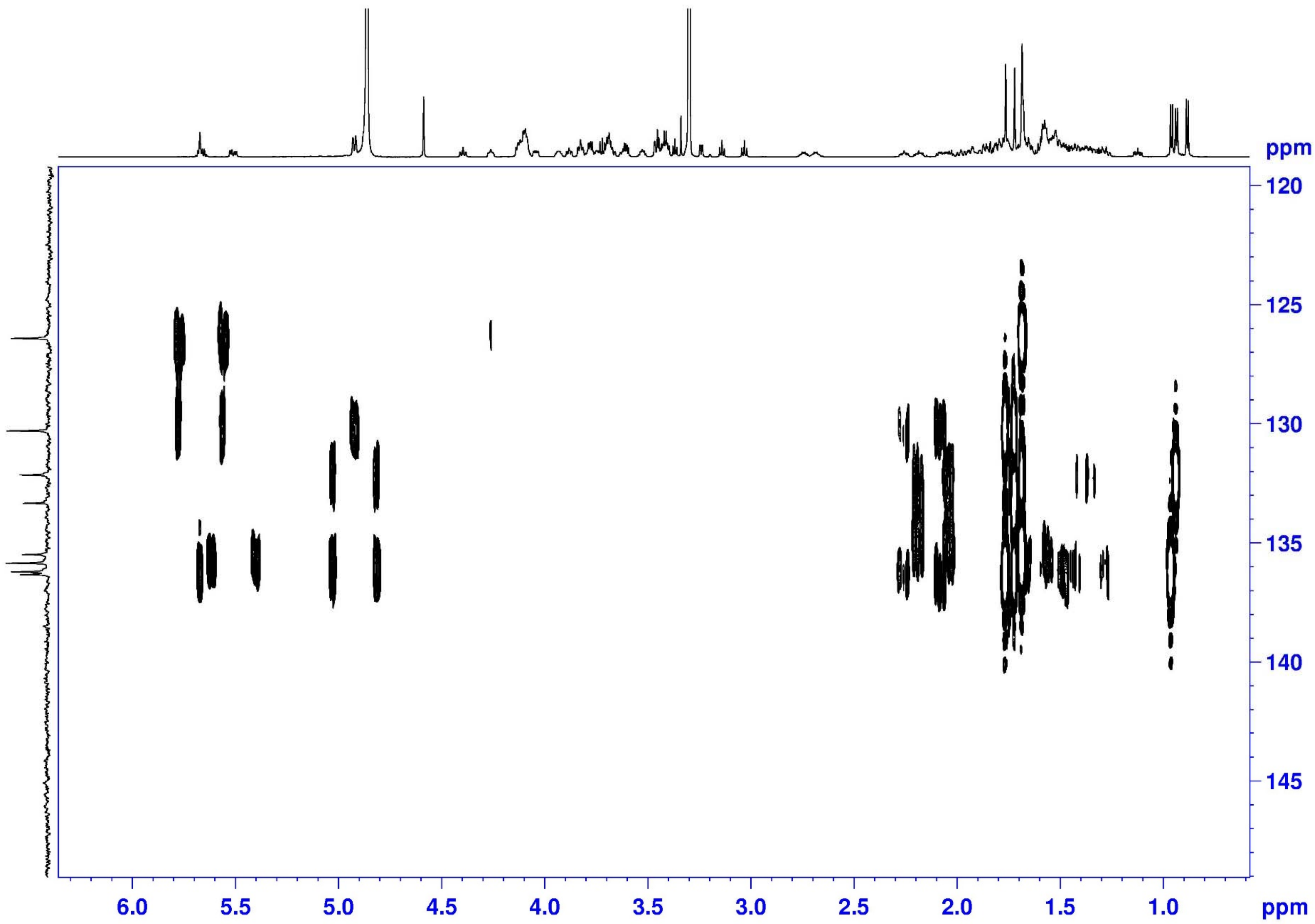

HMBC (700 MHz) spectrum of compound **1** in CD_3OD

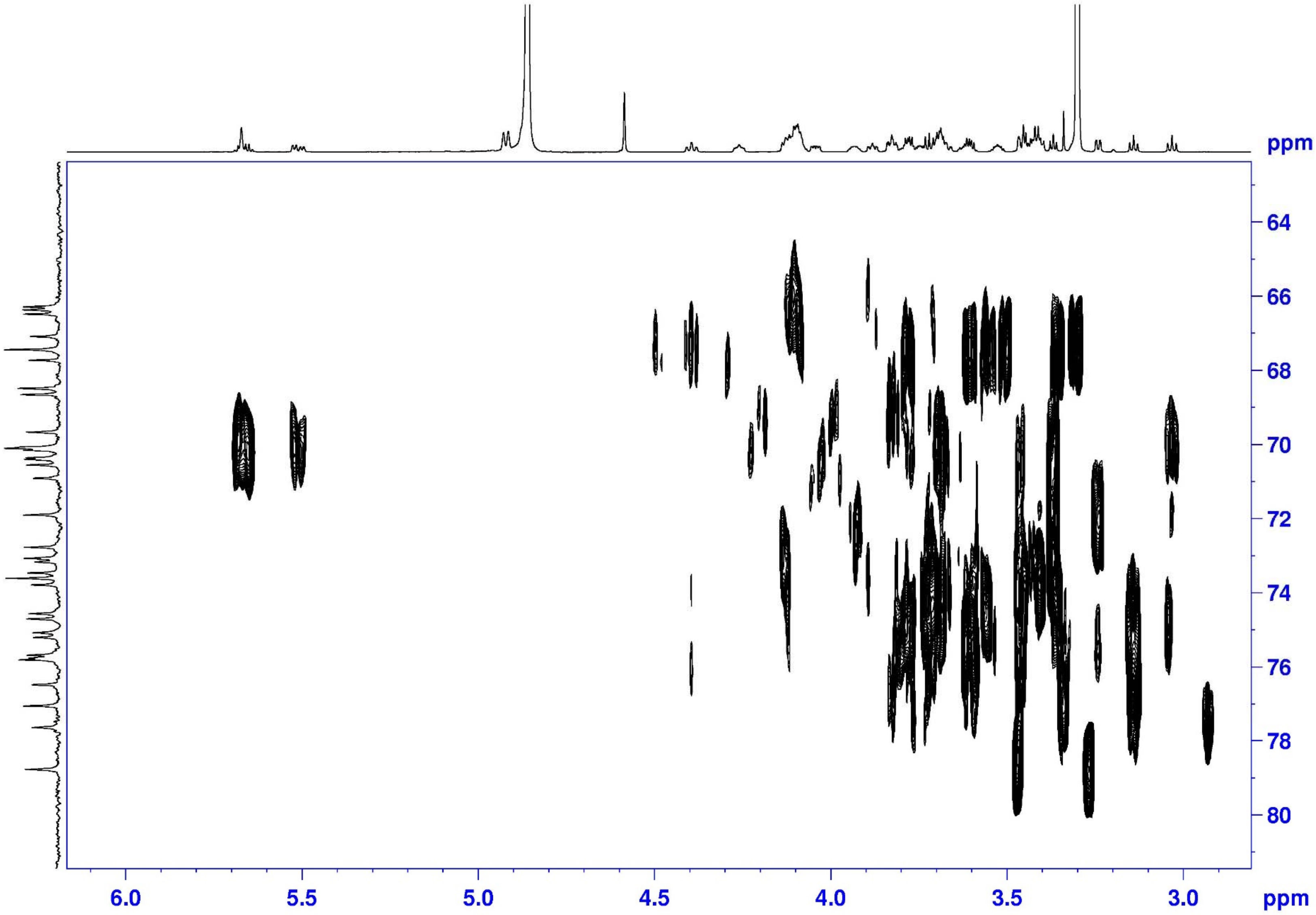

HMBC (700 MHz) spectrum of compound **1** in CD_3OD

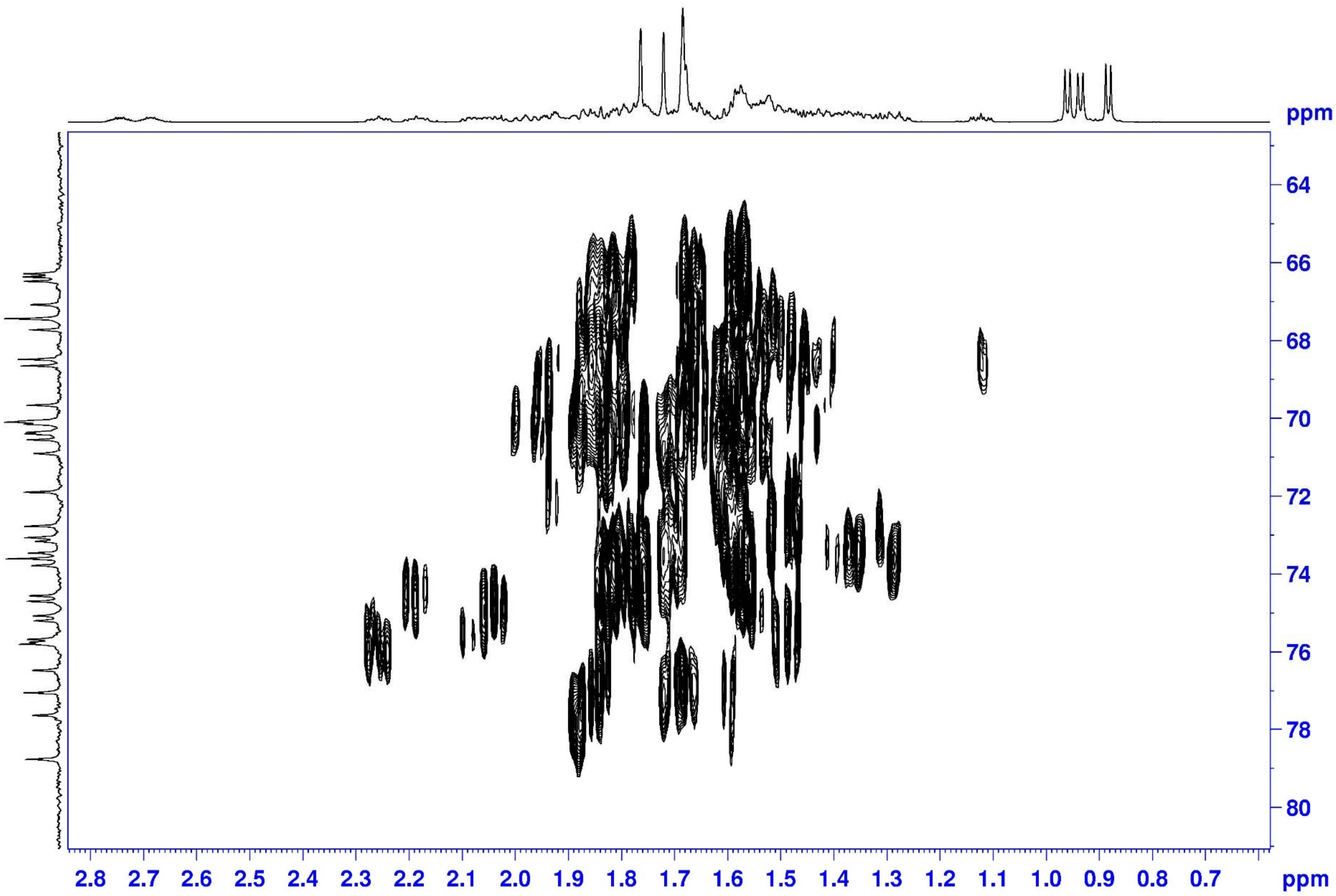

HMBC (700 MHz) spectrum of compound **1** in CD_3OD

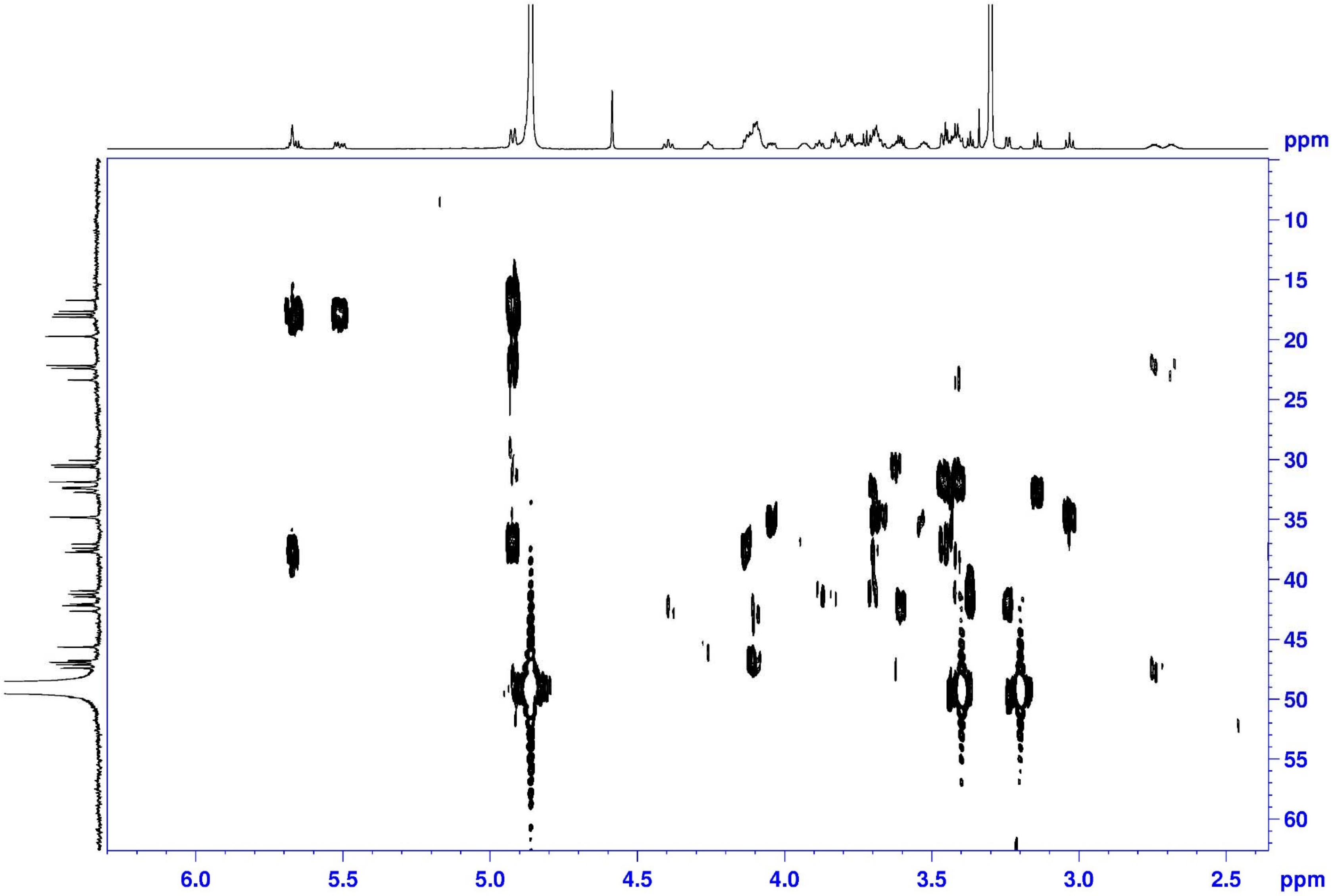

HMBC (700 MHz) spectrum of compound **1** in CD_3OD

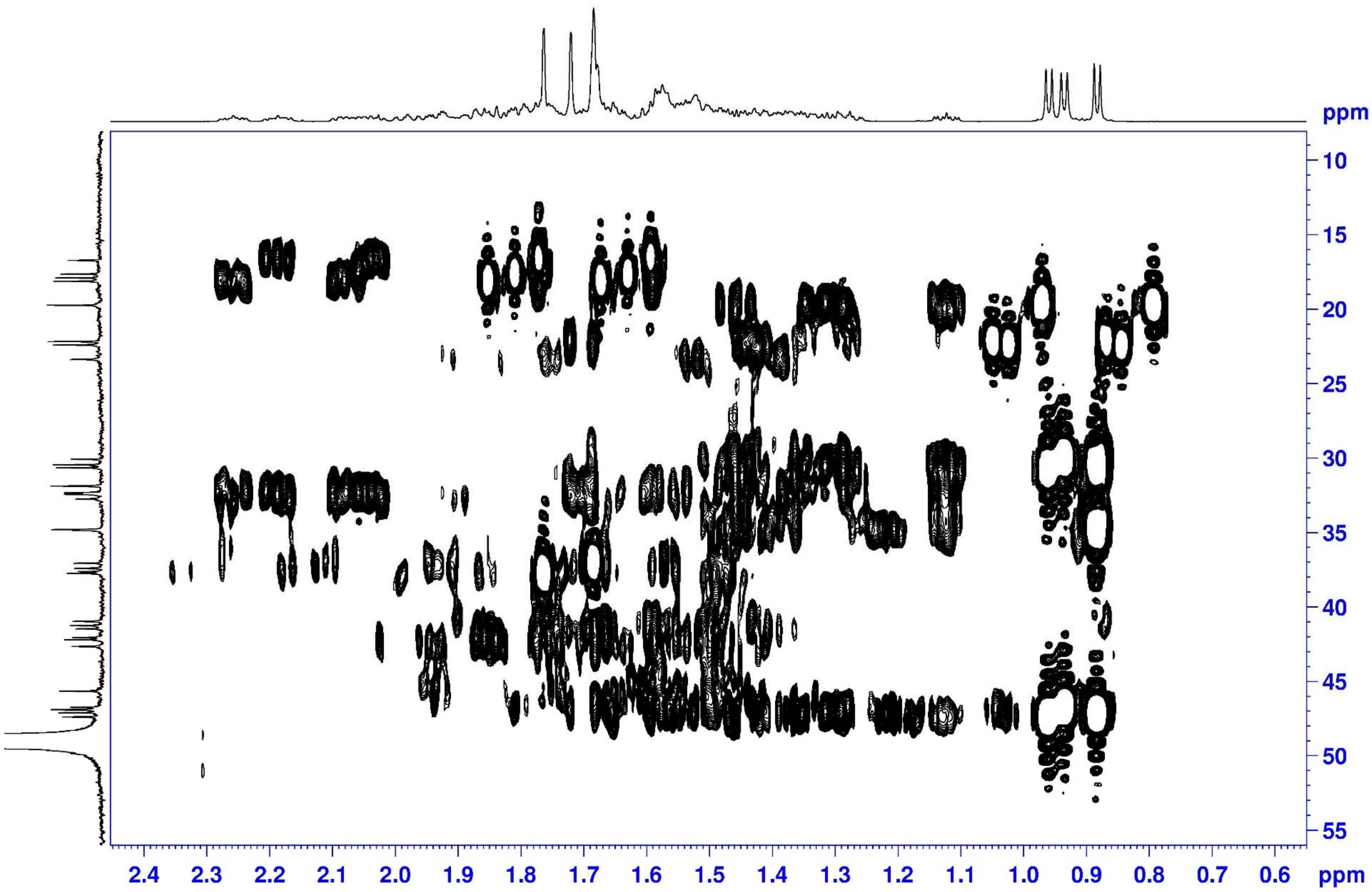

H2BC (700 MHz) spectrum of compound **1** in CD_3OD

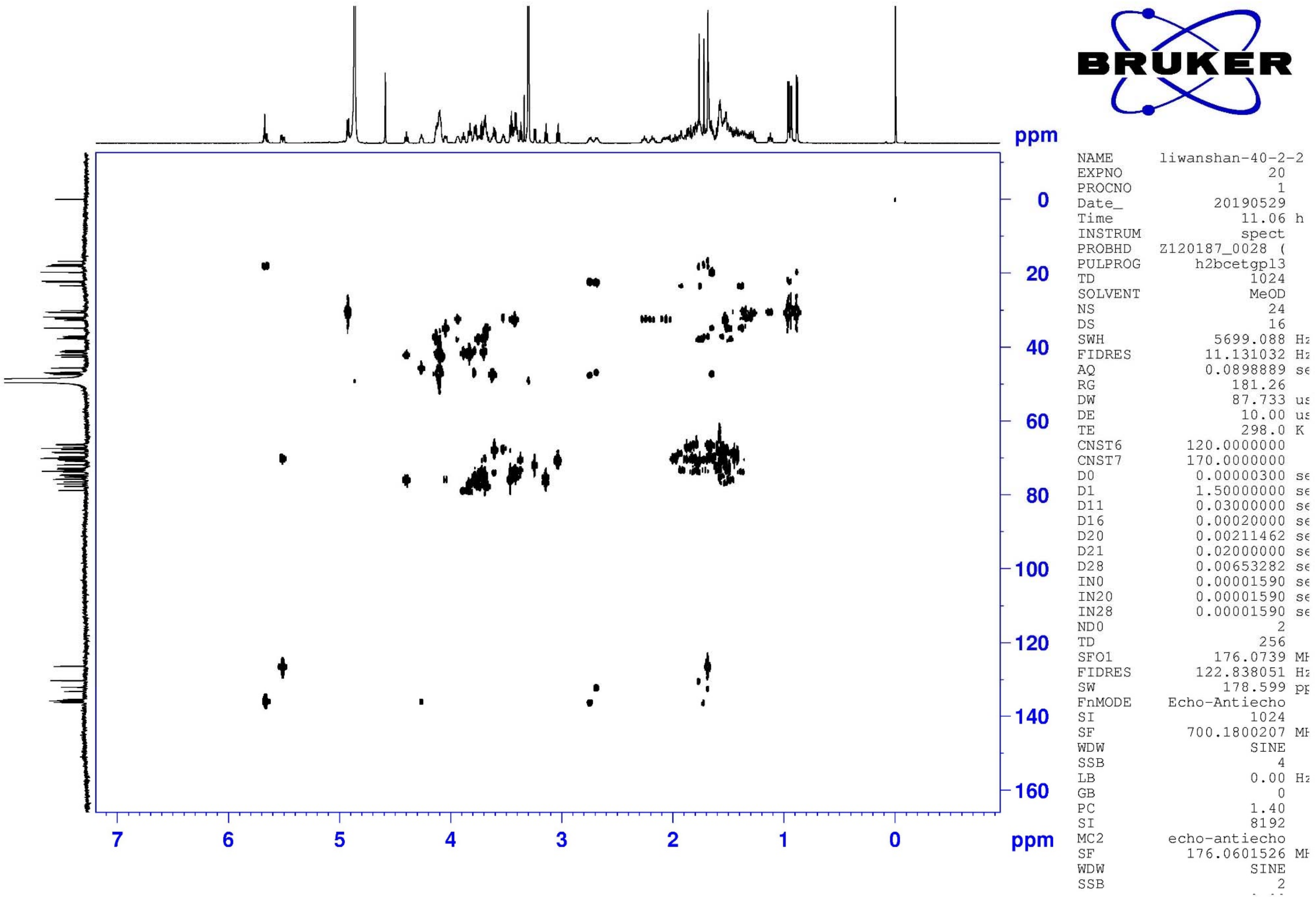

H2BC (700 MHz) spectrum of compound **1** in CD_3OD

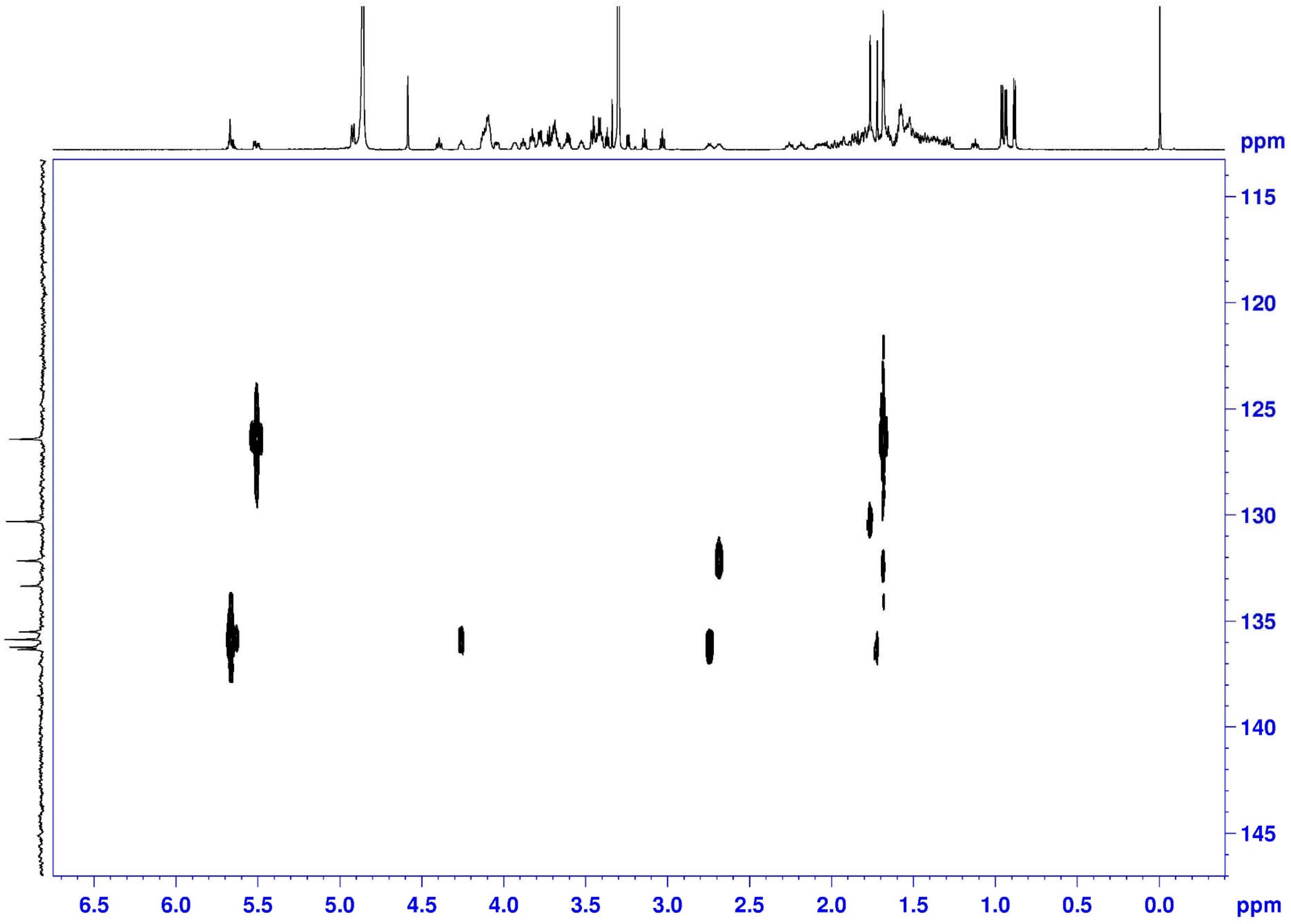

H2BC (700 MHz) spectrum of compound **1** in CD_3OD

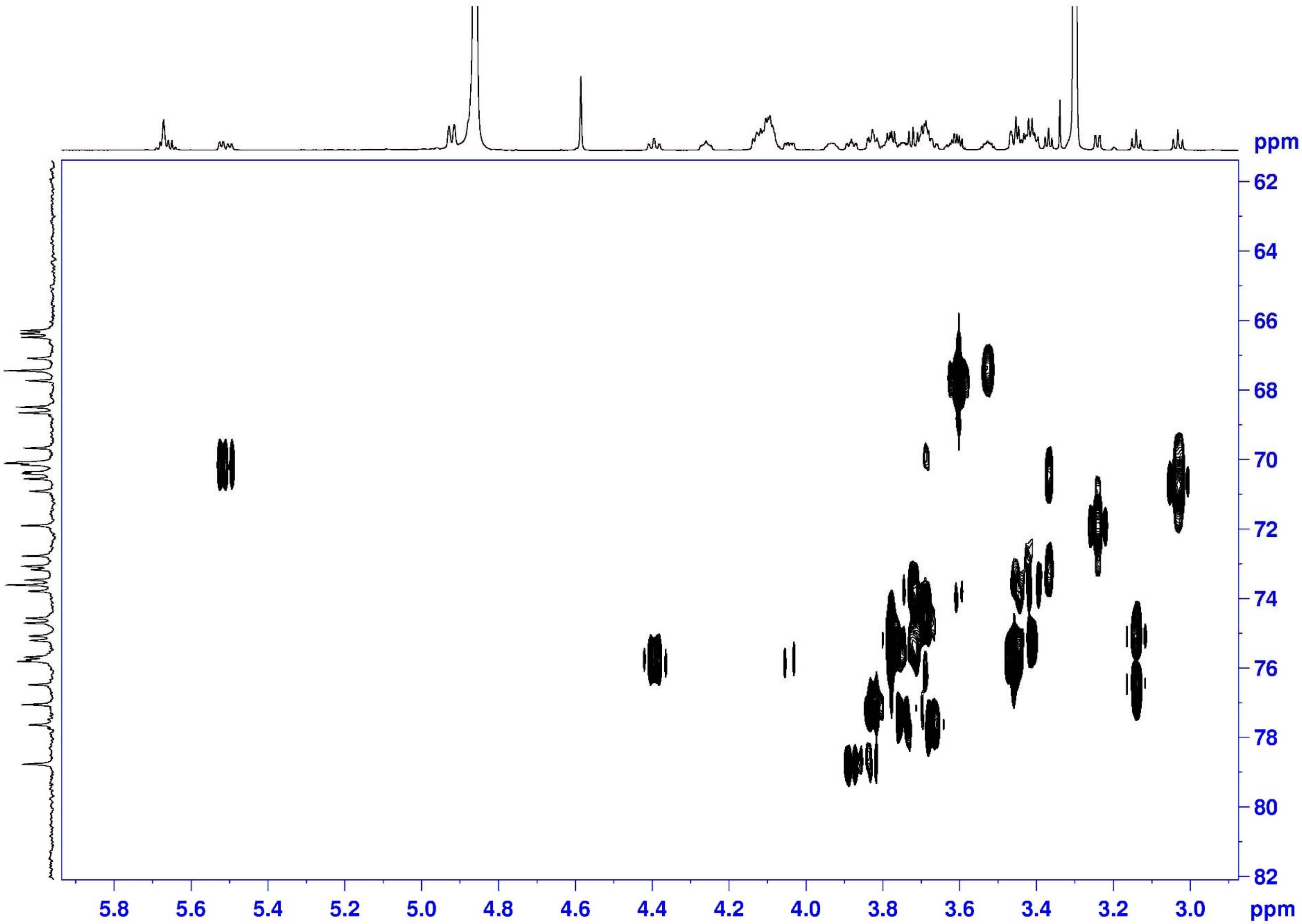

H2BC (700 MHz) spectrum of compound **1** in CD_3OD

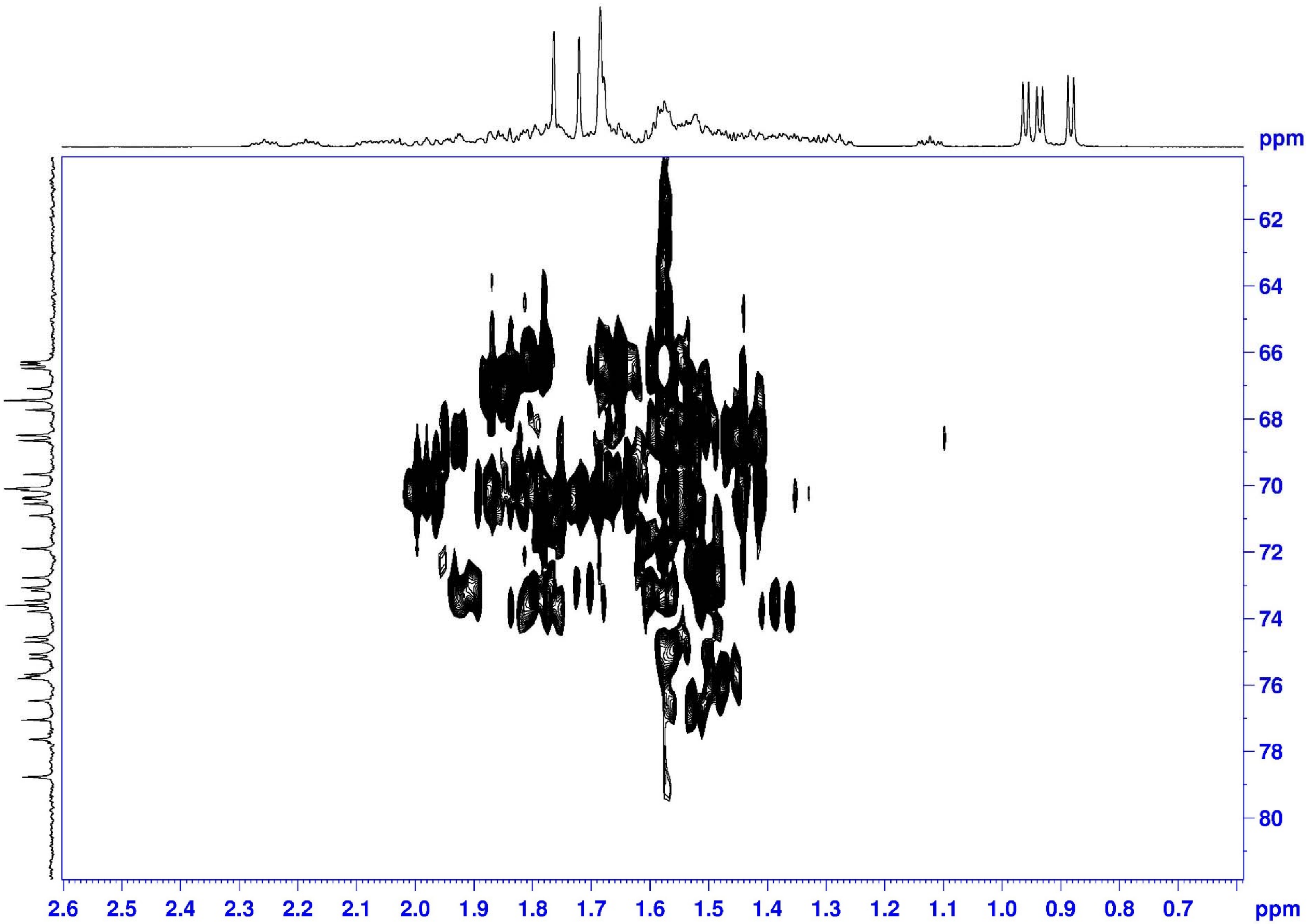

H2BC (700 MHz) spectrum of compound **1** in CD_3OD

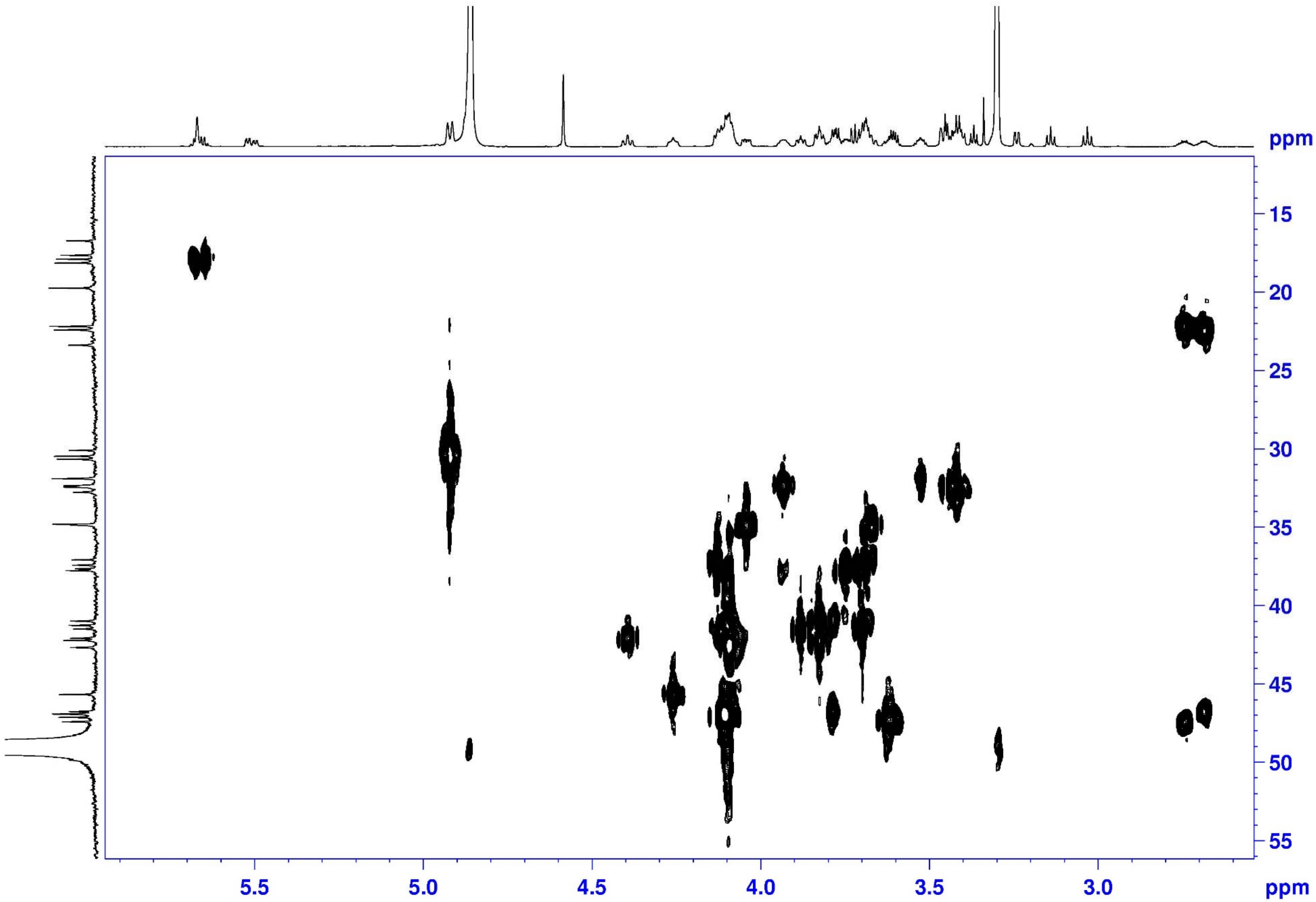

H2BC (700 MHz) spectrum of compound **1** in CD_3OD

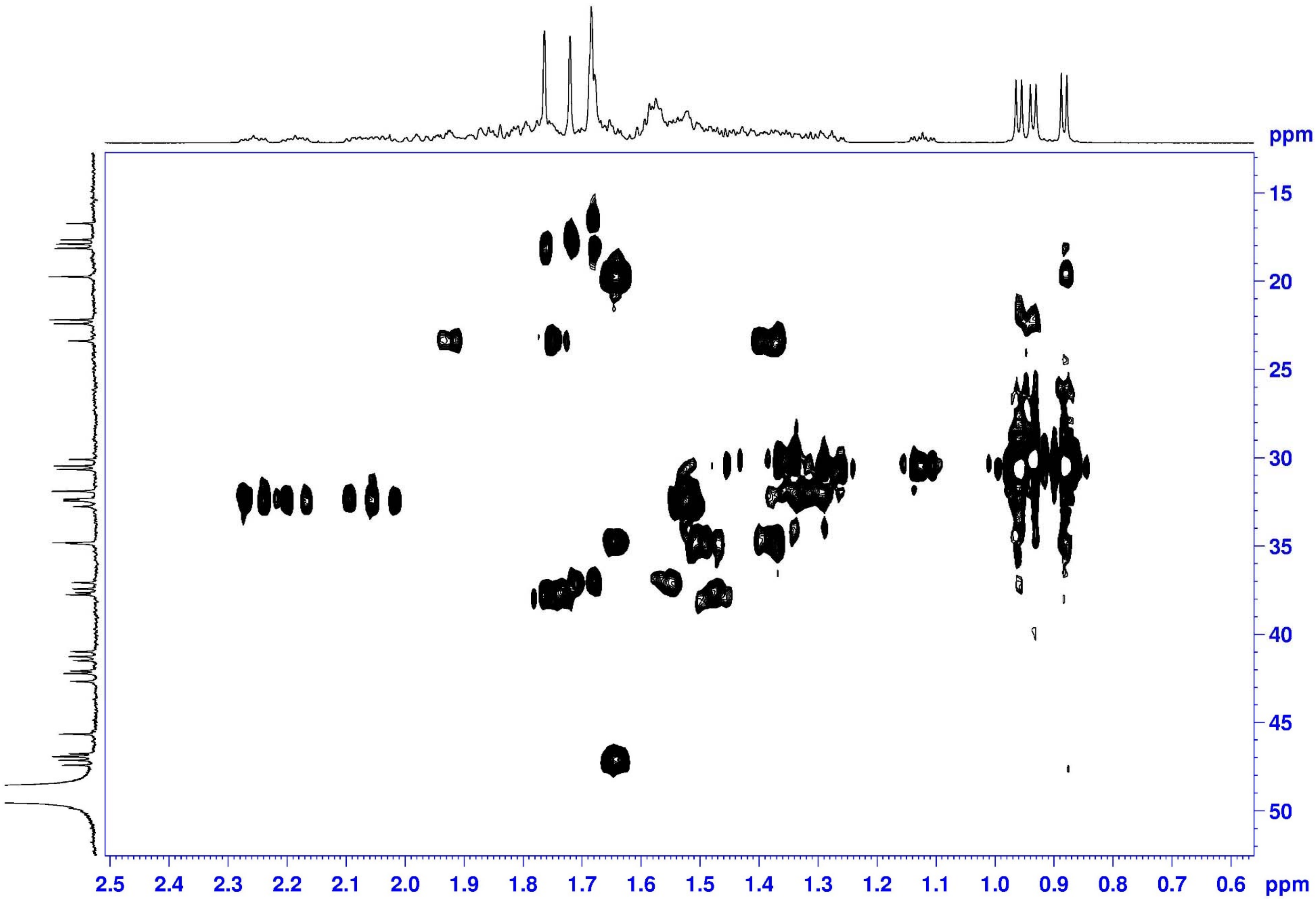

NOESY (700 MHz) spectrum of compound **1** in CD_3OD

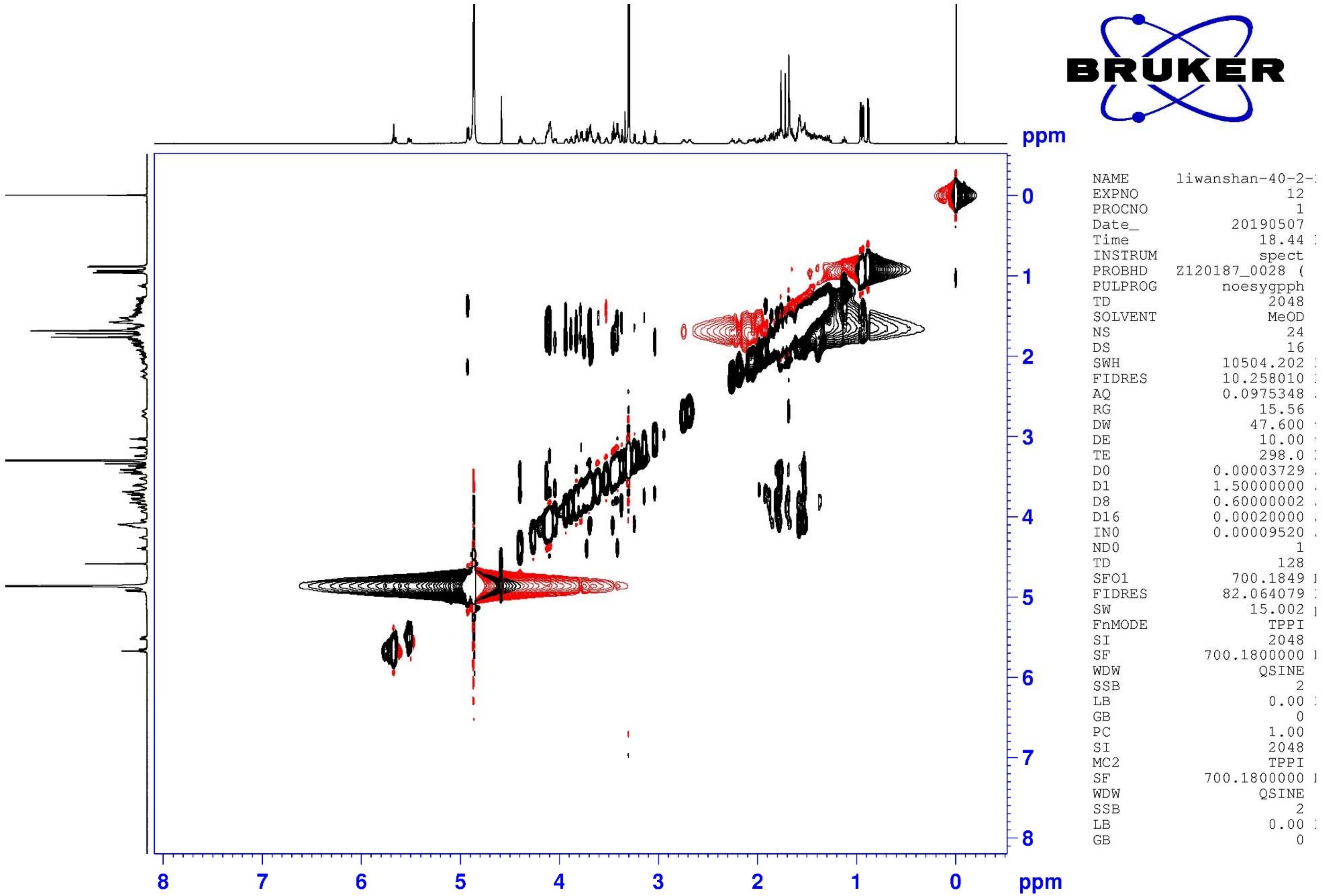

NOESY (700 MHz) spectrum of compound **1** in CD_3OD

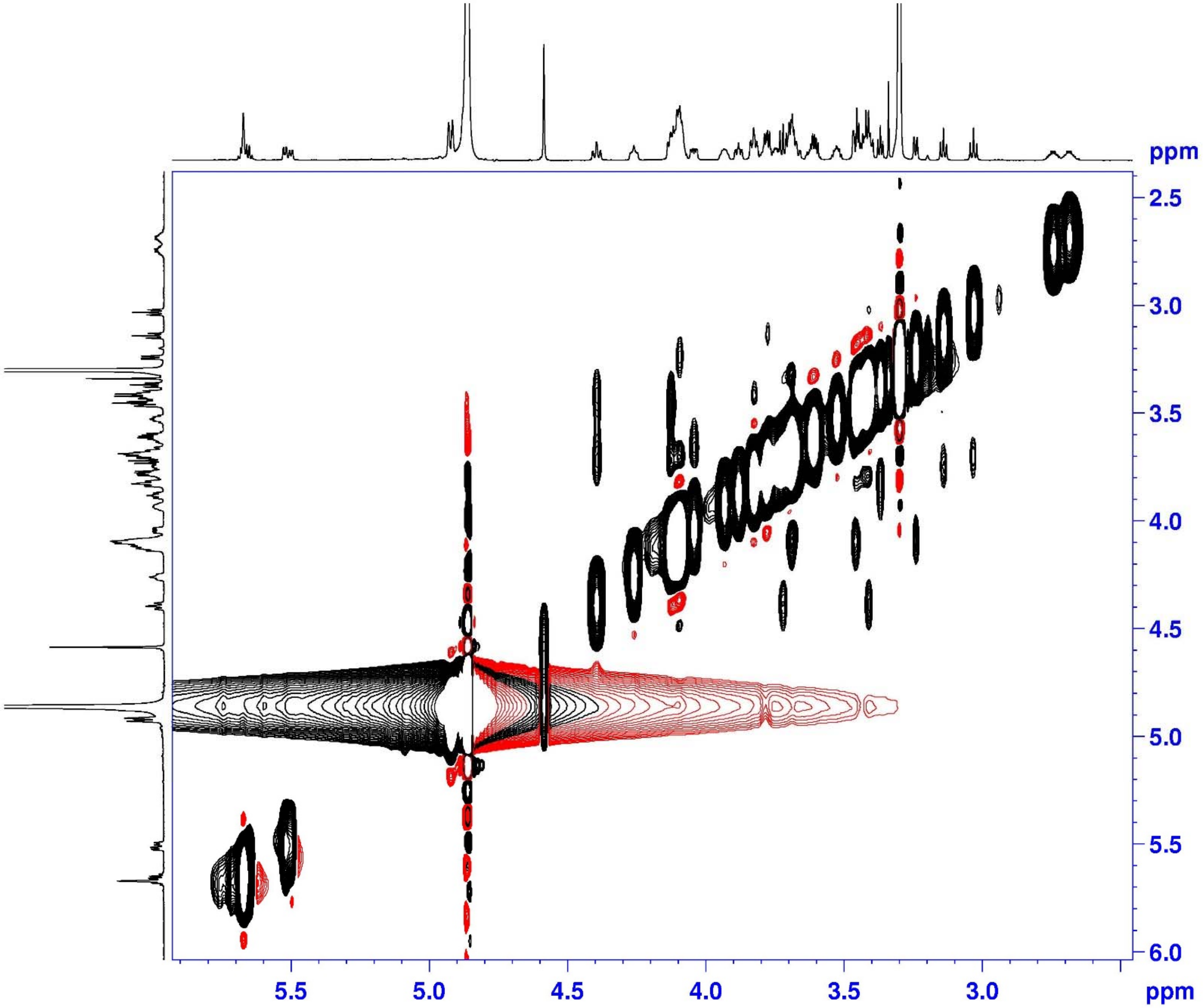

NOESY (700 MHz) spectrum of compound **1** in CD_3OD

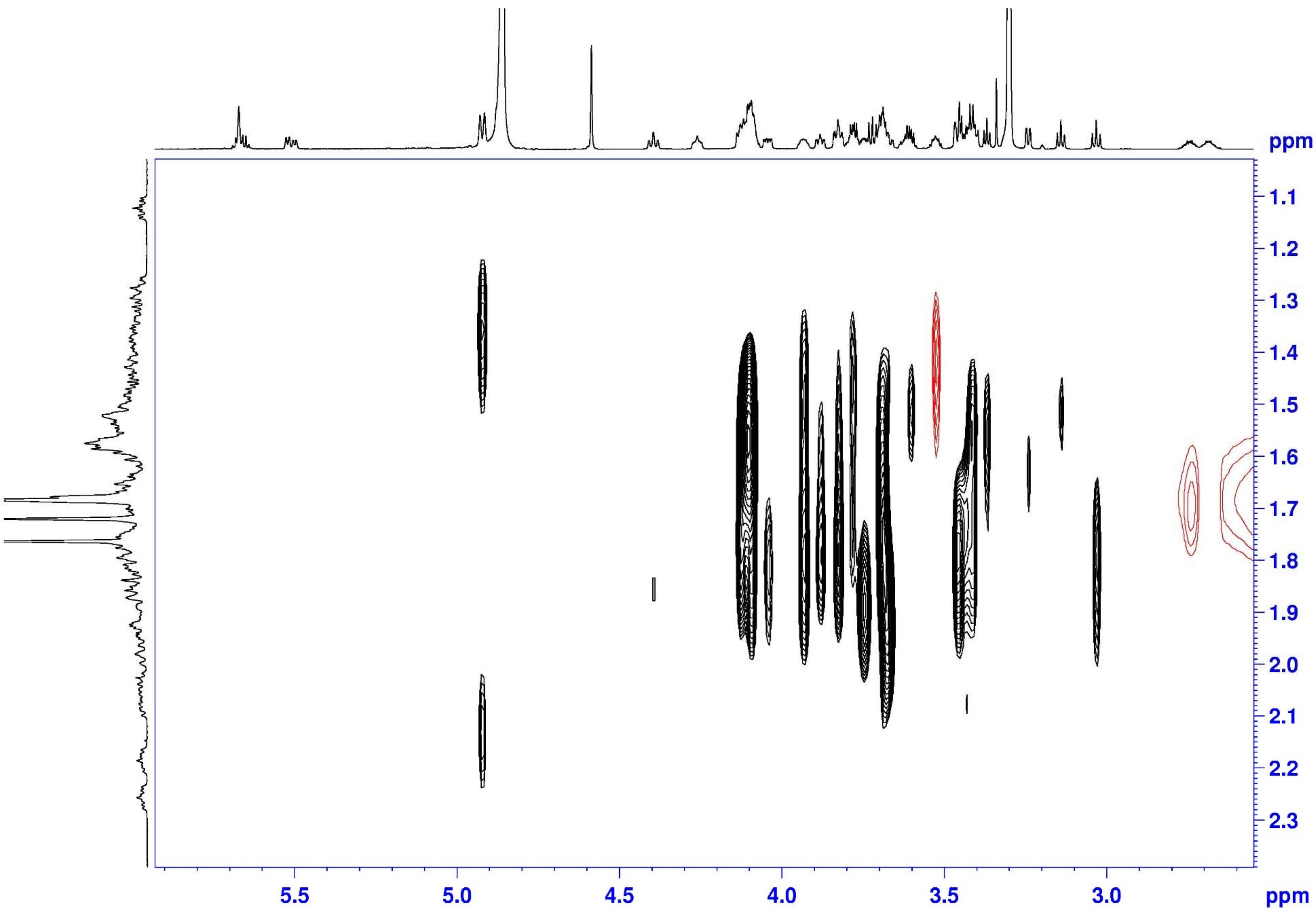

^{13}C (175 MHz) NMR spectrum of compound **1** in CD_3OH

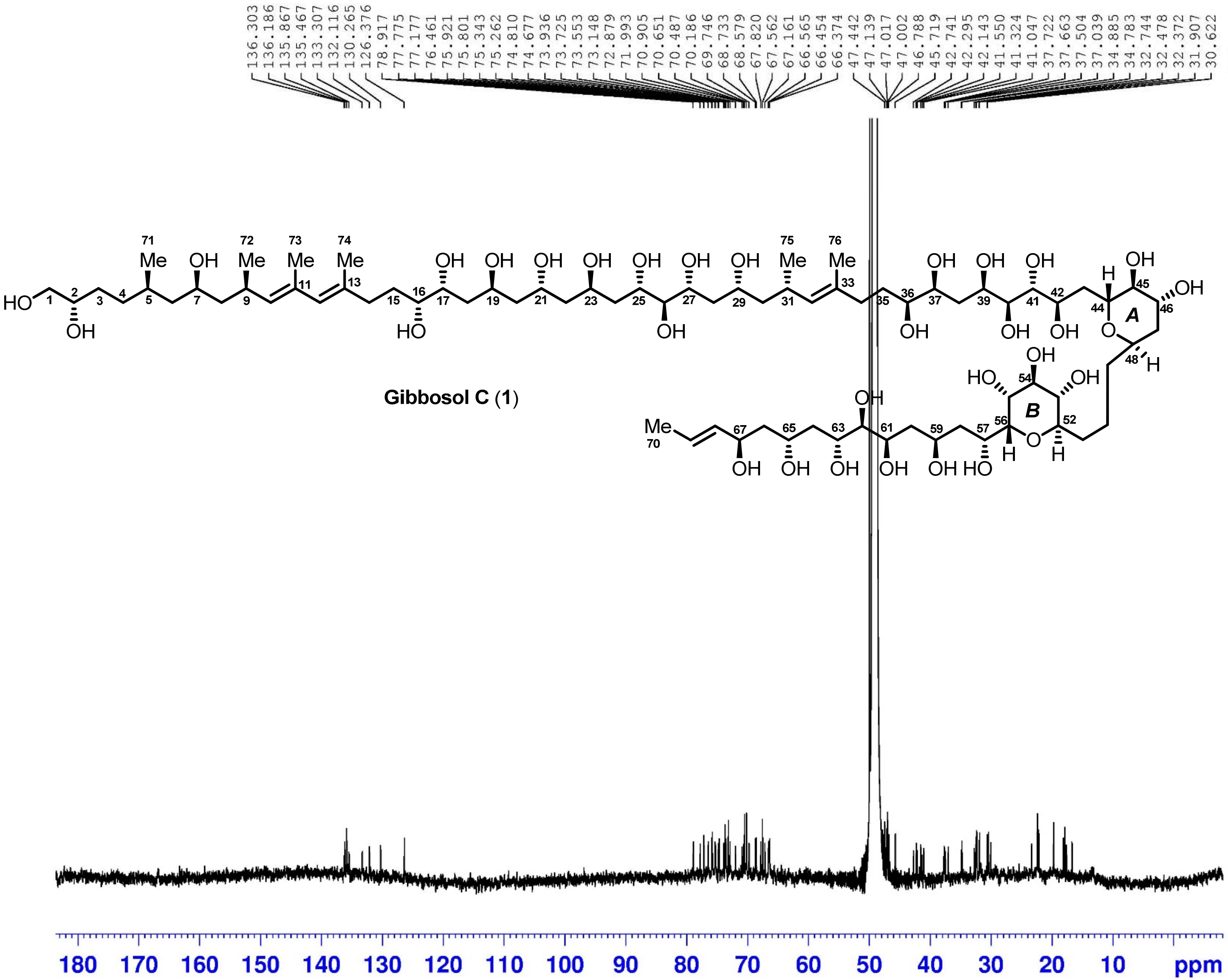

NAME	liwanshan-40-2-2-MEO	
EXPNO	12	
PROCNO	1	
Date_	20200816	
Time	2.44	h
INSTRUM	spect	
PROBHD	Z120187_0028 (	
PULPROG	zgpg30	
TD	32768	
SOLVENT	MeOD	
NS	15000	
DS	8	
SWH	43859.648	Hz
FIDRES	2.676980	Hz
AQ	0.3736052	sec
RG	181.26	
DW	11.400	usec
DE	18.00	usec
TE	298.0	K
D1	1.00000000	sec
D11	0.03000000	sec
TD0	1	
SFO1	176.0797677	MHz
NUC1	13C	
P1	11.90	usec
SI	32768	
SF	176.0604010	MHz
WDW	EM	
SSB	0	
LB	4.00	Hz
GB	0	
PC	1.40	

Comparison of ^{13}C (175 MHz) NMR spectrum of compound **1** in CD_3OH with that in CD_3OD

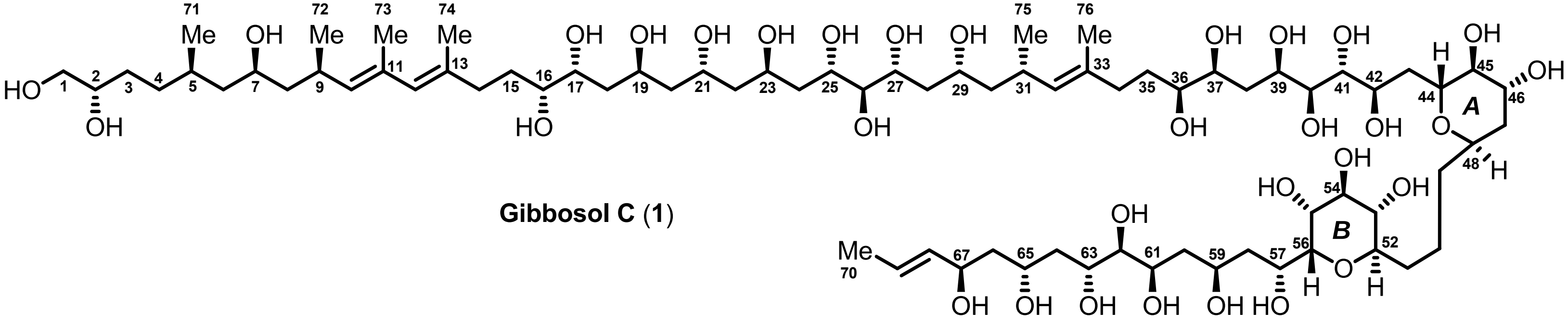

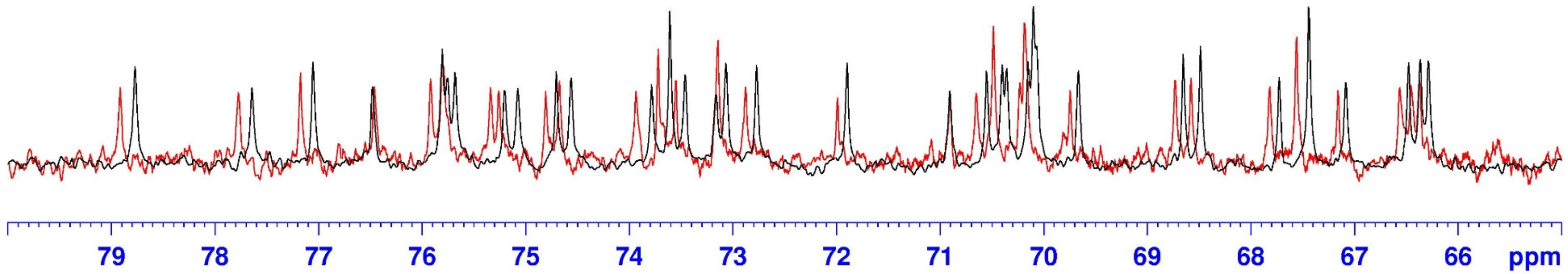

Comparison of ^{13}C (175 MHz) NMR spectrum of compound **1** in CD_3OH with that in CD_3OD

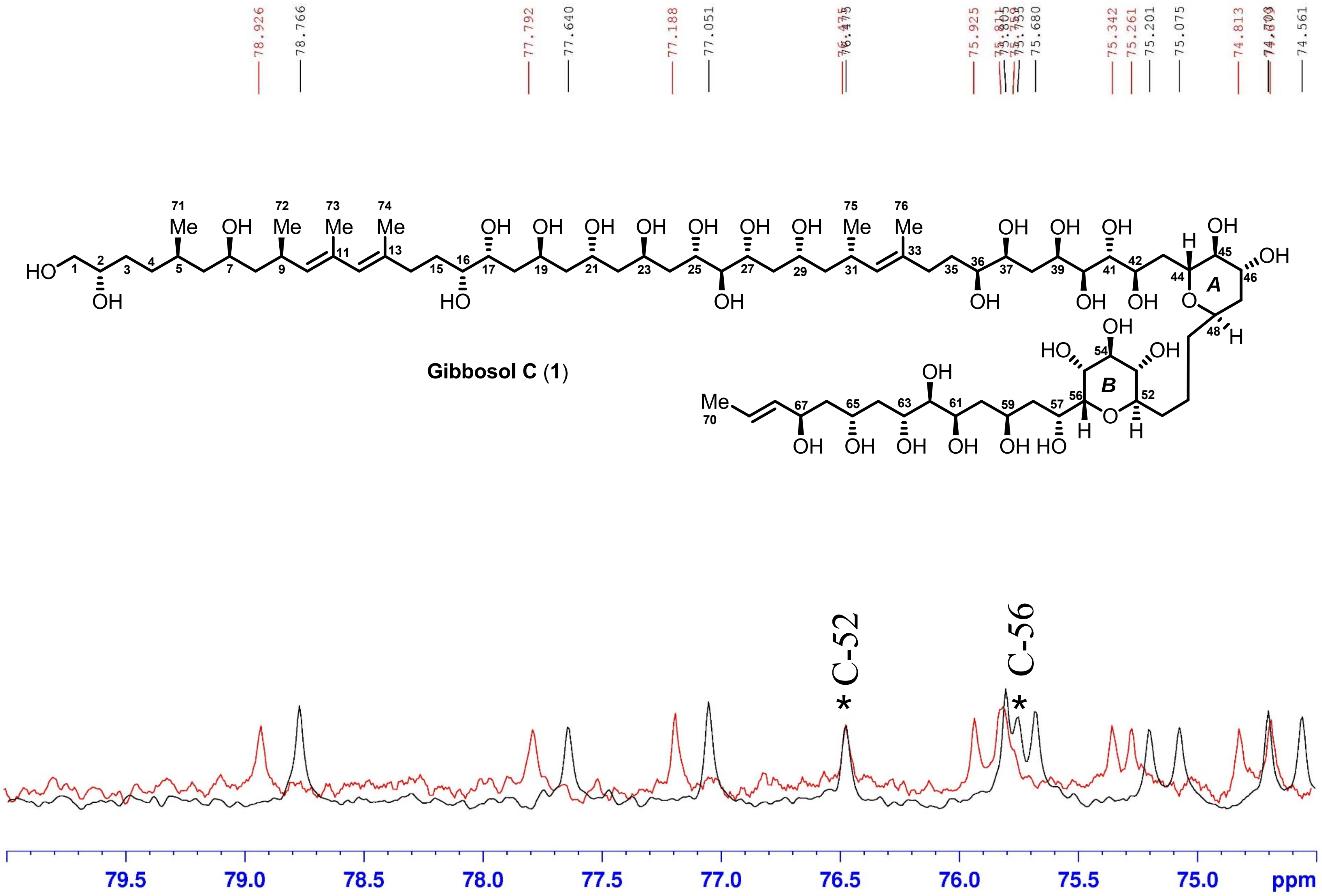

Comparison of ^{13}C (175 MHz) NMR spectrum of compound **1** in CD_3OH with that in CD_3OD

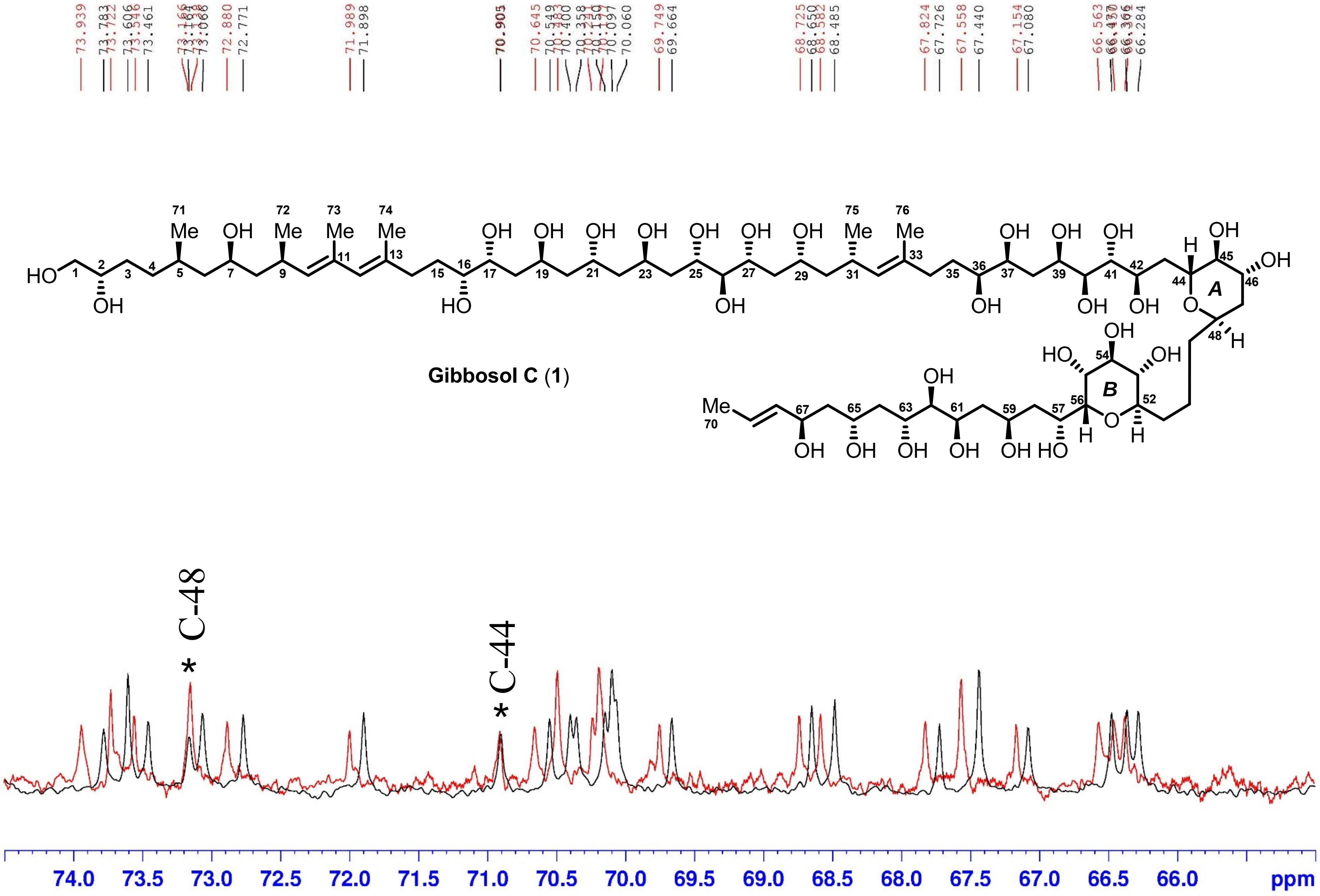

LR-ESI-MS for the fragment **1a**

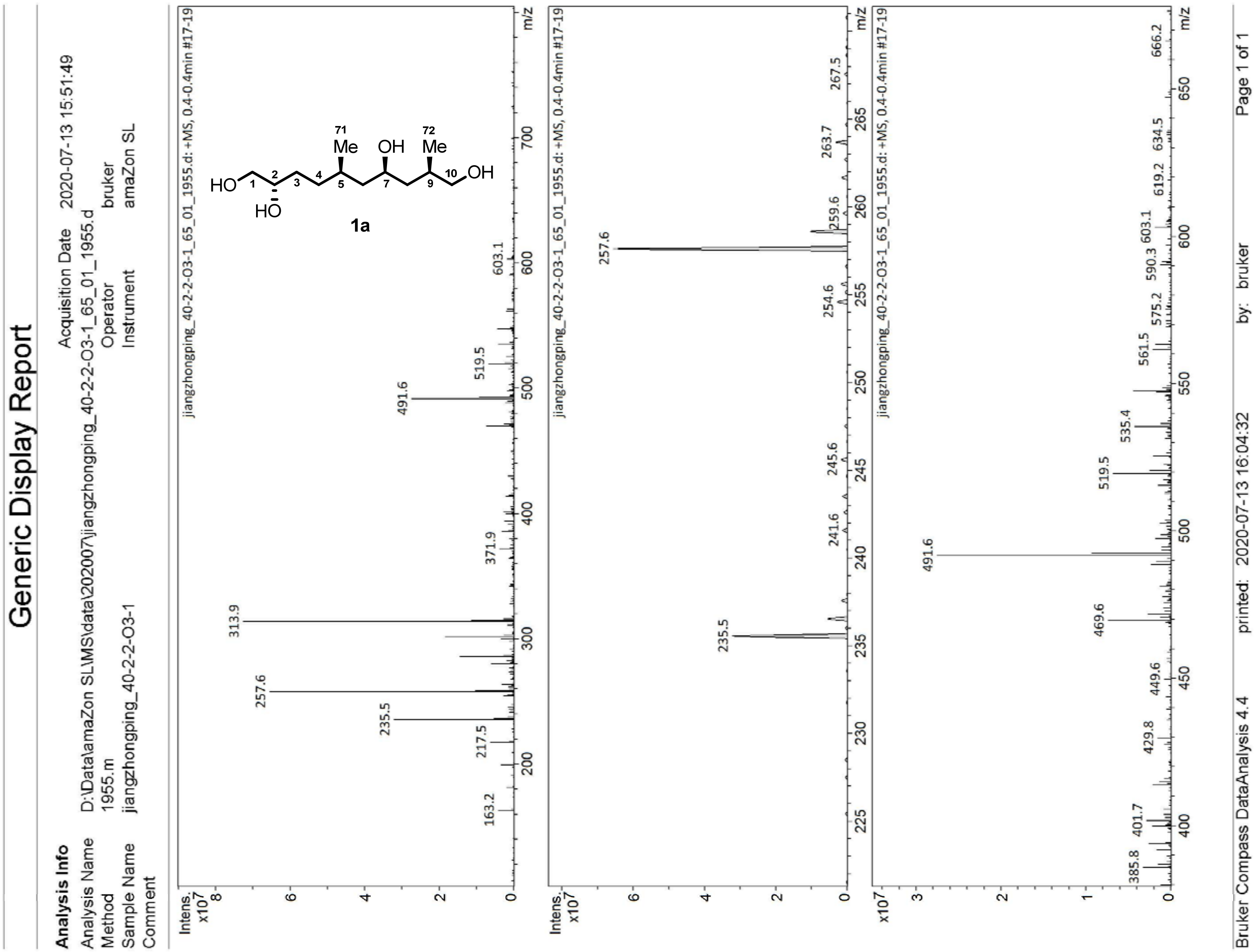

^{1}H (700 MHz) NMR spectrum of the fragment **1a** in CD_3OD

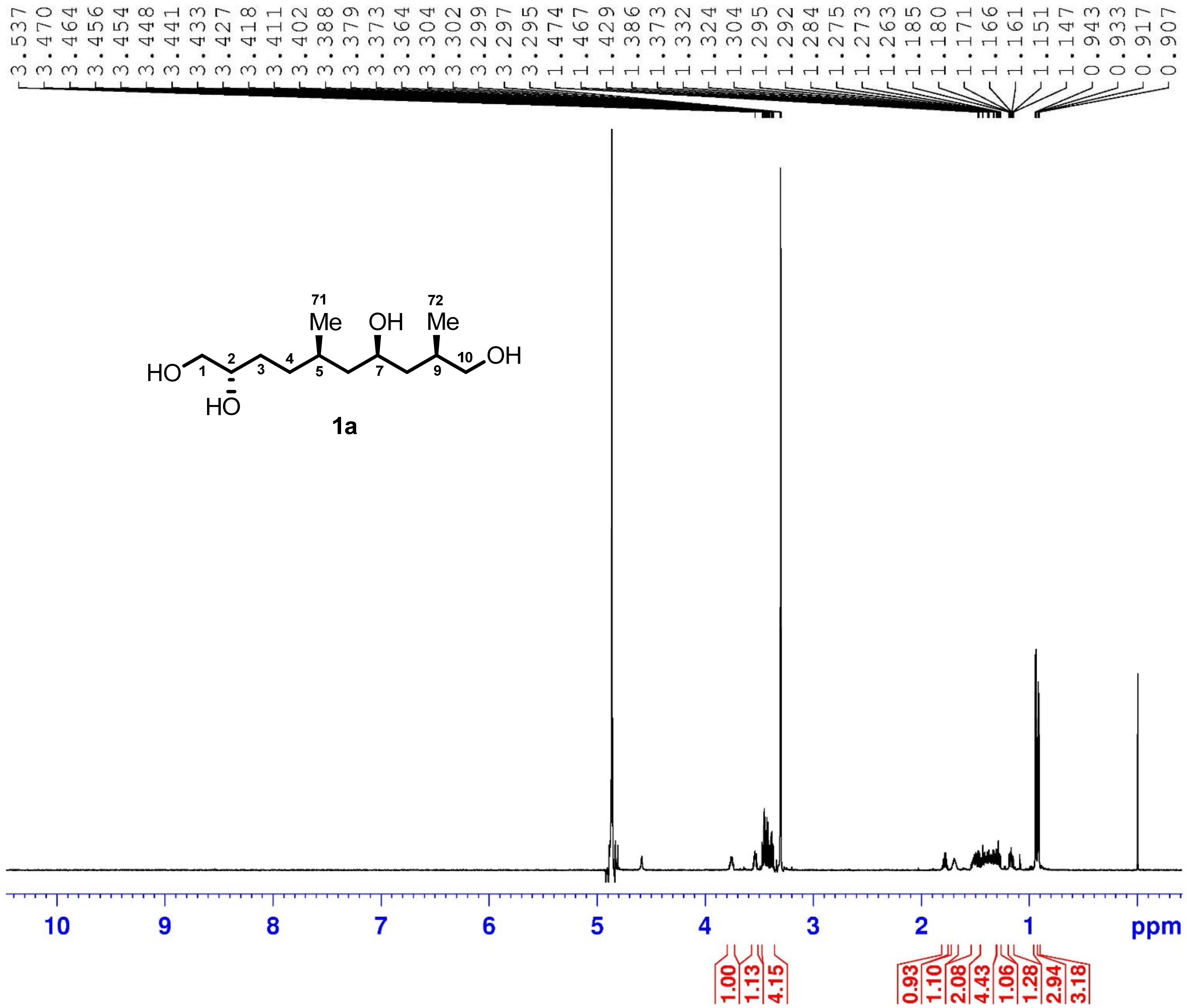

1a

NAME	liwanshan-40-2-2-03-
EXPNO	12
PROCNO	1
Date_	20200306
Time	18.28 h
INSTRUM	spect
PROBHD	Z120187_0028 (
PULPROG	zg30
TD	65536
SOLVENT	MeOD
NS	3
DS	2
SWH	14097.744 Hz
FIDRES	0.430229 Hz
AQ	2.3243935 sec
RG	3.96
DW	35.467 usec
DE	10.00 usec
TE	298.0 K
D1	1.50000000 sec
TD0	1
SFO1	700.1843236 MHz
NUC1	1H
P1	8.10 usec
SI	65536
SF	700.1800209 MHz
WDW	EM
SSB	0
LB	0.30 Hz
GB	0
PC	1.00

^{1}H (700 MHz) NMR spectrum of the fragment **1a** in CD_3OD

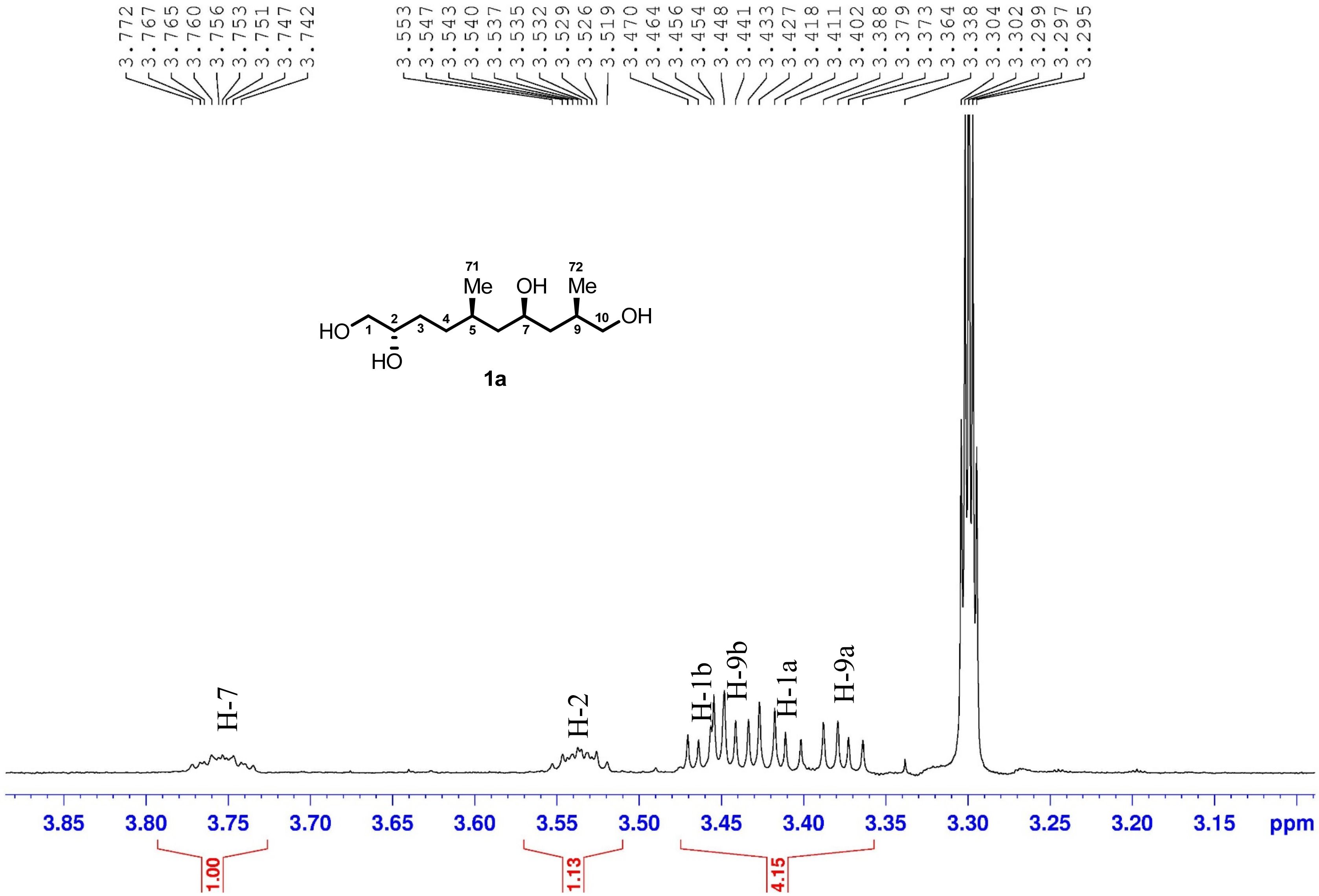

^{1}H (700 MHz) NMR spectrum of the fragment **1a** in CD_3OD

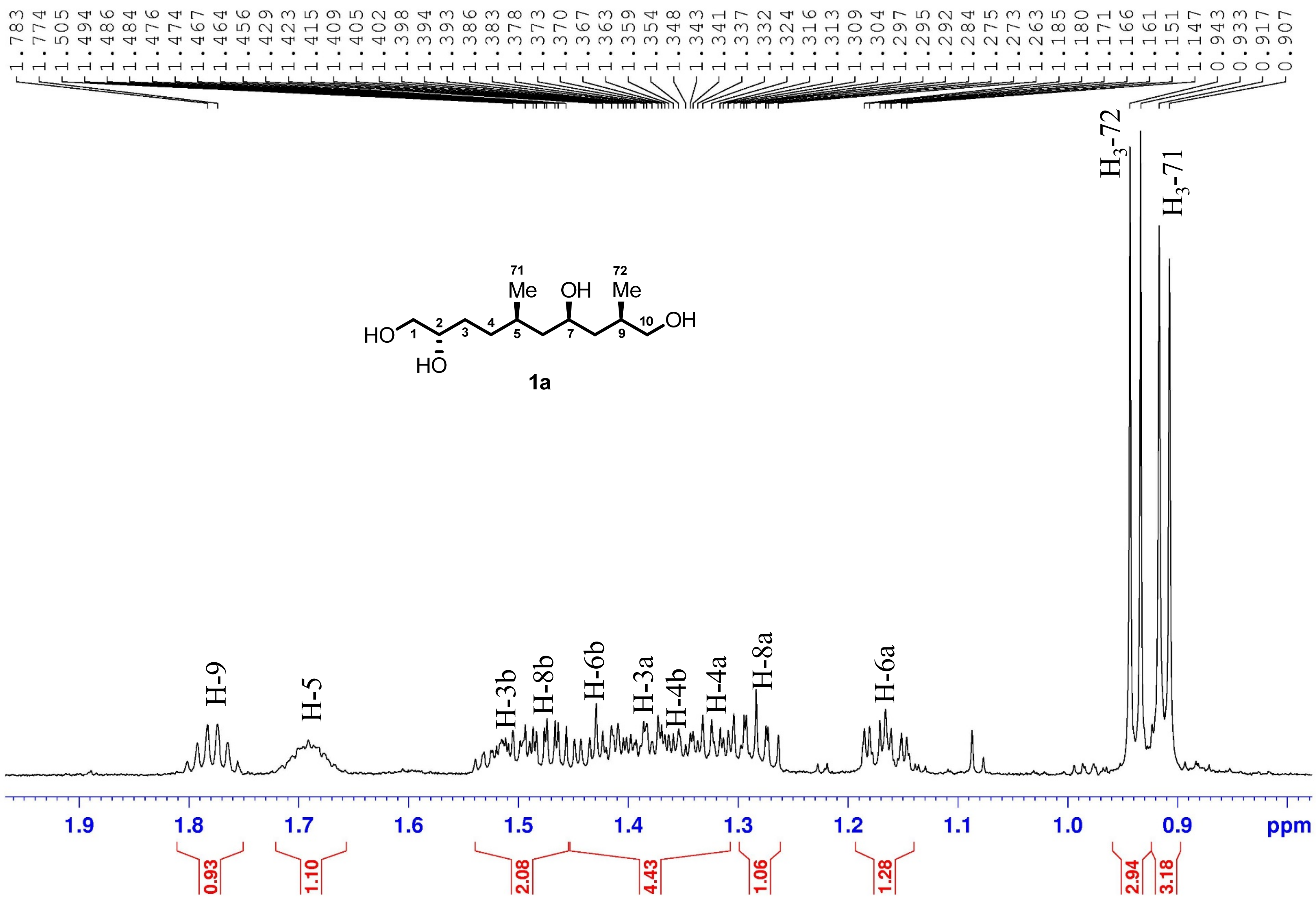

^{13}C (175 MHz) NMR spectrum of the fragment **1a** in CD_3OD

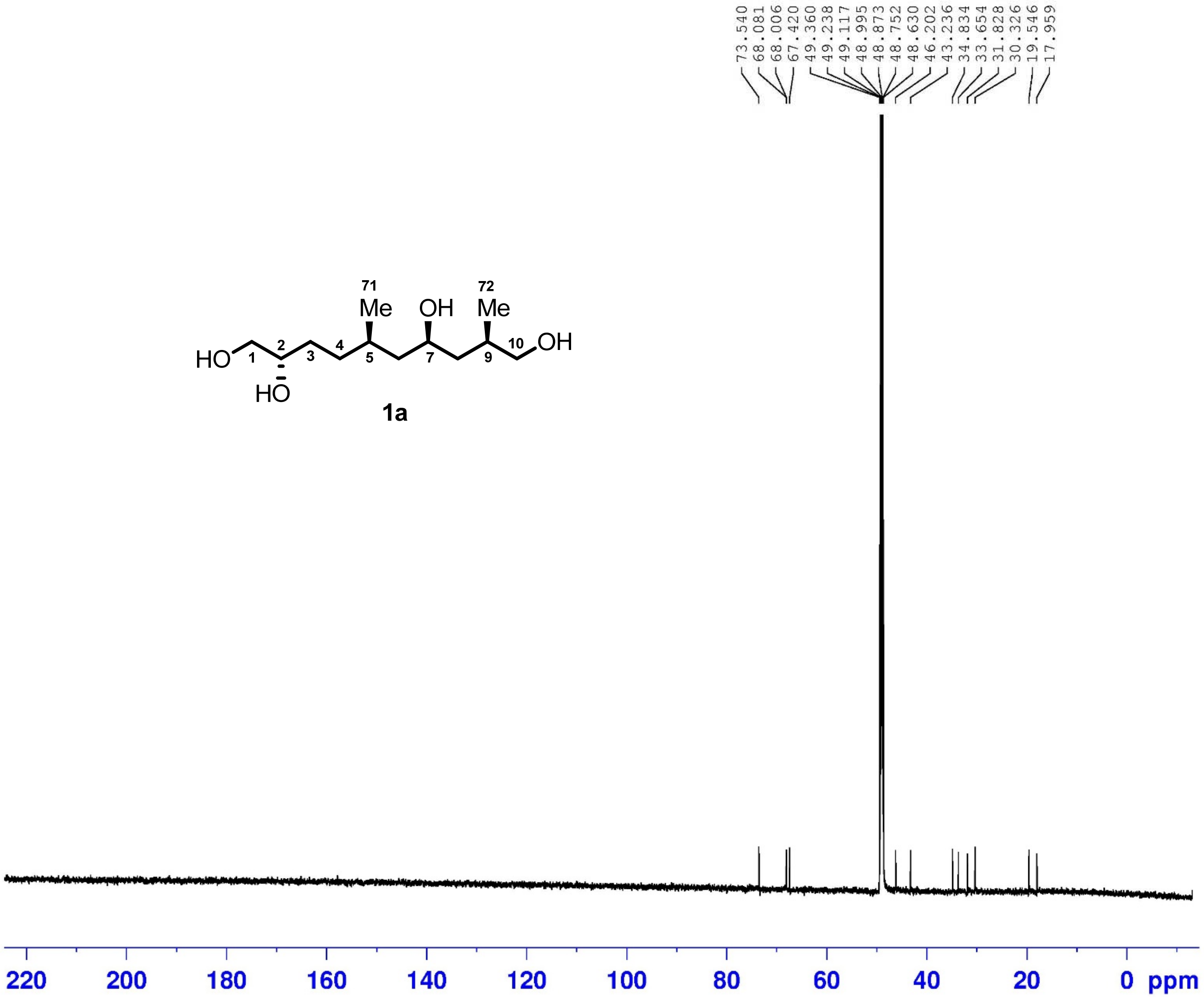

NAME	liwanshan-40-2-2-O	
EXPNO	14	
PROCNO	1	
Date_	20200306	
Time	22.40	h
INSTRUM	spect	
PROBHD	Z120187_0028 (	
PULPROG	zgpg30	
TD	32768	
SOLVENT	MeOD	
NS	8000	
DS	8	
SWH	43859.648	Hz
FIDRES	2.676980	Hz
AQ	0.3736052	sec
RG	181.26	
DW	11.400	use
DE	18.00	use
TE	298.0	K
D1	1.00000000	sec
D11	0.03000000	sec
TD0	1	
SFO1	176.0797677	MHz
NUC1	13C	
P1	11.90	use
SI	32768	
SF	176.0601541	MHz
WDW	EM	
SSB	0	
LB	3.00	Hz
GB	0	
PC	1.40	

^{13}C (175 MHz) NMR spectrum of the fragment **1a** in CD_3OD

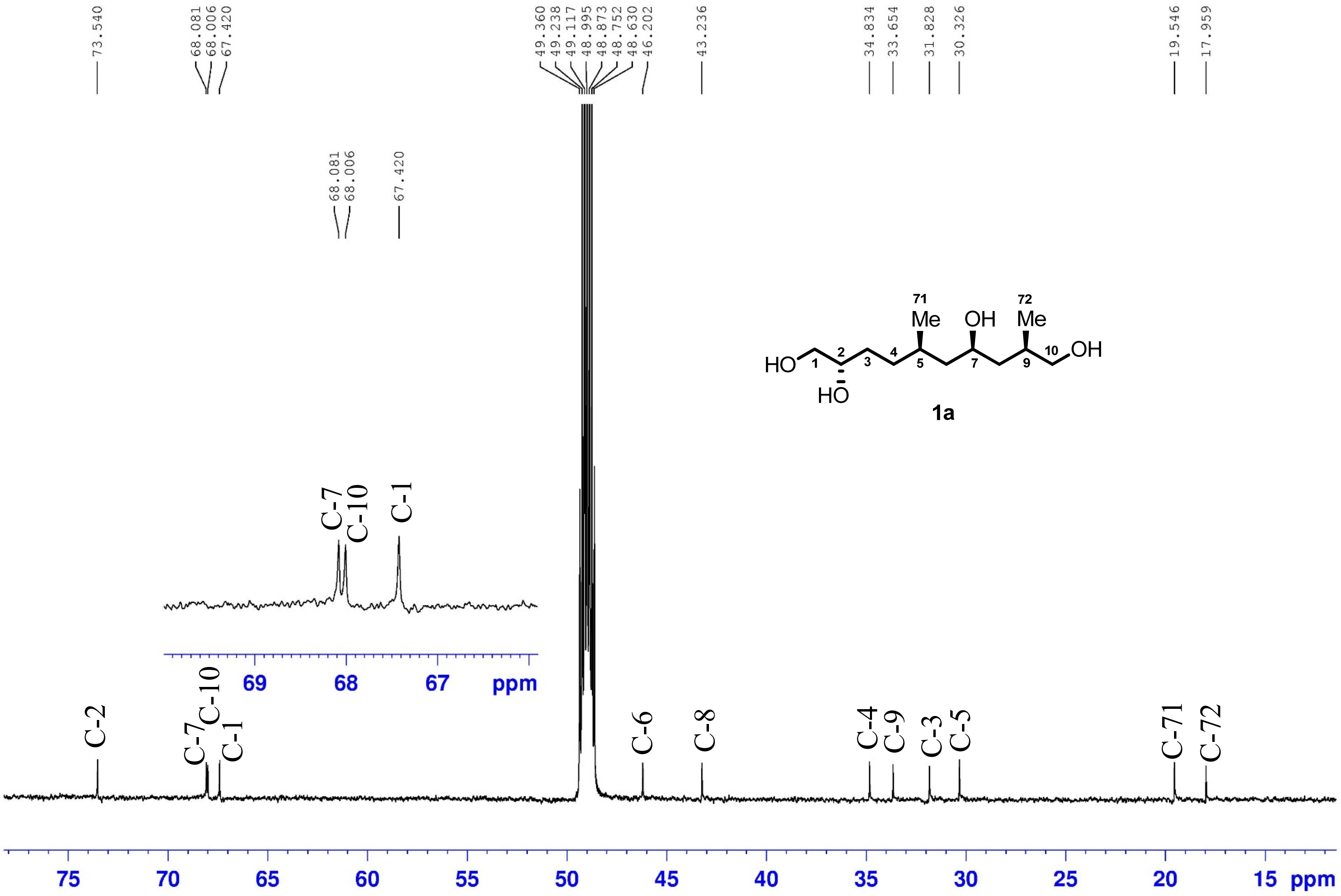

DEPT135 (175 MHz) spectrum of the fragment **1a** in CD_3OD

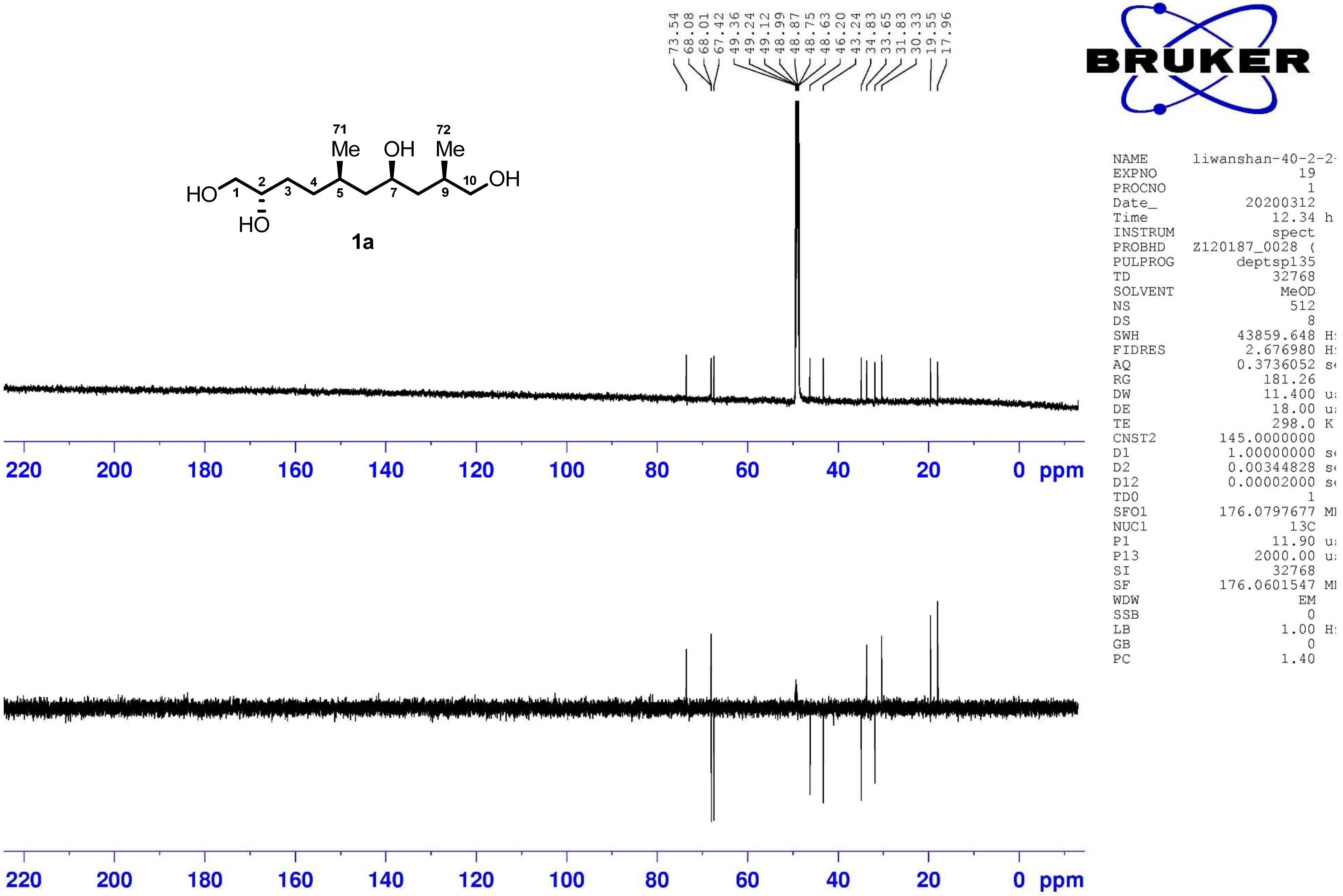

DEPT135 (175 MHz) spectrum of the fragment **1a** in CD_3OD

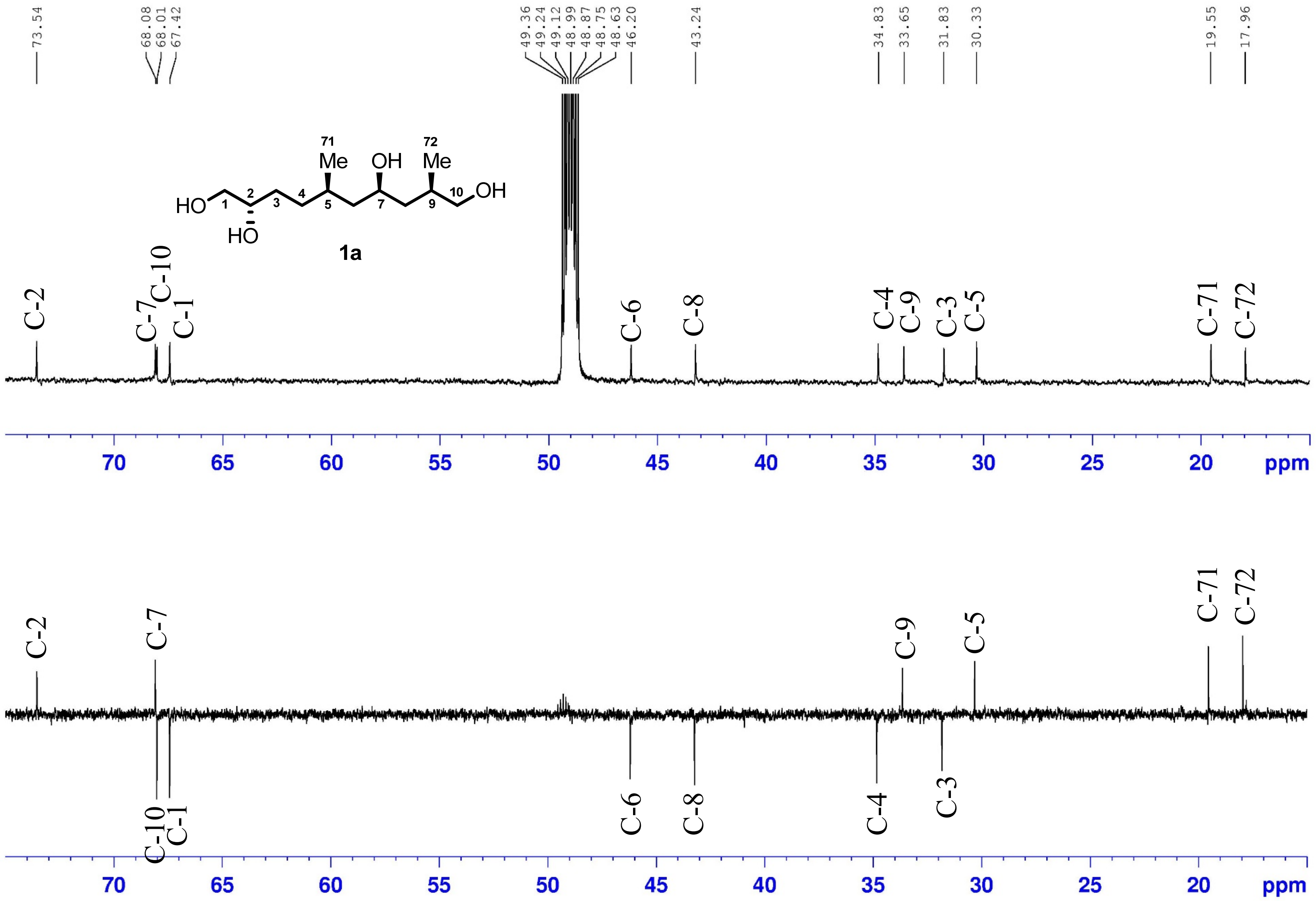

^{1}H–^{1}H COSY (700 MHz) spectrum of the fragment **1a** in CD_3OD

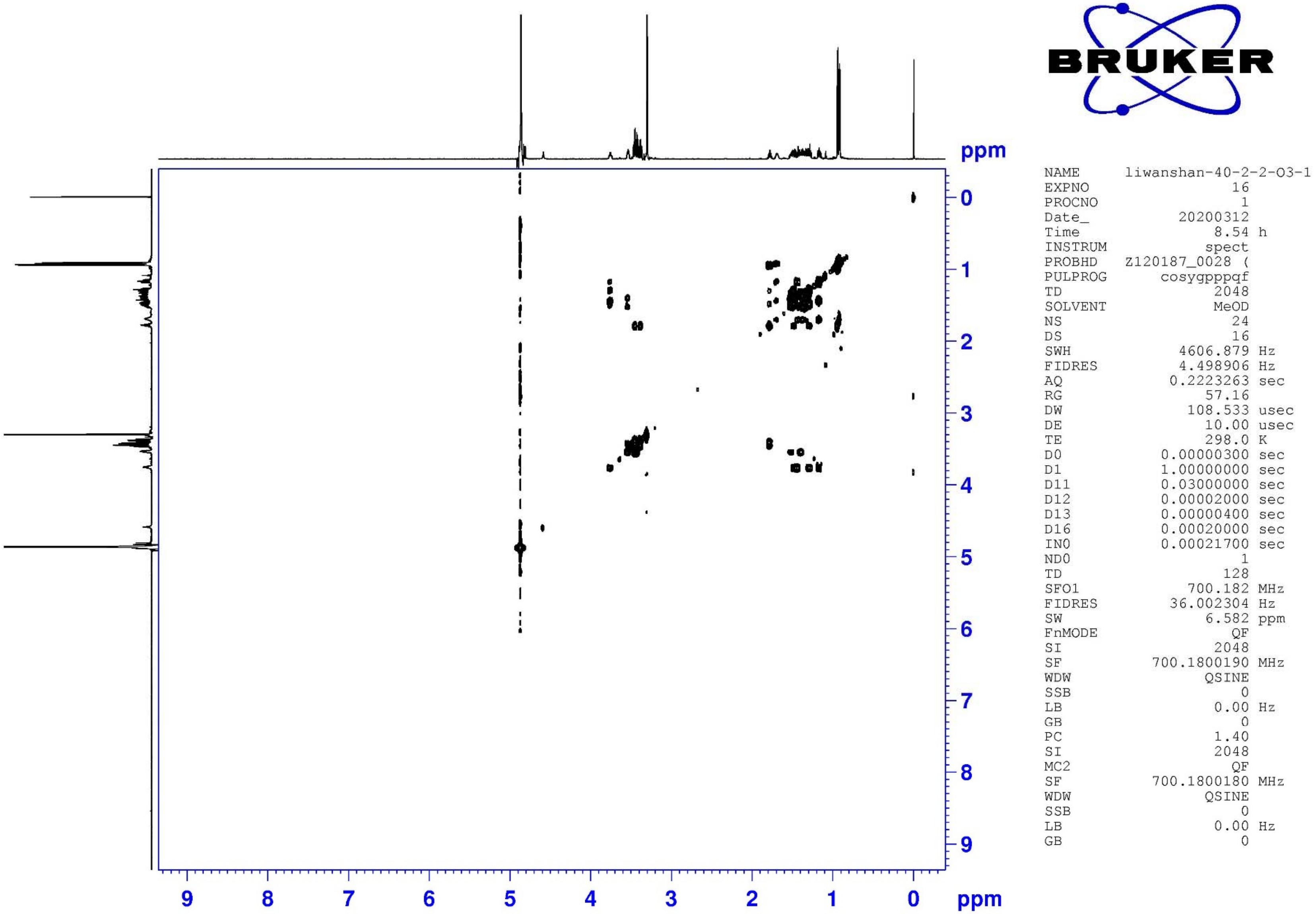

^{1}H–^{1}H COSY (700 MHz) spectrum of the fragment **1a** in CD_3OD

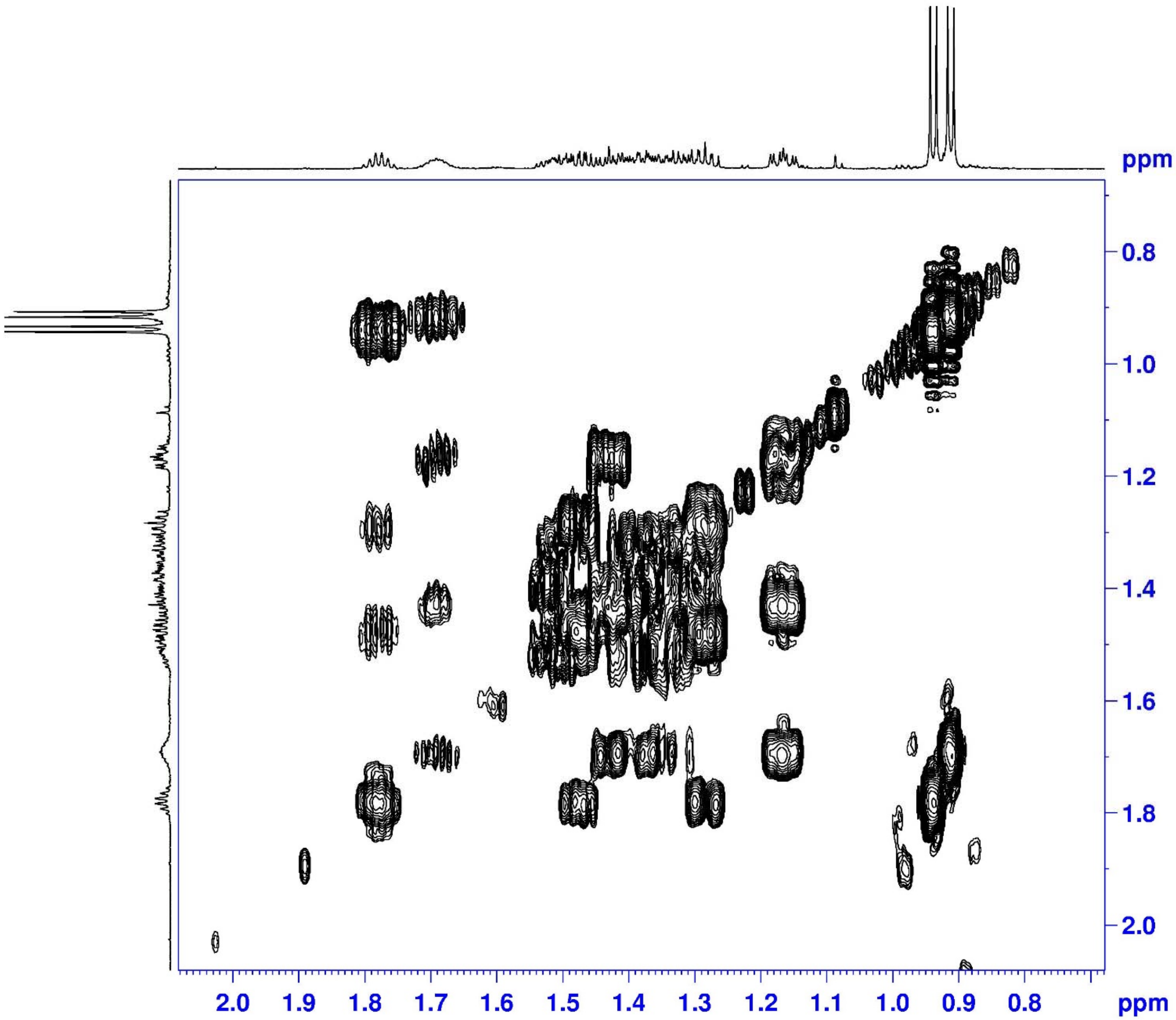

^{1}H–^{1}H COSY (700 MHz) spectrum of the fragment **1a** in CD_3OD

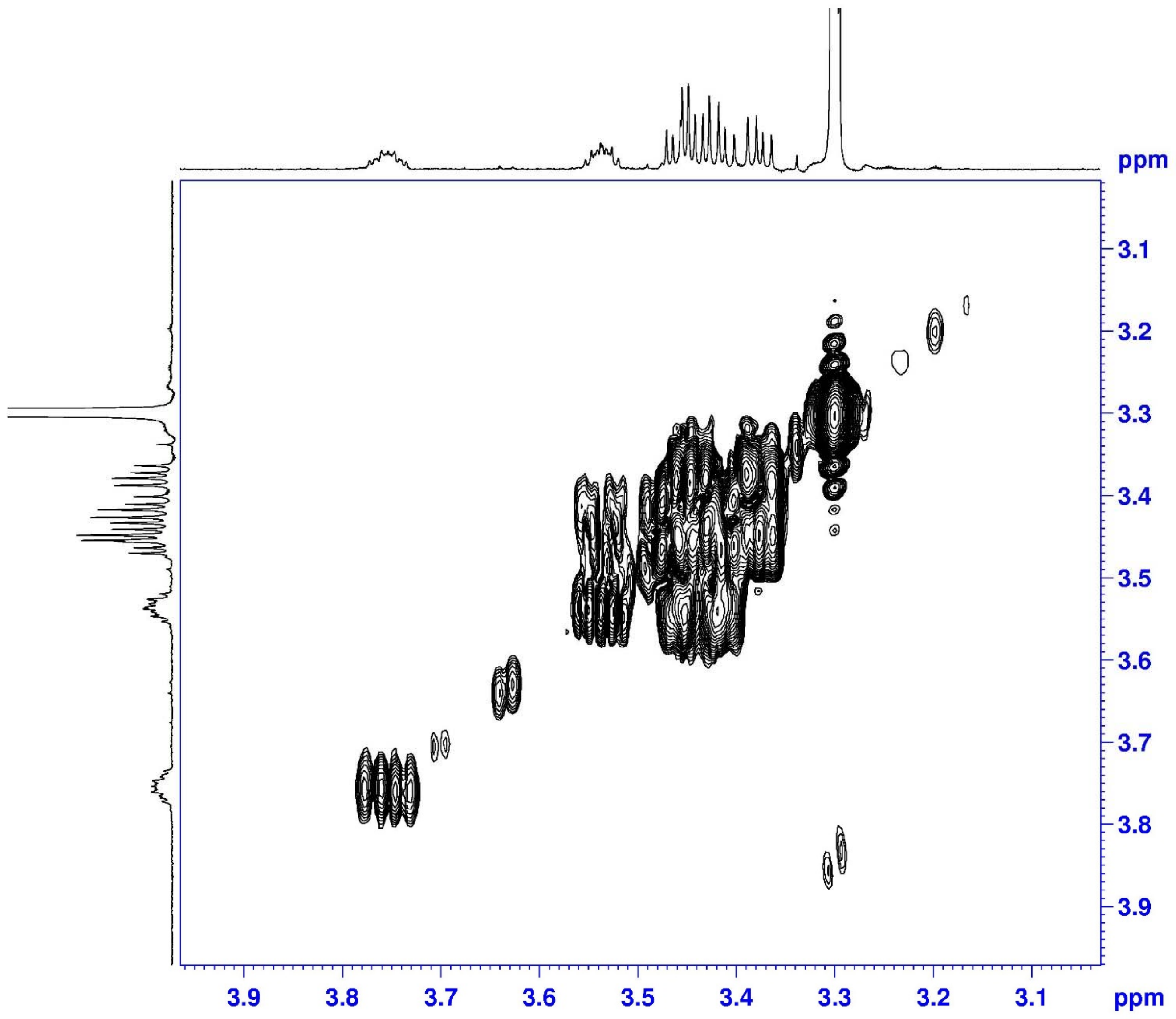

$^{1}H–^{1}H$ COSY (700 MHz) spectrum of the fragment **1a** in CD_3OD

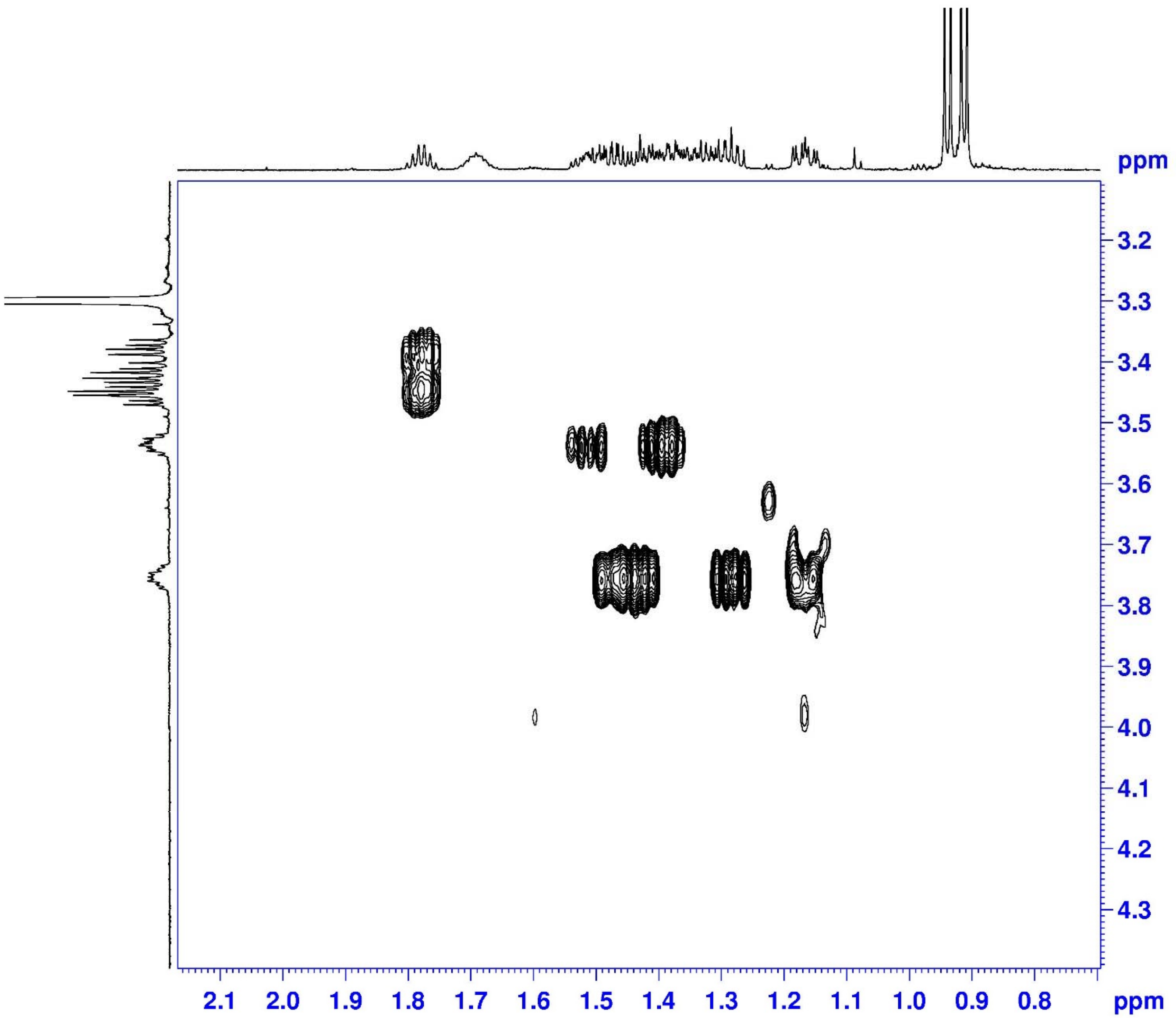

HSQC (700 MHz) spectrum of the fragment **1a** in CD_3OD

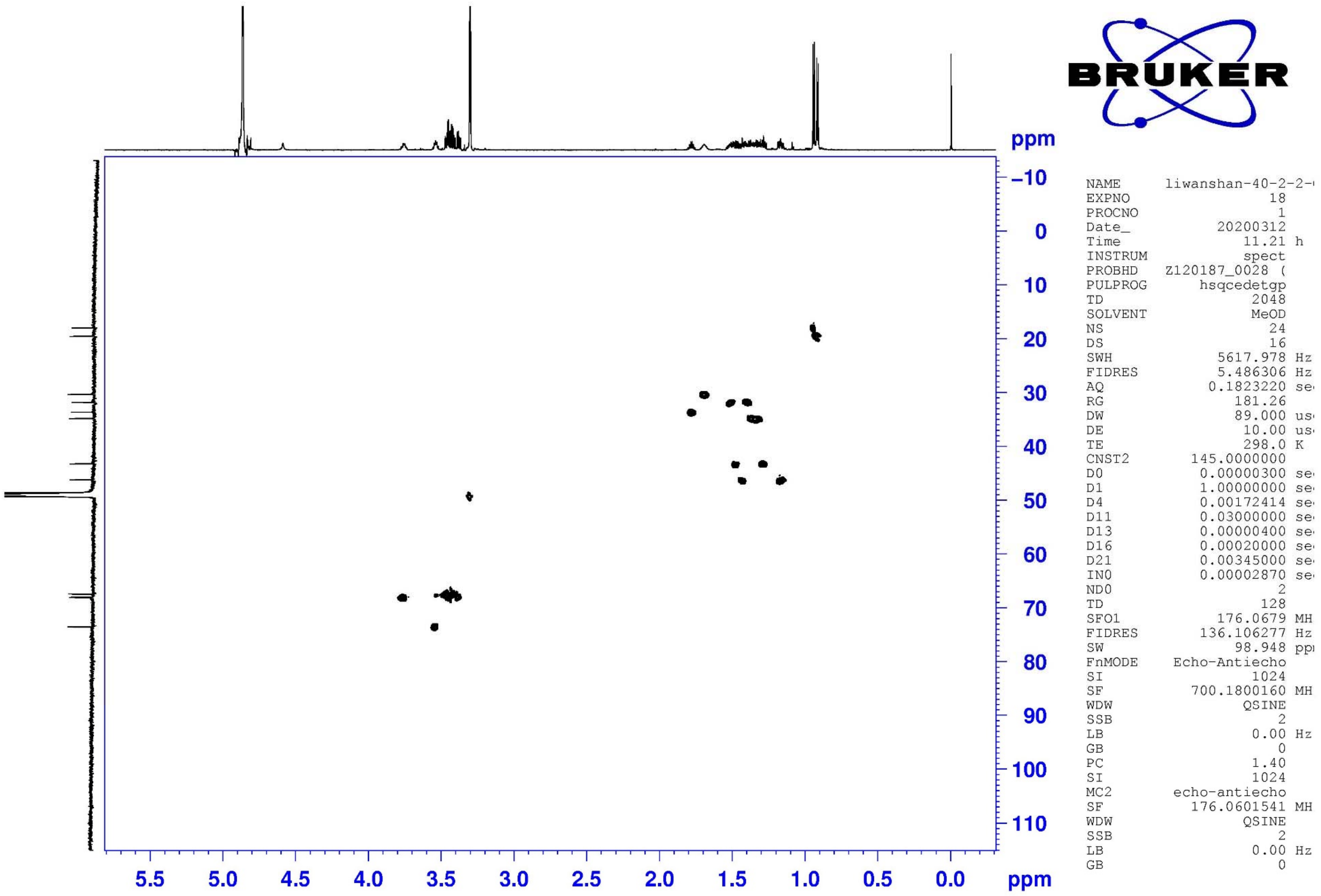

HSQC (700 MHz) spectrum of the fragment **1a** in CD_3OD

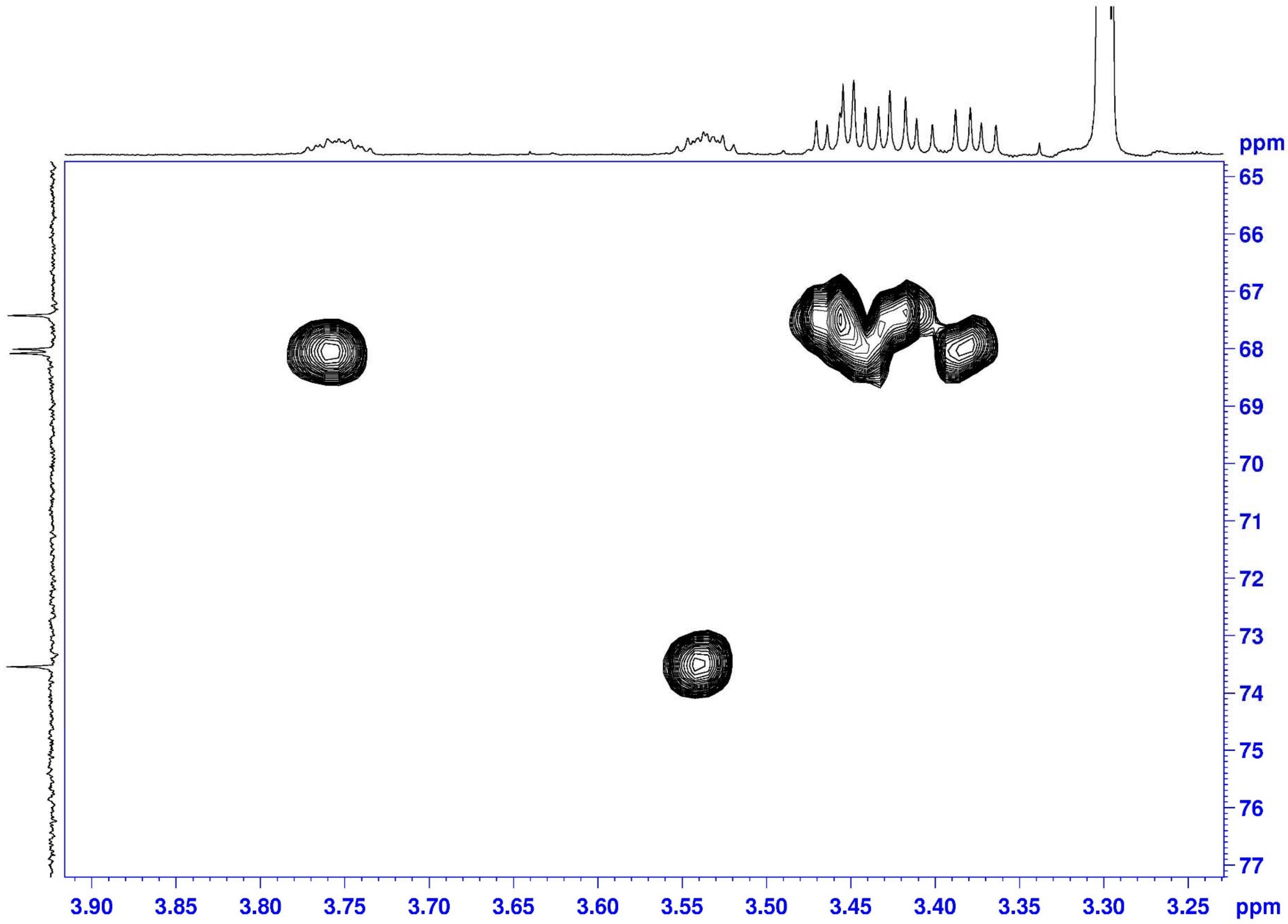

HSQC (700 MHz) spectrum of the fragment **1a** in CD_3OD

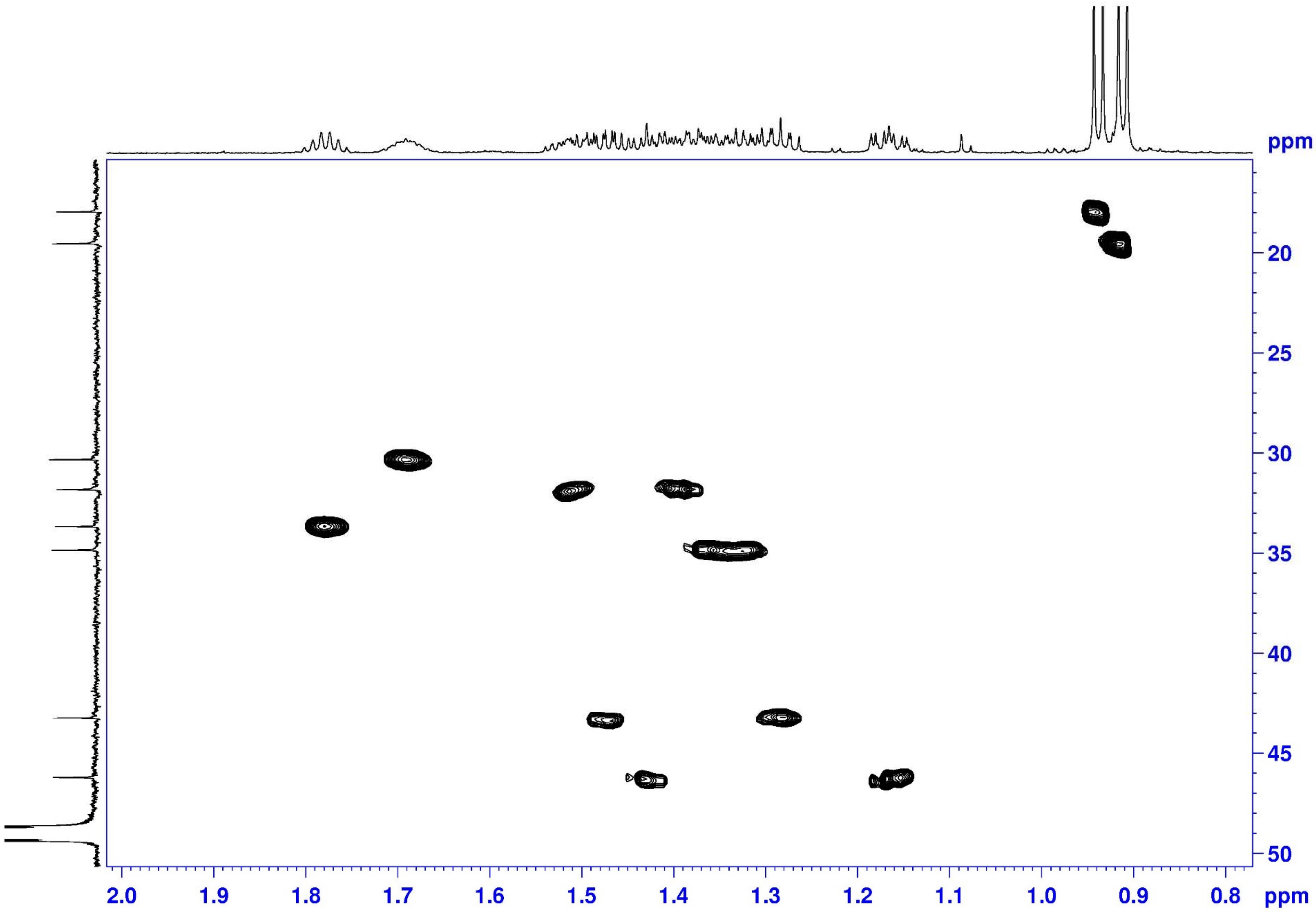

HMBC (700 MHz) spectrum of the fragment **1a** in CD_3OD

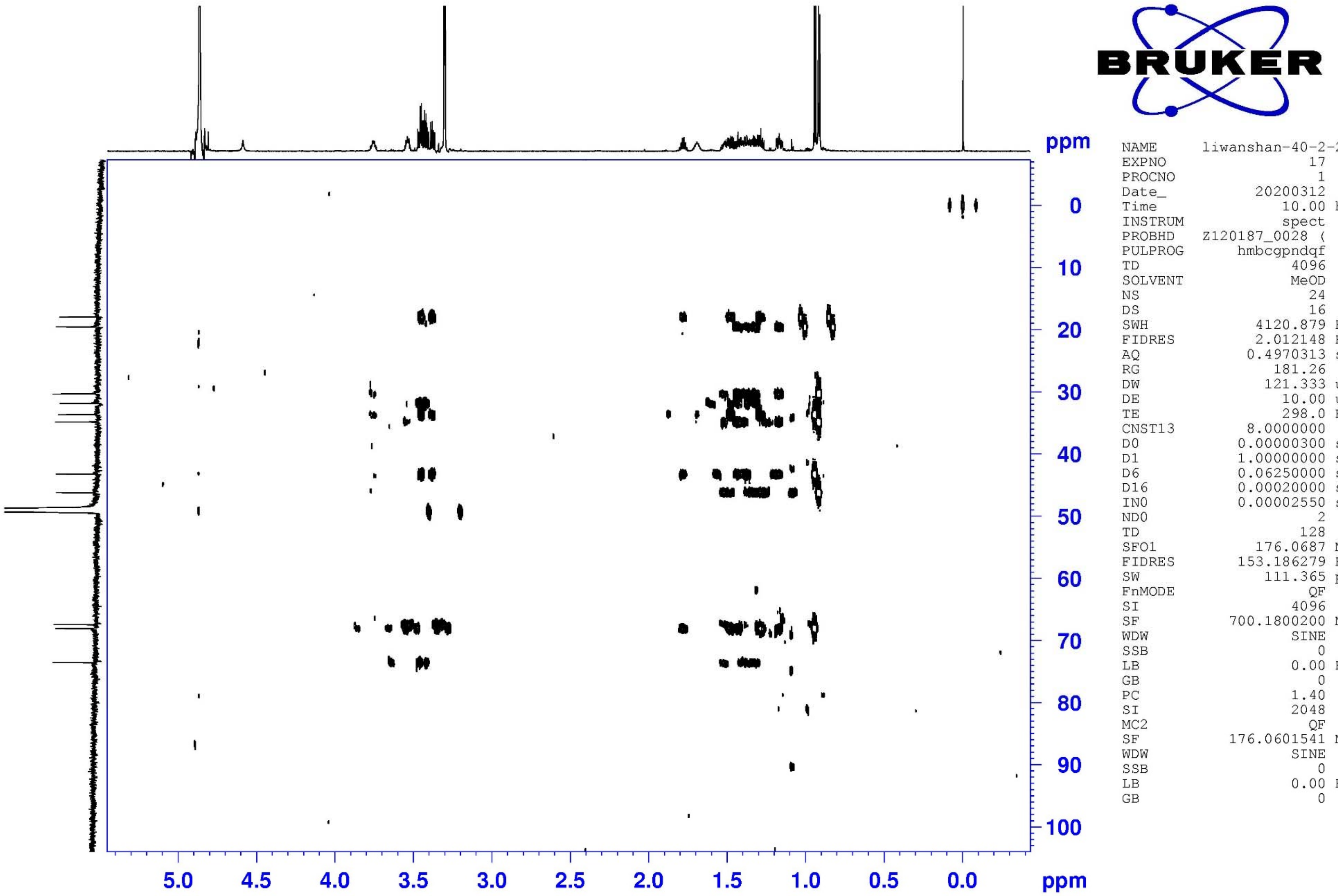

HMBC (700 MHz) spectrum of the fragment **1a** in CD_3OD

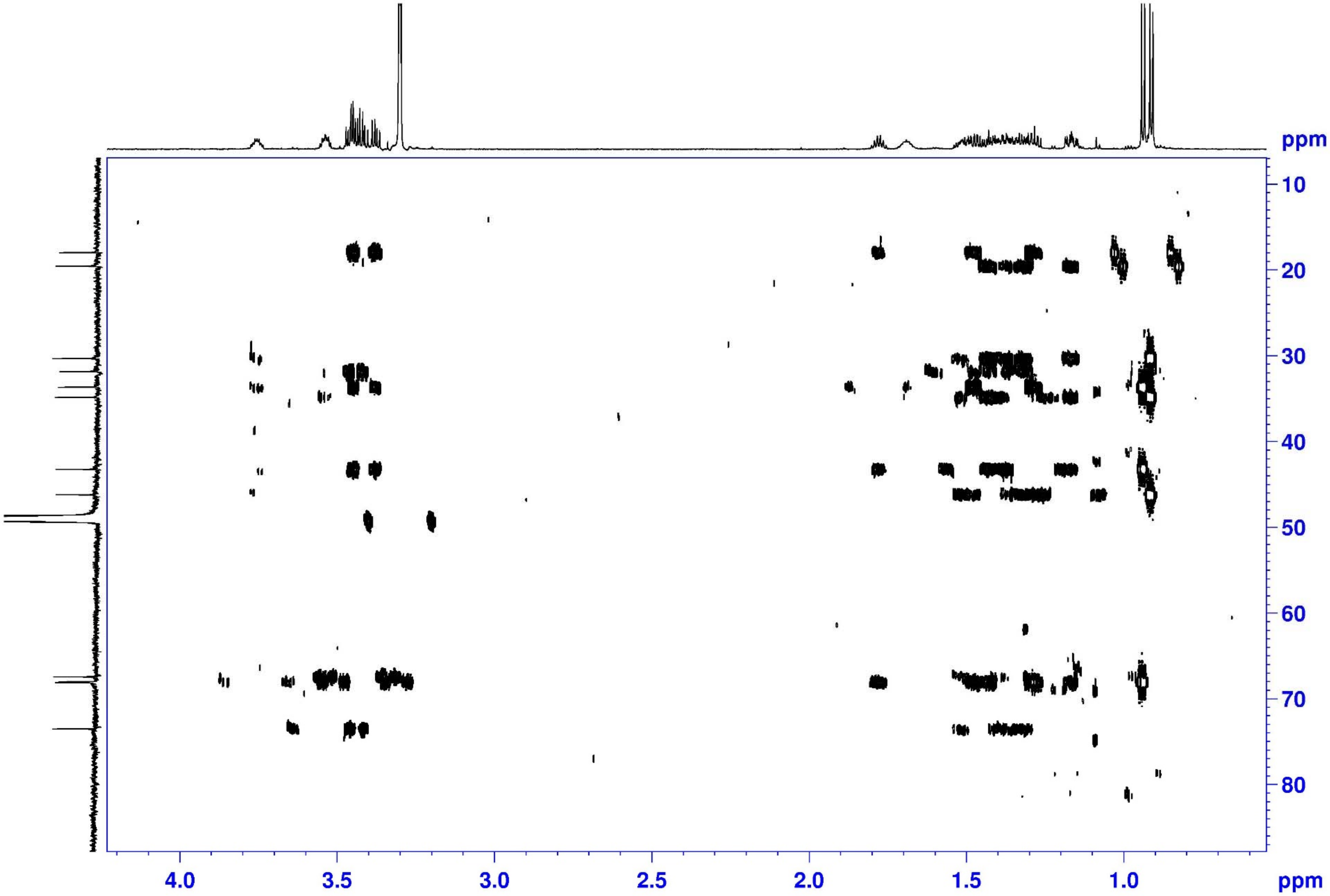

2D *J*RES (700 MHz) spectrum of the fragment **1a** in CD_3OD

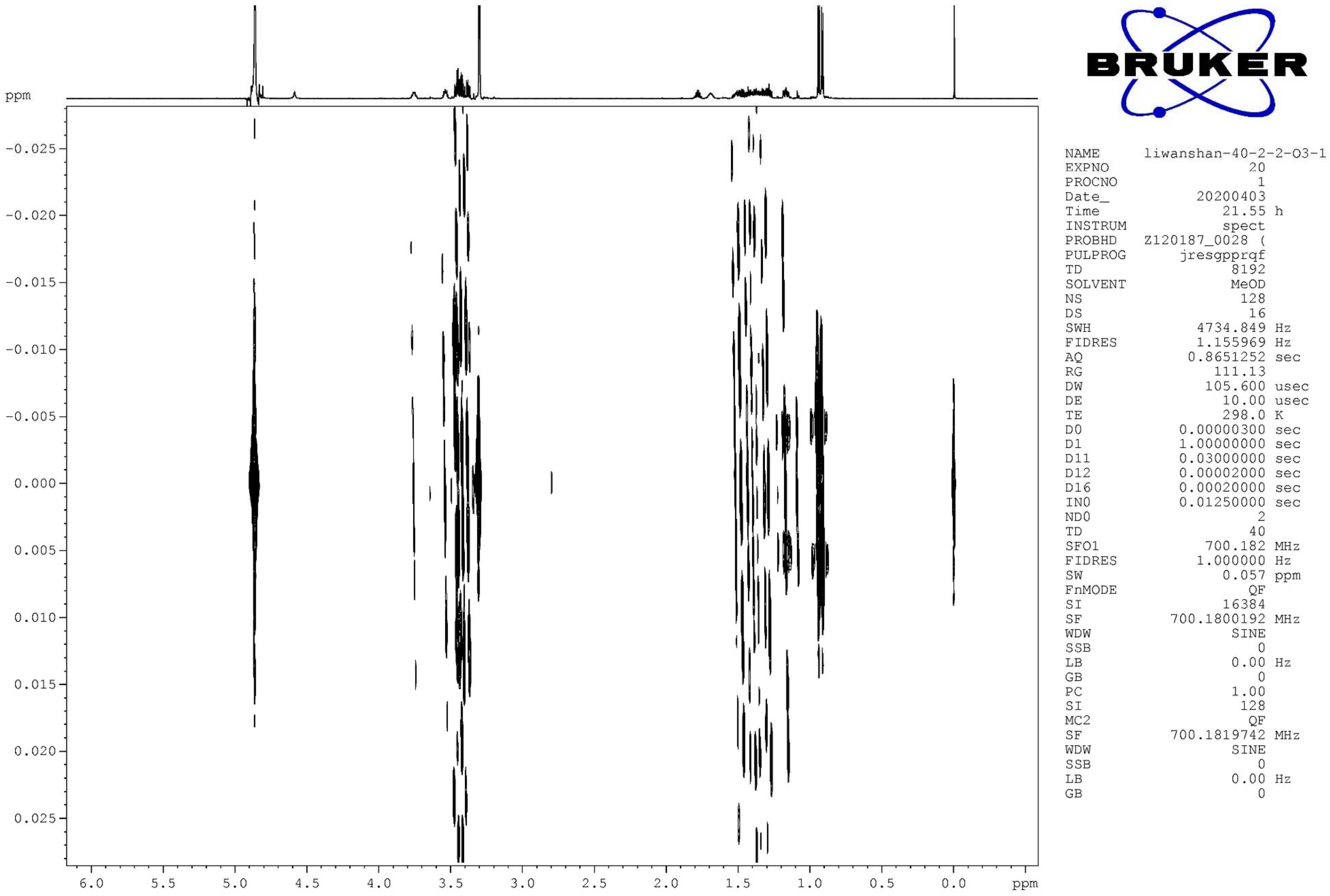

2D *J*RES (700 MHz) spectrum of the fragment **1a** in CD_3OD

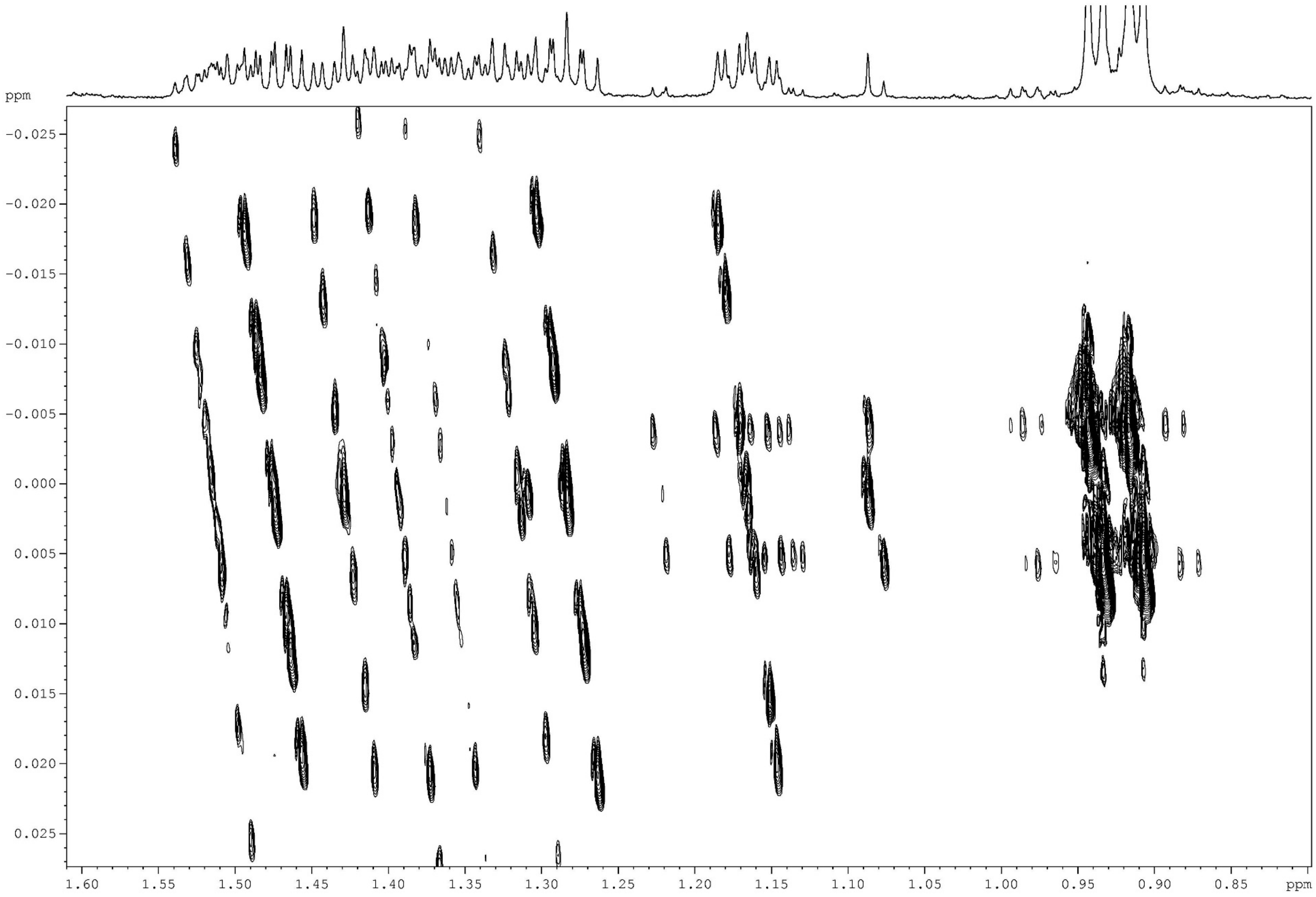

2D *J*RES (700 MHz) spectrum of the fragment **1a** in CD_3OD

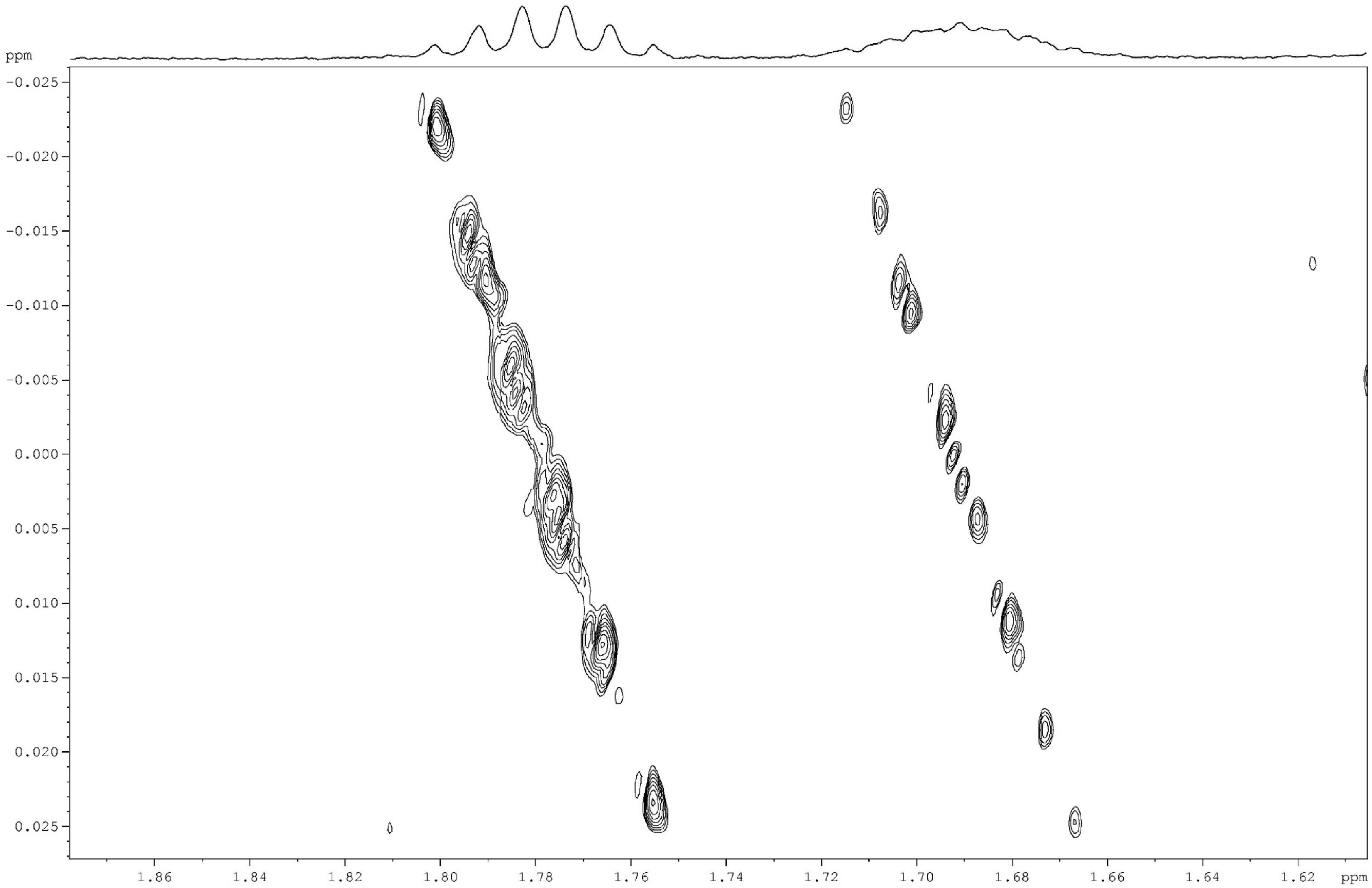

2D *J*RES (700 MHz) spectrum of the fragment **1a** in CD_3OD

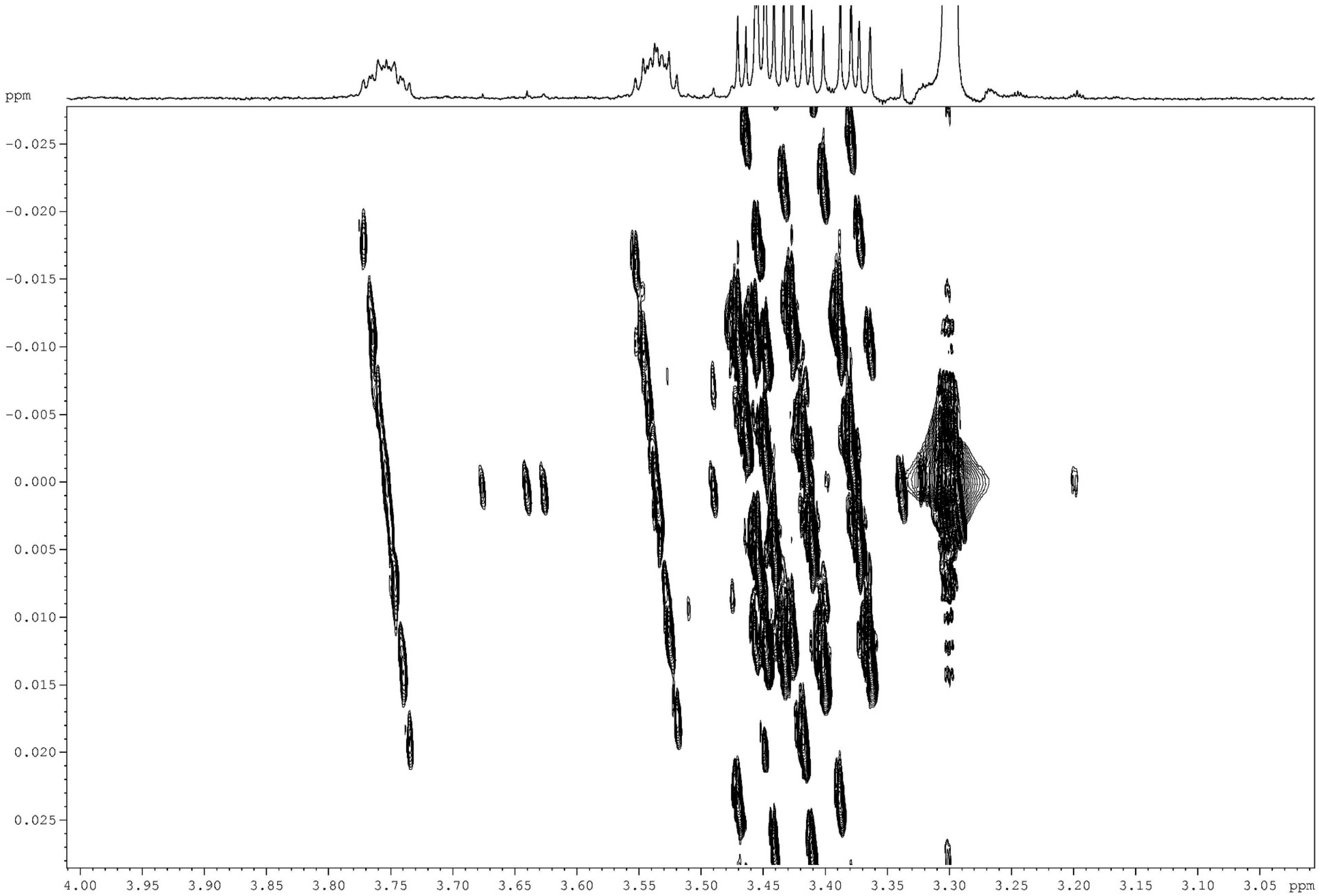

HETLOC (700 MHz) spectrum of the fragment **1a** in CD_3OD

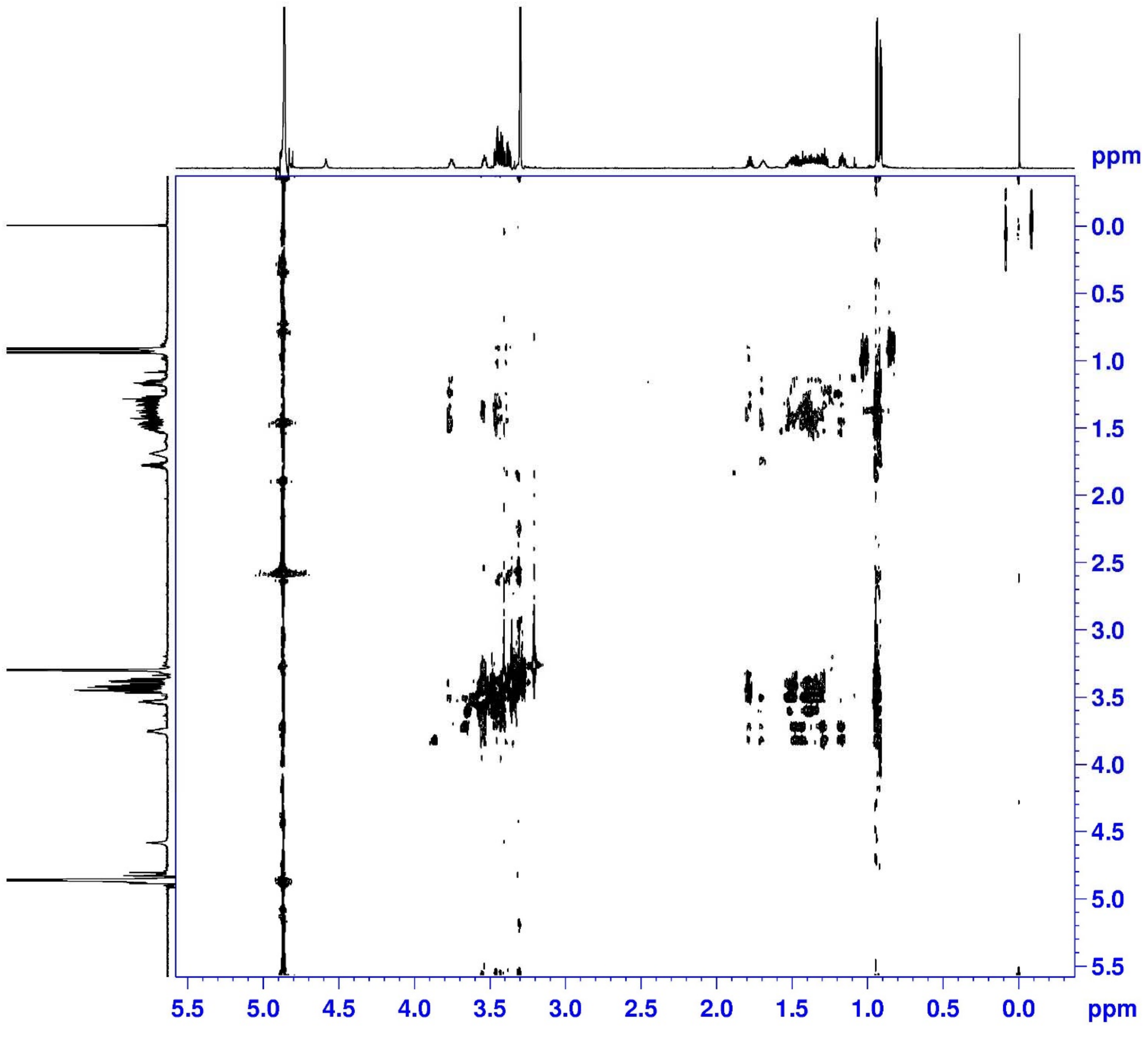

HETLOC (700 MHz) spectrum of the fragment **1a** in CD_3OD

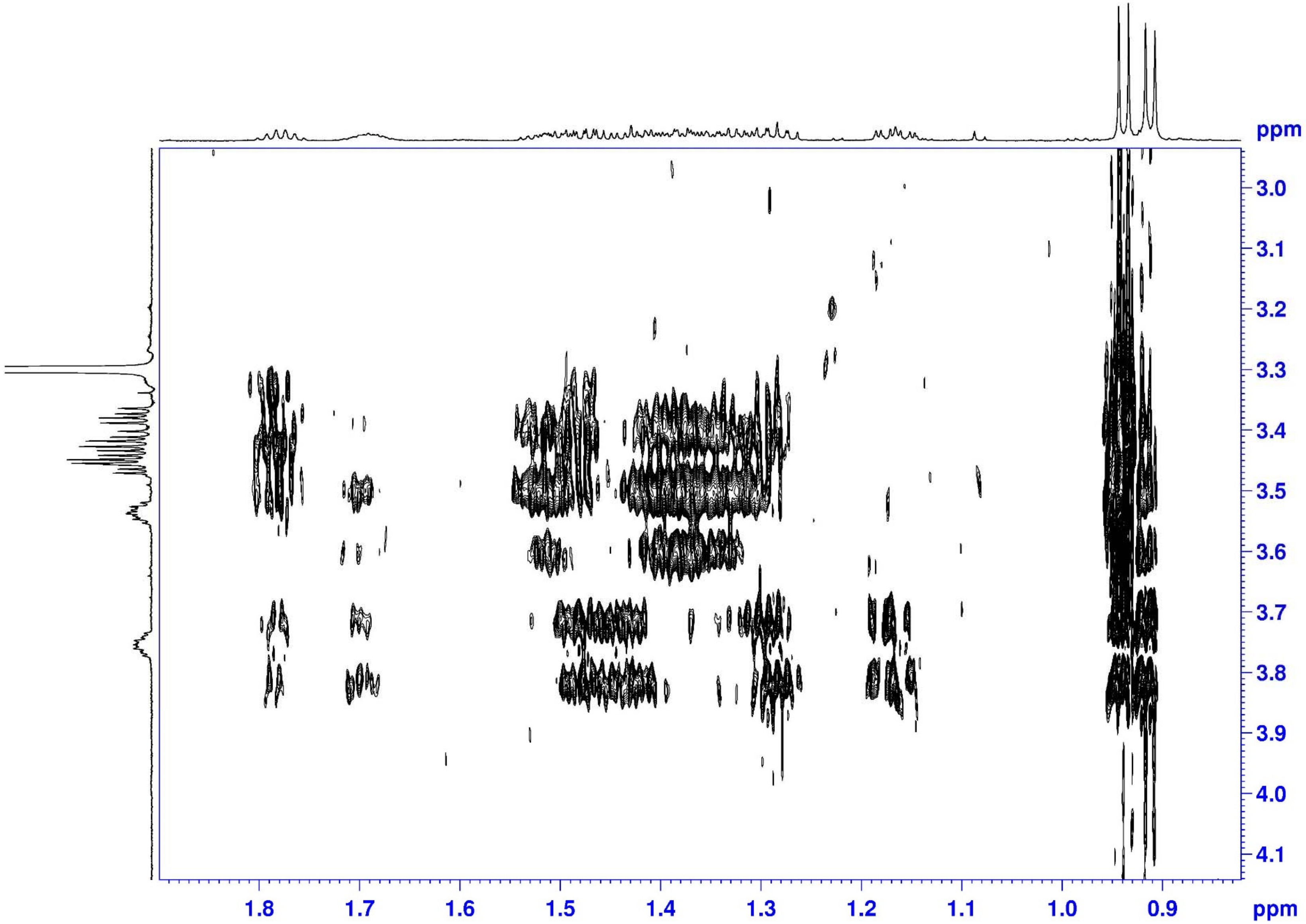

HETLOC (700 MHz) spectrum of the fragment **1a** in CD_3OD

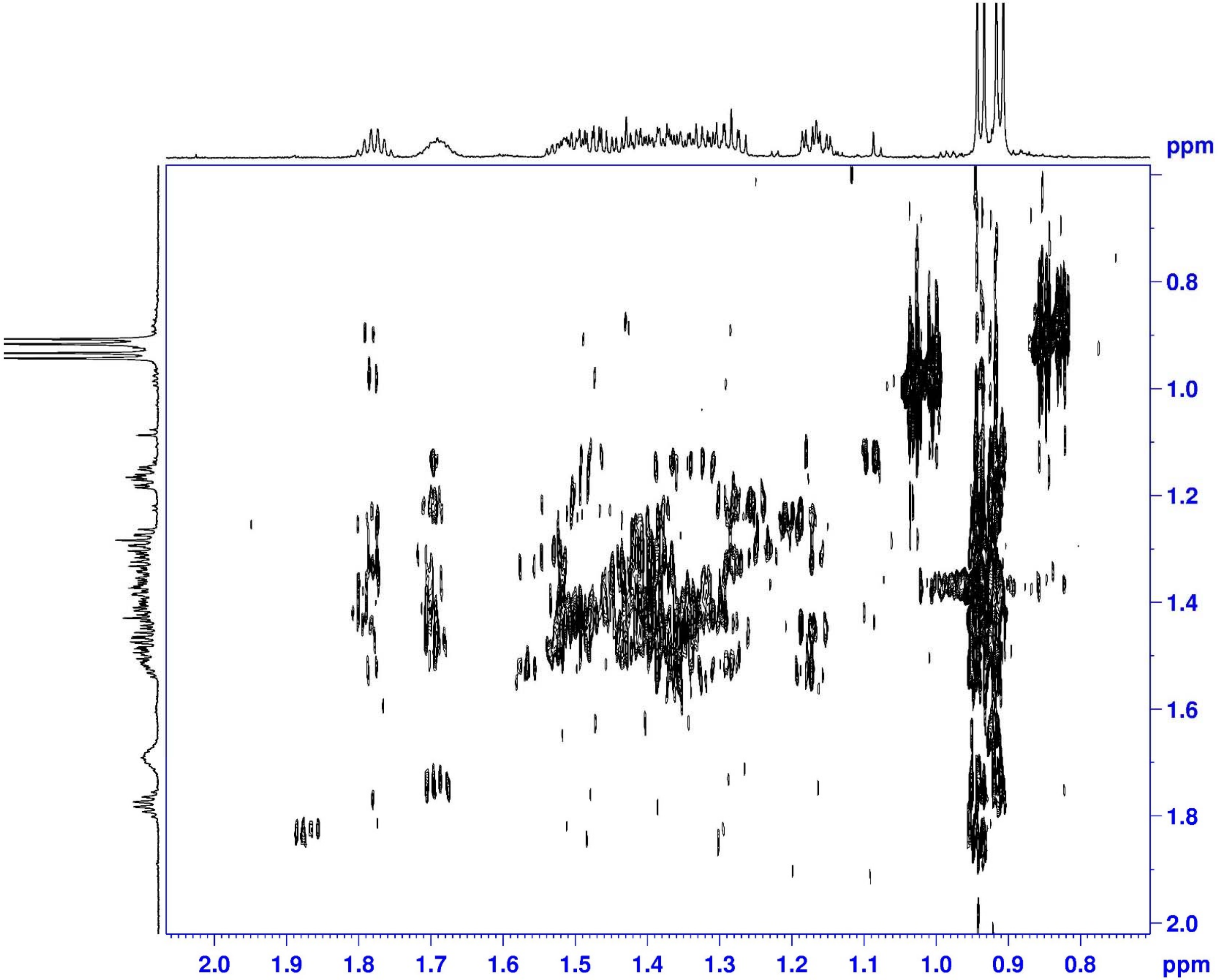

HETLOC (700 MHz) spectrum of the fragment **1a** in CD_3OD

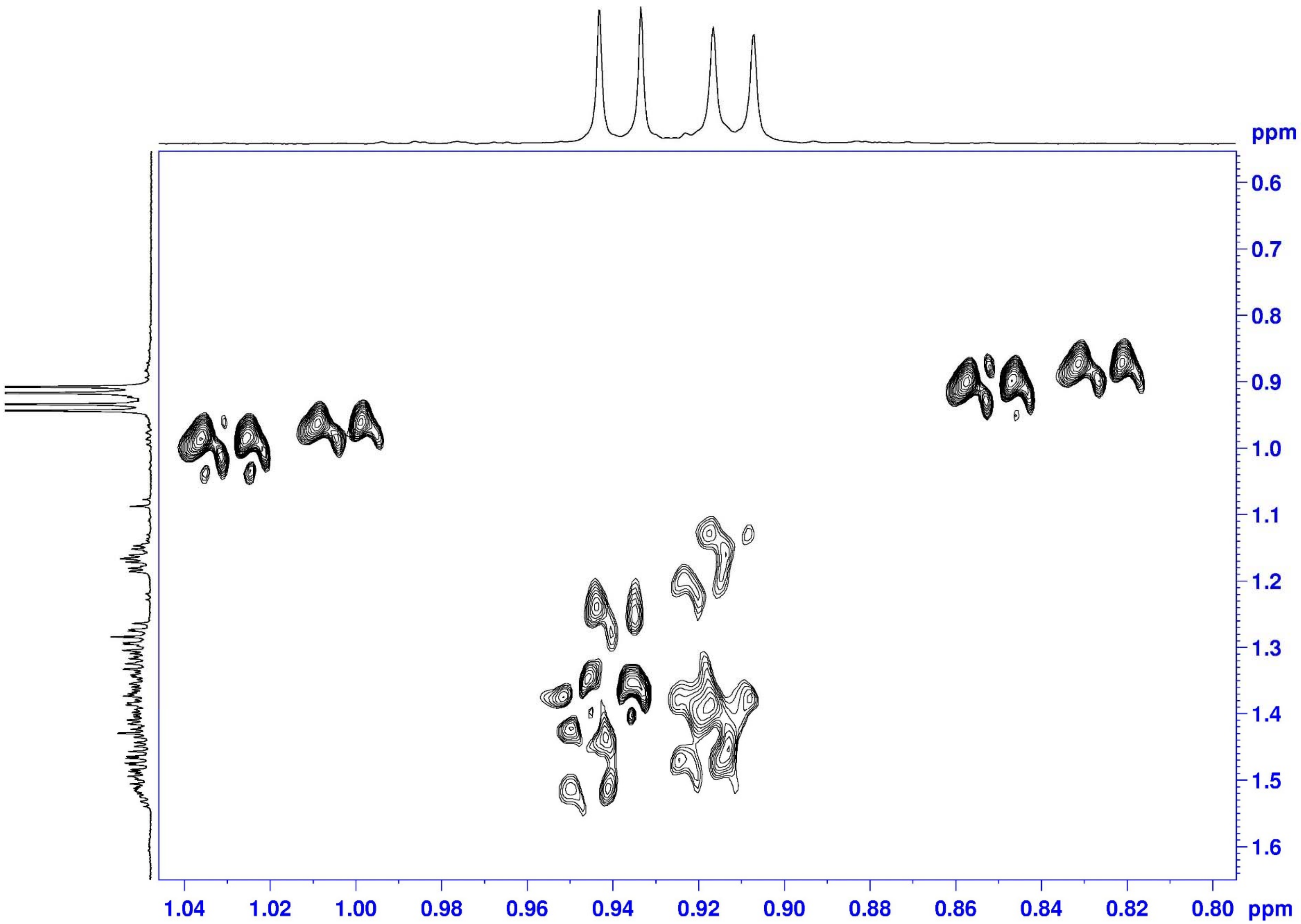

HETLOC (700 MHz) spectrum of the fragment **1a** in CD_3OD

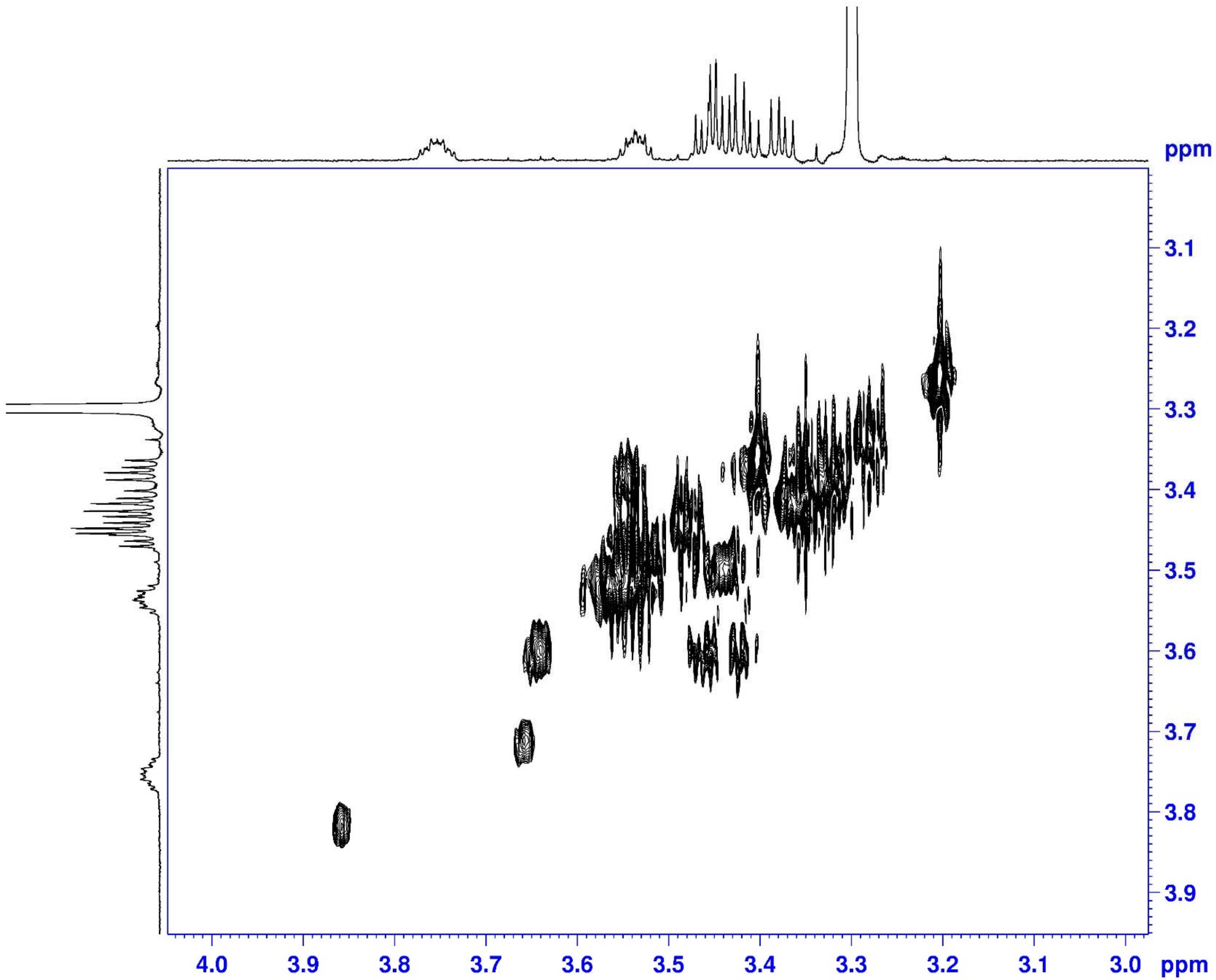

LR-ESI-MS for the fragment **1b**

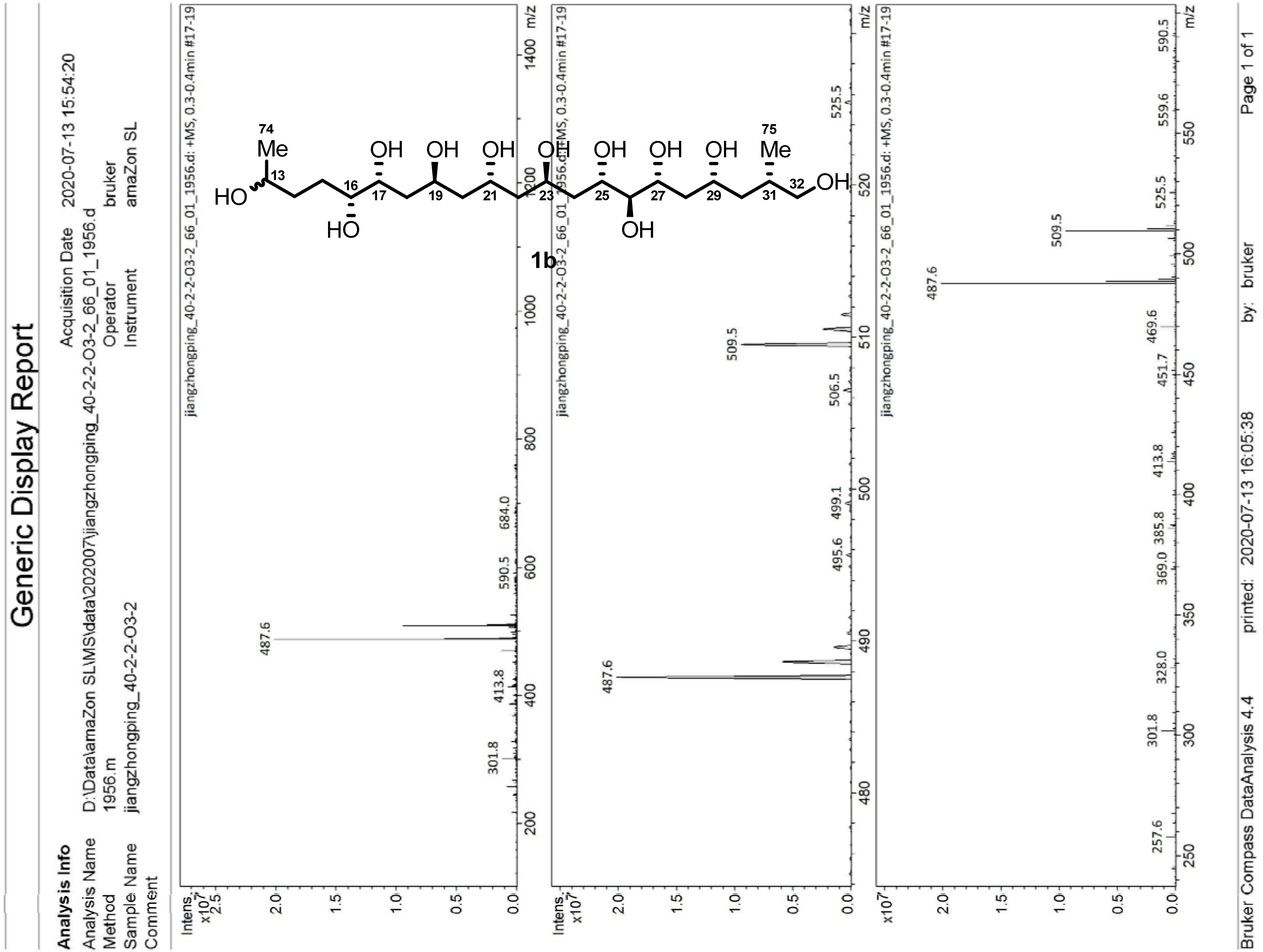

^{1}H (700 MHz) NMR spectrum of the fragment **1b** in CD_3OD

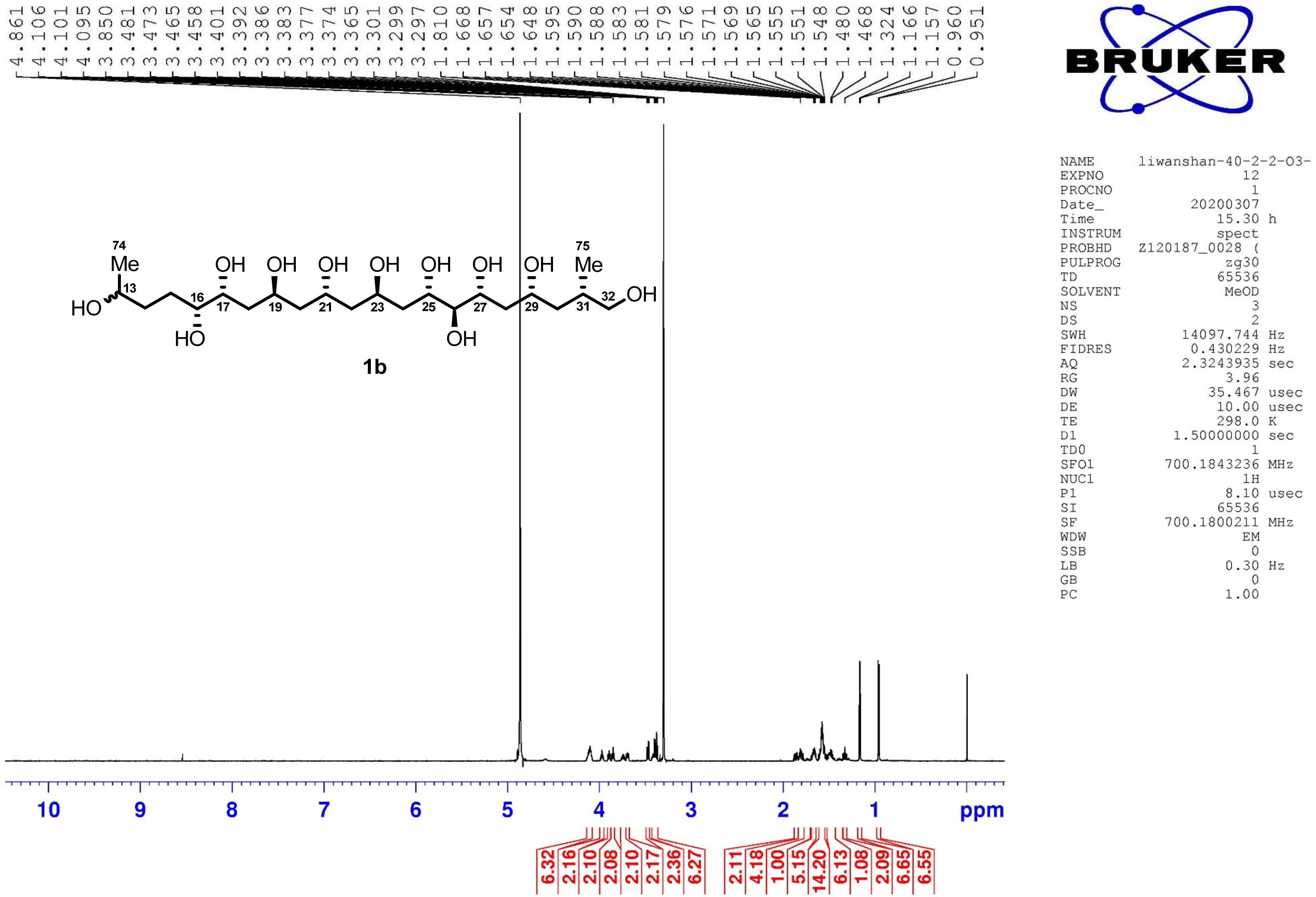

^{1}H (700 MHz) NMR spectrum of the fragment **1b** in CD_3OD

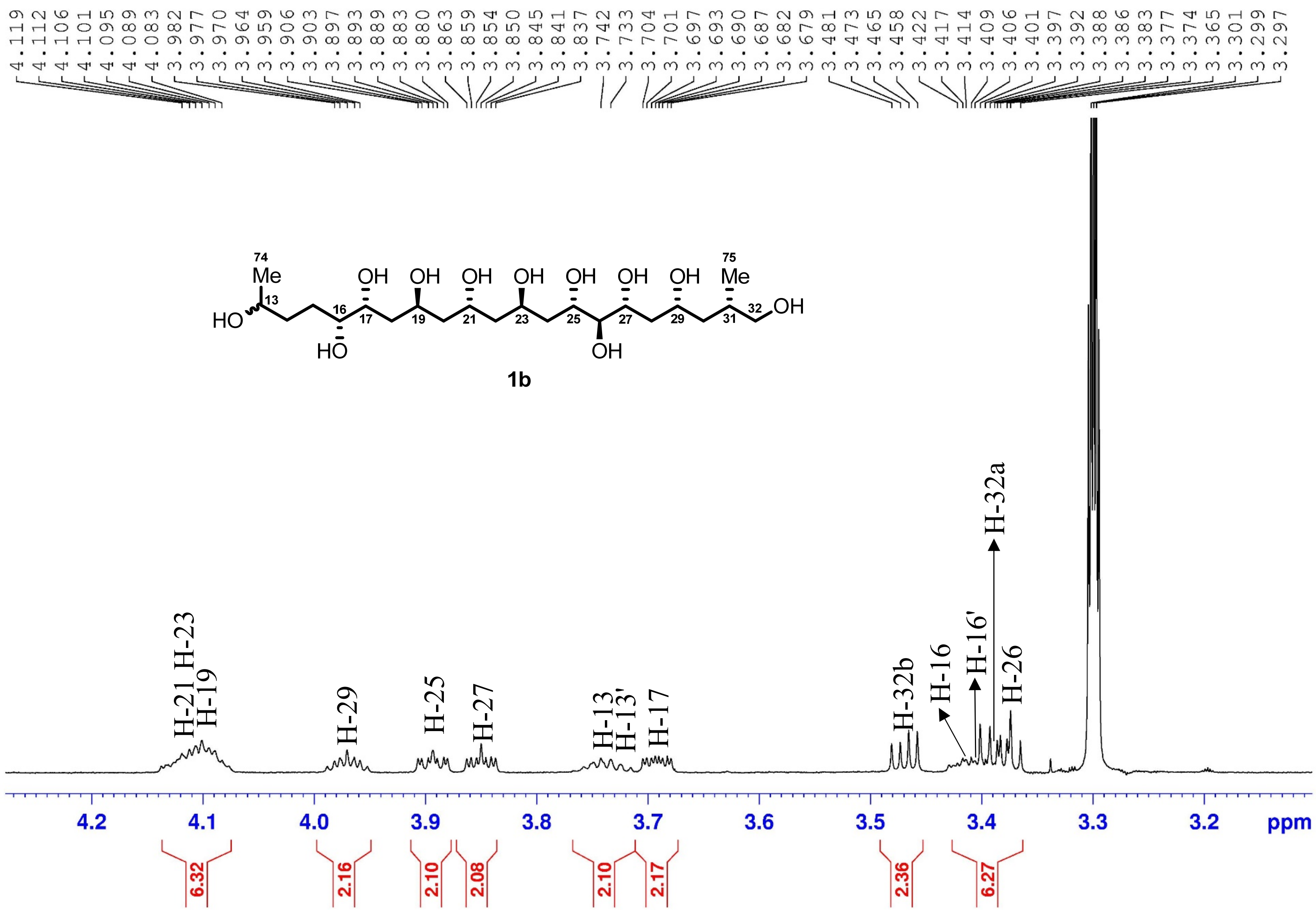

^{1}H (700 MHz) NMR spectrum of the fragment **1b** in CD_3OD

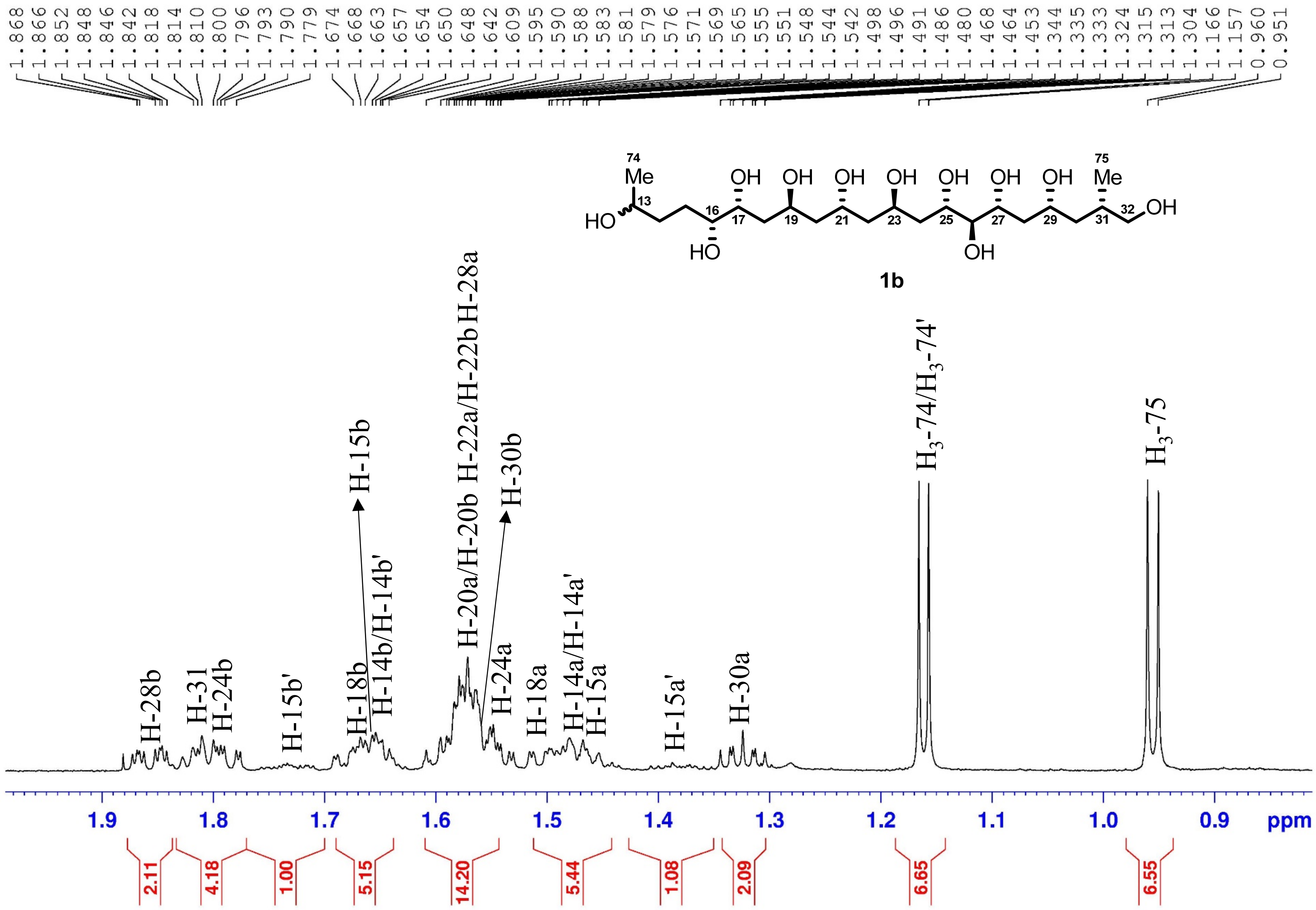

^{13}C (175 MHz) NMR spectrum of the fragment **1b** in CD_3OD

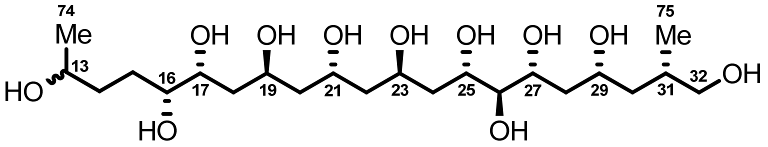

1b

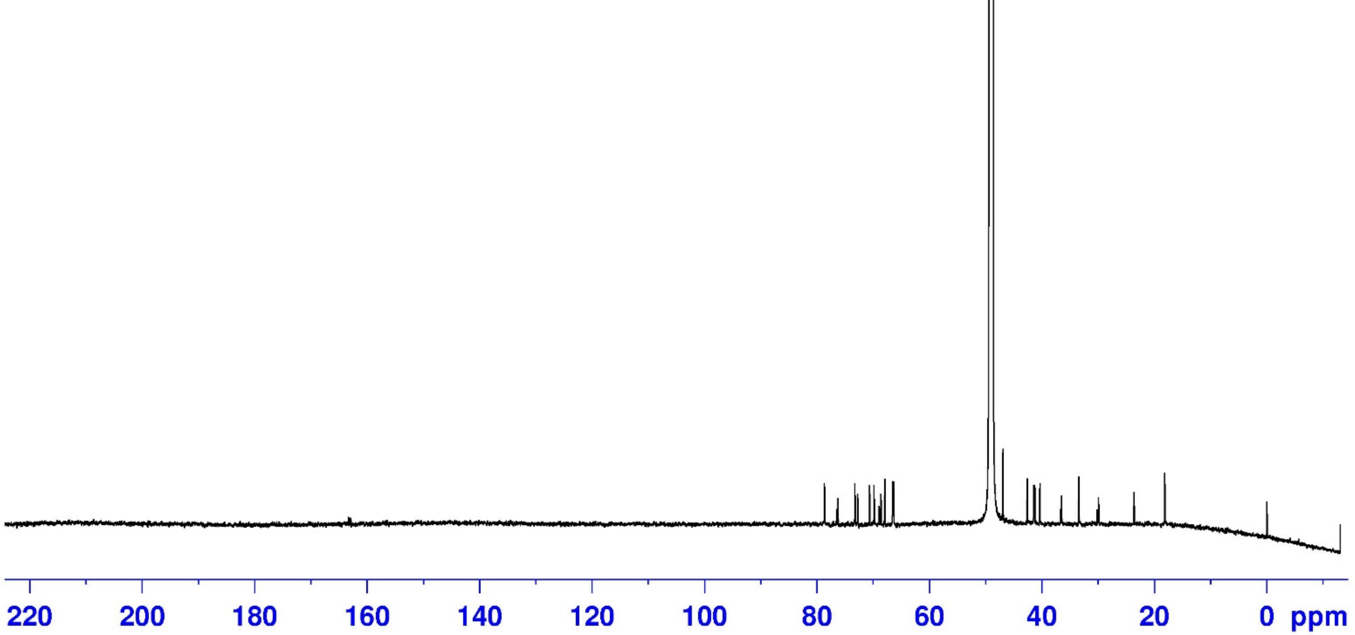

NAME	liwanshan-40-2-2-O
EXPNO	20
PROCNO	1
Date_	20200321
Time	5.36 h
INSTRUM	spect
PROBHD	Z120187_0028 (
PULPROG	zgpg30
TD	32768
SOLVENT	MeOD
NS	10000
DS	8
SWH	43859.648 Hz
FIDRES	2.676980 Hz
AQ	0.3736052 sec
RG	181.26
DW	11.400 use
DE	18.00 use
TE	298.0 K
D1	1.00000000 sec
D11	0.03000000 sec
TD0	1
SFO1	176.0797677 MHz
NUC1	13C
P1	11.90 use
SI	32768
SF	176.0601446 MHz
WDW	EM
SSB	0
LB	5.00 Hz
GB	0
PC	1.40

^{13}C (175 MHz) NMR spectrum of the fragment **1b** in CD_3OD

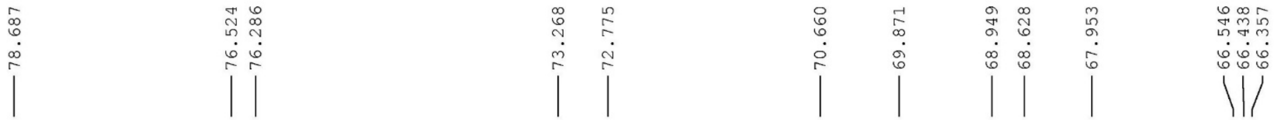

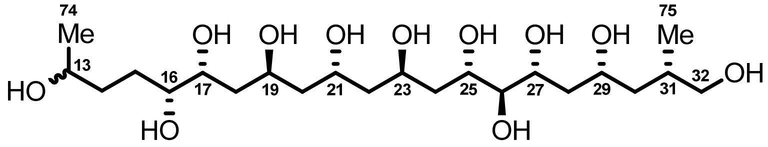

1b

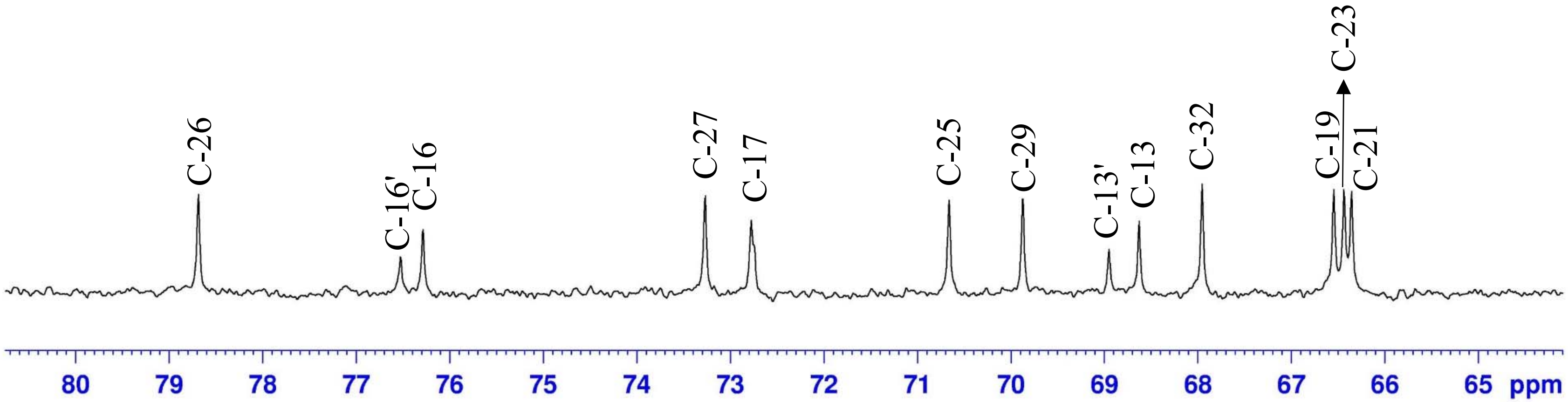

^{13}C (175 MHz) NMR spectrum of the fragment **1b** in CD_3OD

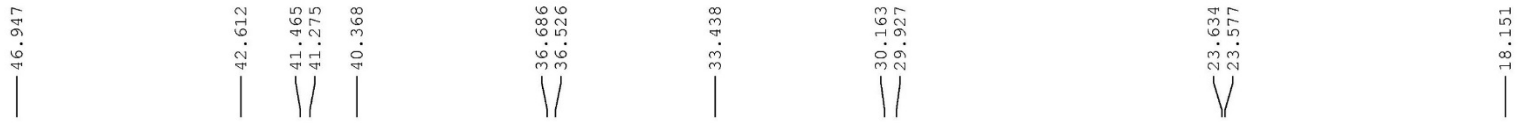

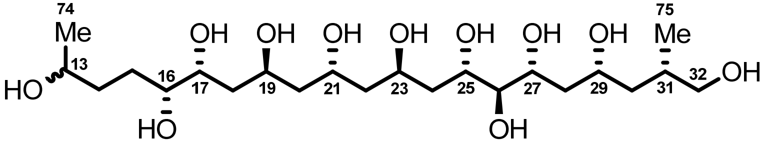

1b

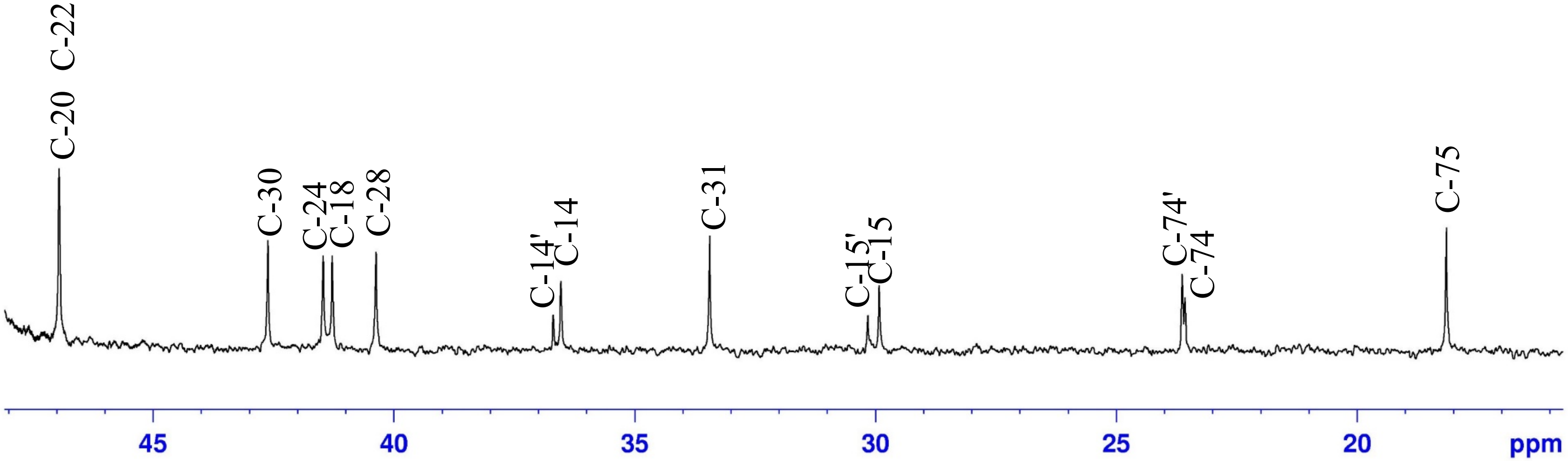

DEPT135 (175 MHz) spectrum of the fragment **1b** in CD_3OD

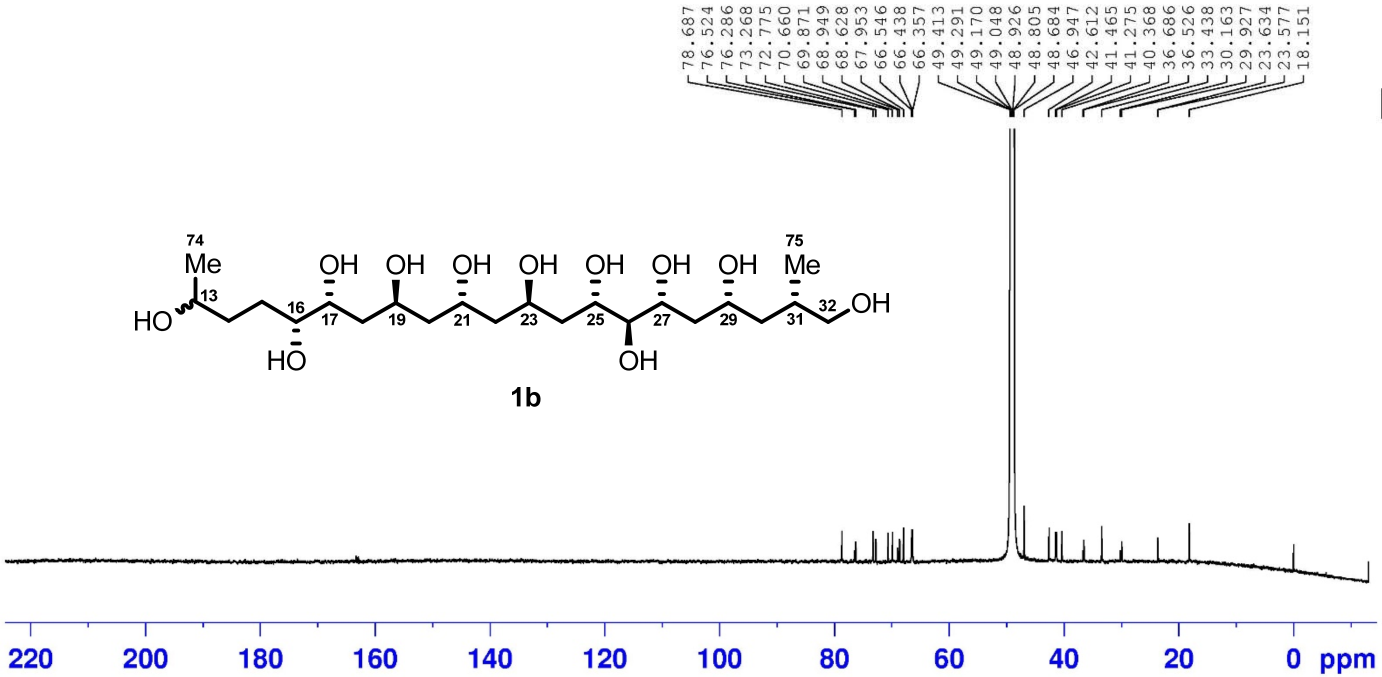

NAME	liwanshan-40-2-2-
EXPNO	21
PROCNO	1
Date_	20200321
Time	7.12 h
INSTRUM	spect
PROBHD	Z120187_0028 (
PULPROG	deptsp135
TD	32768
SOLVENT	MeOD
NS	4000
DS	8
SWH	43859.648 Hz
FIDRES	2.676980 Hz
AQ	0.3736052 se
RG	181.26
DW	11.400 us
DE	18.00 us
TE	298.0 K
CNST2	145.0000000
D1	1.00000000 se
D2	0.00344828 se
D12	0.00002000 se
TD0	1
SFO1	176.0797677 MH
NUC1	13C
P1	11.90 us
P13	2000.00 us
SI	32768
SF	176.0601437 MH
WDW	EM
SSB	0
LB	1.00 Hz
GB	0
PC	1.40

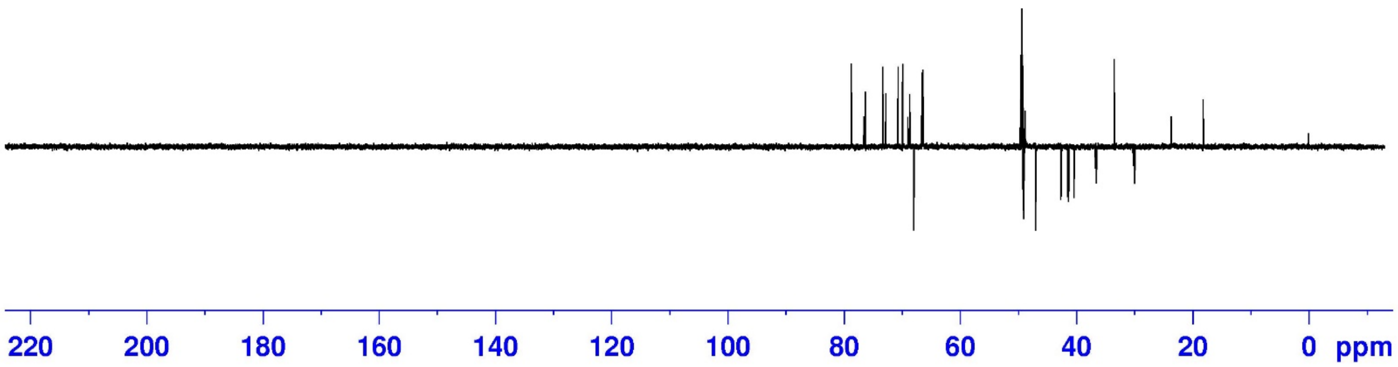

DEPT135 (175 MHz) spectrum of the fragment **1b** in CD_3OD

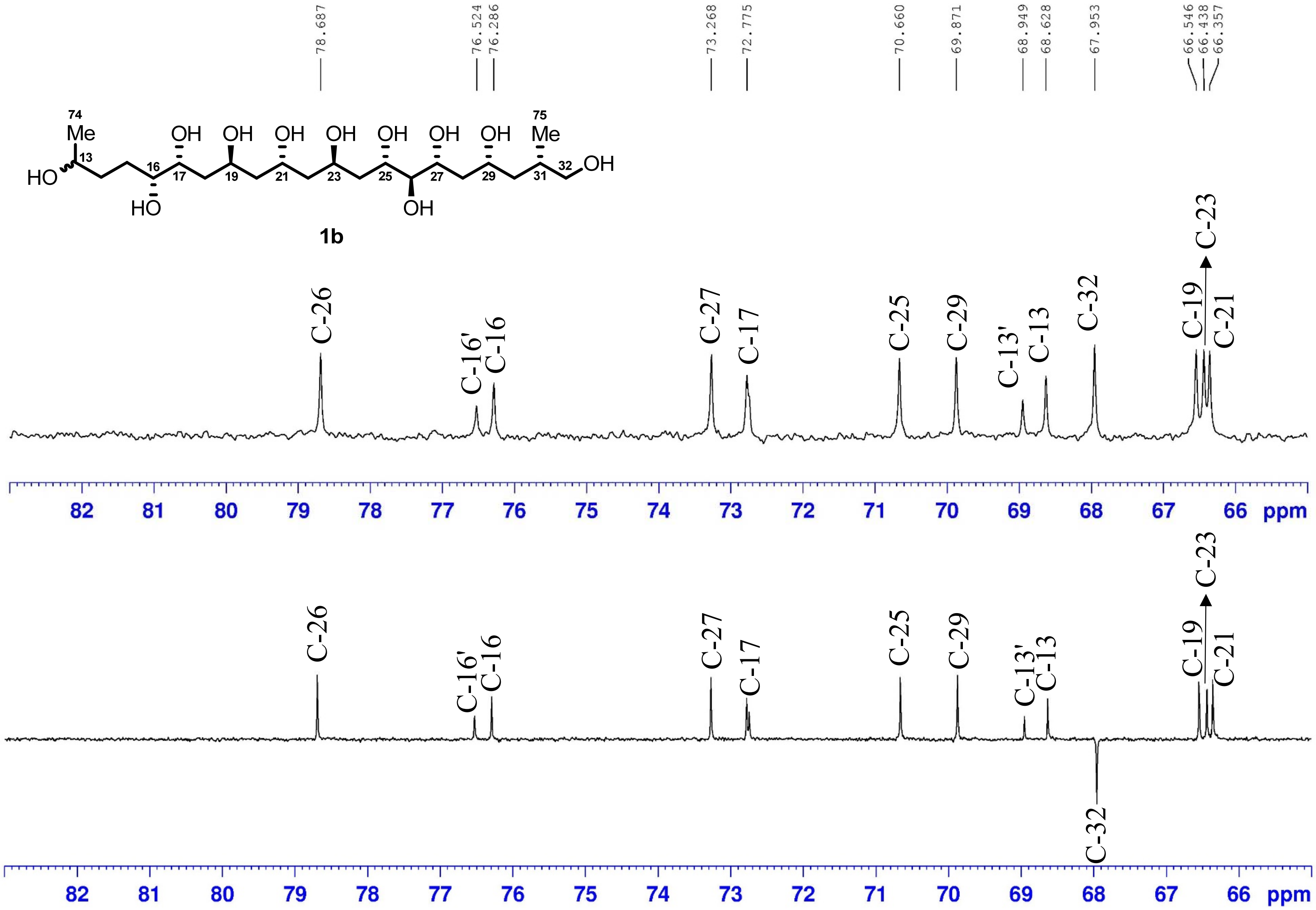

DEPT135 (175 MHz) spectrum of the fragment **1b** in CD_3OD

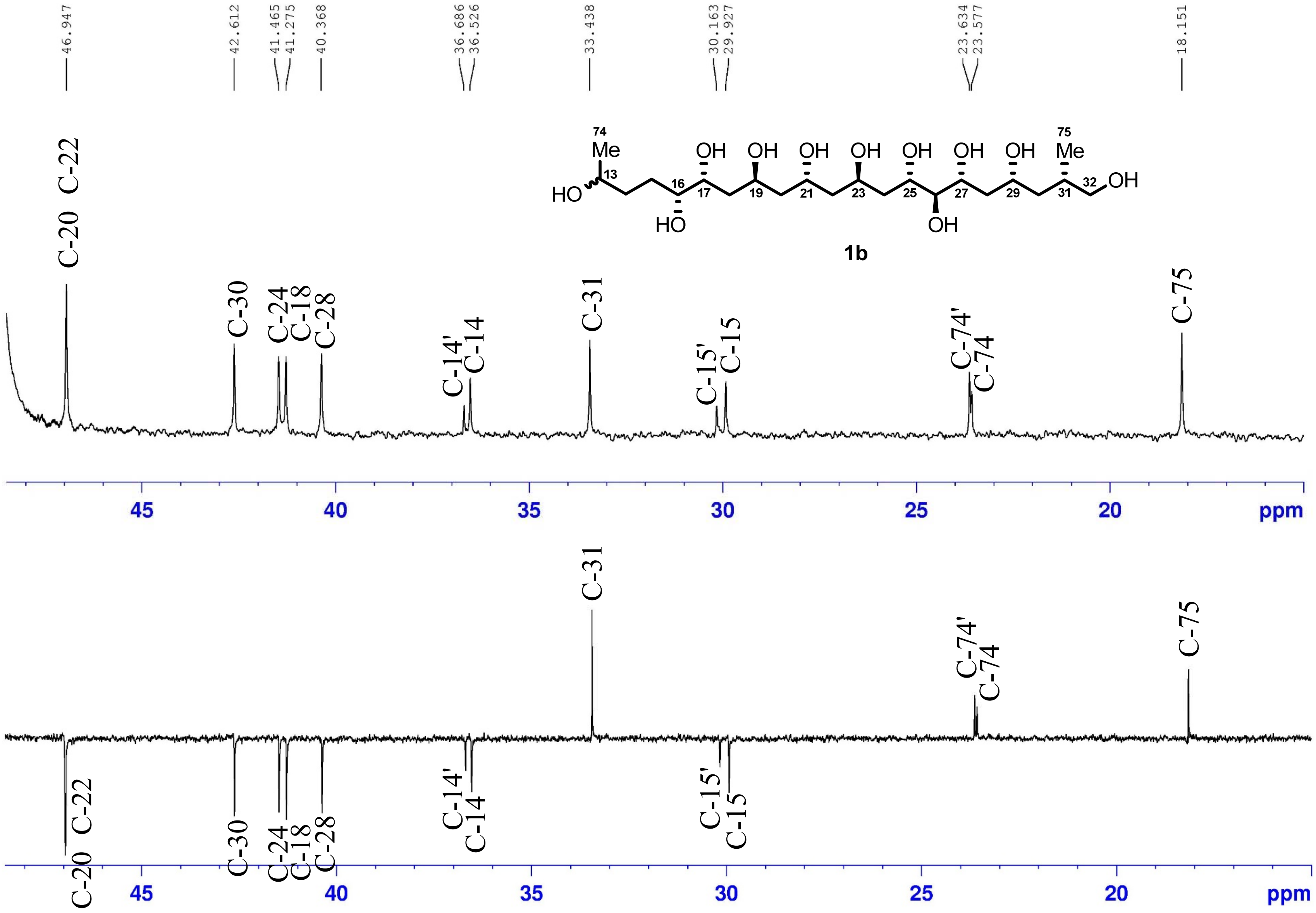

^{1}H–^{1}H COSY (700 MHz) spectrum of the fragment **1b** in CD_3OD

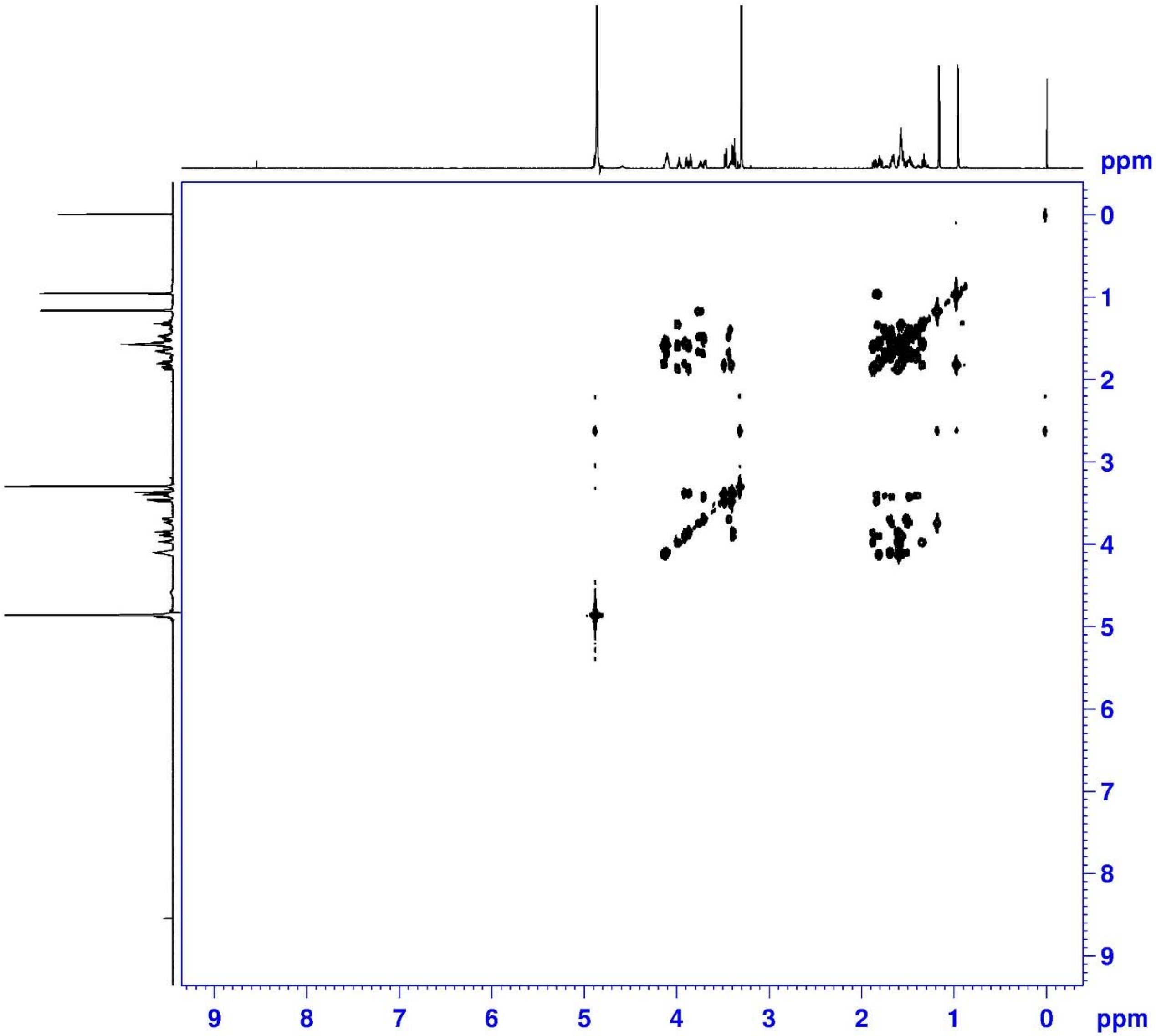

NAME	liwanshan-40-2-2-O3-2	
EXPNO	16	
PROCNO	1	
Date_	20200321	
Time	7.14	h
INSTRUM	spect	
PROBHD	Z120187_0028 (	
PULPROG	cosygpmfqf	
TD	2048	
SOLVENT	MeOD	
NS	32	
DS	16	
SWH	4347.826	Hz
FIDRES	4.245924	Hz
AQ	0.2355700	sec
RG	181.26	
DW	115.000	usec
DE	10.00	usec
TE	298.0	K
D0	0.00000300	sec
D1	1.00000000	sec
D13	0.00000400	sec
D16	0.00020000	sec
IN0	0.00023000	sec
ND0	1	
TD	128	
SFO1	700.1819	MHz
FIDRES	33.967392	Hz
SW	6.210	ppm
FnMODE	QF	
SI	1024	
SF	700.1800165	MHz
WDW	SINE	
SSB	0	
LB	0.00	Hz
GB	0	
PC	1.40	
SI	1024	
MC2	QF	
SF	700.1800165	MHz
WDW	SINE	
SSB	0	
LB	0.00	Hz
GB	0	

^{1}H–^{1}H COSY (700 MHz) spectrum of the fragment **1b** in CD_3OD

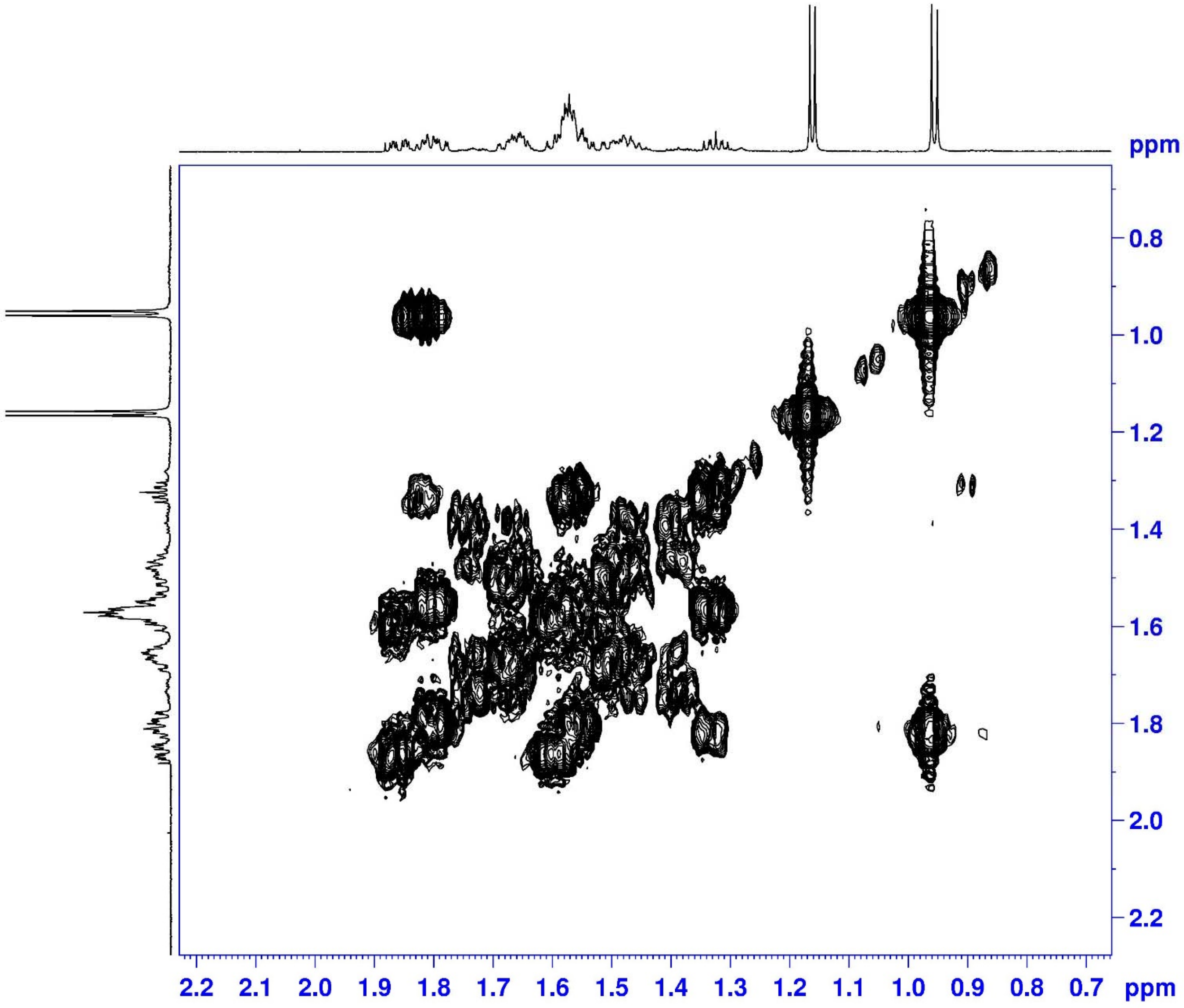

^{1}H–^{1}H COSY (700 MHz) spectrum of the fragment **1b** in CD_3OD

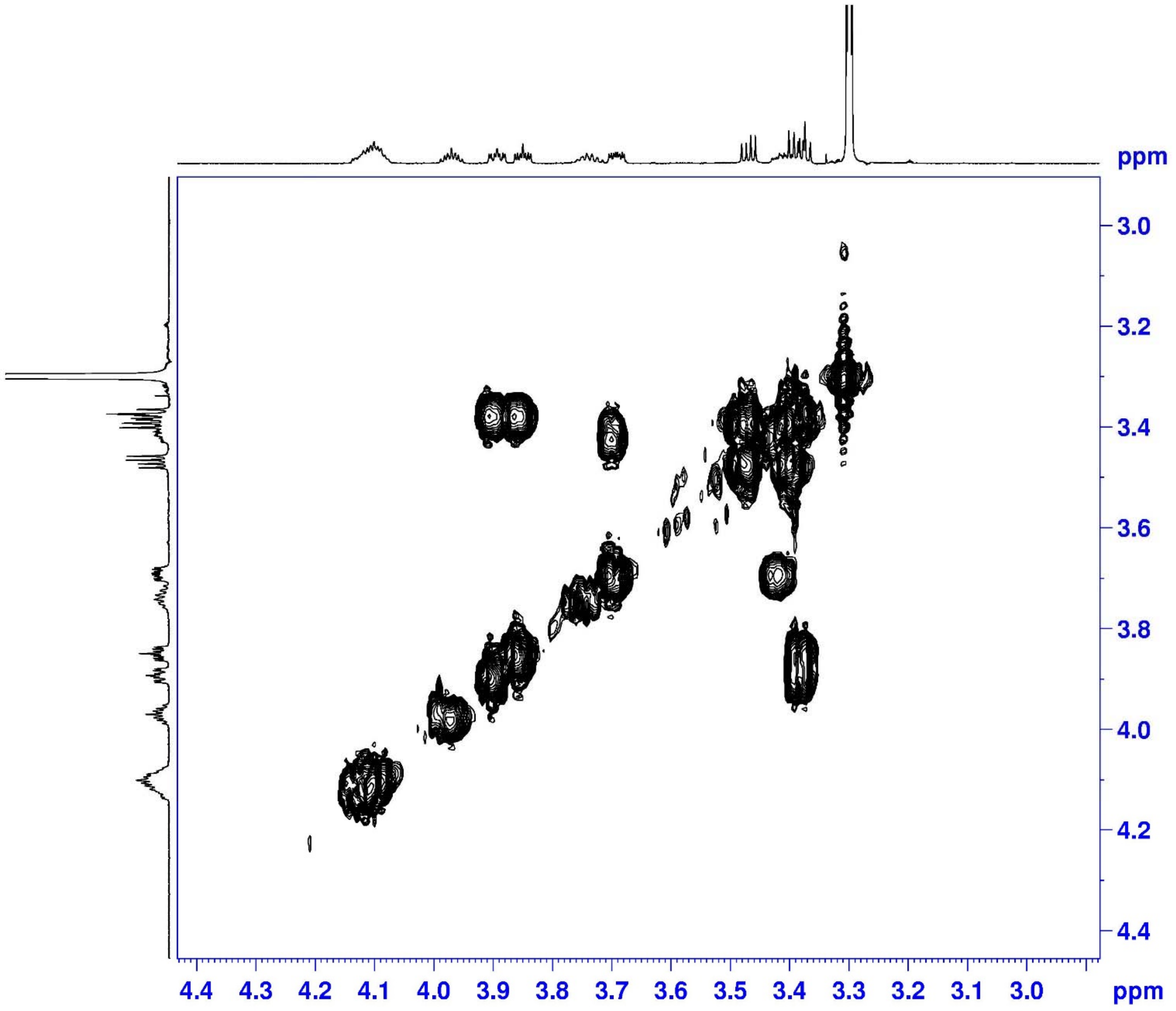

^1H–^1H COSY (700 MHz) spectrum of the fragment **1b** in CD_3OD

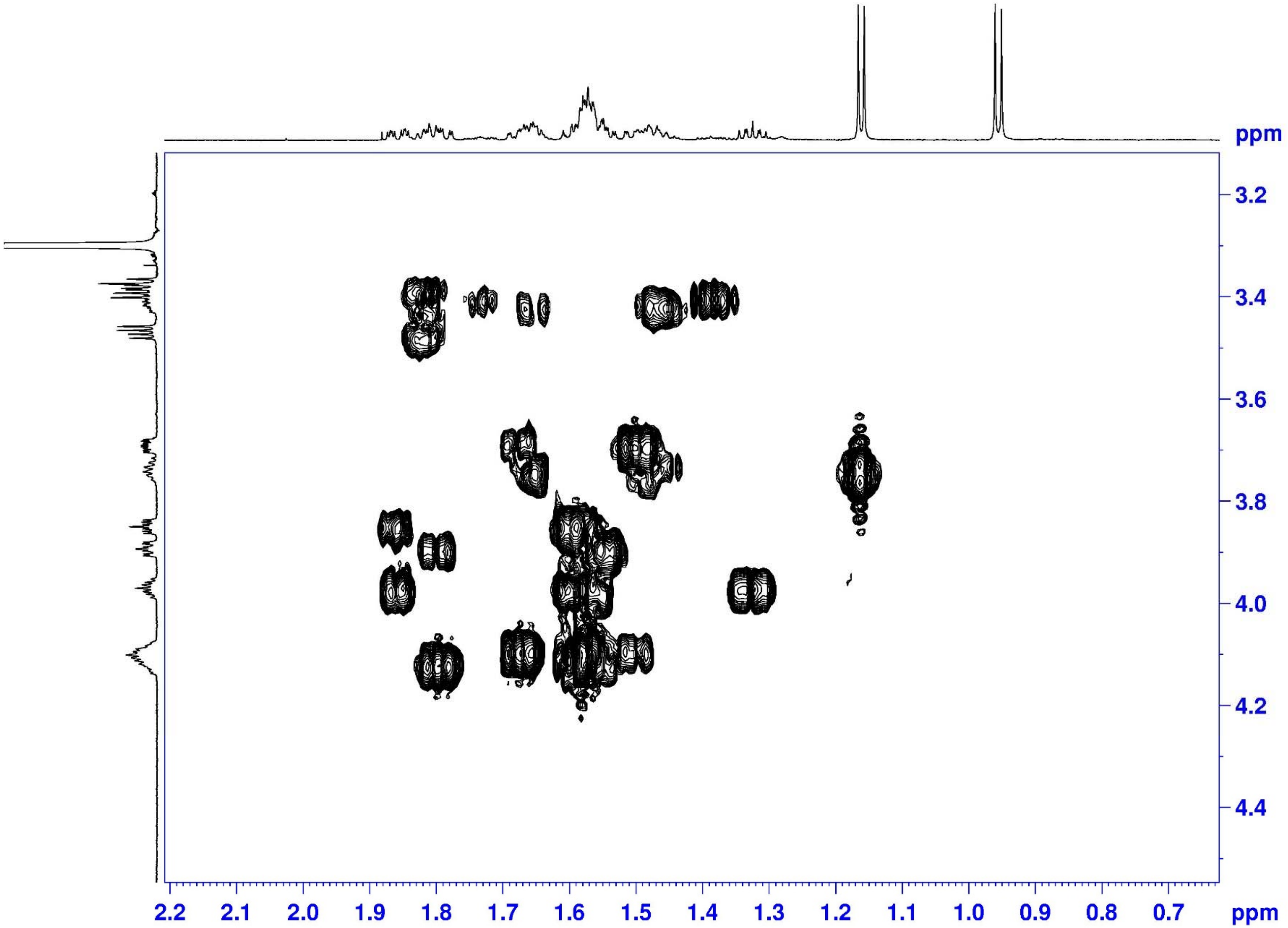

HSQC (700 MHz) spectrum of the fragment **1b** in CD_3OD

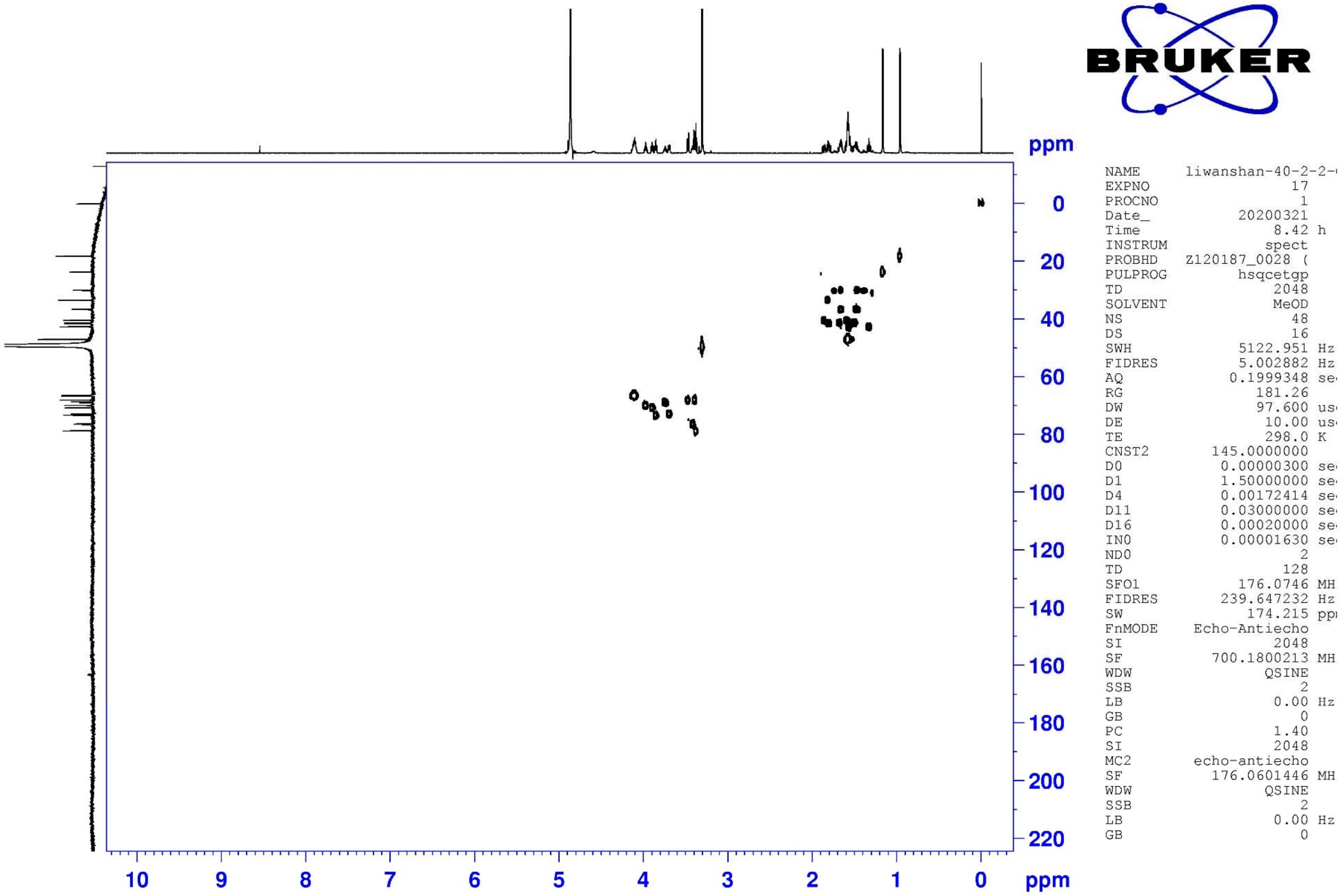

HSQC (700 MHz) spectrum of the fragment **1b** in CD_3OD

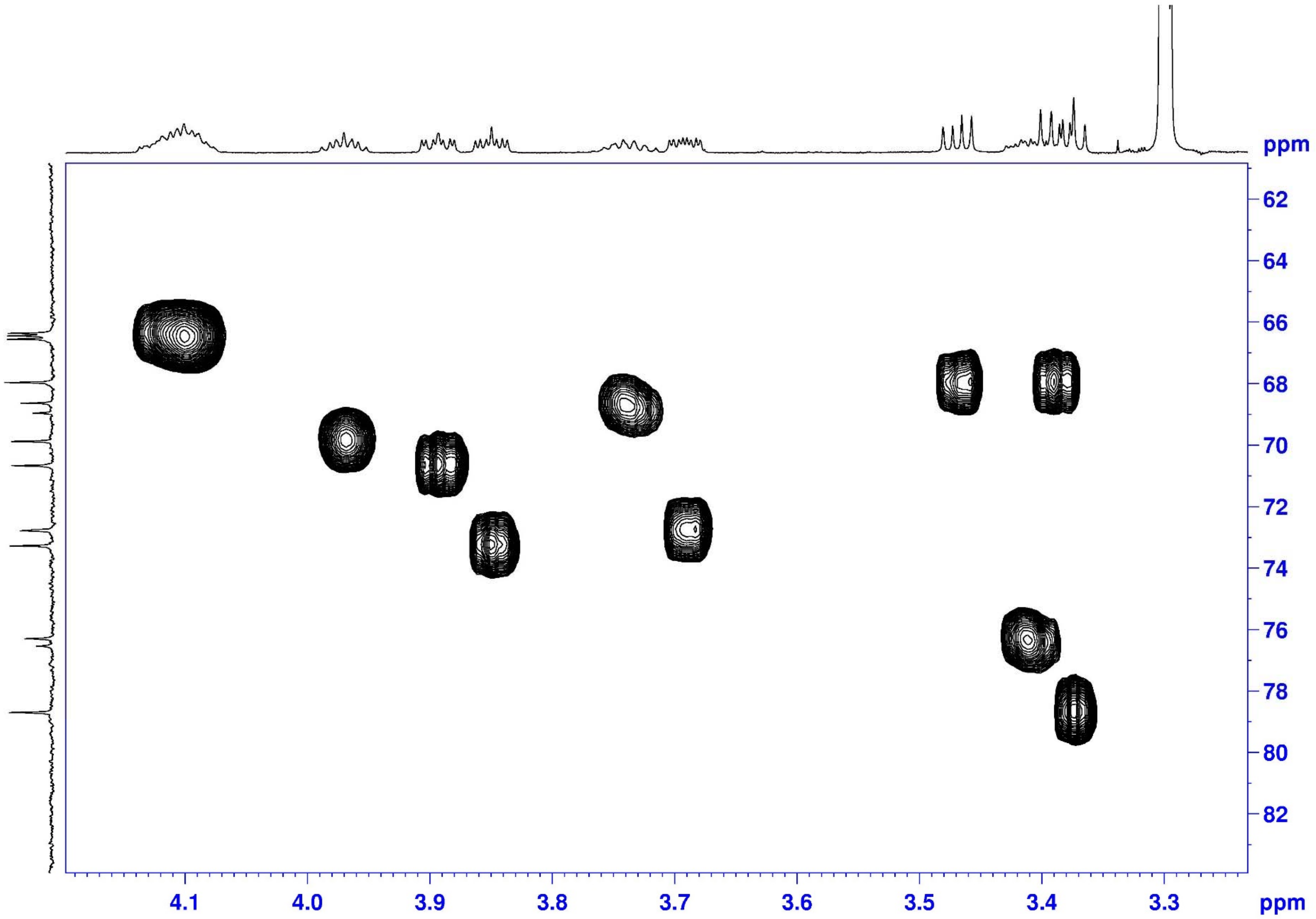

HSQC (700 MHz) spectrum of the fragment **1b** in CD_3OD

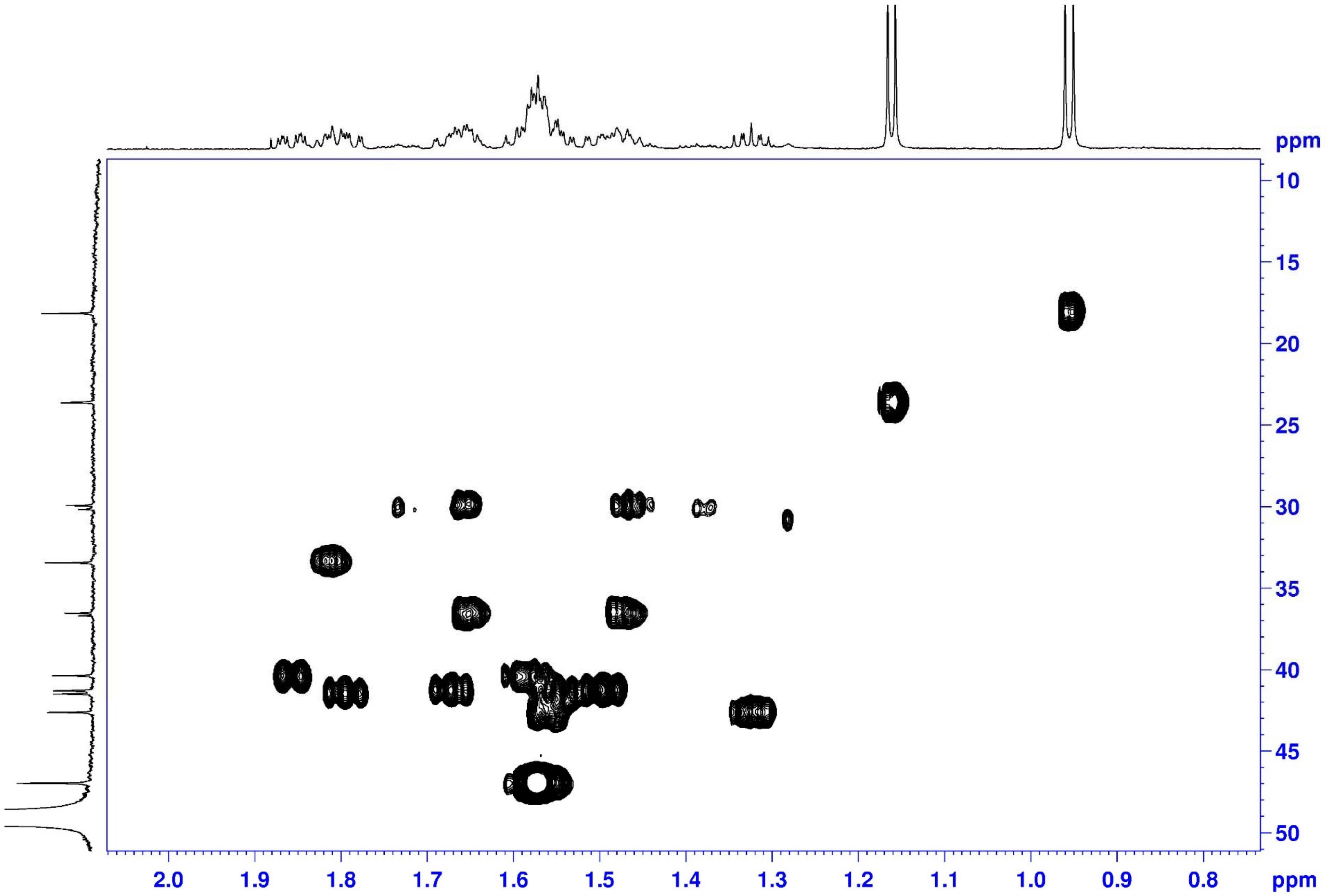

HMBC (700 MHz) spectrum of the fragment **1b** in CD_3OD

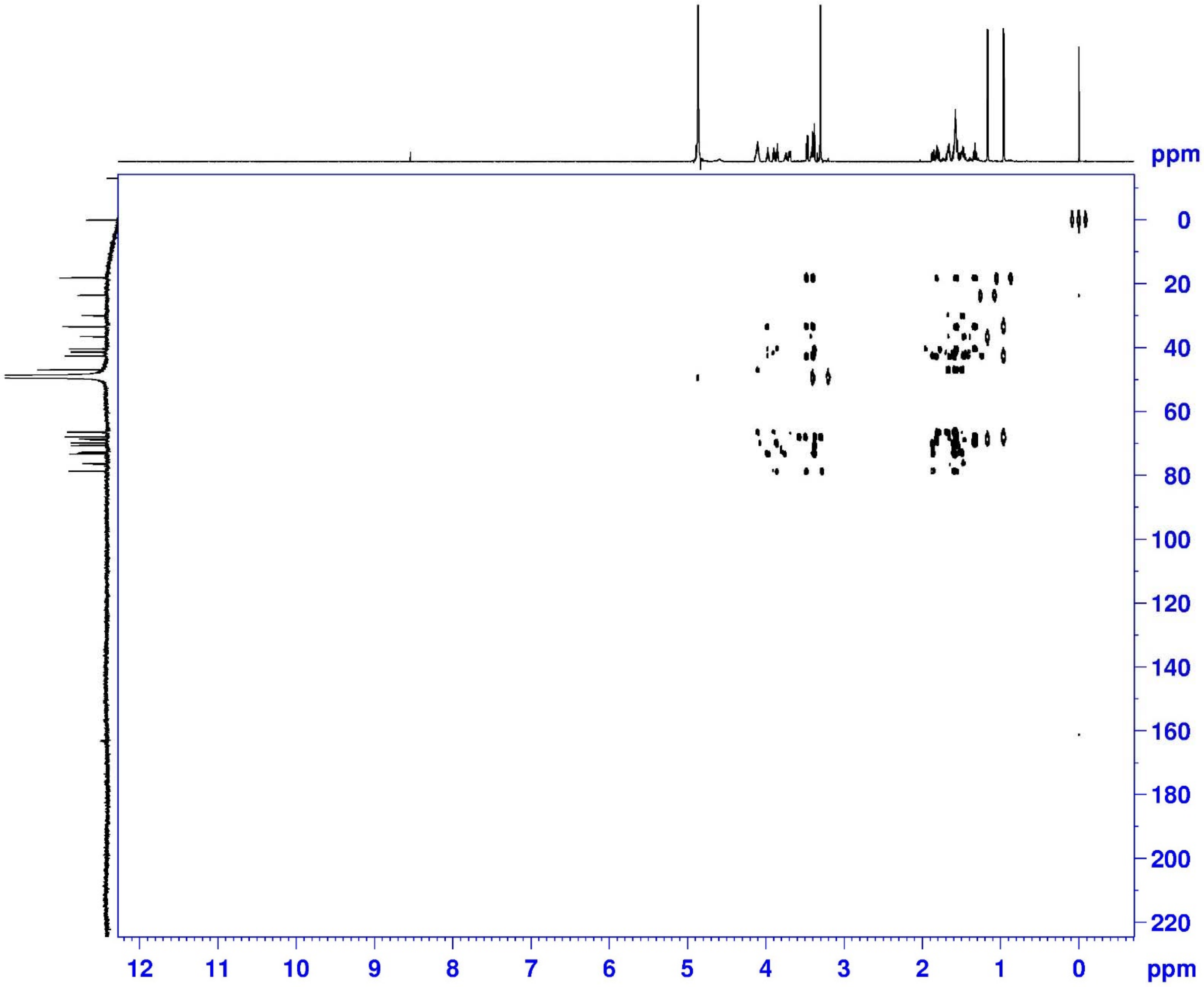

NAME	liwanshan-40-2-2
EXPNO	18
PROCNO	1
Date_	20200321
Time	11.39 h
INSTRUM	spect
PROBHD	Z120187_0028 (
PULPROG	hmbcgpndqf
TD	4096
SOLVENT	MeOD
NS	56
DS	16
SWH	4288.165 H
FIDRES	2.093830 H
AQ	0.4776436 s
RG	181.26
DW	116.600 u
DE	10.00 u
TE	298.0 K
CNST13	8.0000000
D0	0.00000300 s
D1	1.00000000 s
D6	0.06250000 s
D16	0.00020000 s
IN0	0.00001720 s
ND0	2
TD	128
SFO1	176.0741 M
FIDRES	227.107559 H
SW	165.100 p
FnMODE	QF
SI	4096
SF	700.1800250 M
WDW	SINE
SSB	0
LB	0.00 H
GB	0
PC	1.40
SI	2048
MC2	QF
SF	176.0601446 M
WDW	SINE
SSB	0
LB	0.00 H
GB	0

HMBC (700 MHz) spectrum of the fragment **1b** in CD_3OD

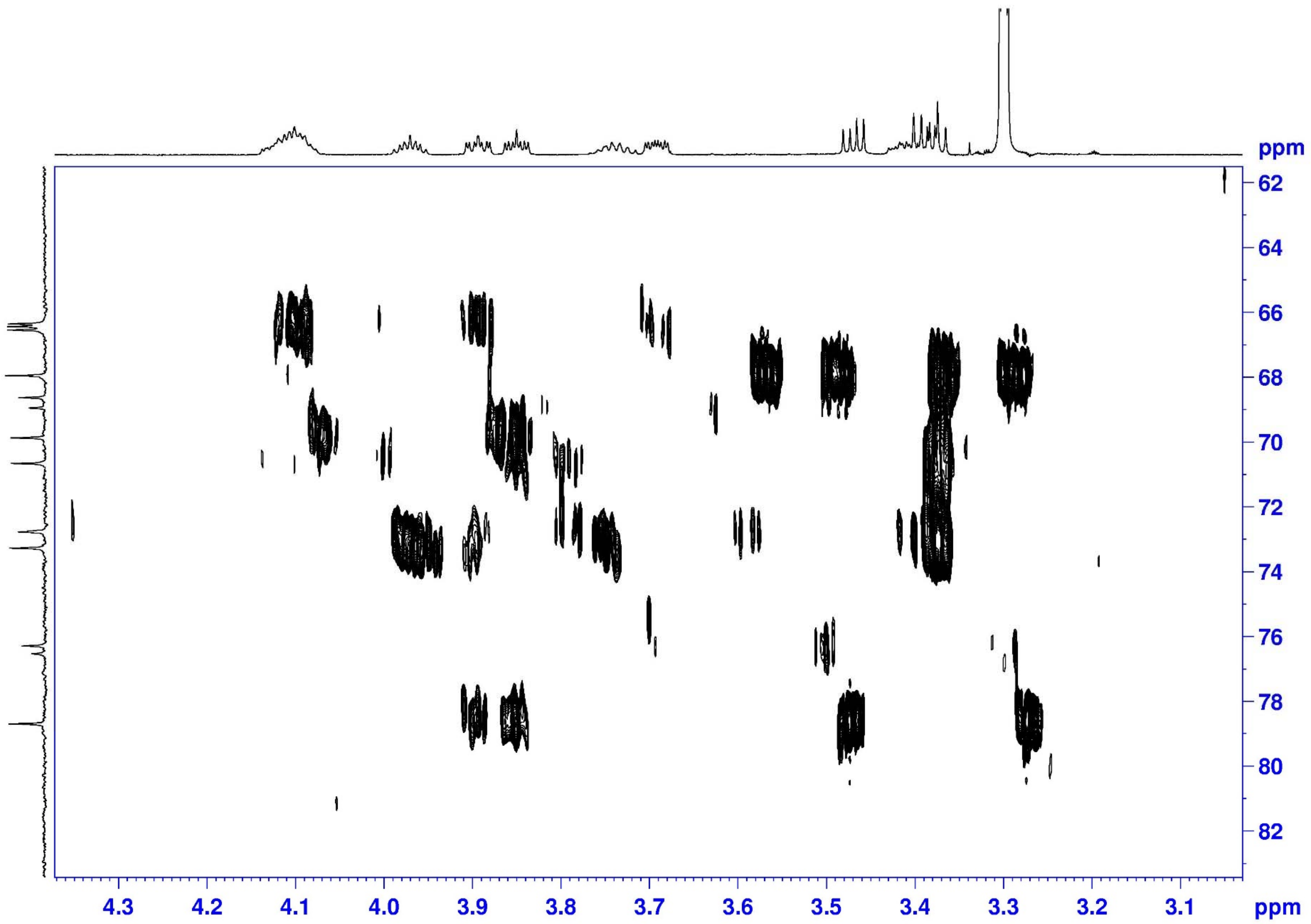

HMBC (700 MHz) spectrum of the fragment **1b** in CD_3OD

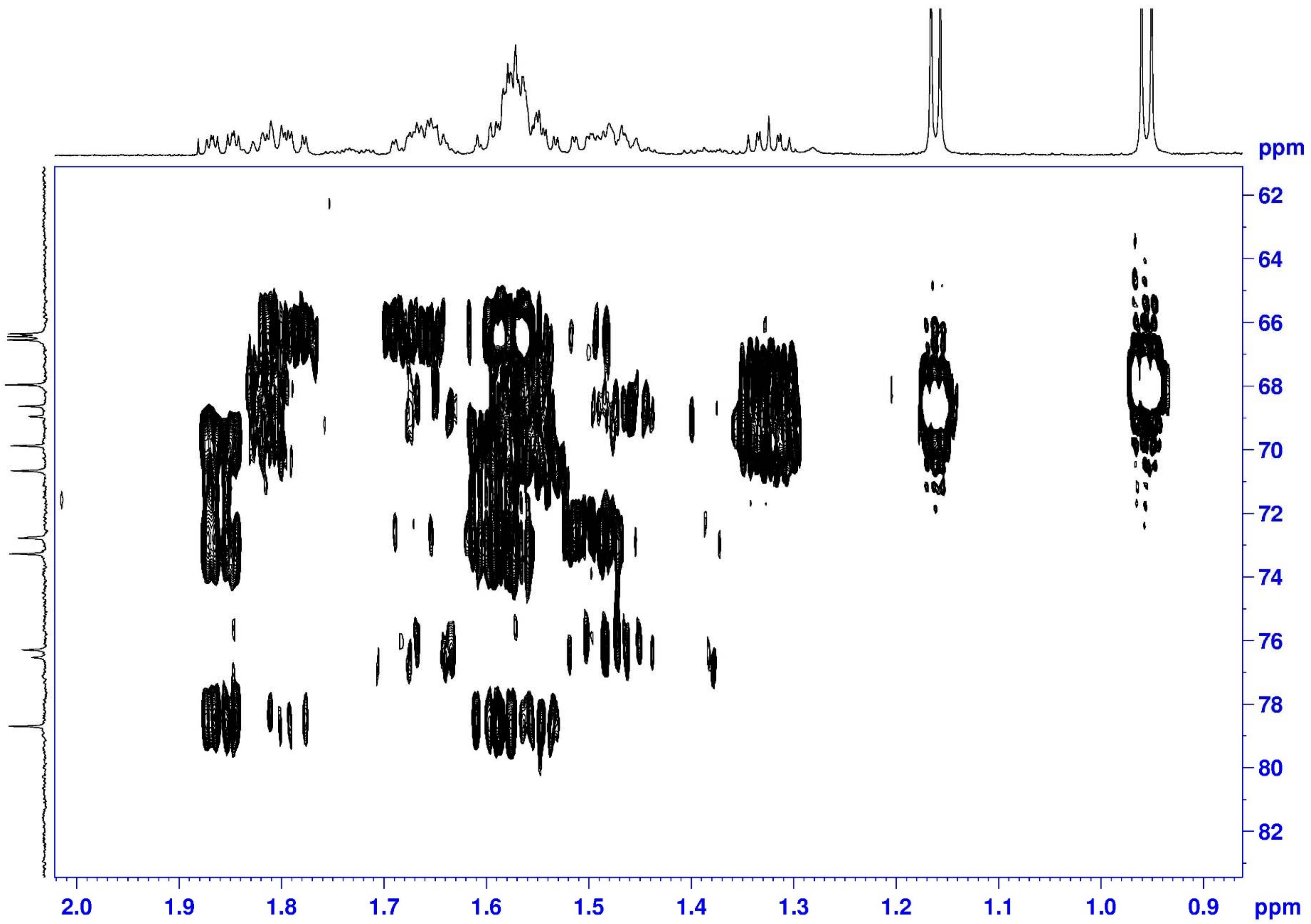

HMBC (700 MHz) spectrum of the fragment **1b** in CD_3OD

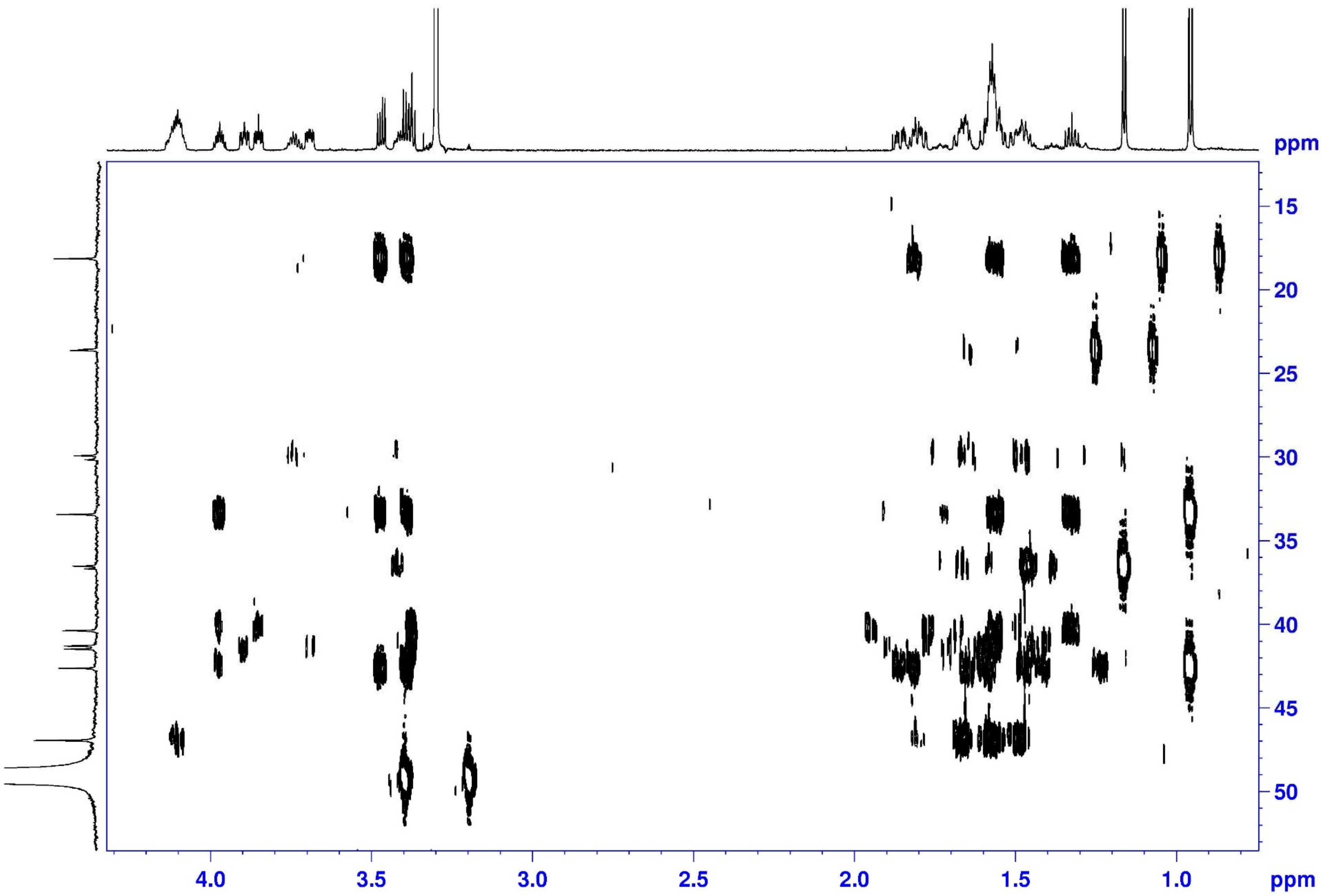

2D *J*RES (700 MHz) spectrum of the fragment **1b** in CD_3OD

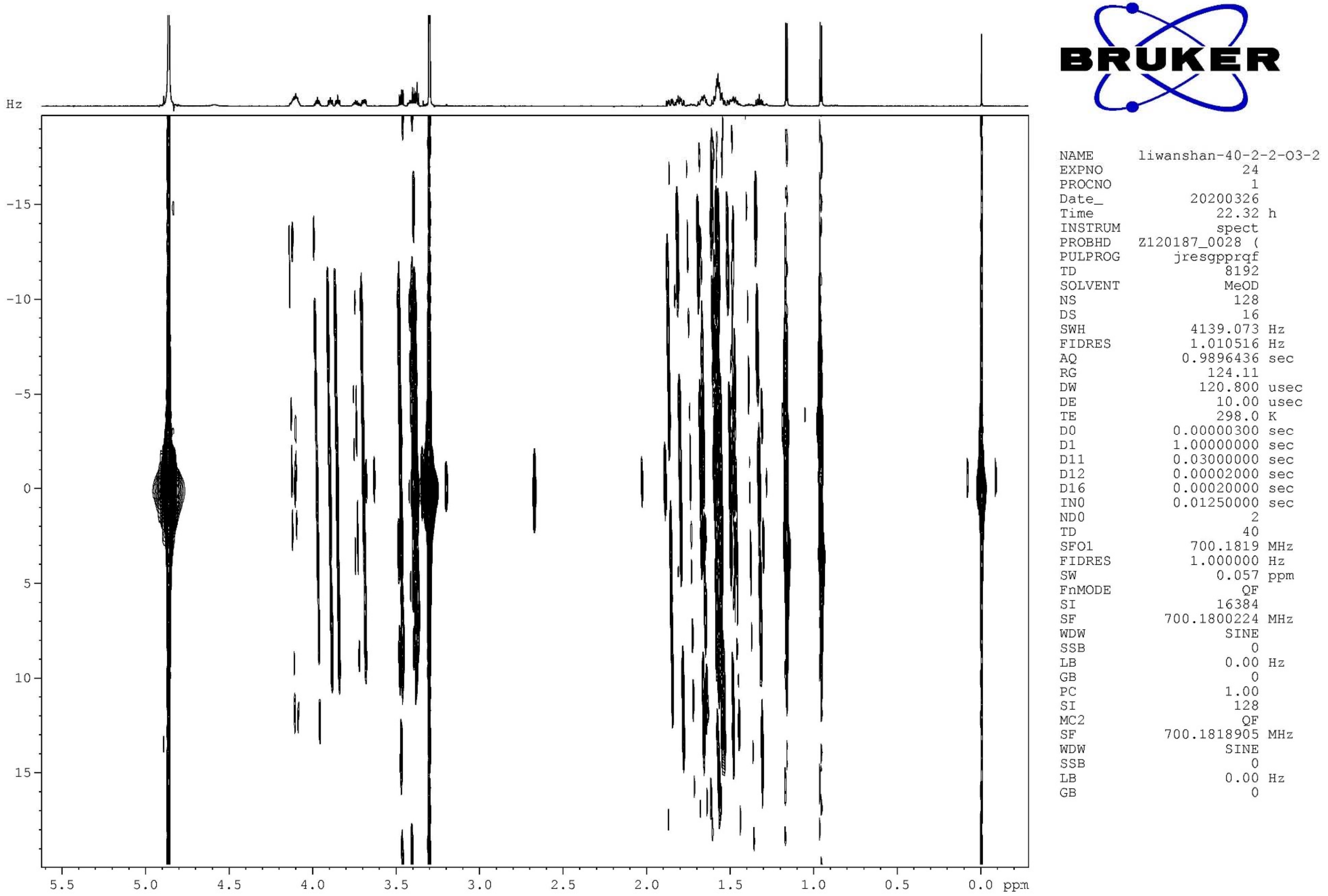

2D *J*RES (700 MHz) spectrum of the fragment **1b** in CD_3OD

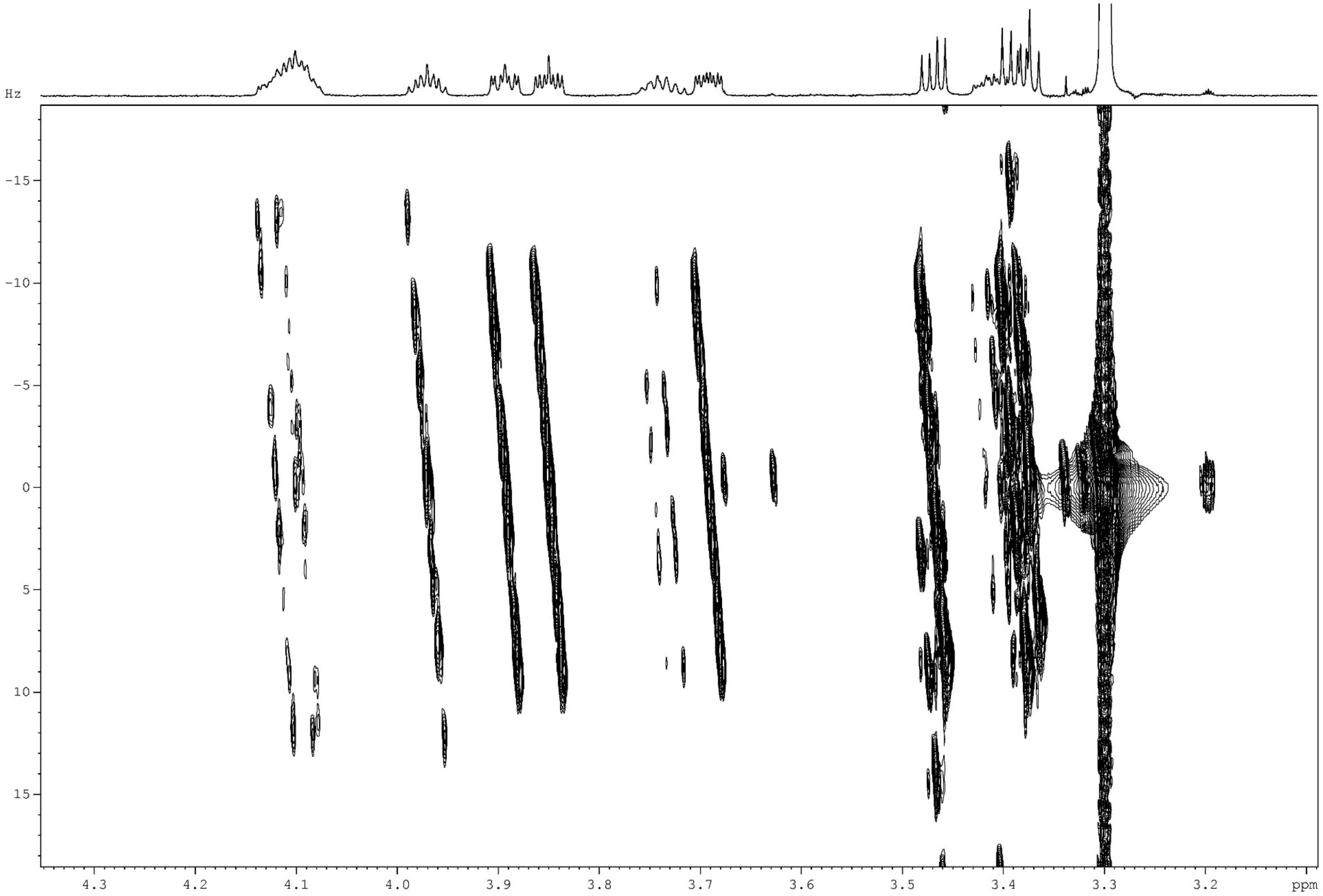

2D *J*RES (700 MHz) spectrum of the fragment **1b** in CD_3OD

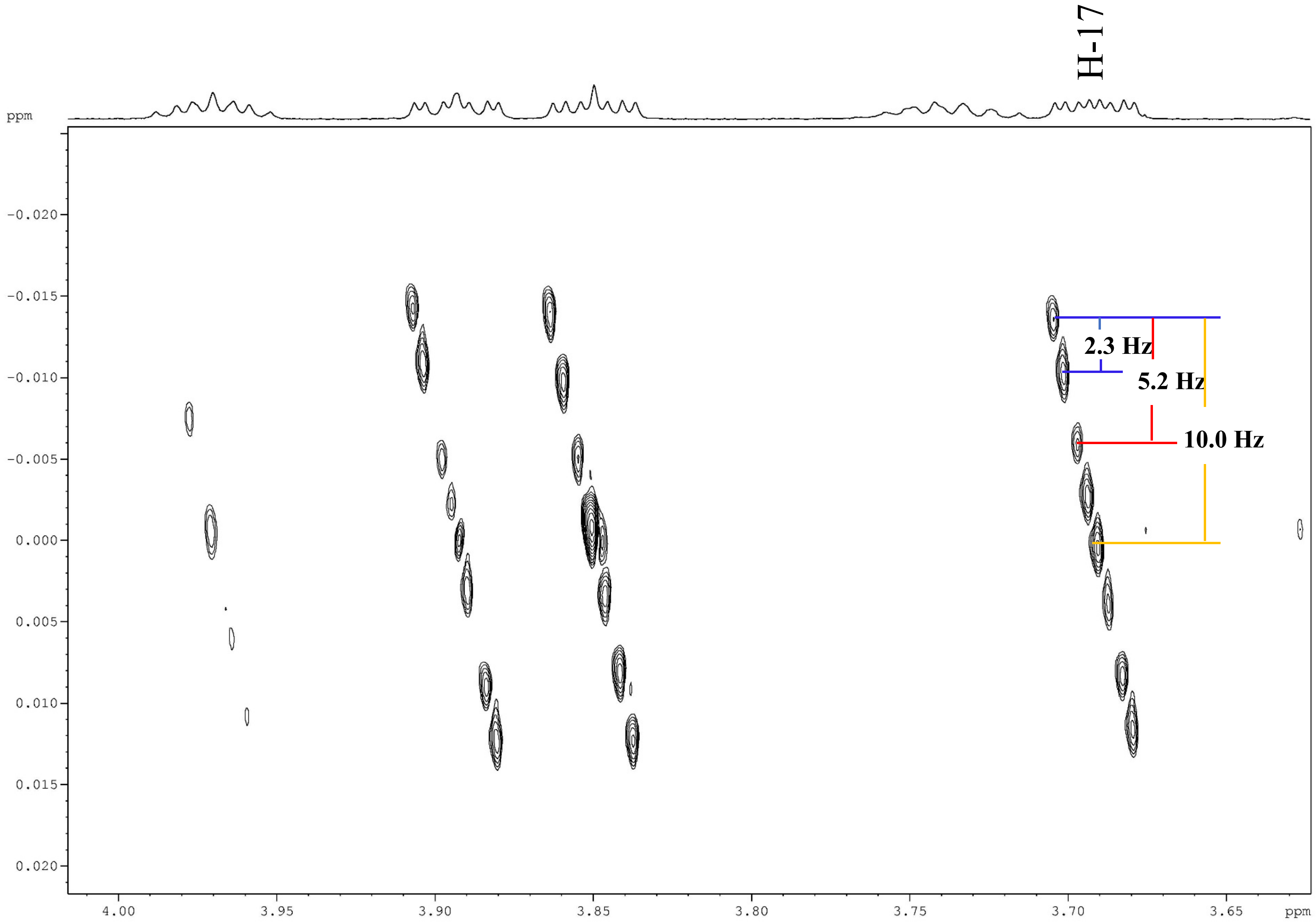

2D *J*RES (700 MHz) spectrum of the fragment **1b** in CD_3OD

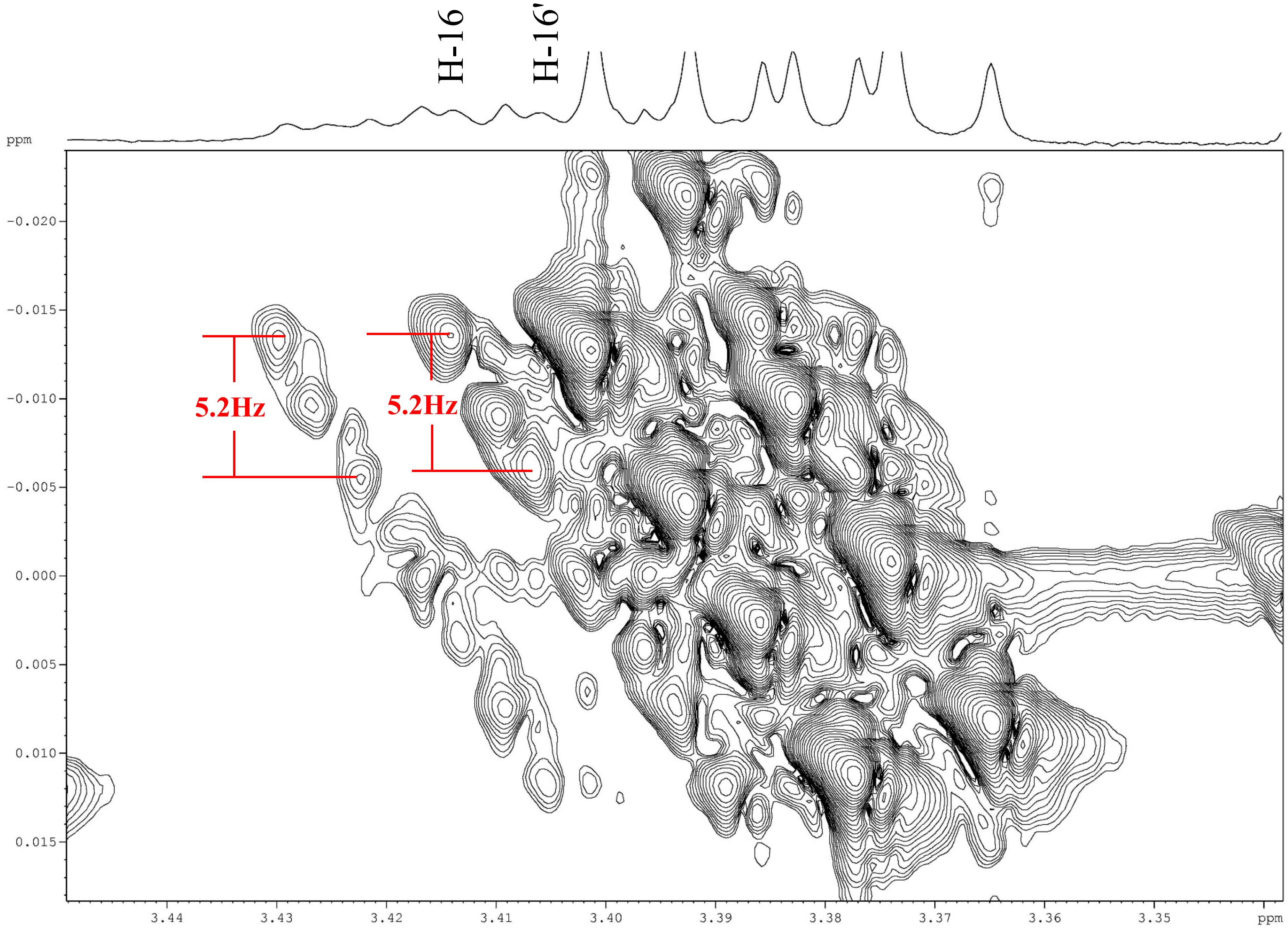

2D *J*RES (700 MHz) spectrum of the fragment **1b** in CD_3OD

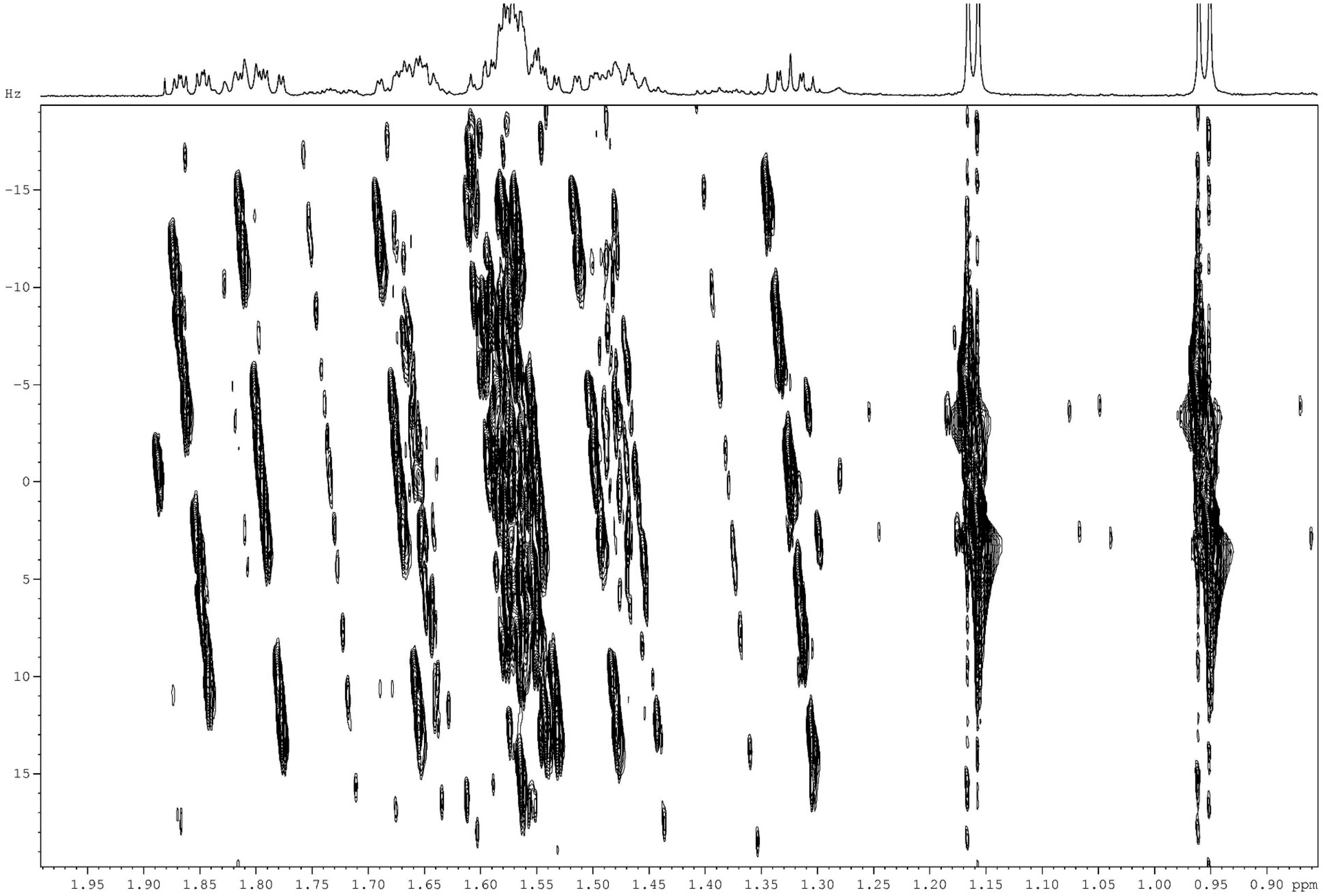

2D *J*RES (700 MHz) spectrum of the fragment **1b** in CD_3OD

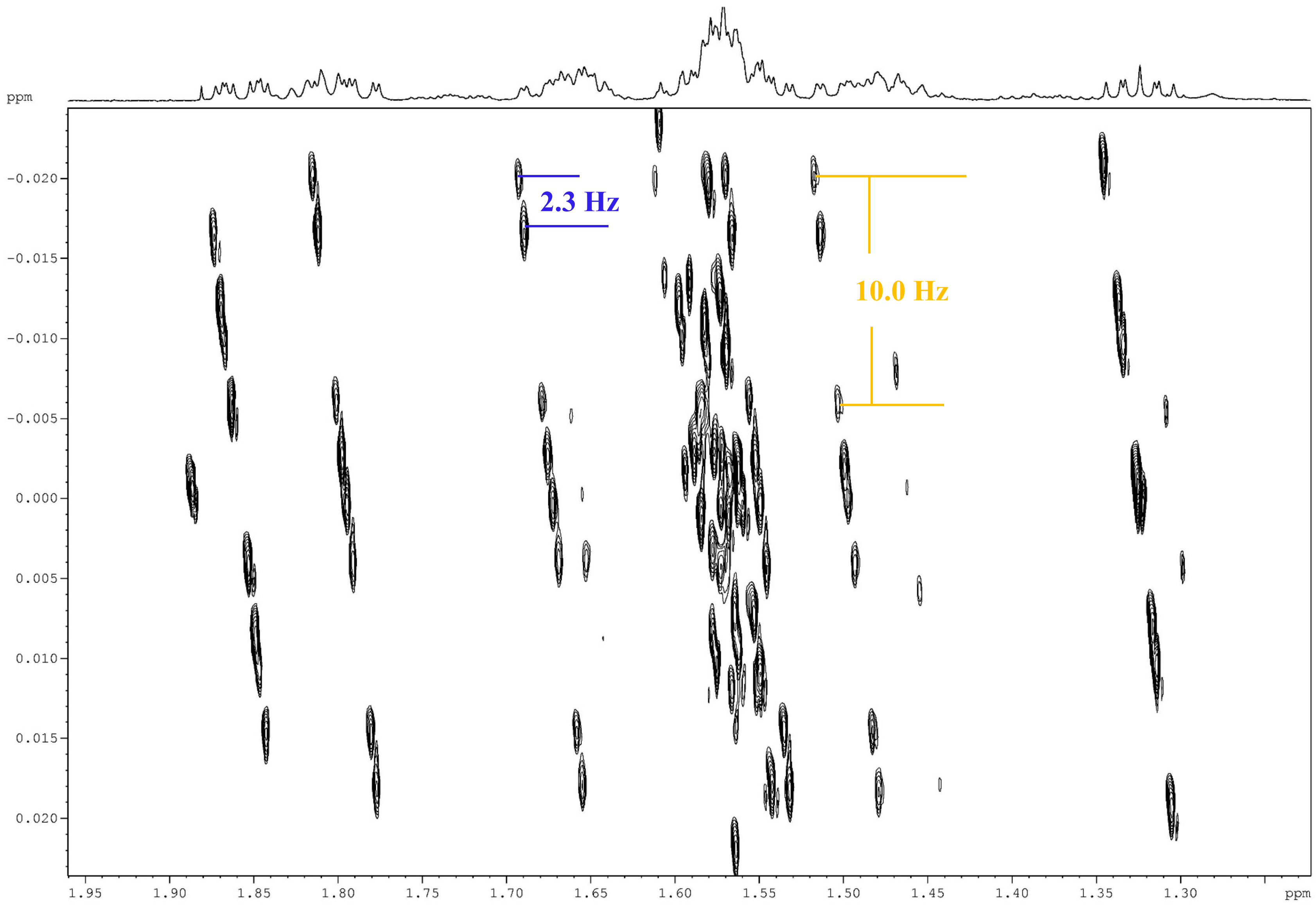

HECADE (700 MHz) spectrum of the fragment **1b** in CD_3OD

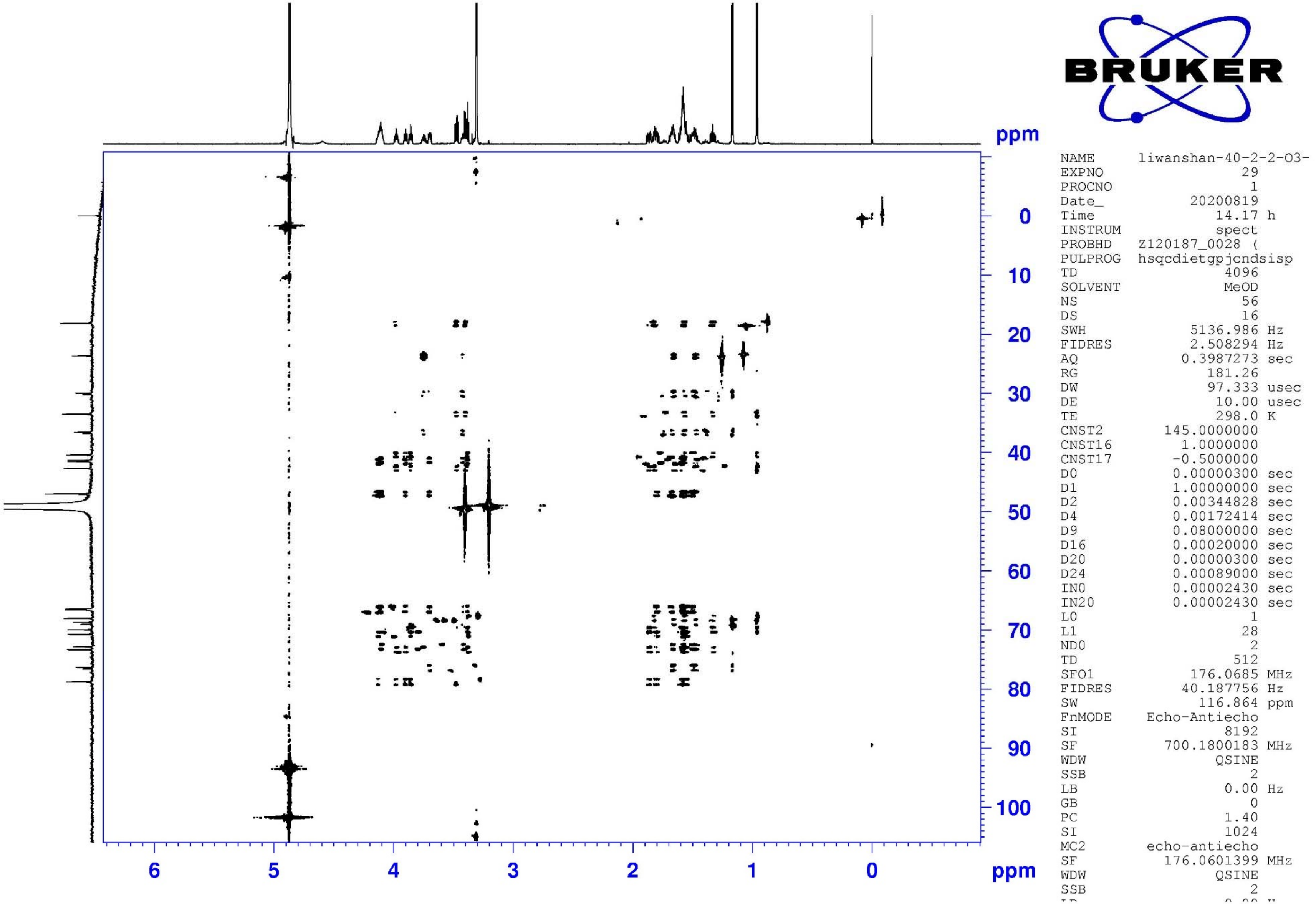

HECADE (700 MHz) spectrum of the fragment **1b** in CD_3OD

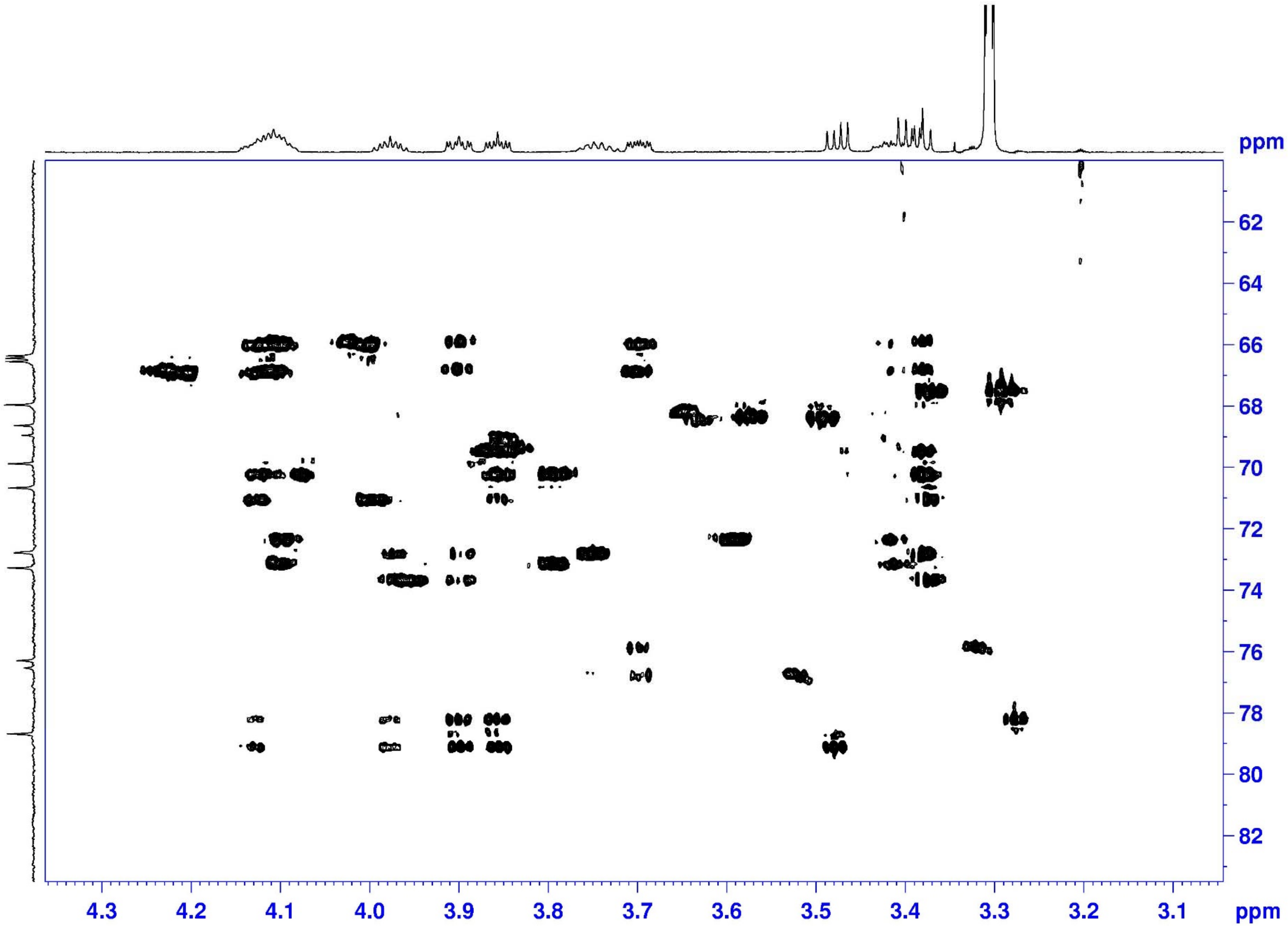

HECADE (700 MHz) spectrum of the fragment **1b** in CD_3OD

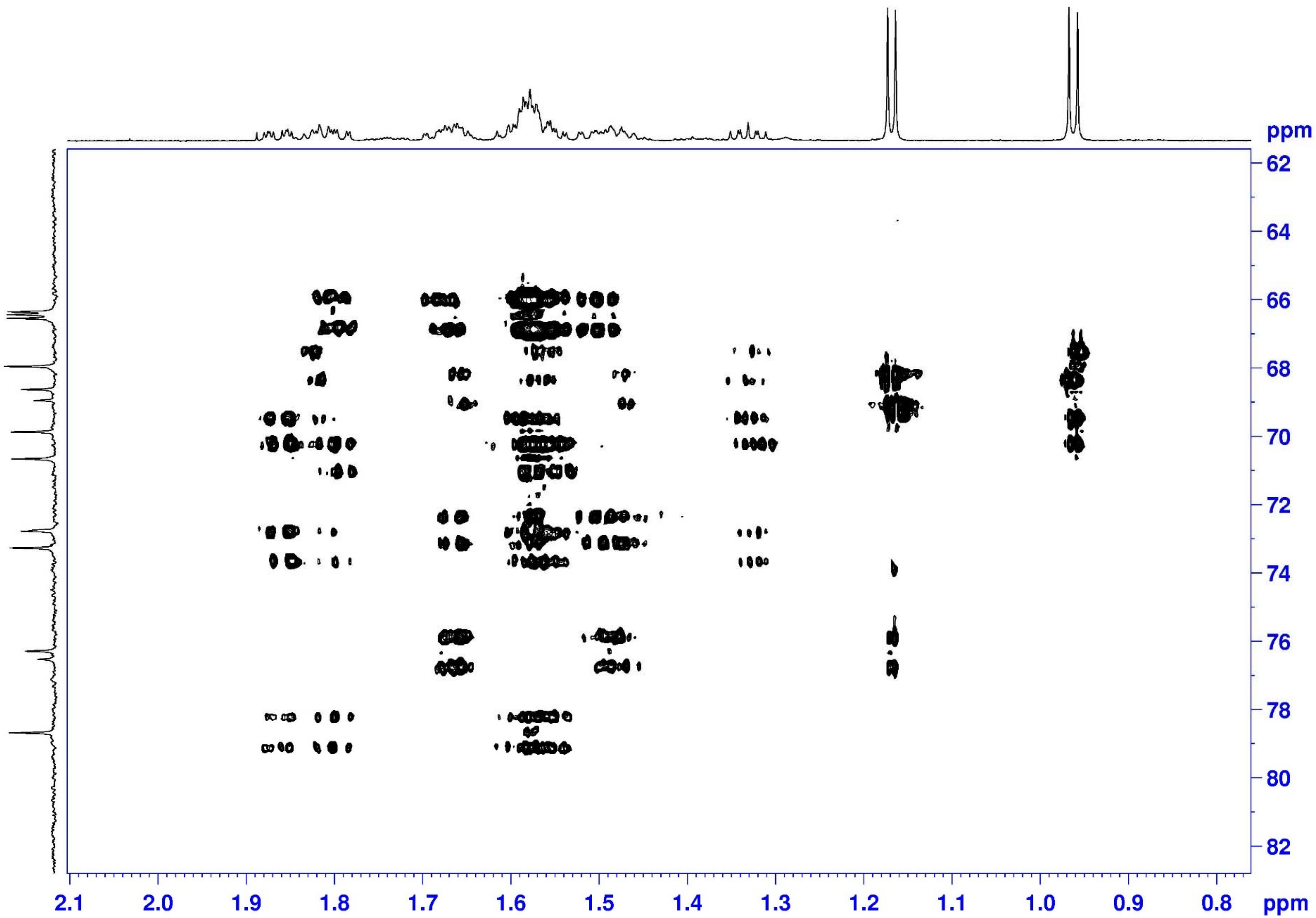

HECADE (700 MHz) spectrum of the fragment **1b** in CD_3OD

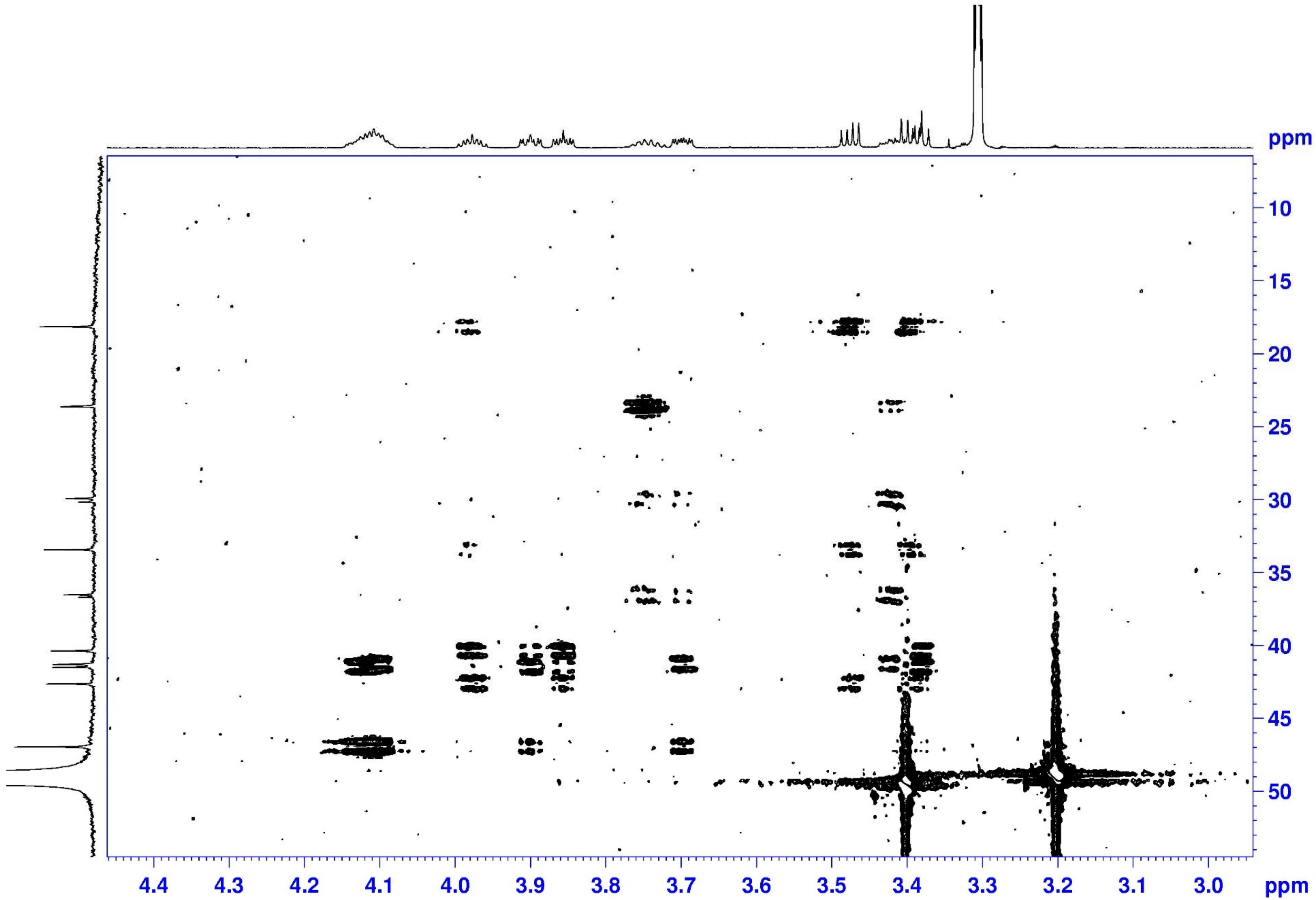

HECADE (700 MHz) spectrum of the fragment **1b** in CD_3OD

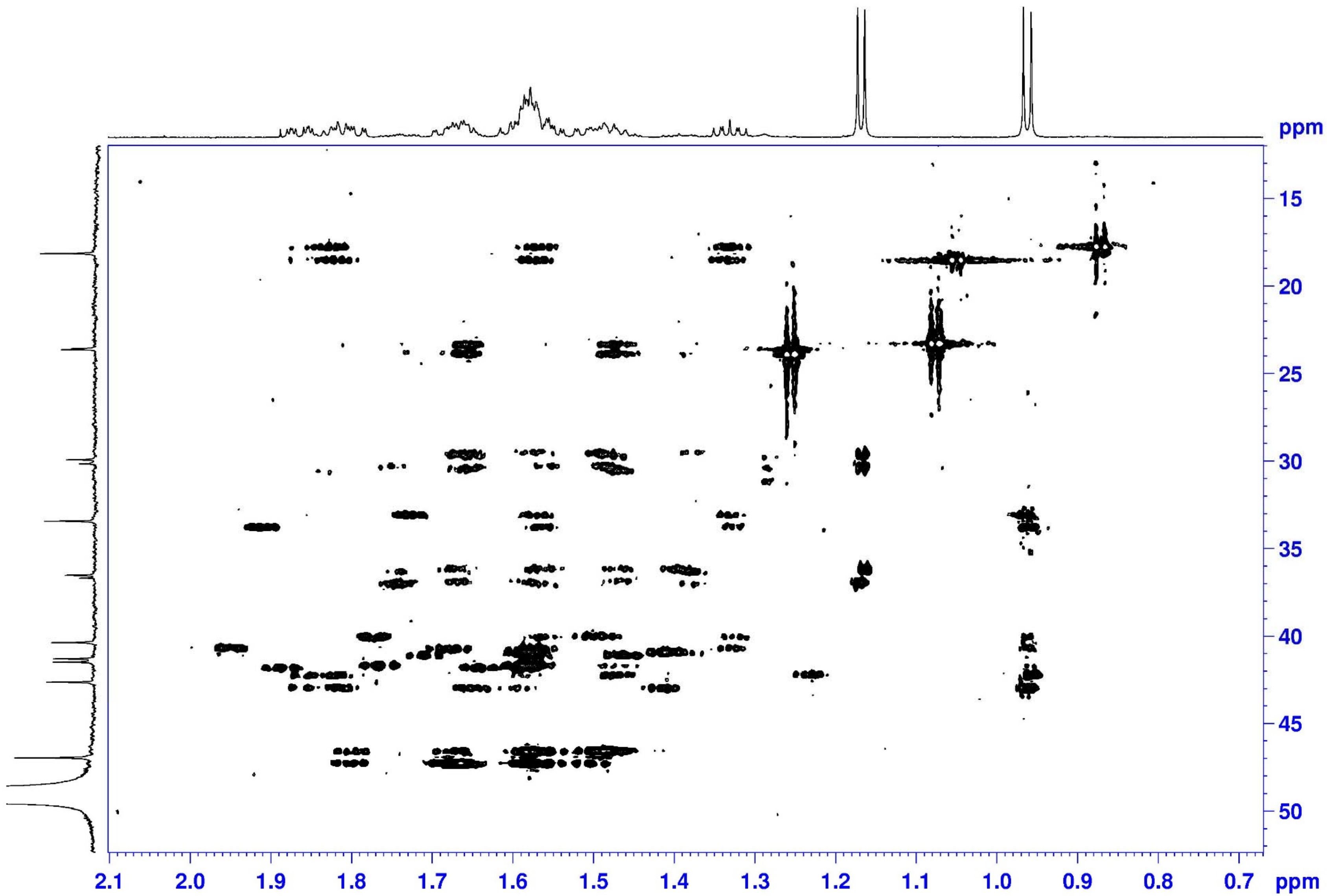

HECADE (700 MHz) spectrum of the fragment **1b** in CD_3OD

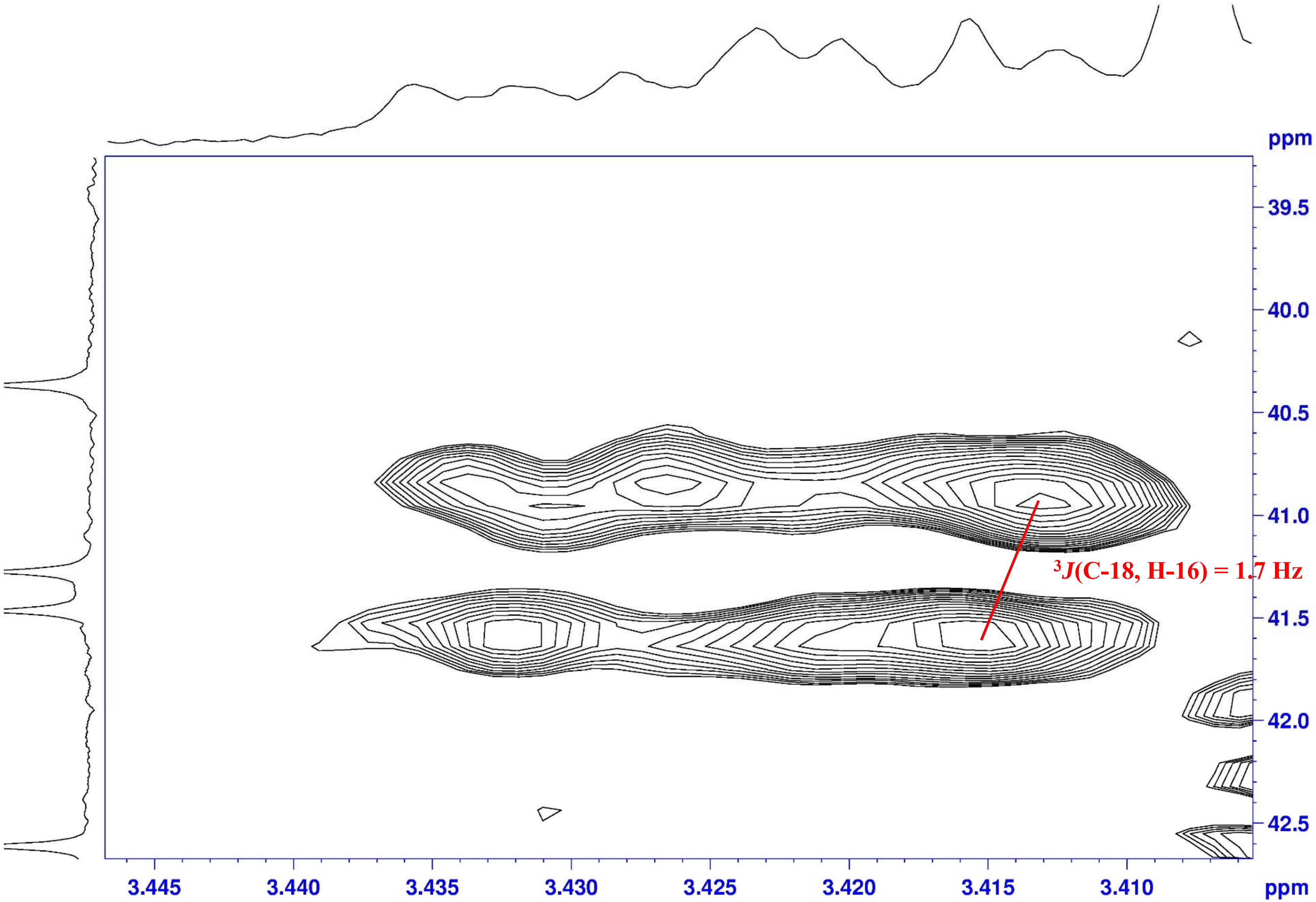

HECADE (700 MHz) spectrum of the fragment **1b** in CD_3OD

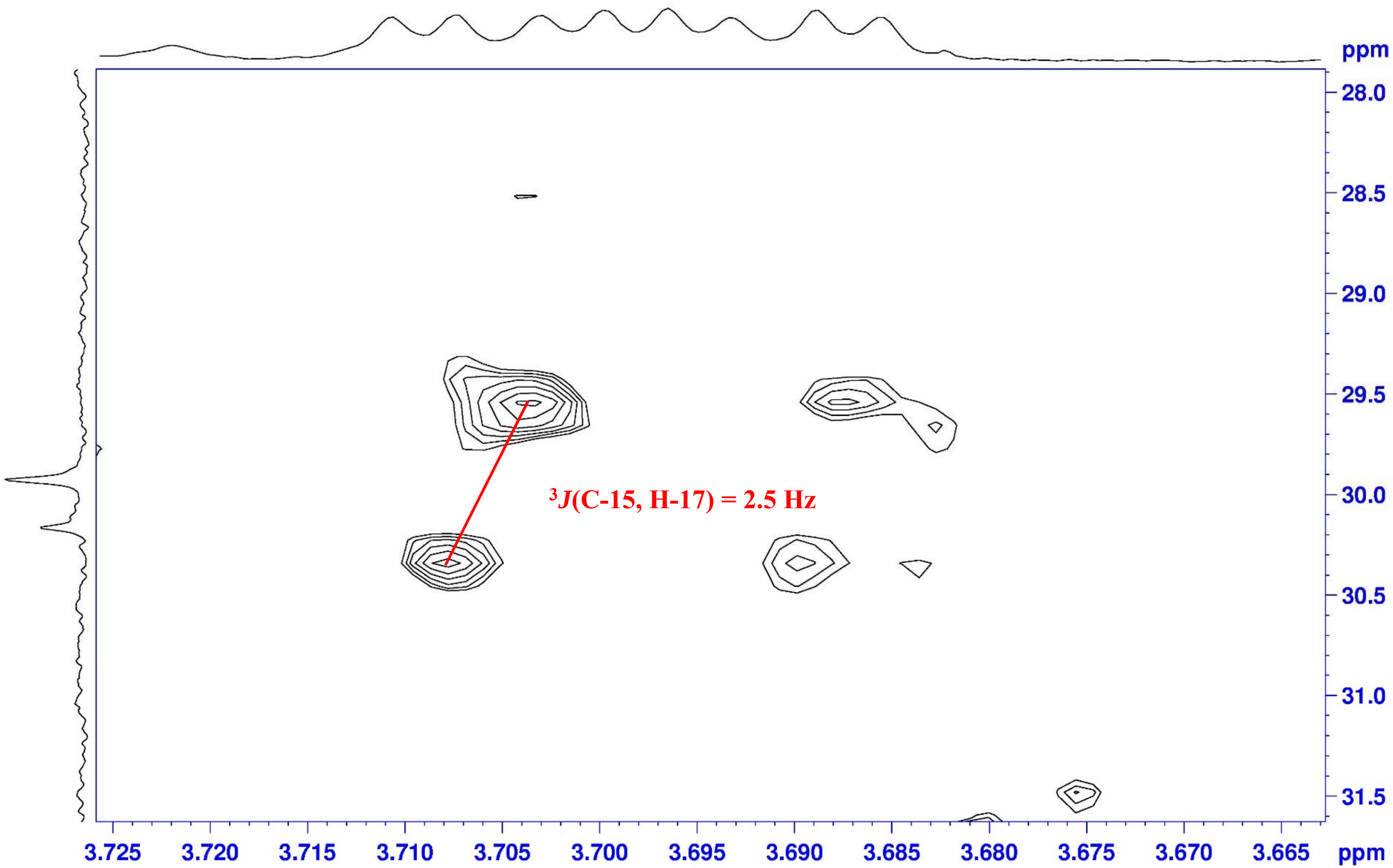

HECADE (700 MHz) spectrum of the fragment **1b** in CD_3OD

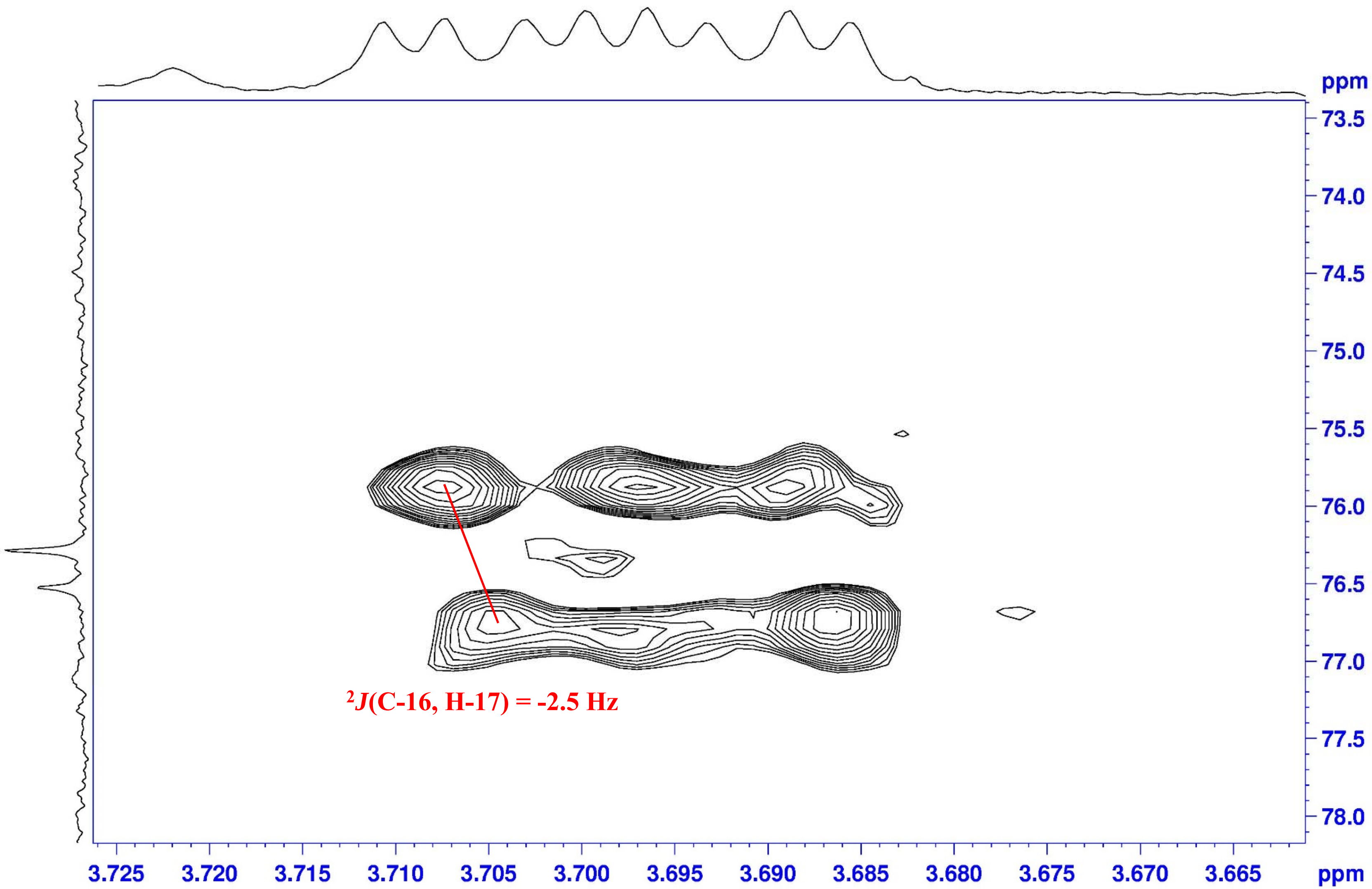

HECADE (700 MHz) spectrum of the fragment **1b** in CD_3OD

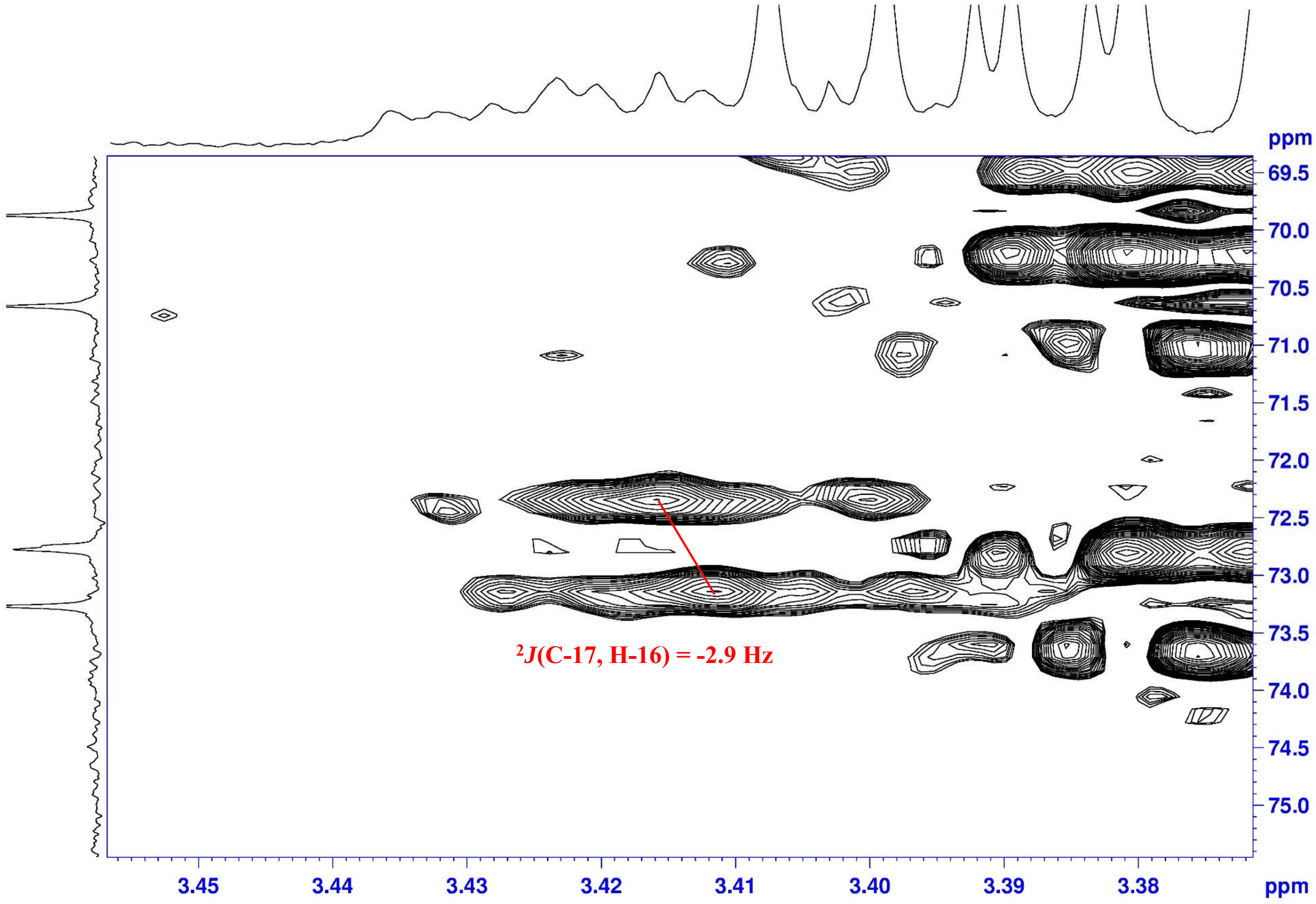

HETLOC (700 MHz) spectrum of the fragment **1b** in CD_3OD

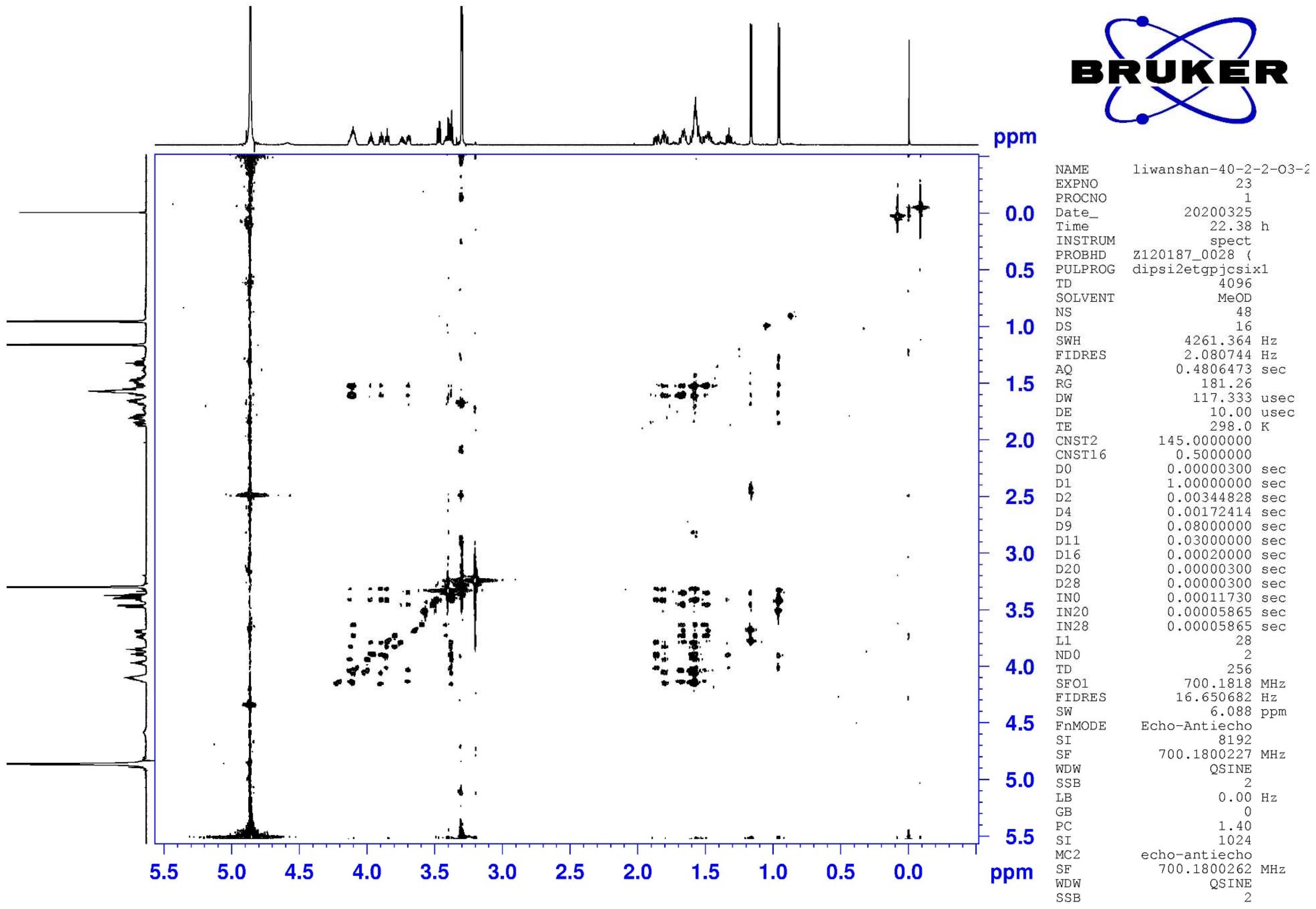

HETLOC (700 MHz) spectrum of the fragment **1b** in CD_3OD

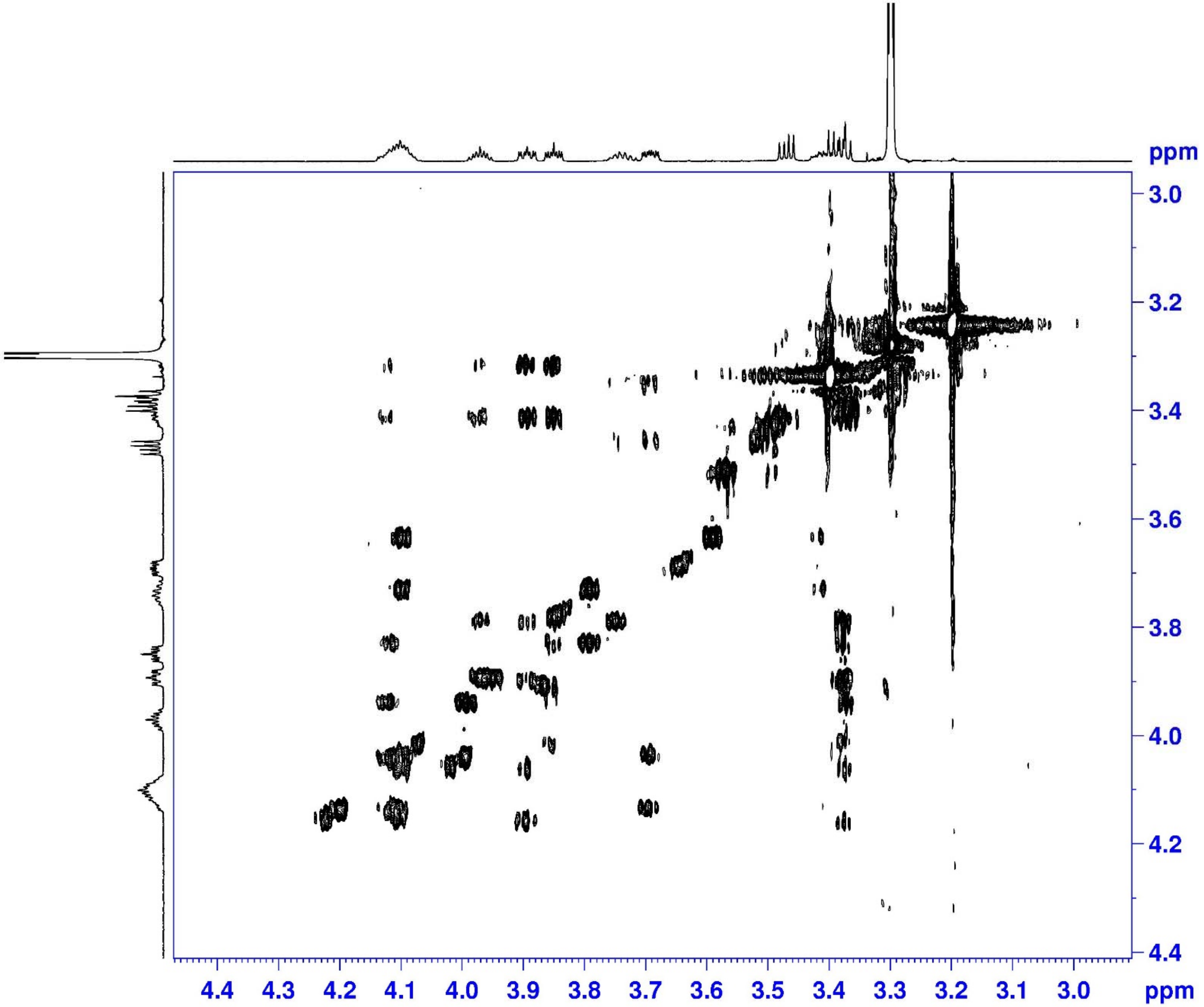

HETLOC (700 MHz) spectrum of the fragment **1b** in CD_3OD

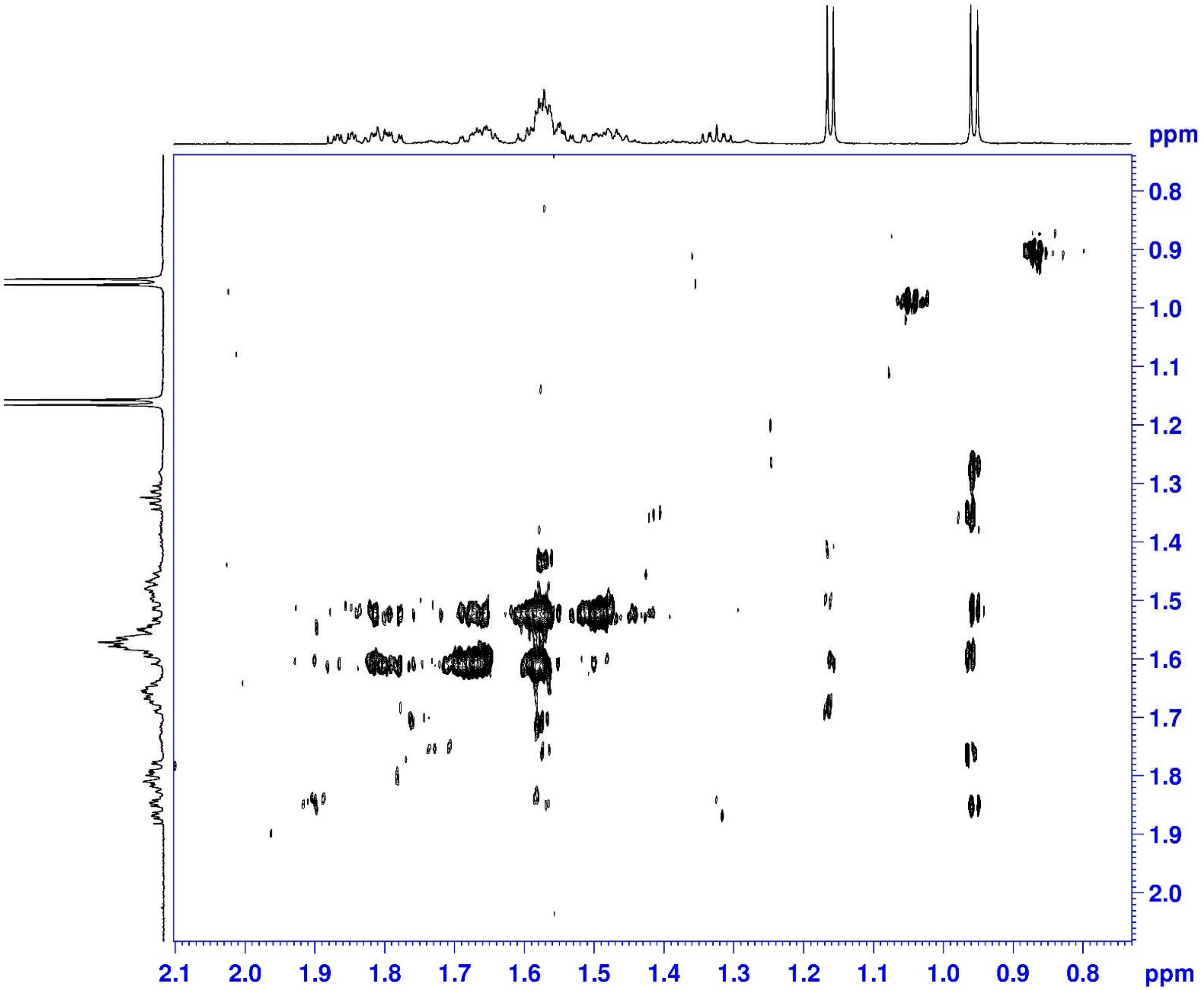

HETLOC (700 MHz) spectrum of the fragment **1b** in CD_3OD

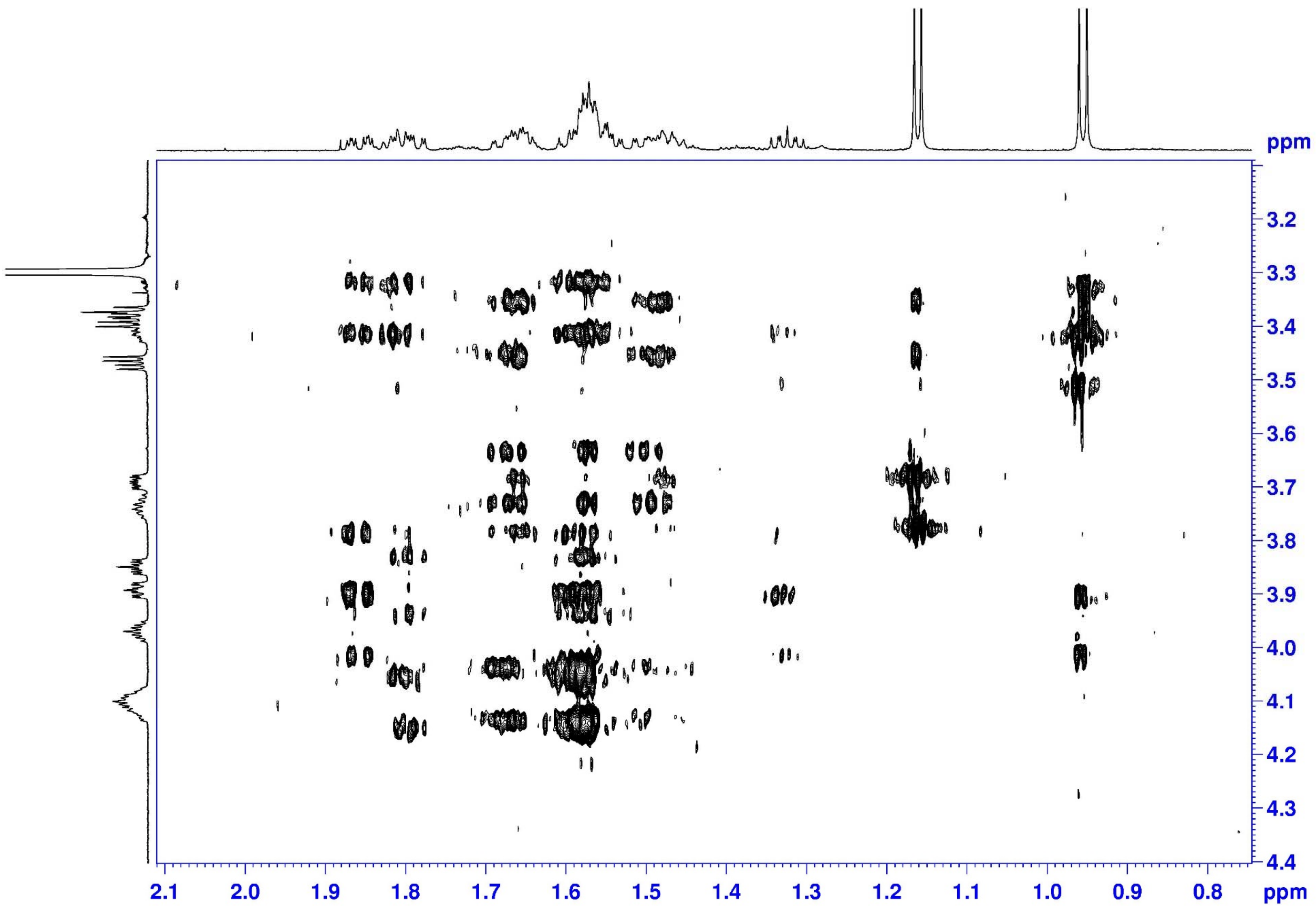

HETLOC (700 MHz) spectrum of the fragment **1b** in CD_3OD

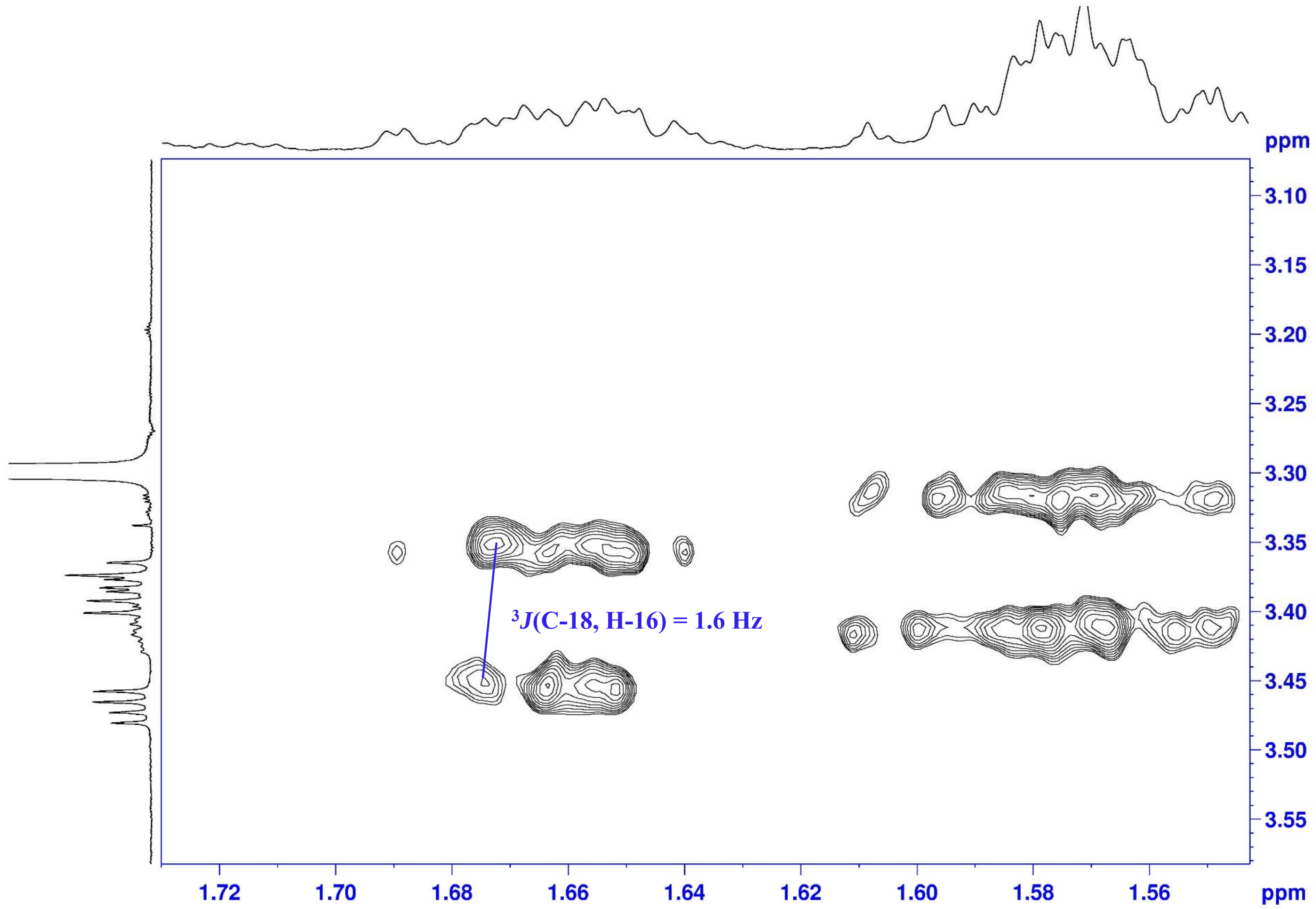

HETLOC (700 MHz) spectrum of the fragment **1b** in CD_3OD

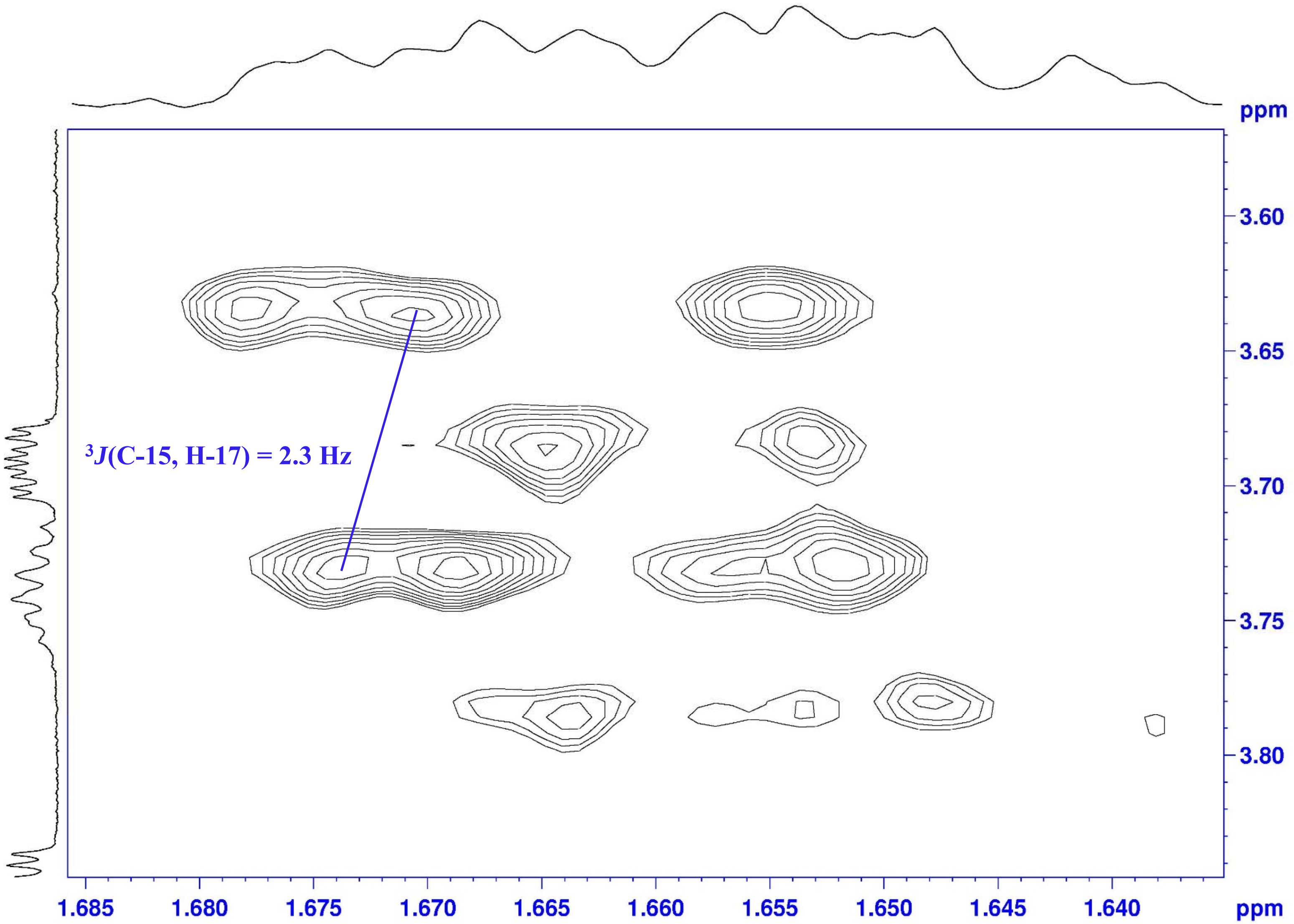

HETLOC (700 MHz) spectrum of the fragment **1b** in CD_3OD

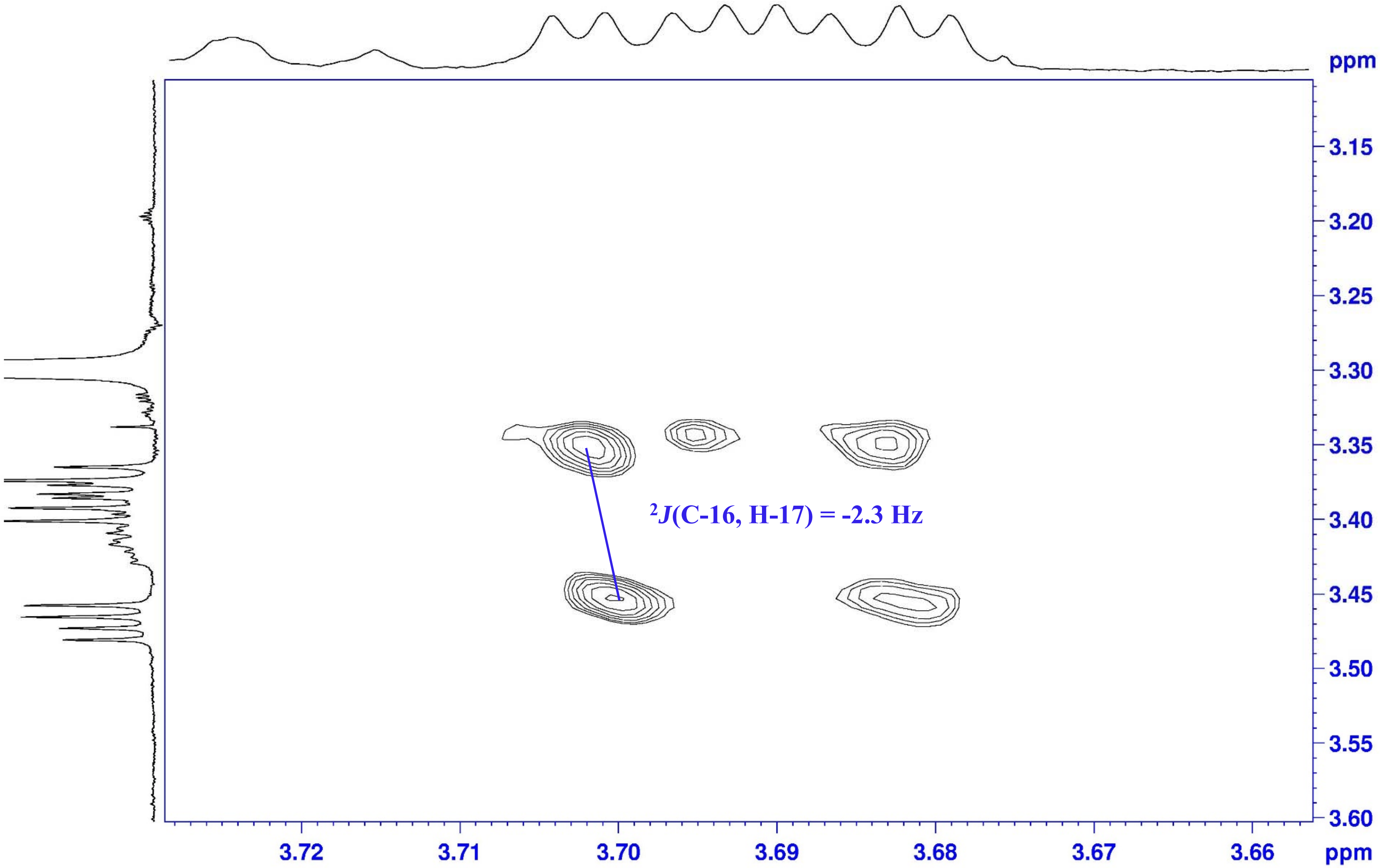

HETLOC (700 MHz) spectrum of the fragment **1b** in CD_3OD

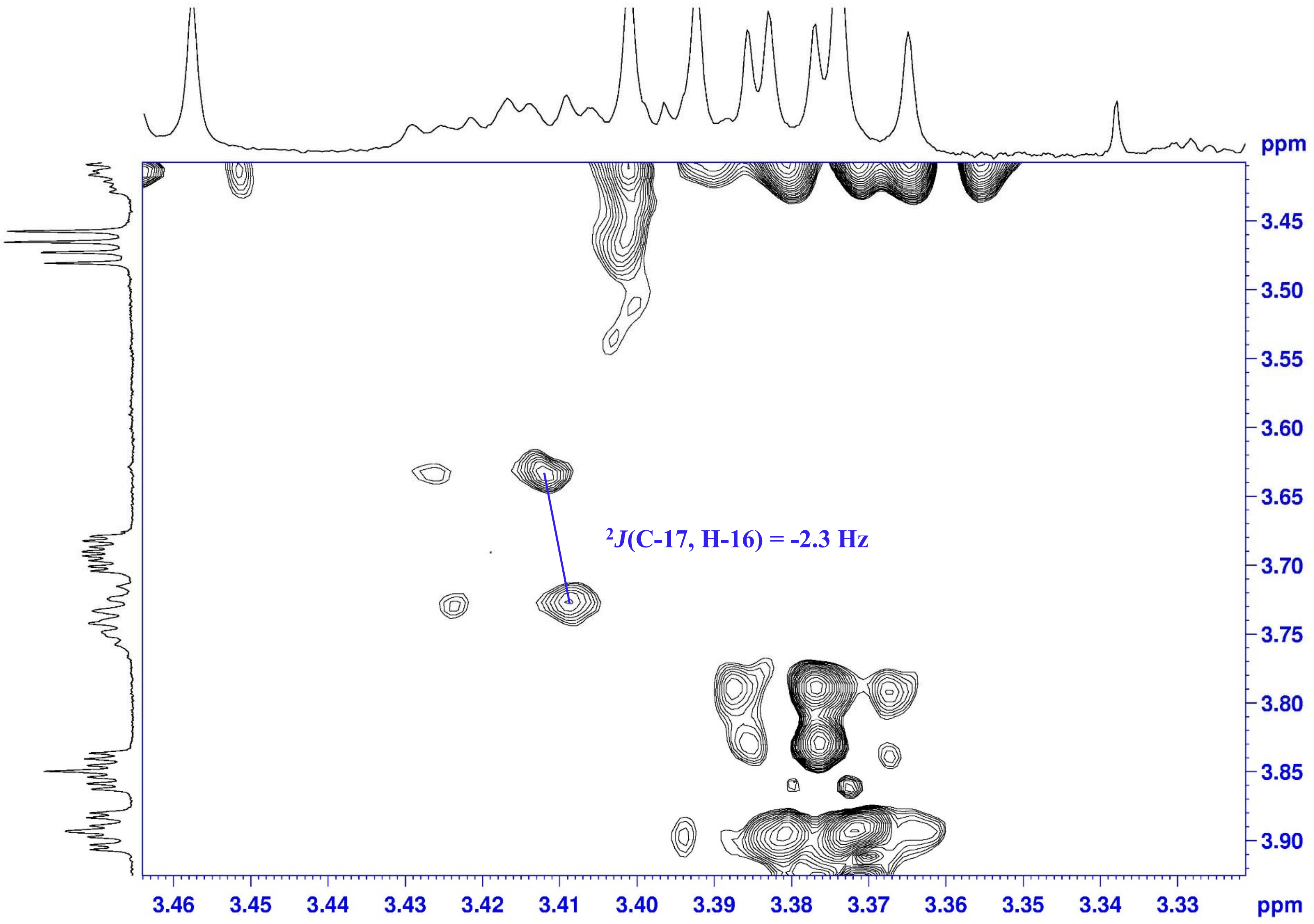

LR-ESI-MS for the fragment **1c**

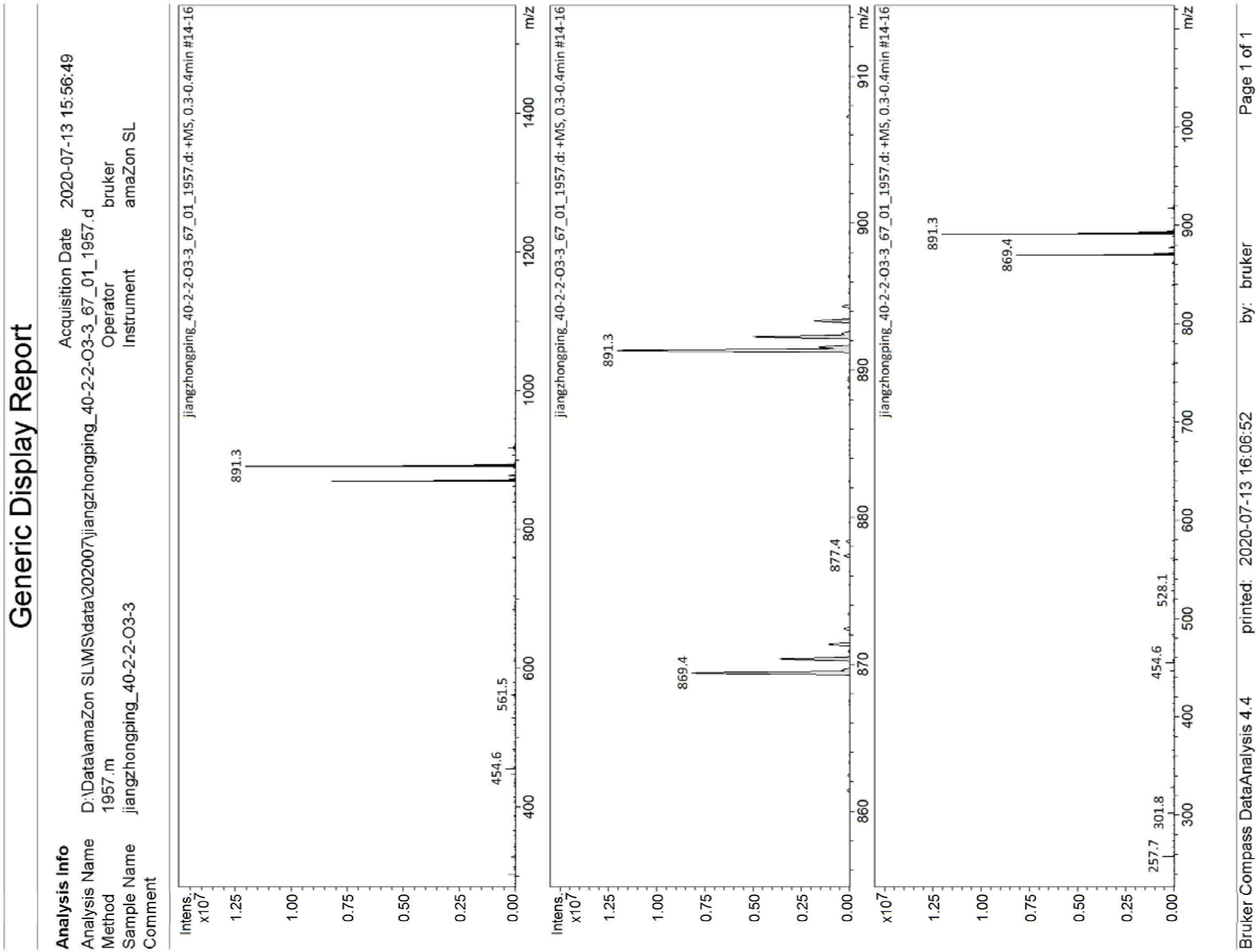

^{1}H (700 MHz) NMR spectrum of the fragment **1c** in CD_3OD

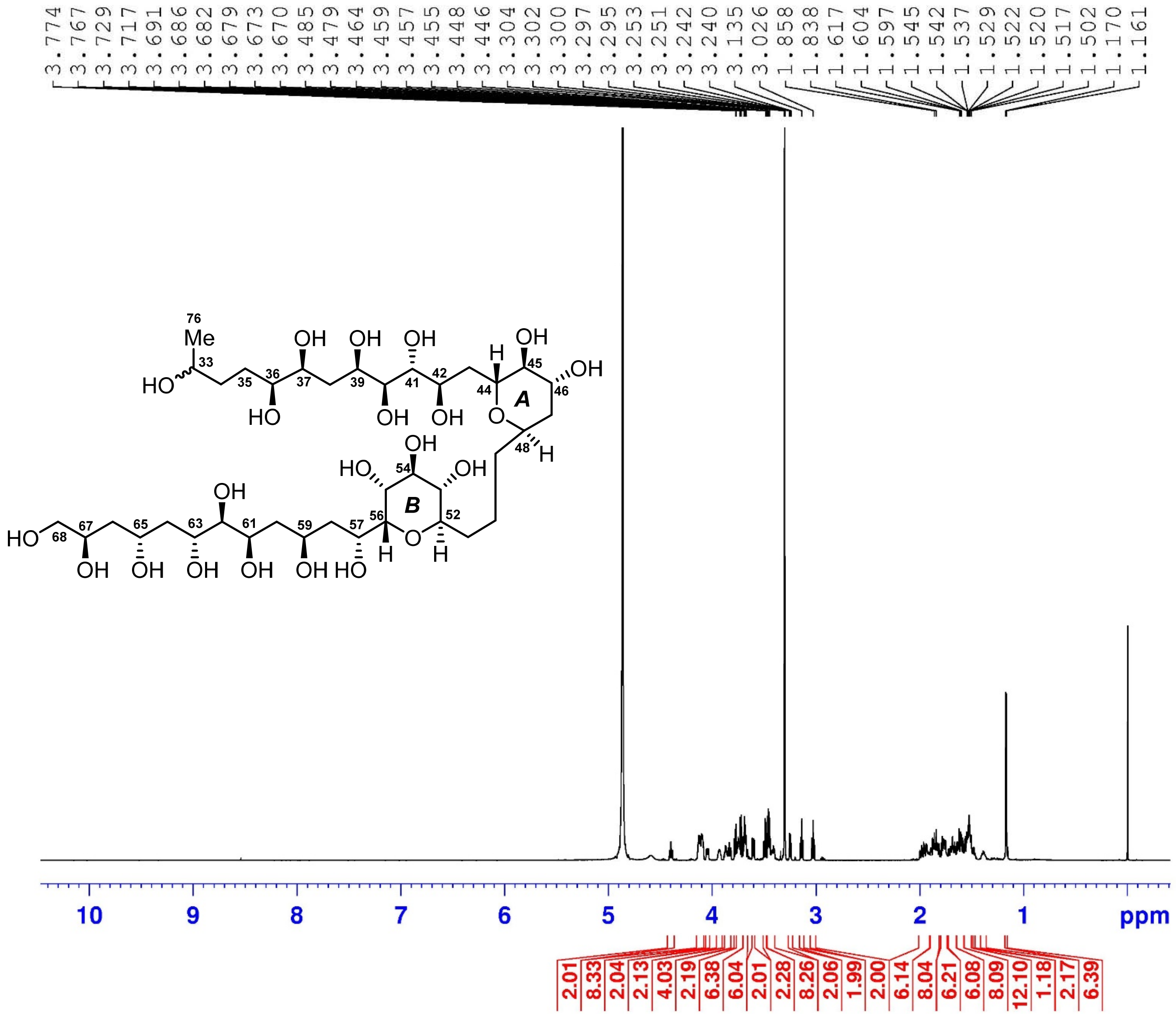

NAME	liwanshan-40-2-2-03-
EXPNO	12
PROCNO	1
Date_	20200321
Time	14.57 h
INSTRUM	spect
PROBHD	Z120187_0028 (
PULPROG	zg30
TD	65536
SOLVENT	MeOD
NS	32
DS	2
SWH	14097.744 Hz
FIDRES	0.430229 Hz
AQ	2.3243935 sec
RG	4.29
DW	35.467 usec
DE	10.00 usec
TE	298.0 K
D1	1.50000000 sec
TD0	1
SFO1	700.1843236 MHz
NUC1	1H
P1	7.55 usec
SI	65536
SF	700.1800209 MHz
WDW	EM
SSB	0
LB	0.30 Hz
GB	0
PC	1.00

^{1}H (700 MHz) NMR spectrum of the fragment **1c** in CD_3OD

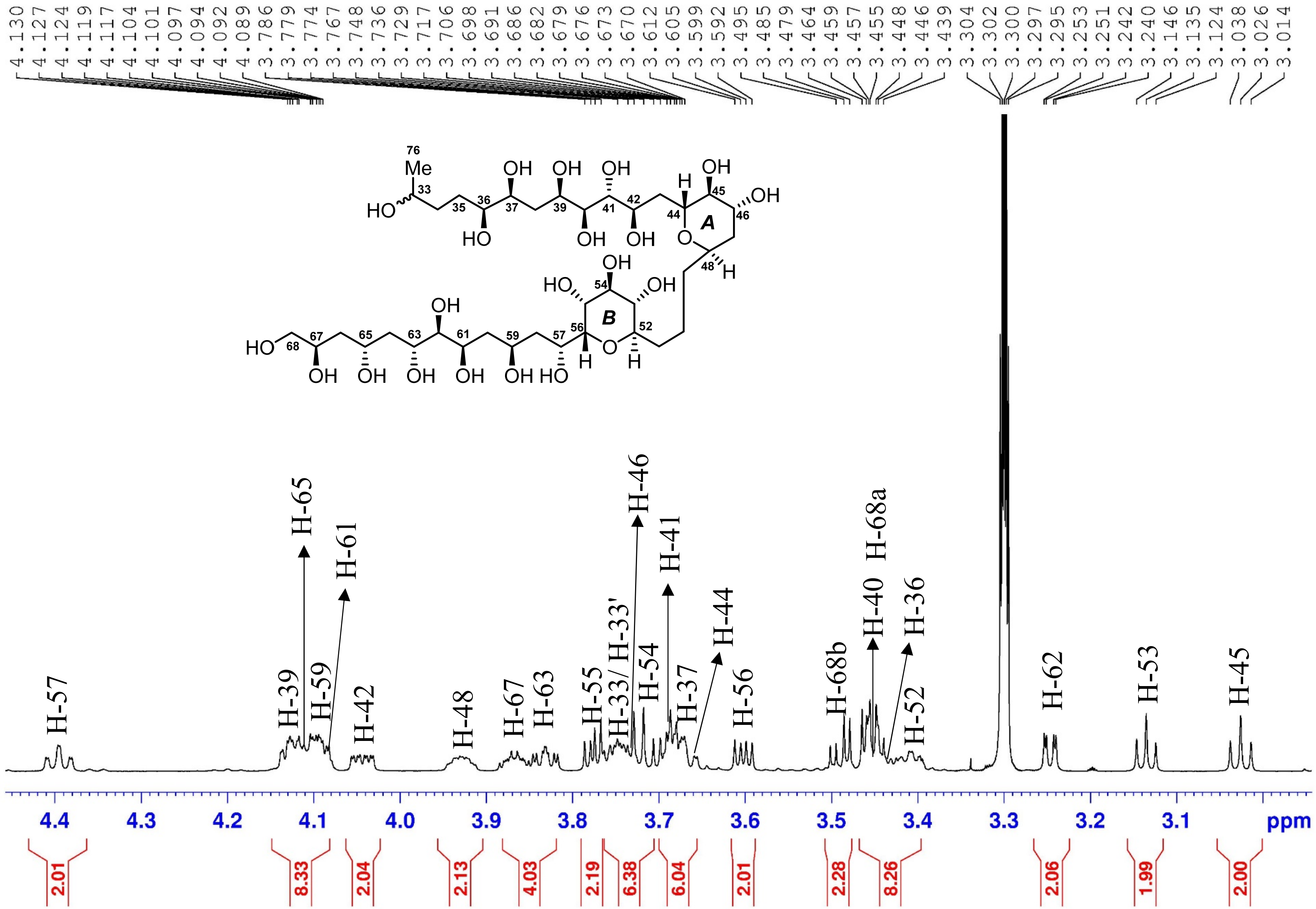

^{1}H (700 MHz) NMR spectrum of the fragment **1c** in CD_3OD

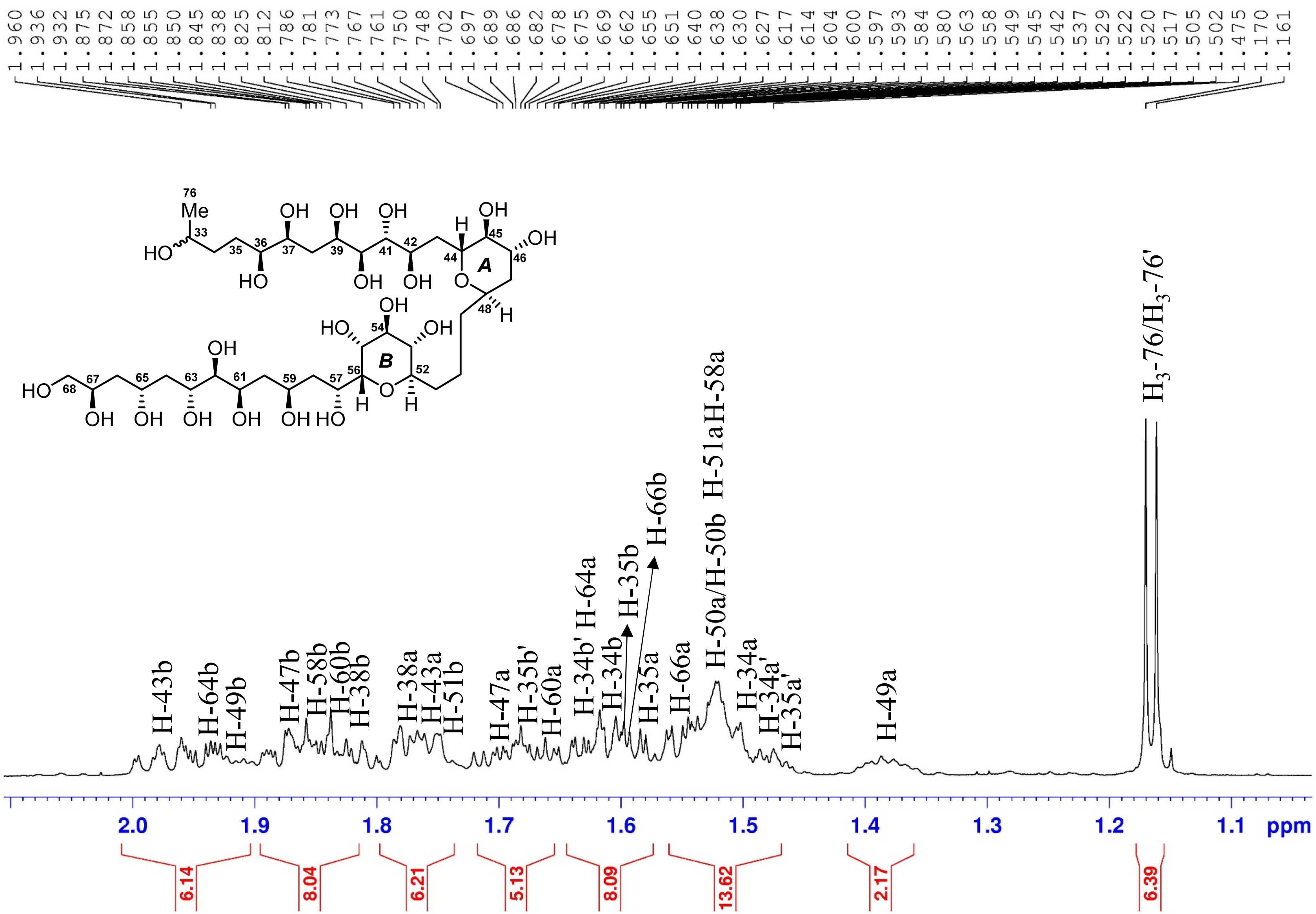

^{13}C (175 MHz) NMR spectrum of the fragment **1c** in CD_3OD

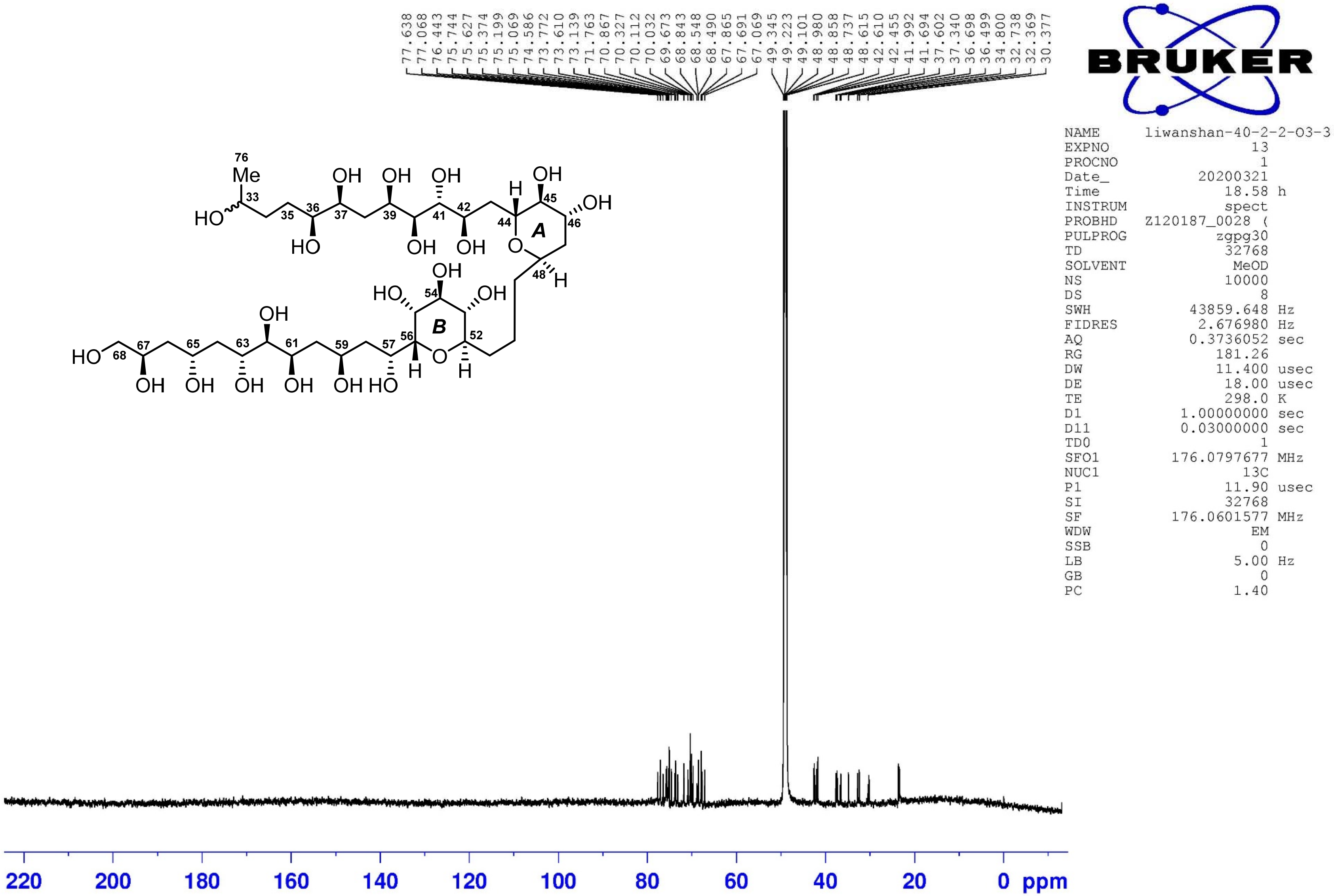

NAME	liwanshan-40-2-2-03-3	
EXPNO	13	
PROCNO	1	
Date_	20200321	
Time	18.58	h
INSTRUM	spect	
PROBHD	Z120187_0028 (	
PULPROG	zgpg30	
TD	32768	
SOLVENT	MeOD	
NS	10000	
DS	8	
SWH	43859.648	Hz
FIDRES	2.676980	Hz
AQ	0.3736052	sec
RG	181.26	
DW	11.400	usec
DE	18.00	usec
TE	298.0	K
D1	1.00000000	sec
D11	0.03000000	sec
TD0	1	
SFO1	176.0797677	MHz
NUC1	13C	
P1	11.90	usec
SI	32768	
SF	176.0601577	MHz
WDW	EM	
SSB	0	
LB	5.00	Hz
GB	0	
PC	1.40	

^{13}C (175 MHz) NMR spectrum of the fragment **1c** in CD_3OD

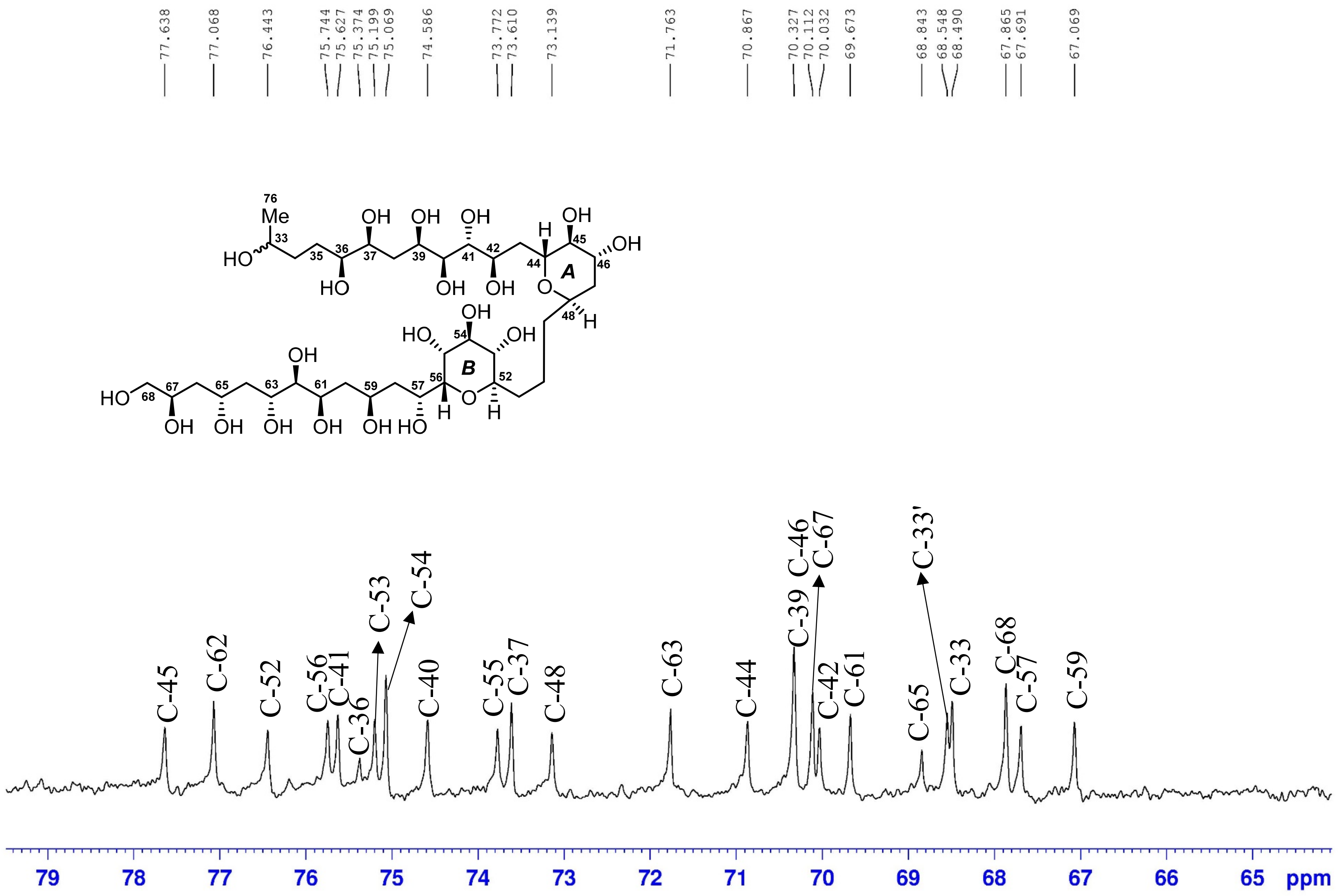

^{13}C (175 MHz) NMR spectrum of the fragment **1c** in CD_3OD

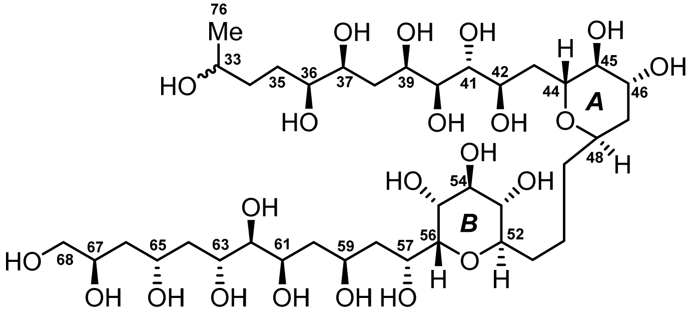

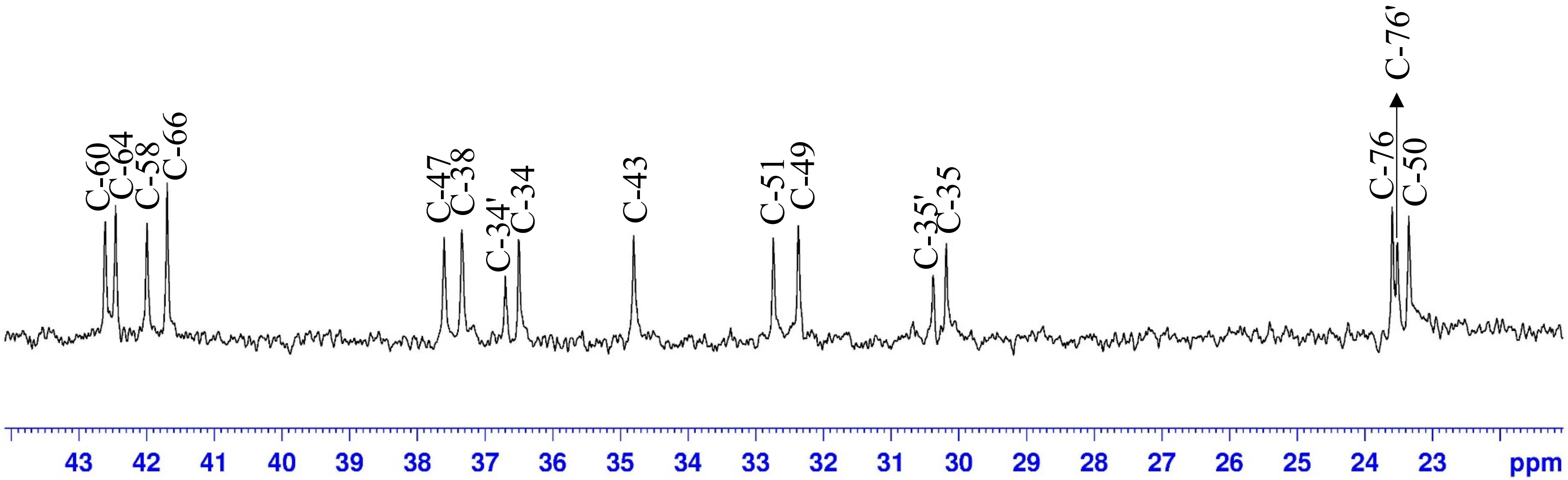

DEPT135 (175 MHz) spectrum of the fragment **1c** in CD_3OD

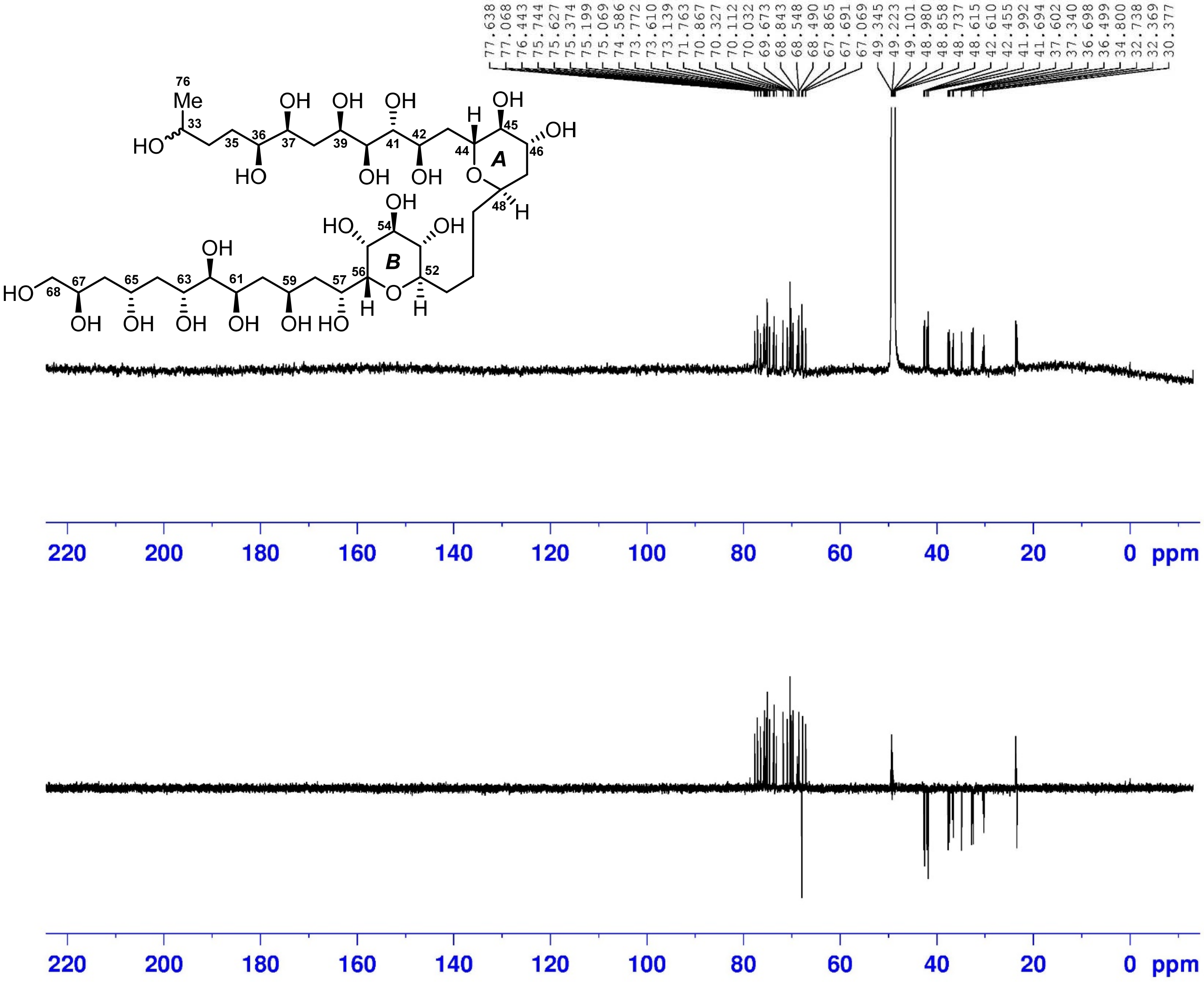


```
NAME        liwanshan-40-2-2-03-3
EXPNO                  14
PROCNO                  1
Date_            20200321
Time                20.33 h
INSTRUM             spect
PROBHD     Z120187_0028 (
PULPROG        deptsp135
TD                  32768
SOLVENT              MeOD
NS                   4000
DS                      8
SWH             43859.648 Hz
FIDRES           2.676980 Hz
AQ              0.3736052 sec
RG                 181.26
DW                 11.400 usec
DE                  18.00 usec
TE                  298.0 K
CNST2       145.0000000
D1           1.00000000 sec
D2           0.00344828 sec
D12          0.00002000 sec
TD0                     1
SFO1        176.0797677 MHz
NUC1                  13C
P1                  11.90 usec
P13               2000.00 usec
SI                  32768
SF          176.0601605 MHz
WDW                    EM
SSB                     0
LB                   1.00 Hz
GB                      0
PC                   1.40
```

DEPT135 (175 MHz) spectrum of the fragment **1c** in CD_3OD

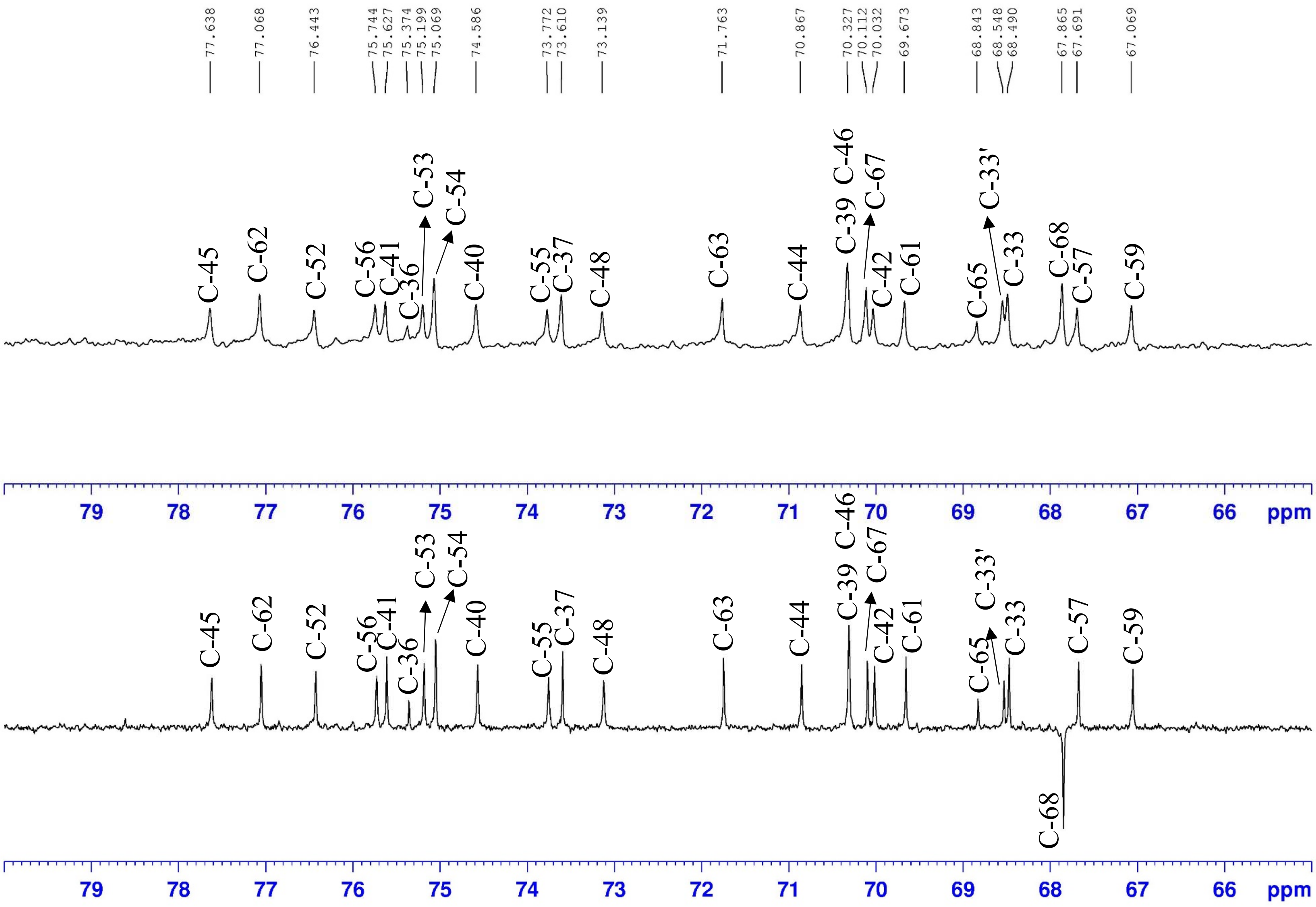

DEPT135 (175 MHz) spectrum of the fragment **1c** in CD_3OD

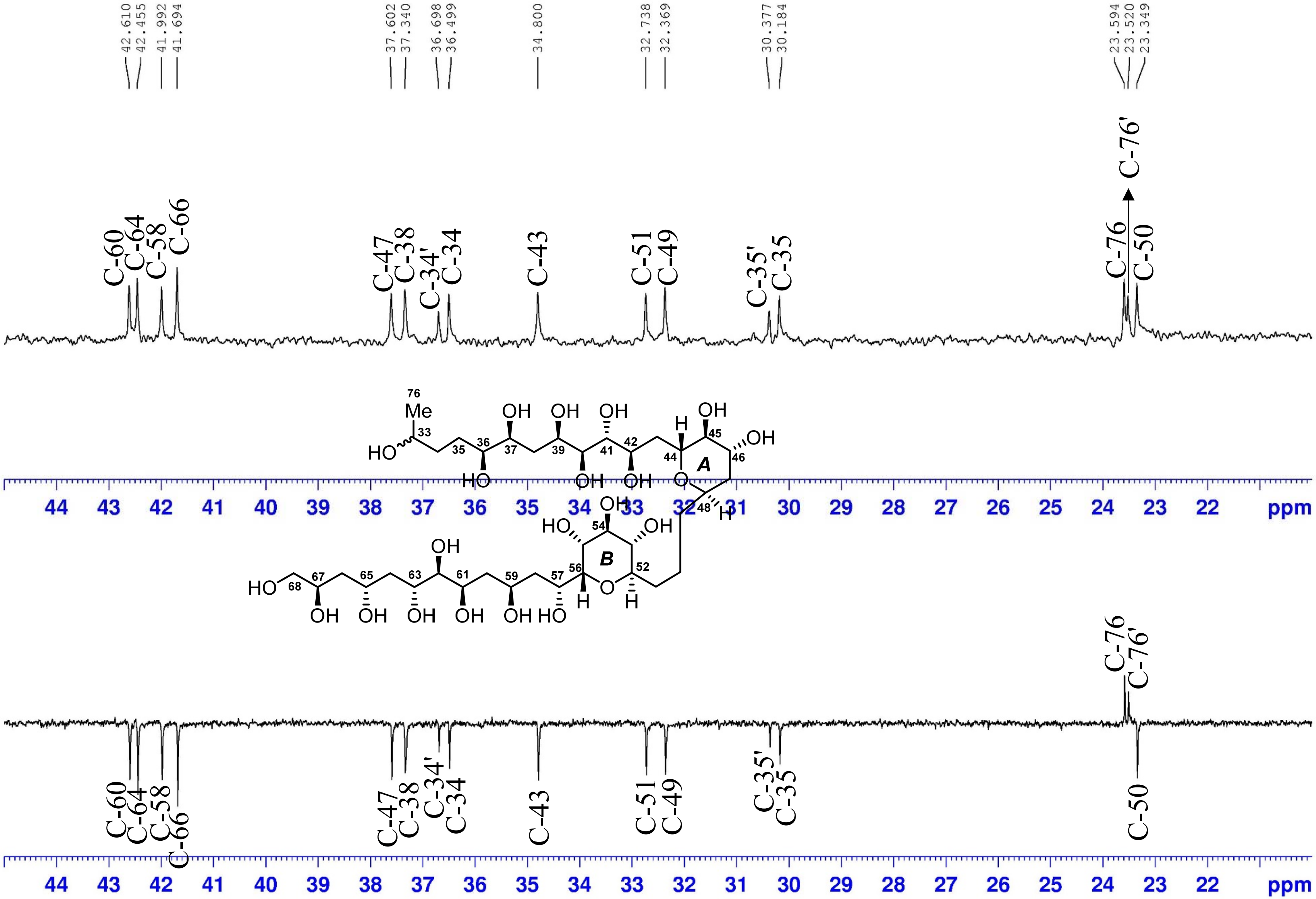

^{1}H–^{1}H COSY (700 MHz) spectrum of the fragment **1c** in CD_3OD

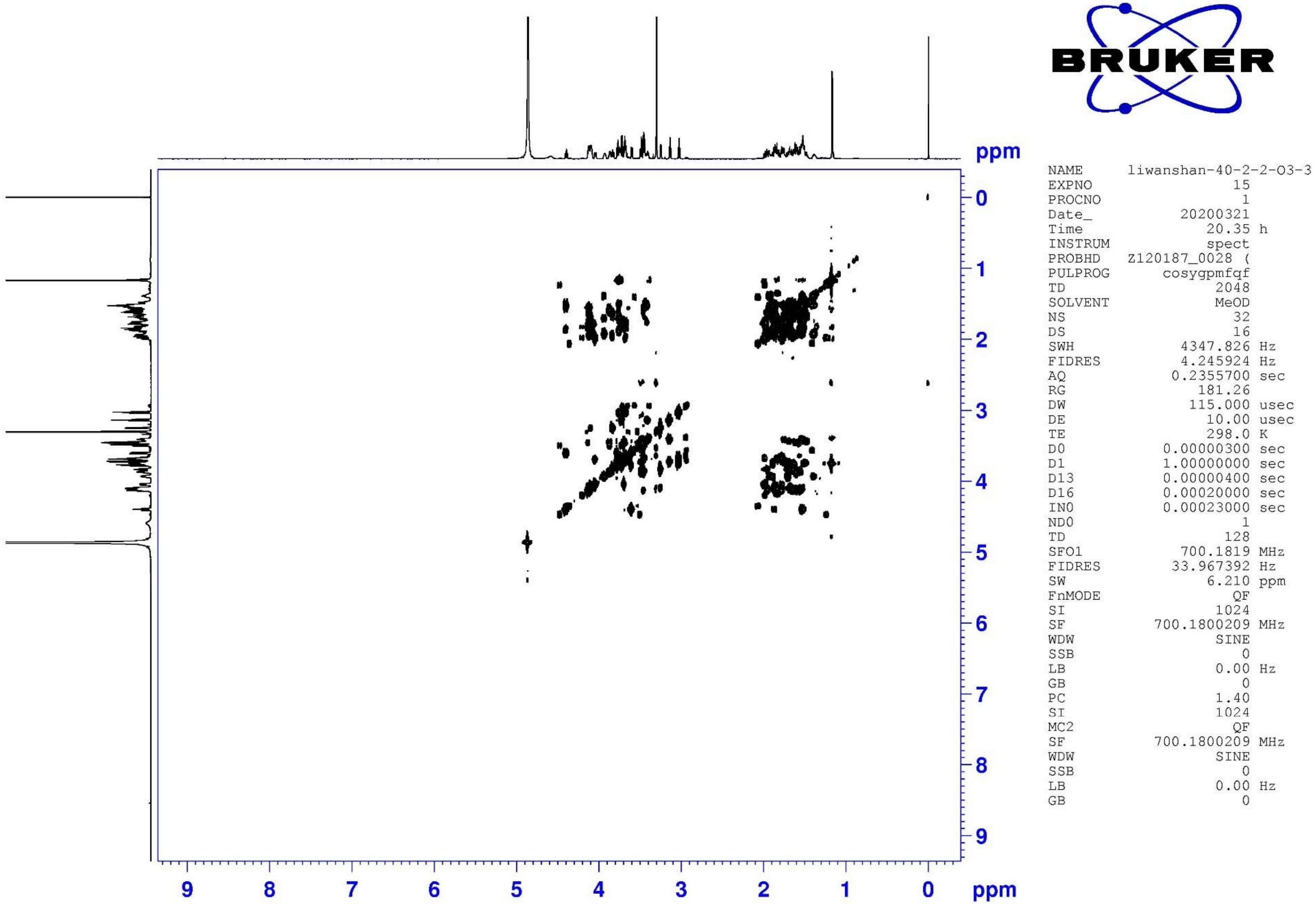

^{1}H–^{1}H COSY (700 MHz) spectrum of the fragment **1c** in CD_3OD

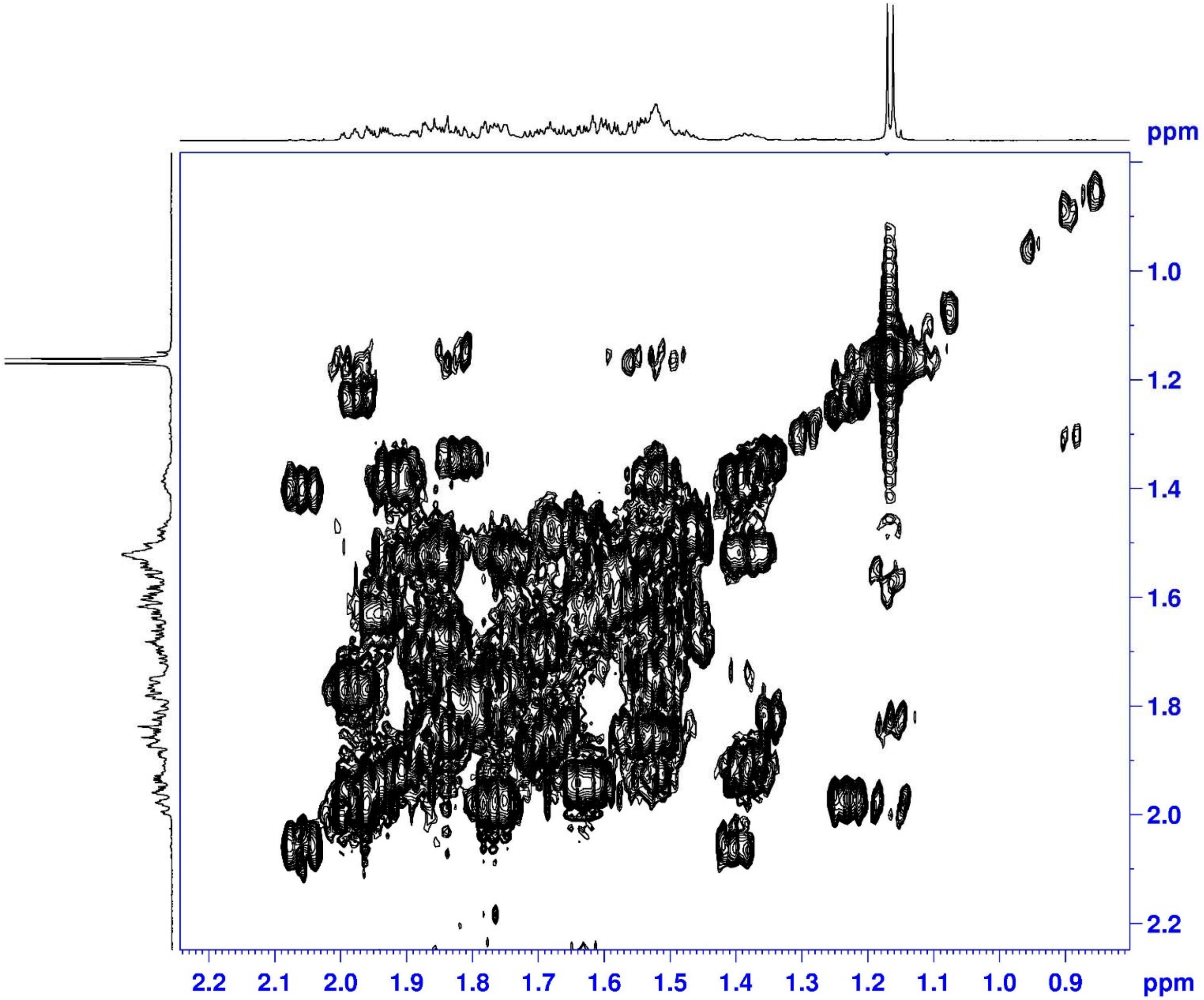

^{1}H–^{1}H COSY (700 MHz) spectrum of the fragment **1c** in CD_3OD

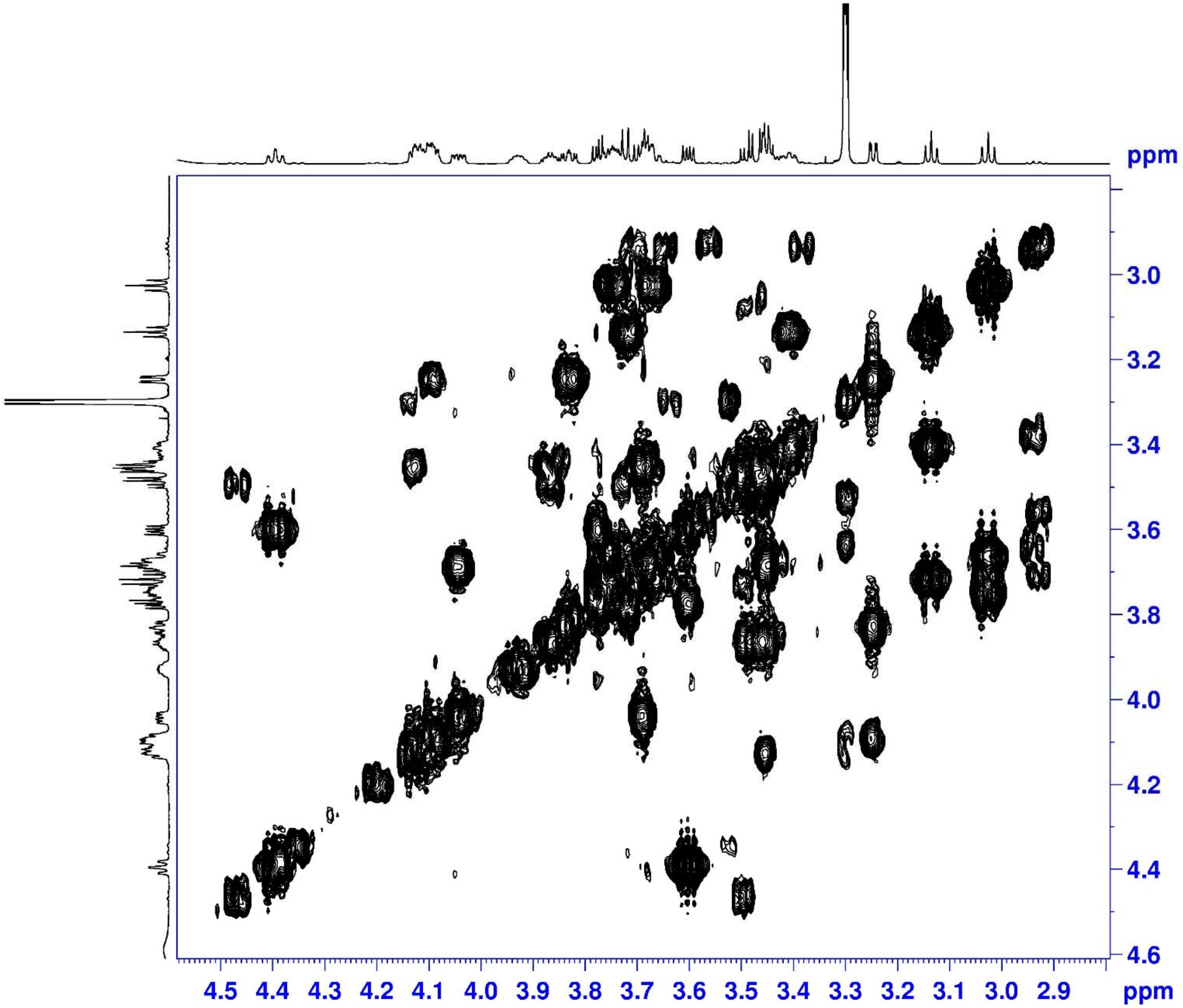

^{1}H–^{1}H COSY (700 MHz) spectrum of the fragment **1c** in CD_3OD

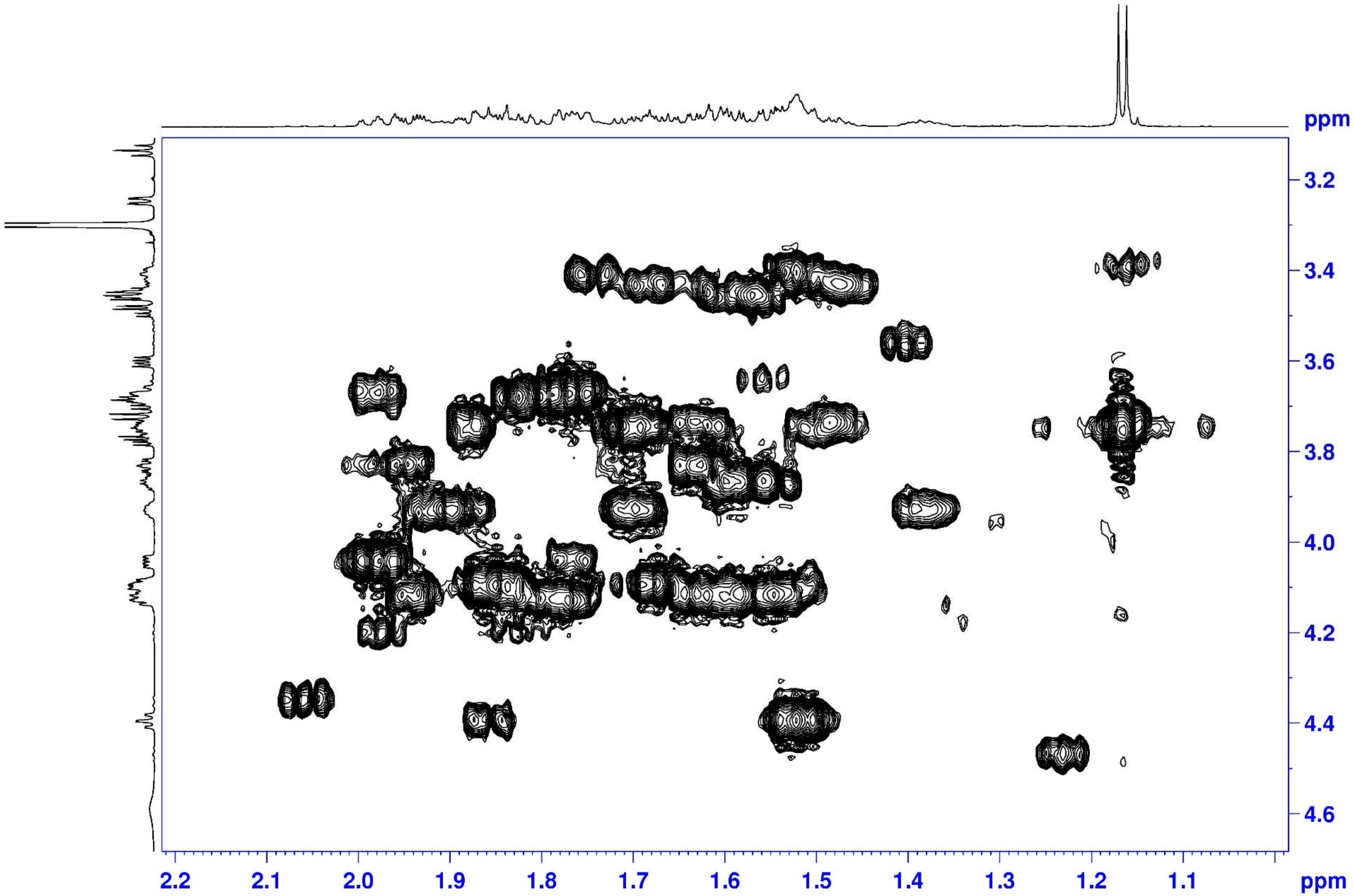

HSQC (700 MHz) spectrum of the fragment **1c** in CD_3OD

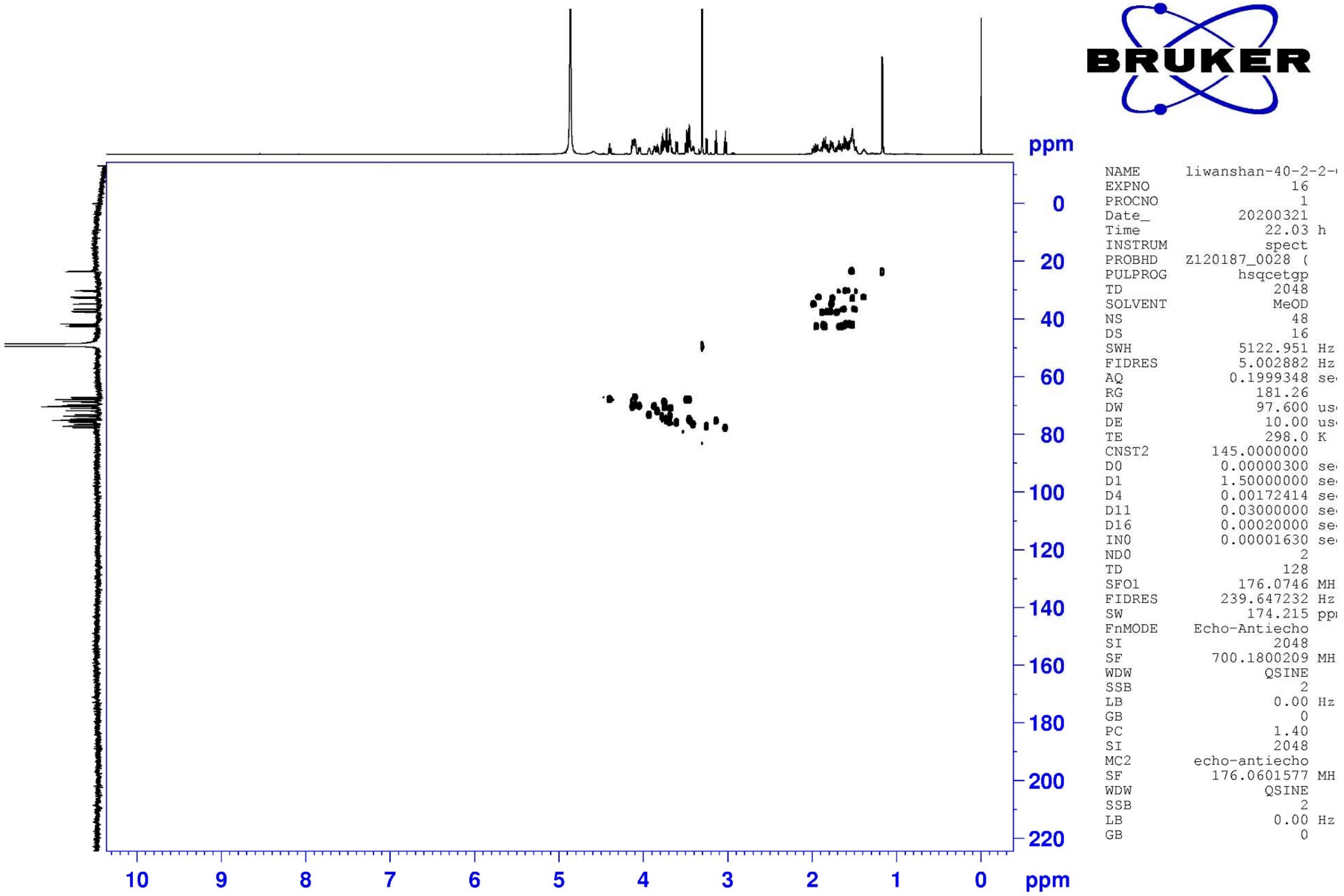

HSQC (700 MHz) spectrum of the fragment **1c** in CD_3OD

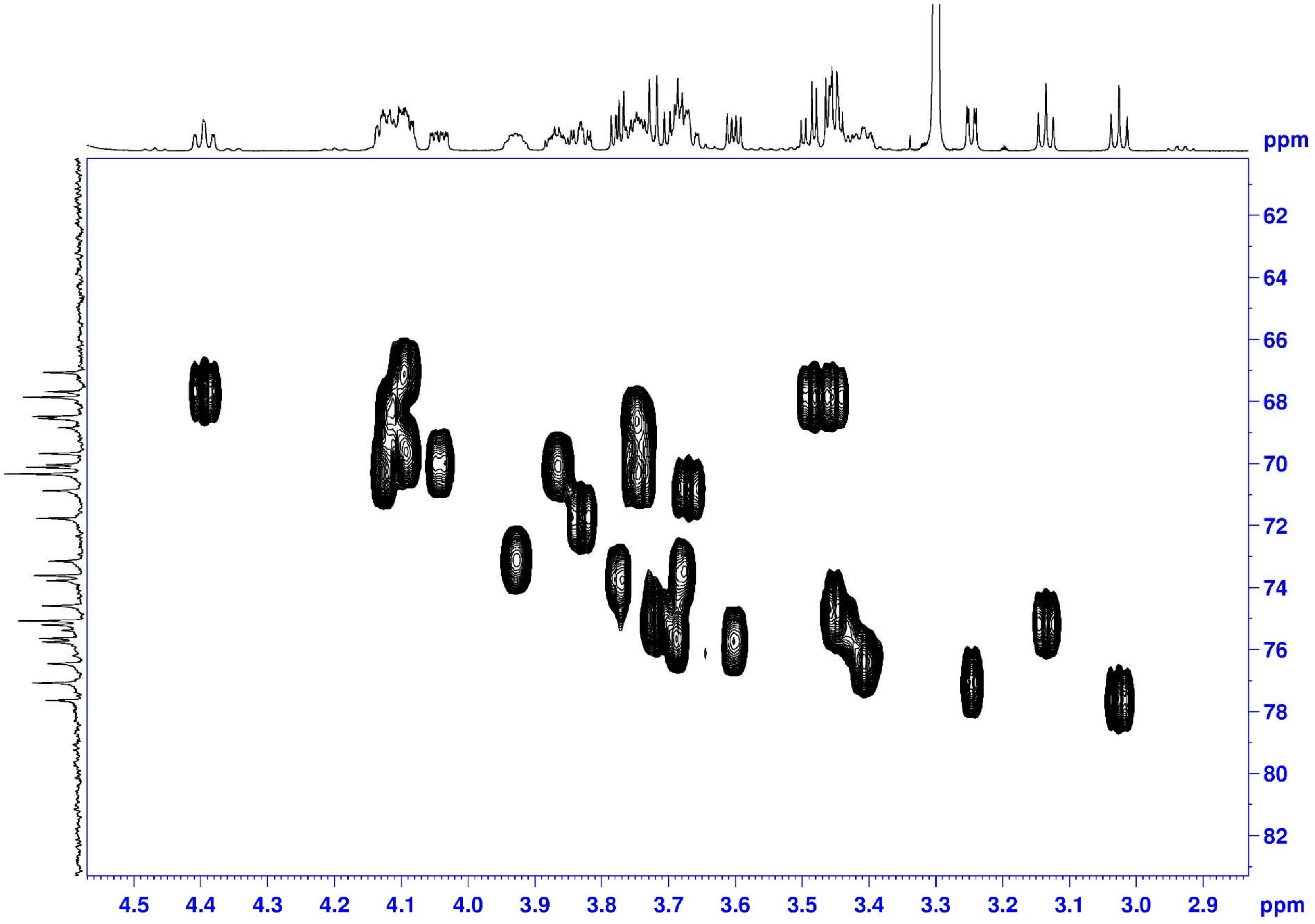

HSQC (700 MHz) spectrum of the fragment **1c** in CD_3OD

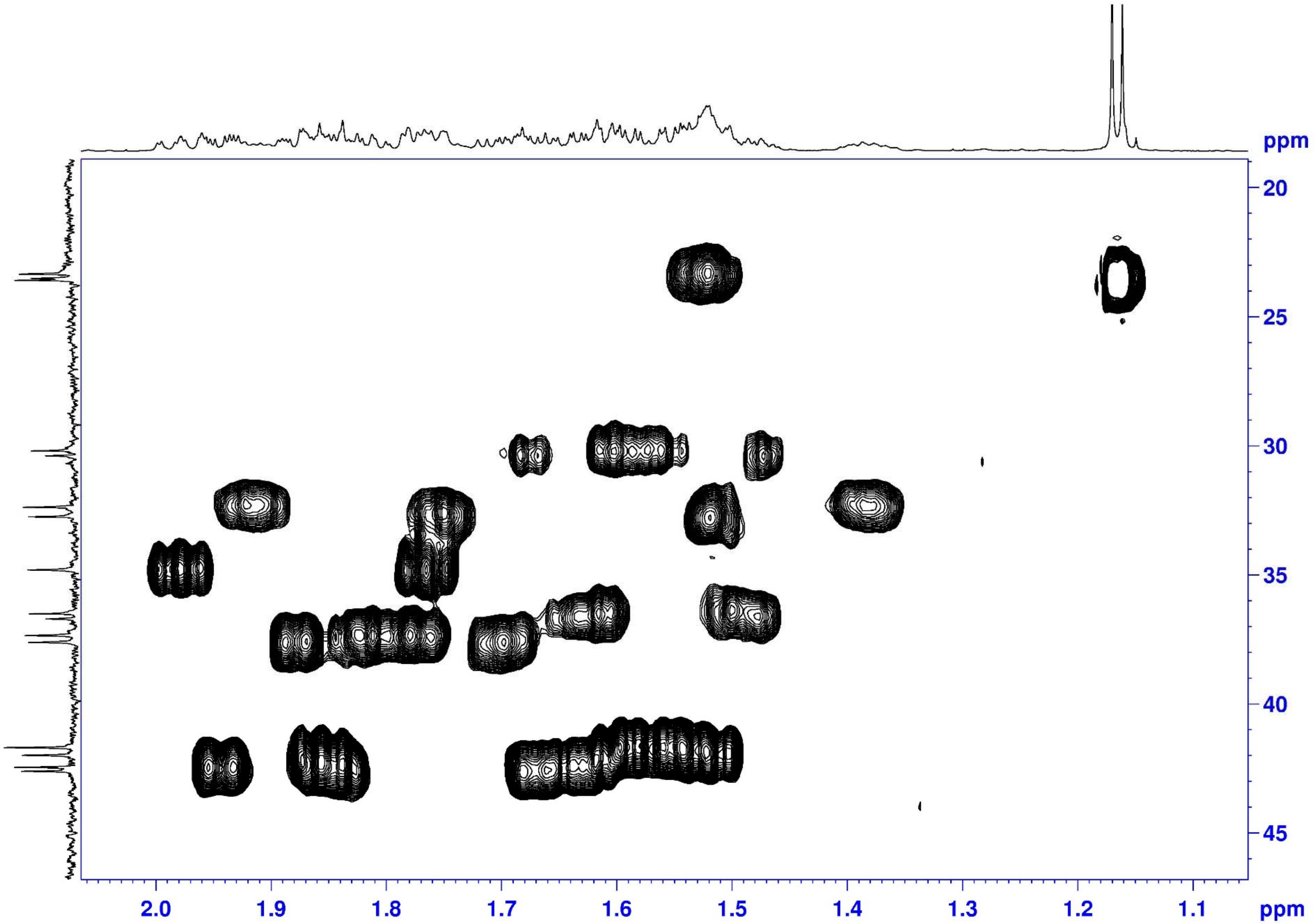

HMBC (700 MHz) spectrum of the fragment **1c** in CD_3OD

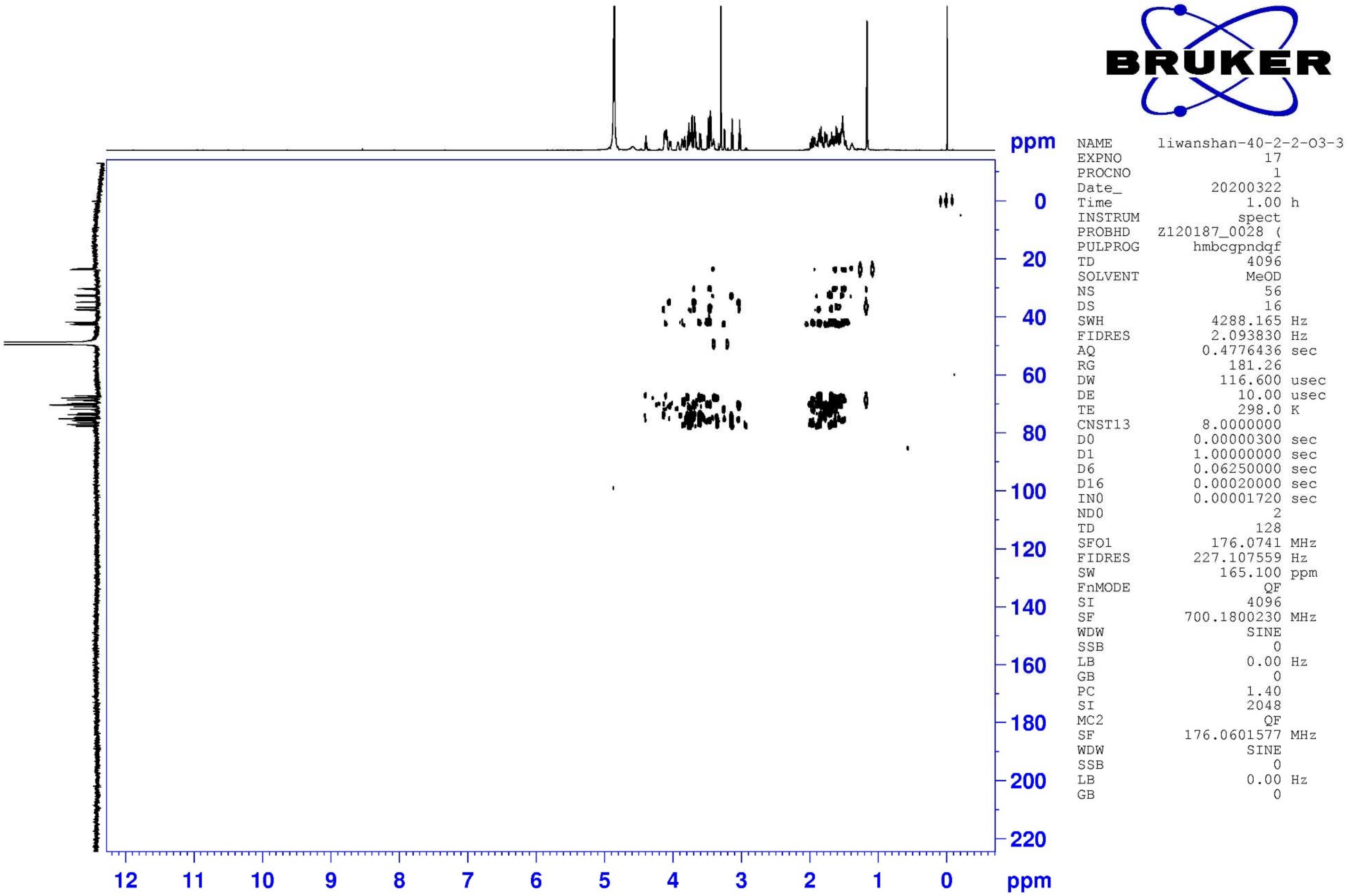

HMBC (700 MHz) spectrum of the fragment **1c** in CD_3OD

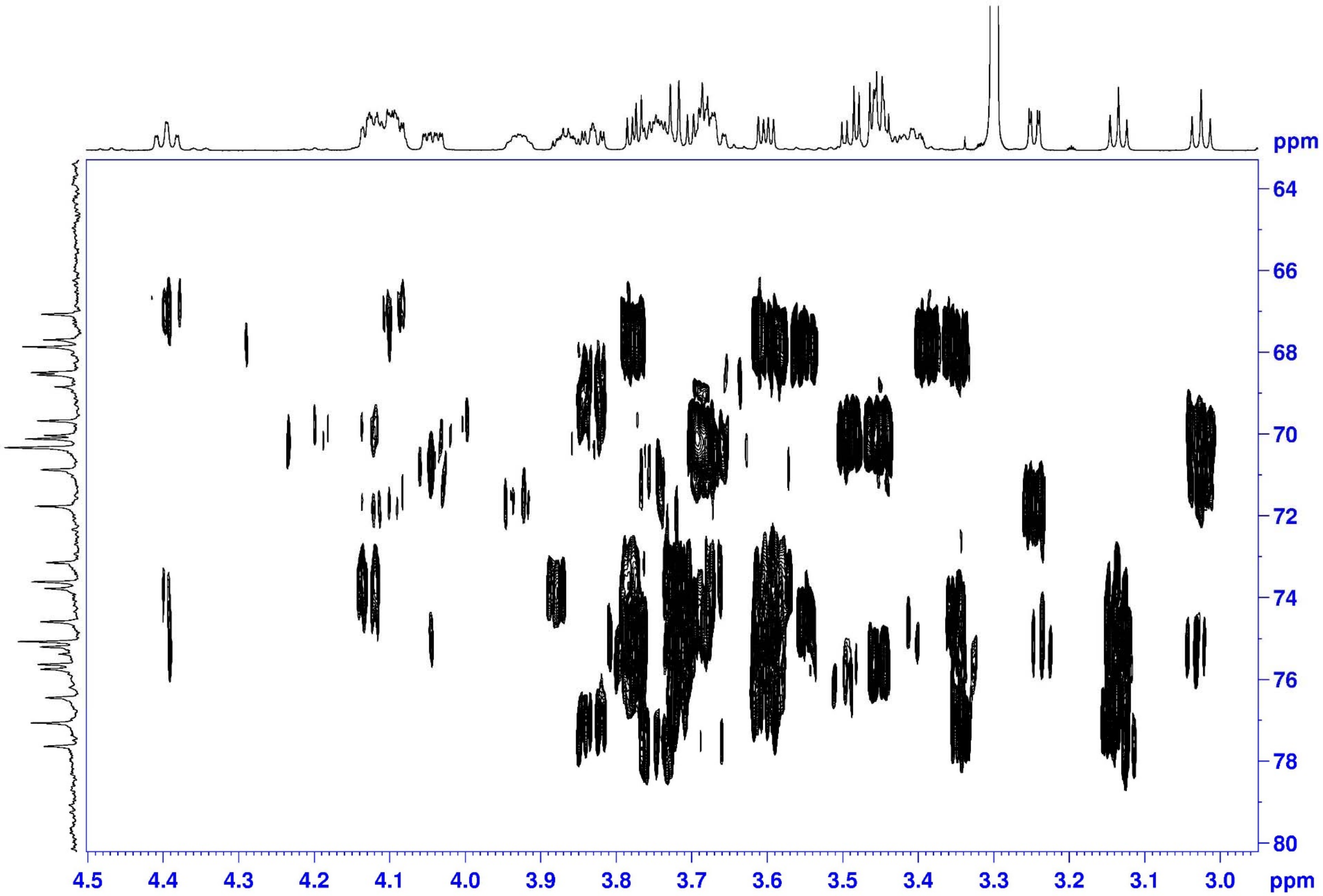

HMBC (700 MHz) spectrum of the fragment **1c** in CD_3OD

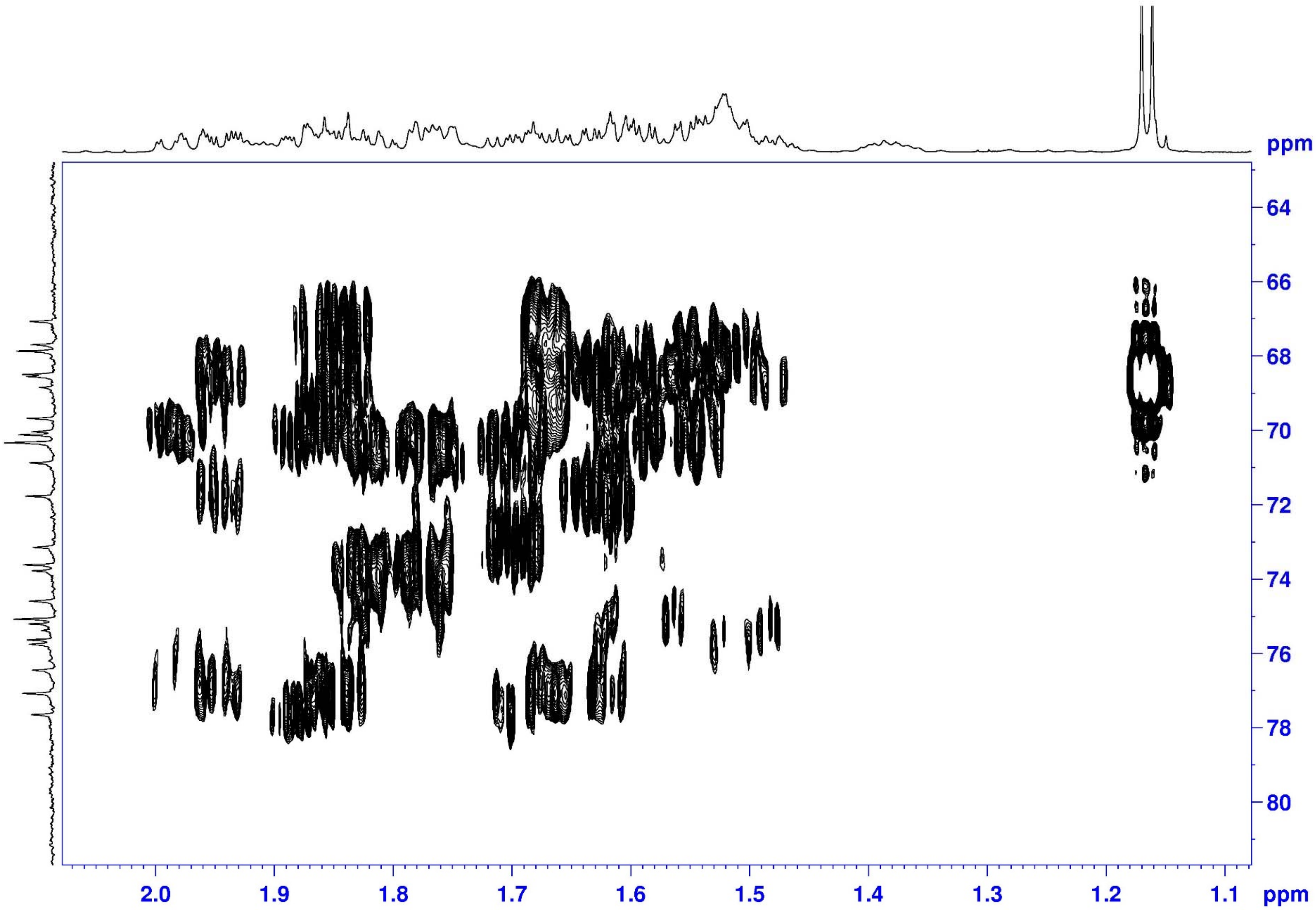

HMBC (700 MHz) spectrum of the fragment **1c** in CD_3OD

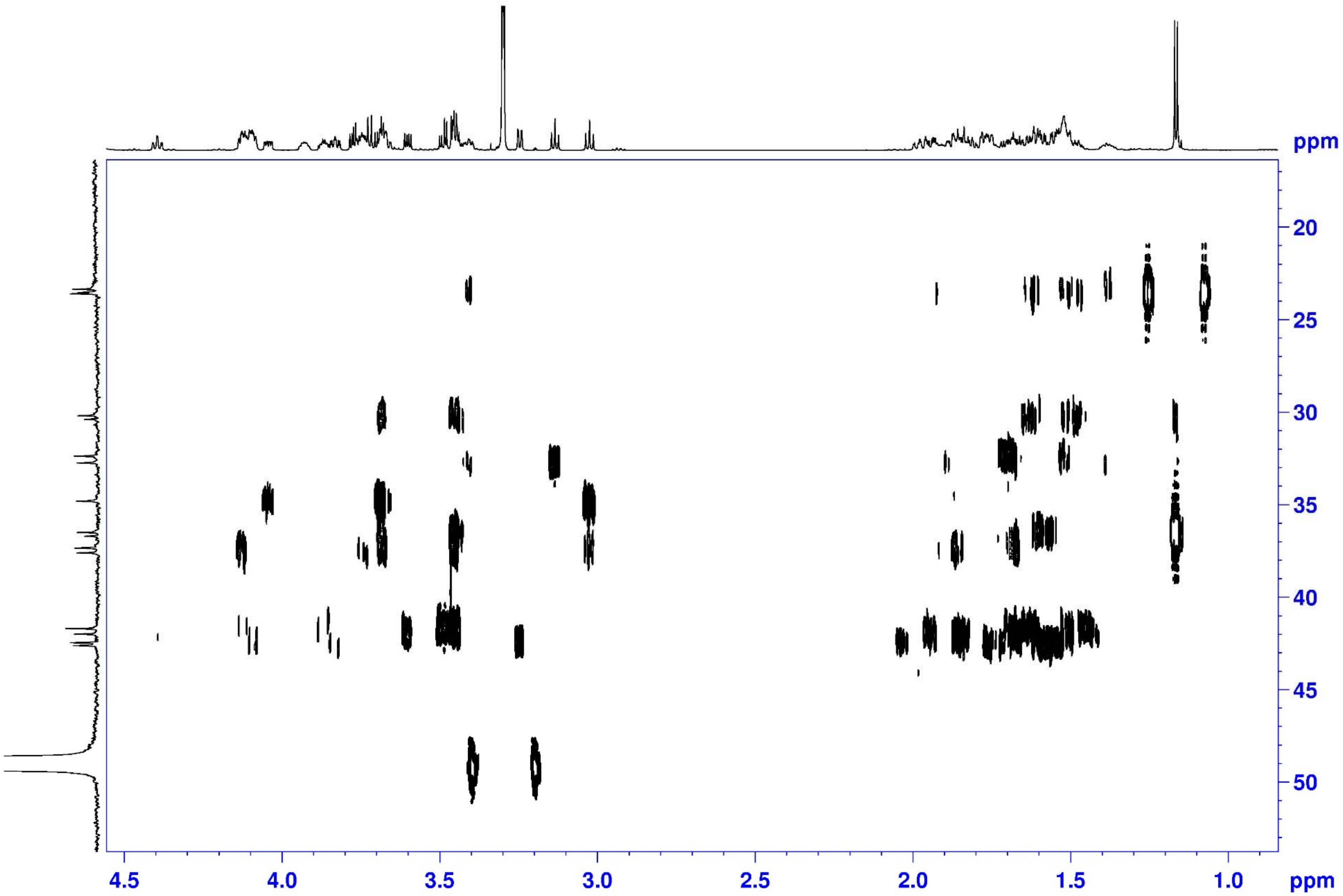

NOESY (700 MHz) spectrum of the fragment **1c** in CD_3OD

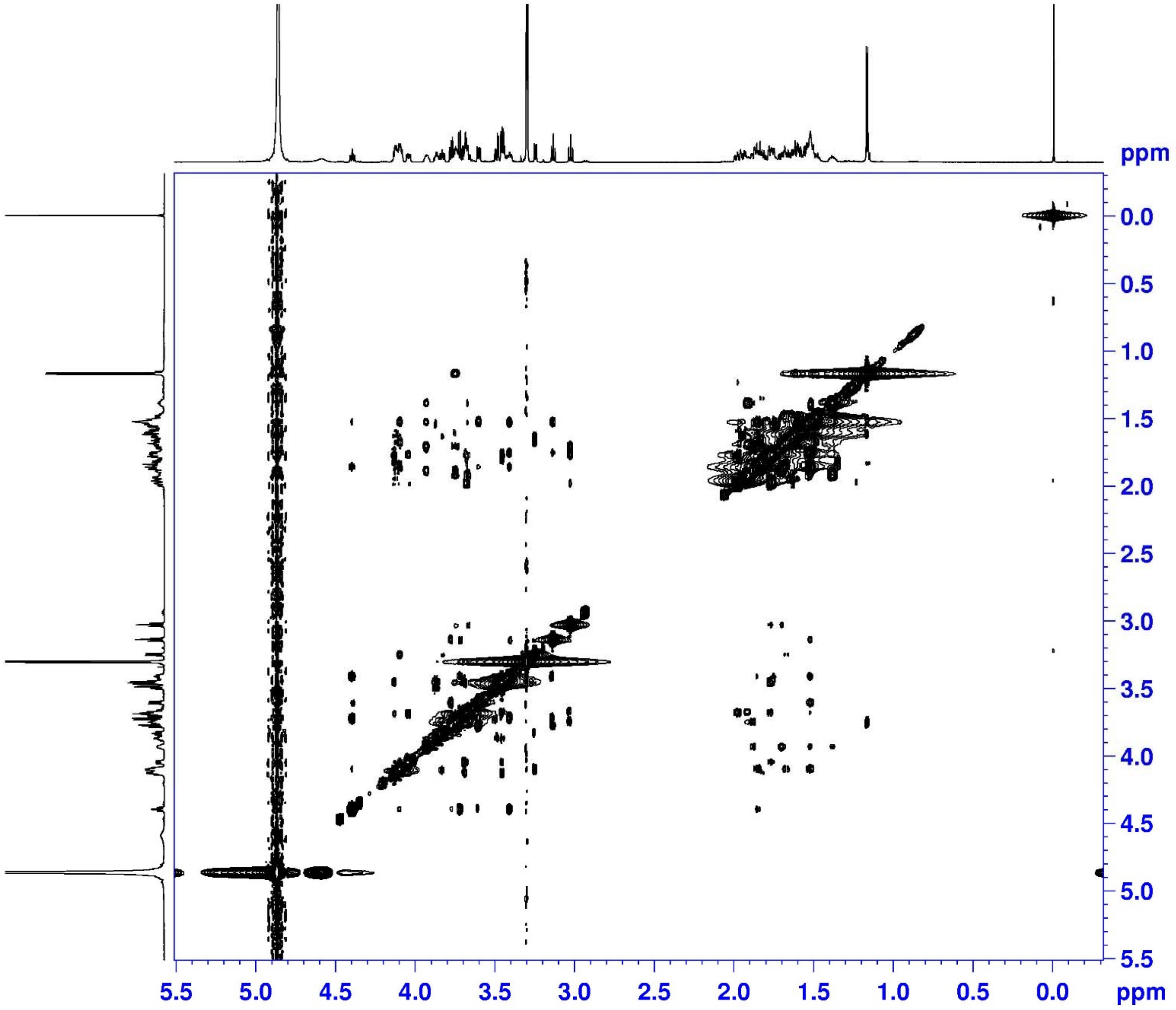

NAME	liwanshan-40-2-2-03-	
EXPNO	23	
PROCNO	1	
Date_	20200330	
Time	18.43	h
INSTRUM	spect	
PROBHD	Z120187_0028 (	
PULPROG	noesygpph	
TD	2048	
SOLVENT	MeOD	
NS	24	
DS	16	
SWH	4084.967	Hz
FIDRES	3.989226	Hz
AQ	0.2507252	sec
RG	15.56	
DW	122.400	usec
DE	10.00	usec
TE	298.0	K
D0	0.00011279	sec
D1	1.50000000	sec
D8	0.60000002	sec
D16	0.00020000	sec
IN0	0.00024480	sec
ND0	1	
TD	256	
SFO1	700.1818	MHz
FIDRES	15.956903	Hz
SW	5.834	ppm
FnMODE	TPPI	
SI	2048	
SF	700.1800209	MHz
WDW	QSINE	
SSB	2	
LB	0.00	Hz
GB	0	
PC	1.00	
SI	2048	
MC2	TPPI	
SF	700.1800209	MHz
WDW	QSINE	
SSB	2	
LB	0.00	Hz
GB	0	

NOESY (700 MHz) spectrum of the fragment **1c** in CD_3OD

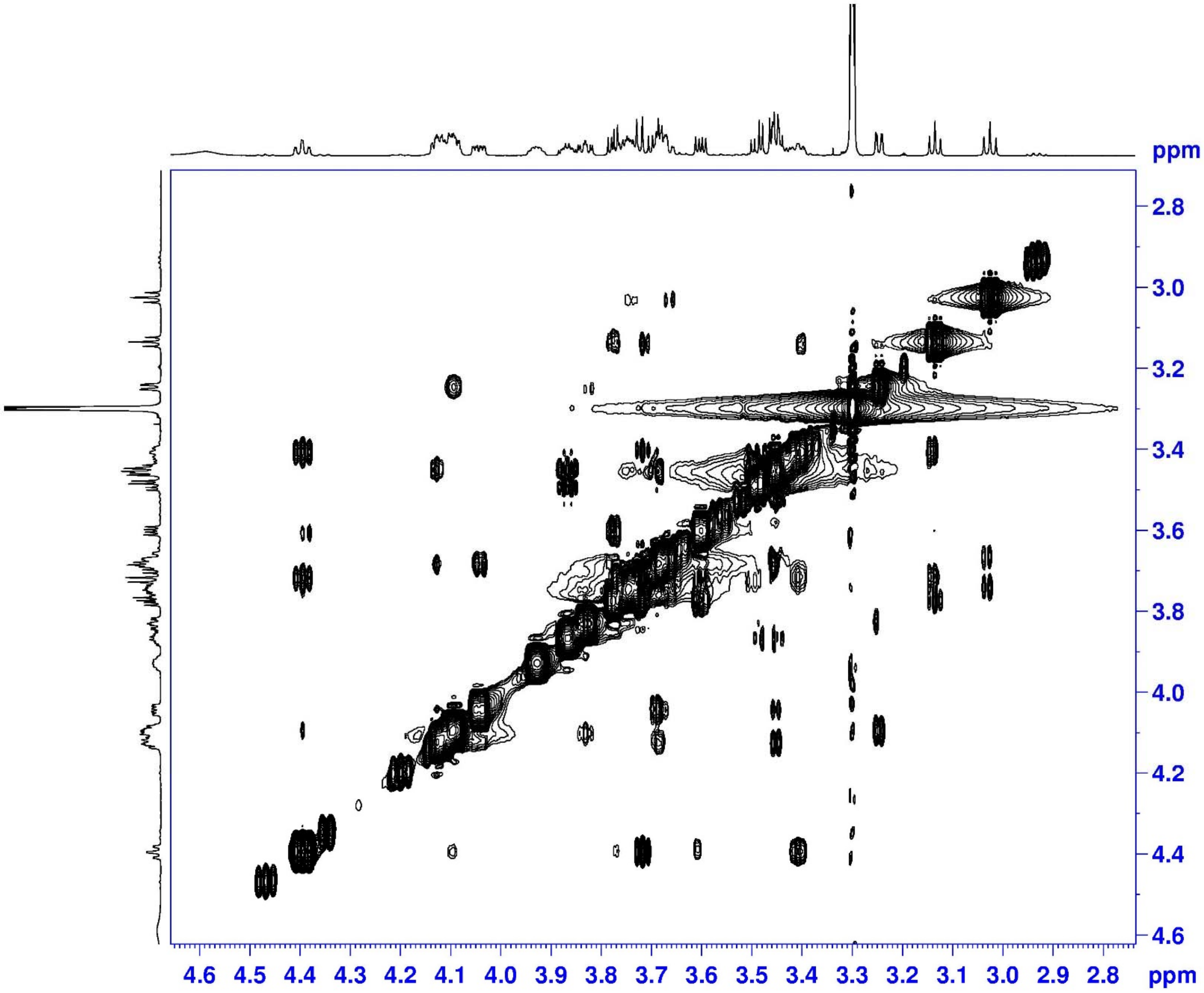

NOESY (700 MHz) spectrum of the fragment **1c** in CD_3OD

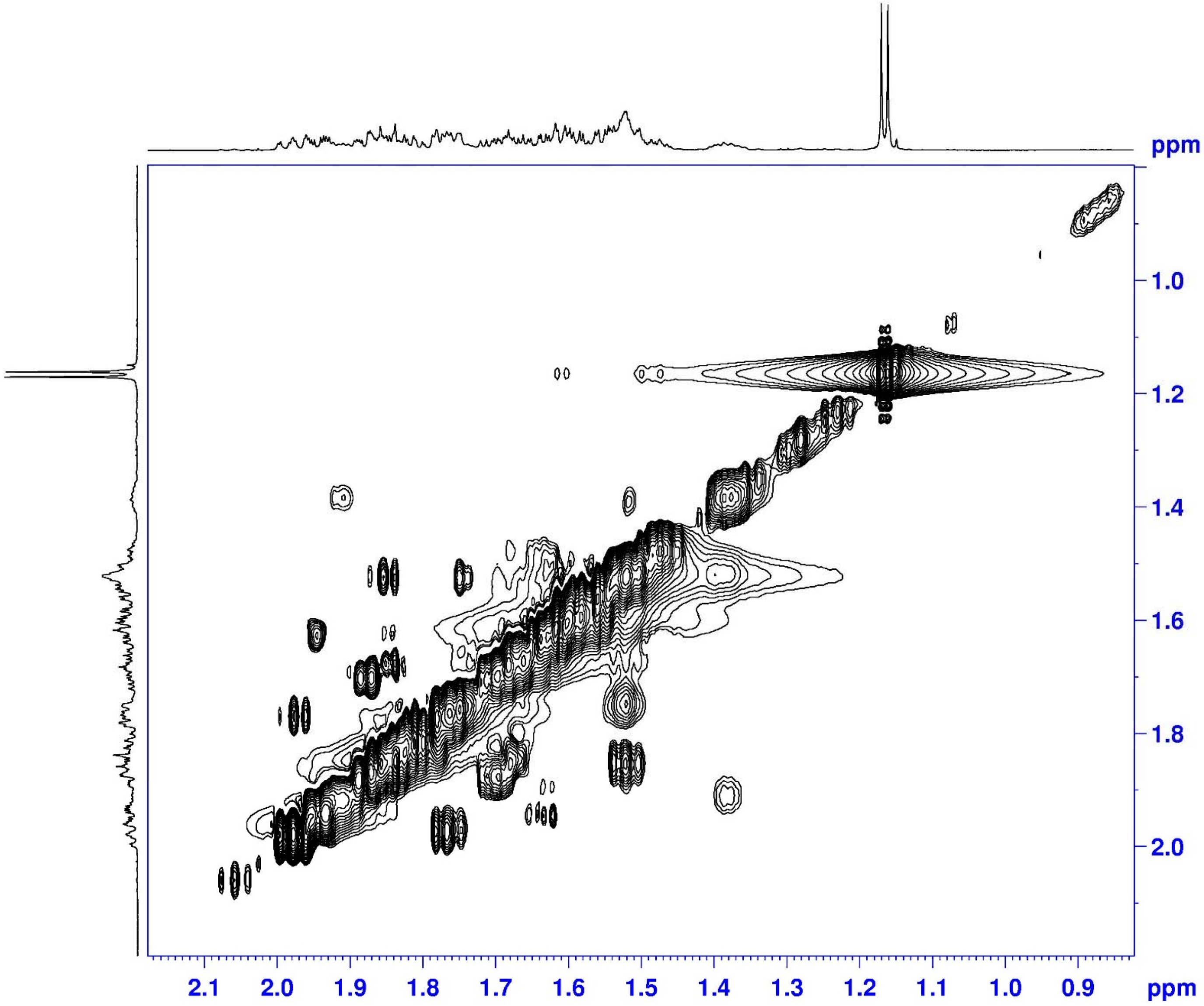

NOESY (700 MHz) spectrum of the fragment **1c** in CD_3OD

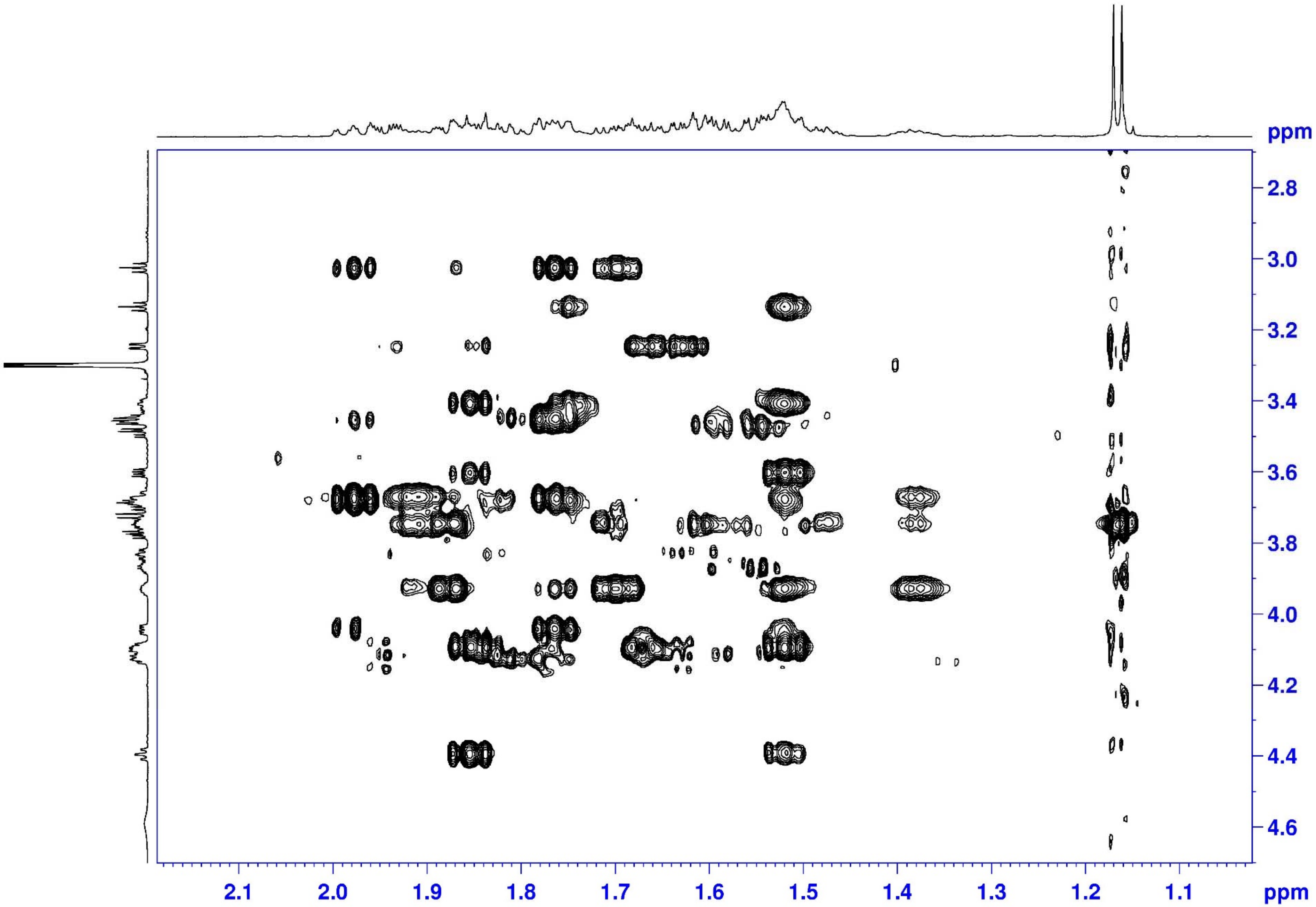

2D *J*RES (700 MHz) spectrum of the fragment **1c** in CD_3OD

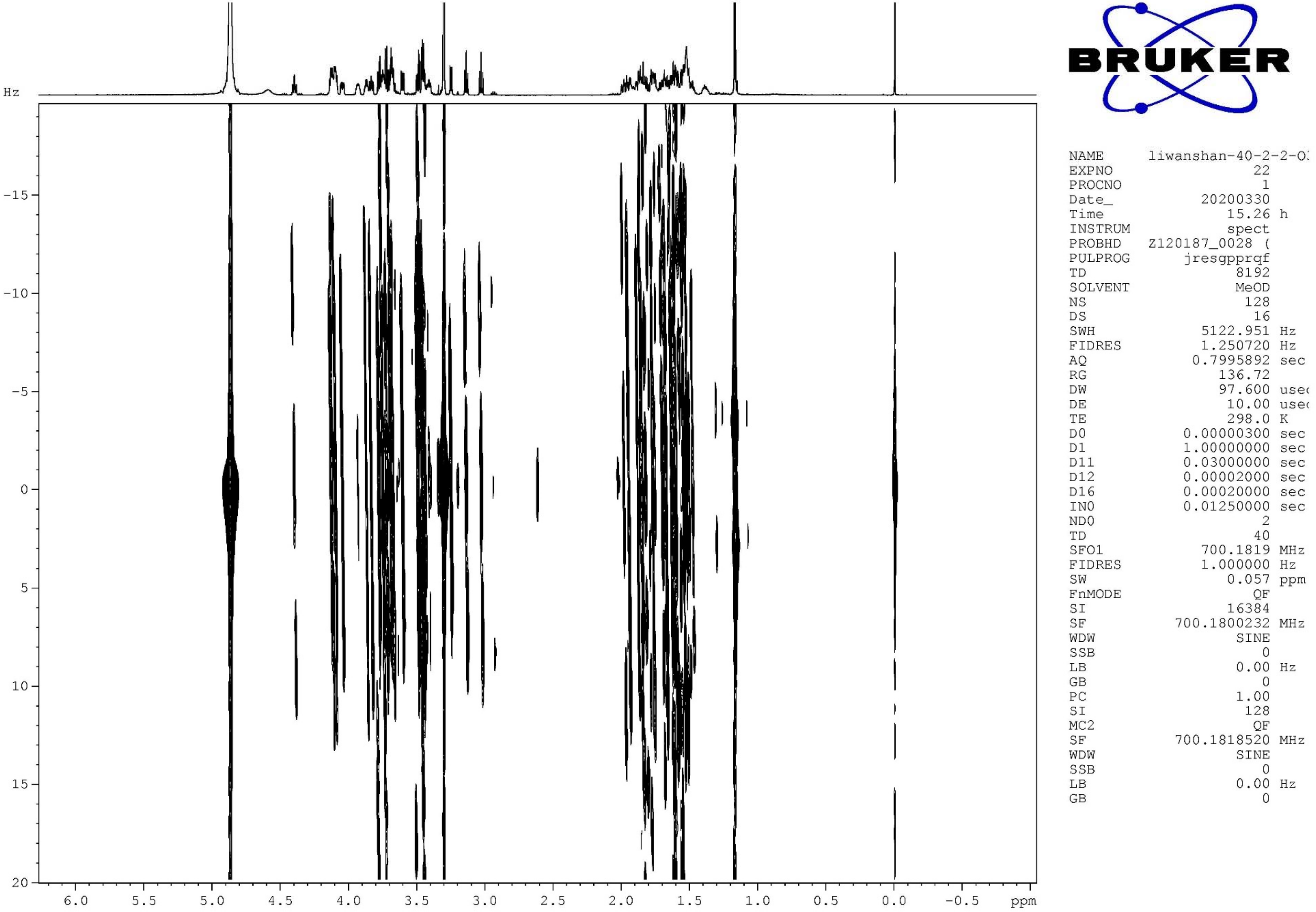

2D *J*RES (700 MHz) spectrum of the fragment **1c** in CD_3OD

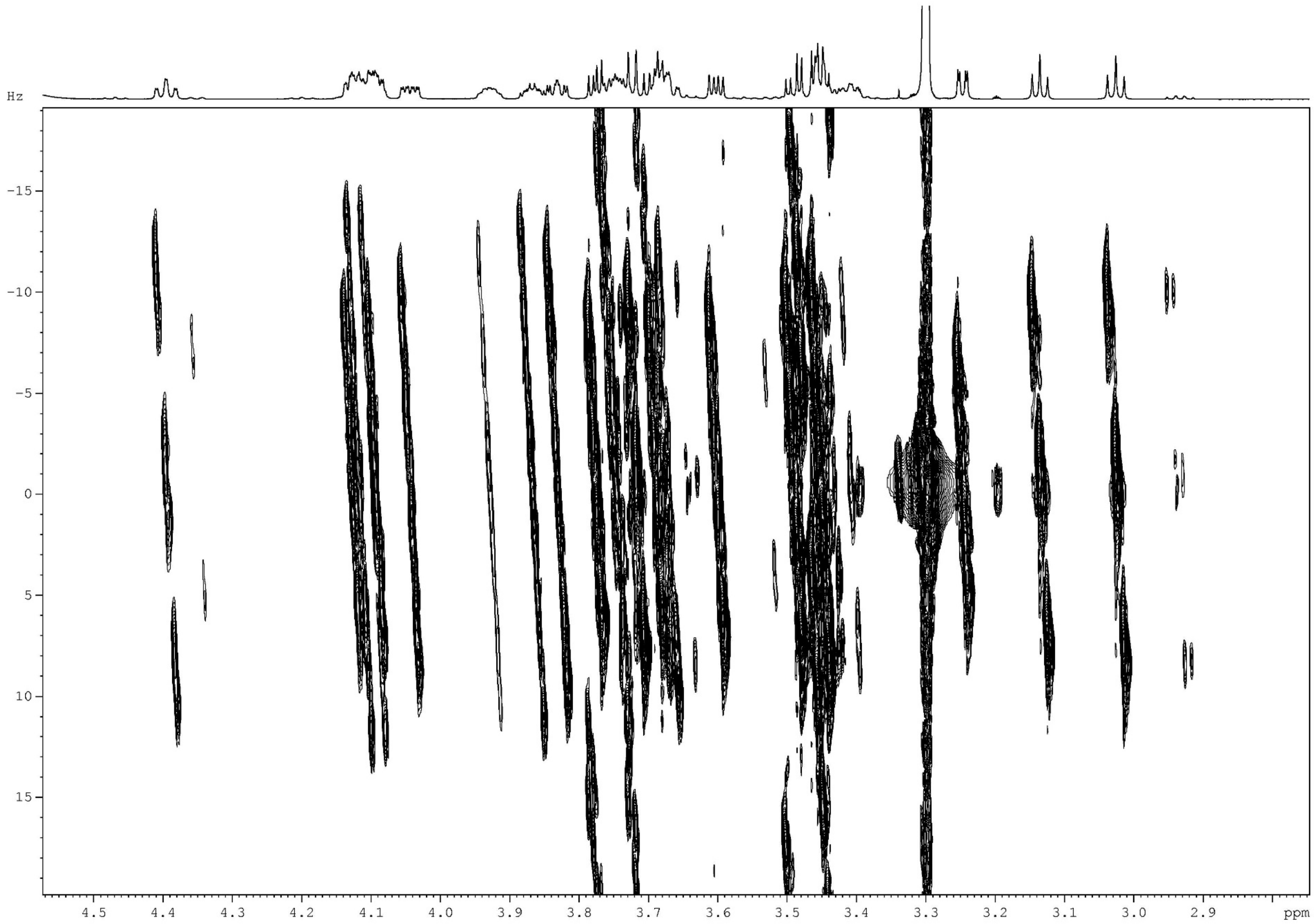

2D *J*RES (700 MHz) spectrum of the fragment **1c** in CD_3OD

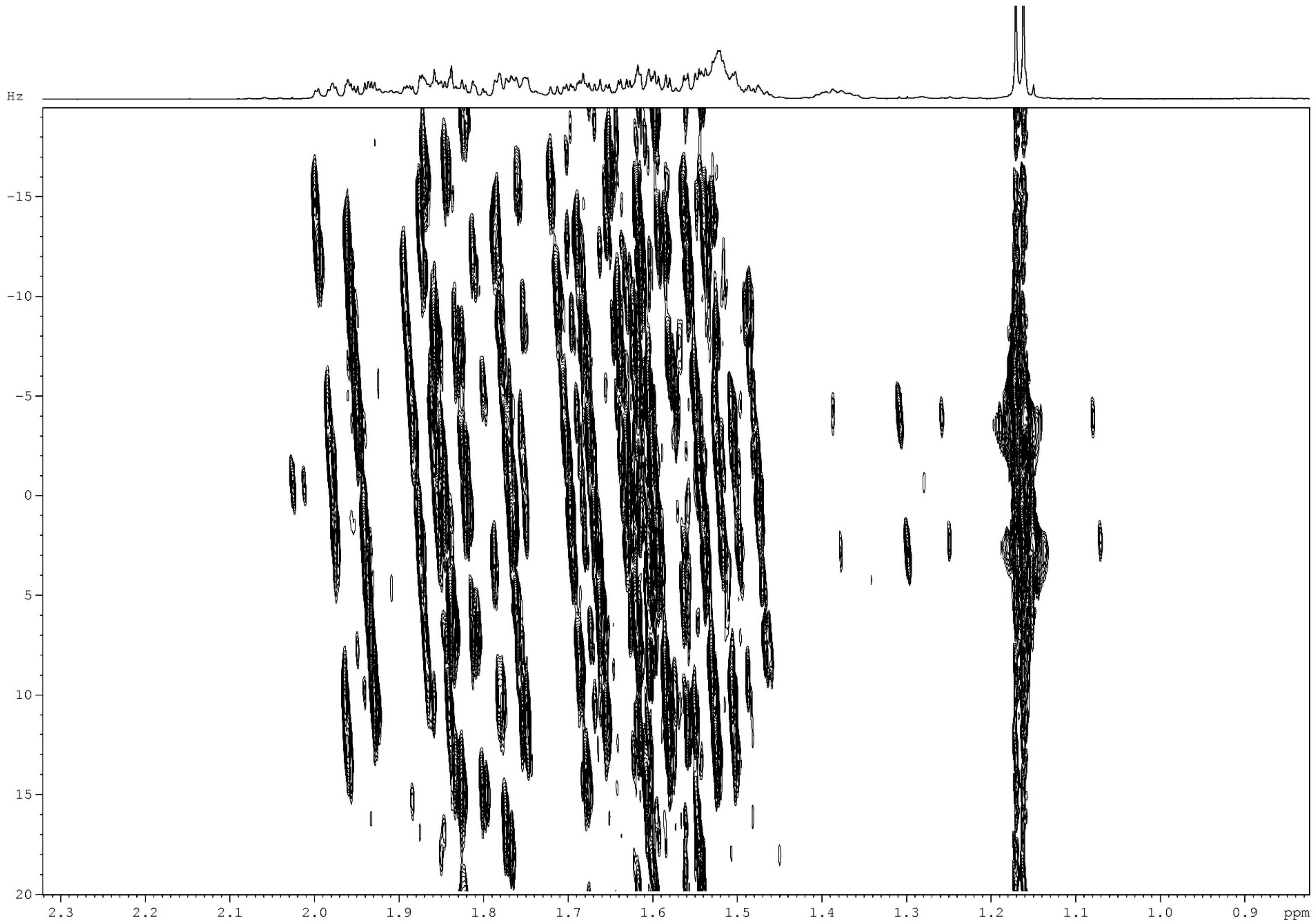

HECADE (700 MHz) spectrum of the fragment **1c** in CD_3OD

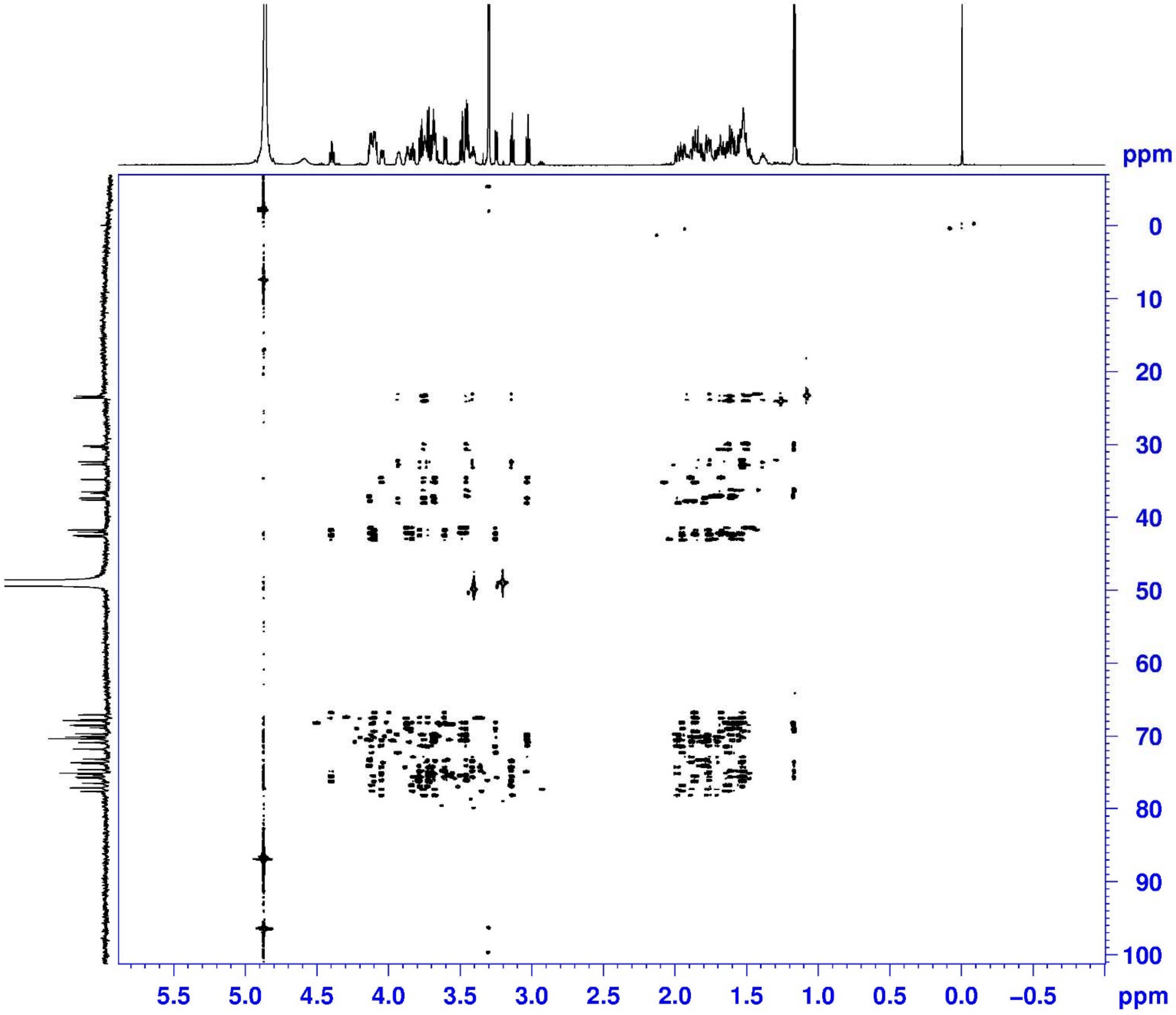

NAME	liwanshan-40-2-2-O3-3	
EXPNO	25	
PROCNO	1	
Date_	20200820	
Time	2.37	h
INSTRUM	spect	
PROBHD	Z120187_0028 (	
PULPROG	hsqcdietgpjcndsisp	
TD	4096	
SOLVENT	MeOD	
NS	56	
DS	16	
SWH	4826.255	Hz
FIDRES	2.356570	Hz
AQ	0.4243956	sec
RG	181.26	
DW	103.600	usec
DE	10.00	usec
TE	298.0	K
CNST2	145.0000000	
CNST16	1.0000000	
CNST17	-0.5000000	
D0	0.00000300	sec
D1	1.00000000	sec
D2	0.00344828	sec
D4	0.00172414	sec
D9	0.08000000	sec
D16	0.00020000	sec
D20	0.00000300	sec
D24	0.00089000	sec
IN0	0.00002620	sec
IN20	0.00002620	sec
L0	1	
L1	28	
ND0	2	
TD	512	
SFO1	176.0684	MHz
FIDRES	37.273376	Hz
SW	108.389	ppm
FnMODE	Echo-Antiecho	
SI	8192	
SF	700.1800200	MHz
WDW	QSINE	
SSB	2	
LB	0.00	Hz
GB	0	
PC	1.40	
SI	1024	
MC2	echo-antiecho	
SF	176.0601510	MHz
WDW	QSINE	
SSB	2	
LB	0.00	Hz

HECADE (700 MHz) spectrum of the fragment **1c** in CD_3OD

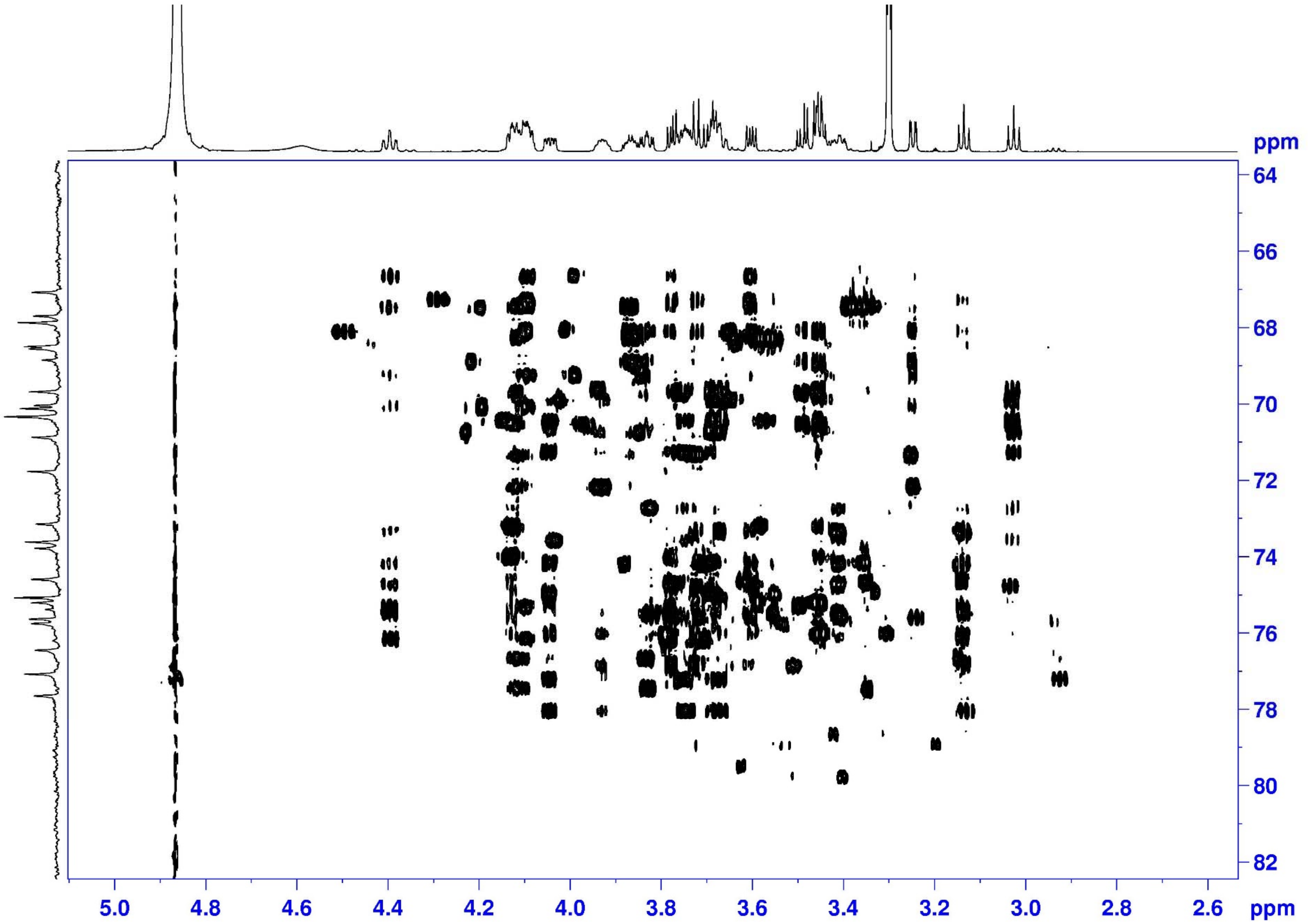

HECADE (700 MHz) spectrum of the fragment **1c** in CD_3OD

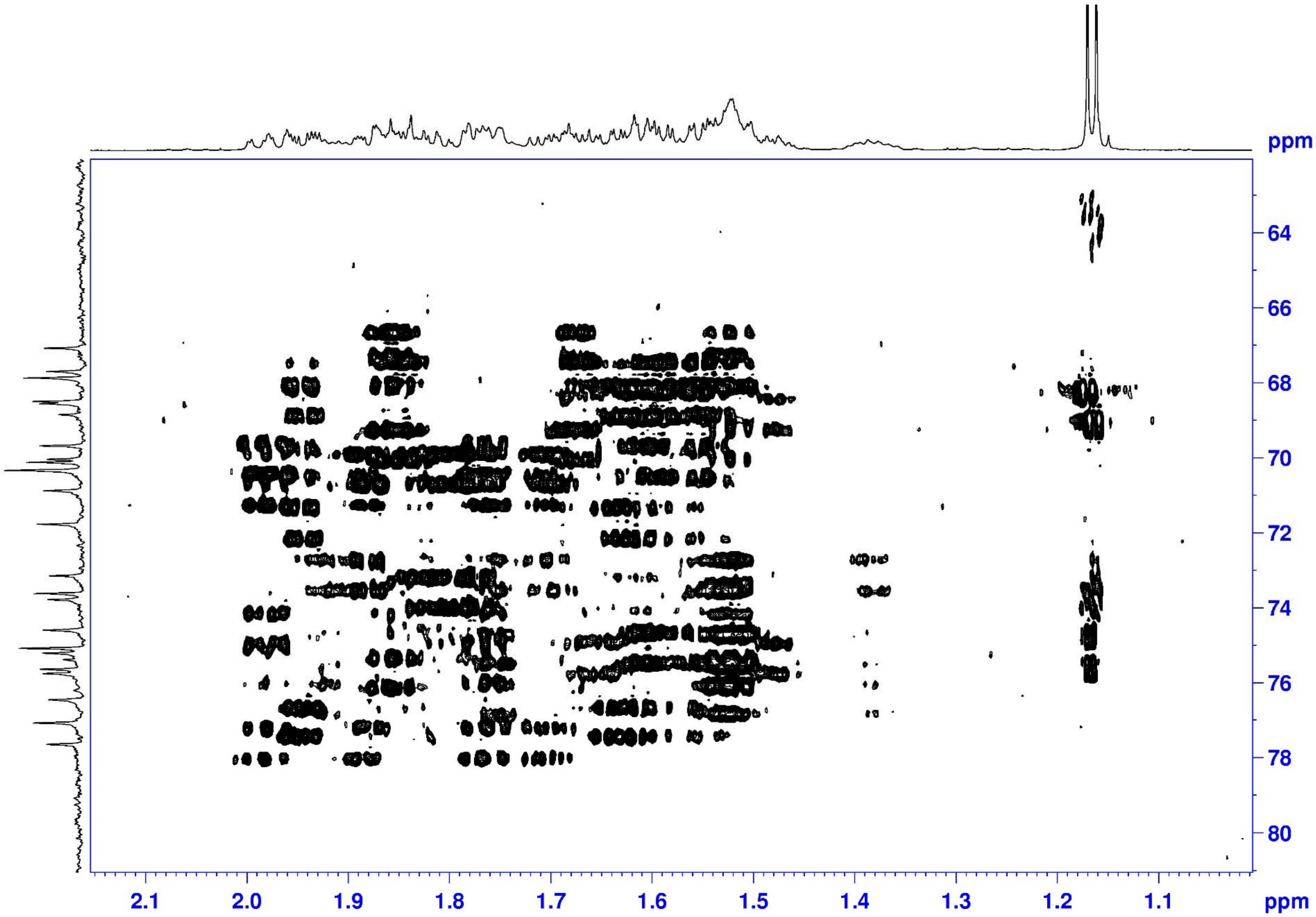

HECADE (700 MHz) spectrum of the fragment **1c** in CD_3OD

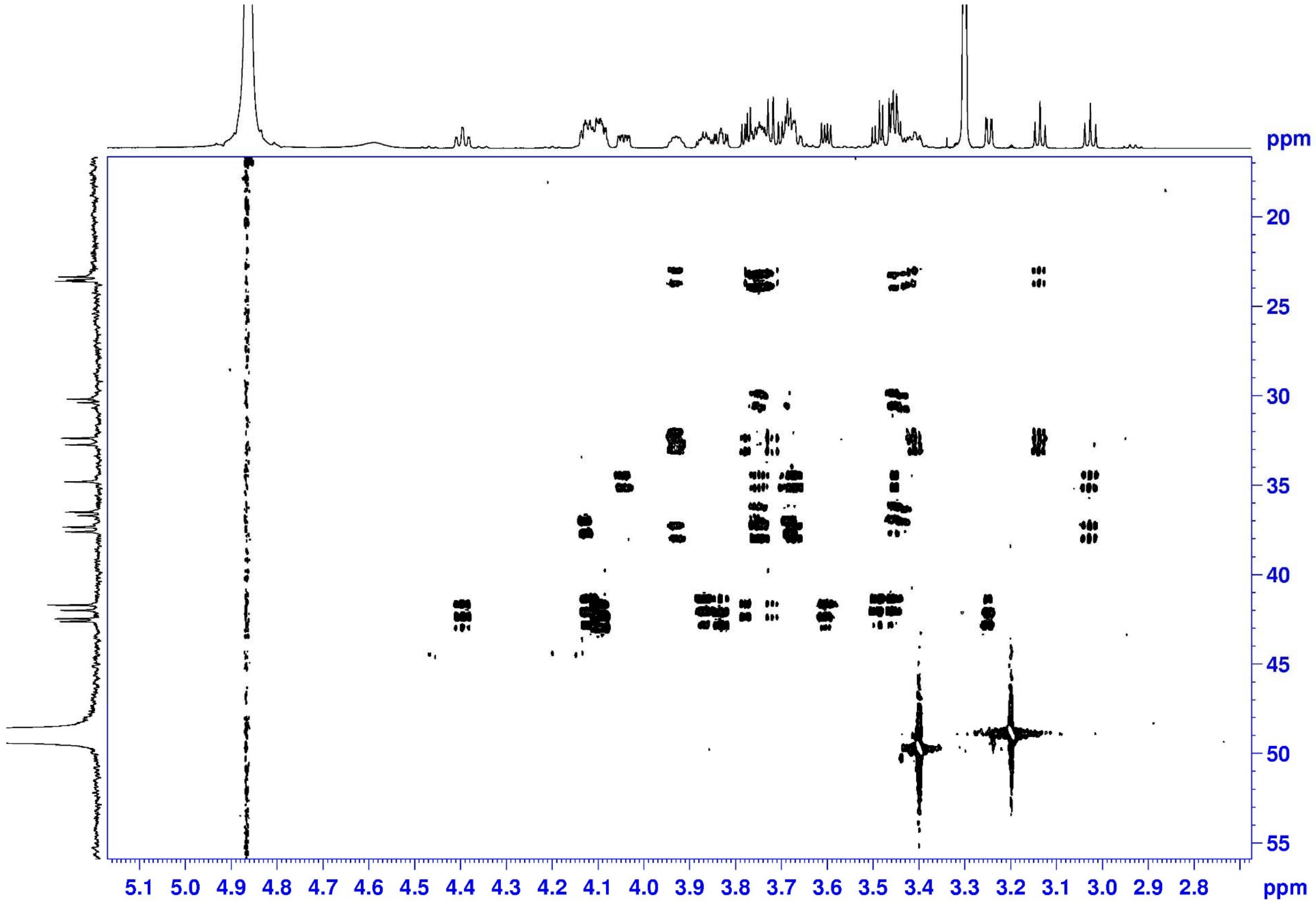

HECADE (700 MHz) spectrum of the fragment **1c** in CD_3OD

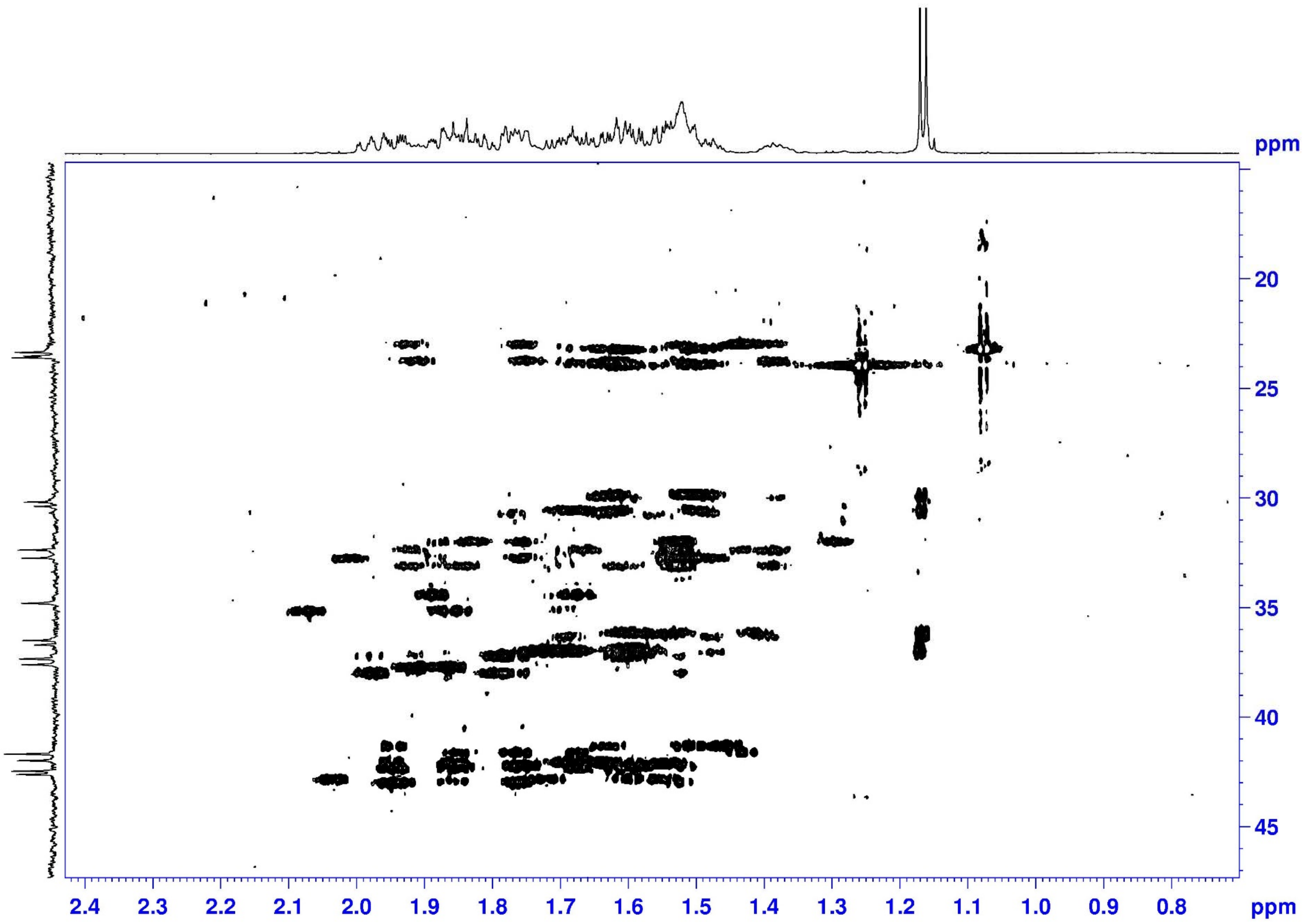

LR-ESI-MS for the fragment **1as**

Generic Display Report

Analysis Info

Analysis Name	D:\Data\amaZon SL\MS\data\202008\liwanshan_40-2-2-O3-1-R-Cl_34_01_2059.d	Acquisition Date	2020-08-21 16:35:57
Method	2059.m	Operator	bruker
Sample Name	liwanshan_40-2-2-O3-1-R-Cl	Instrument	amaZon SL
Comment			

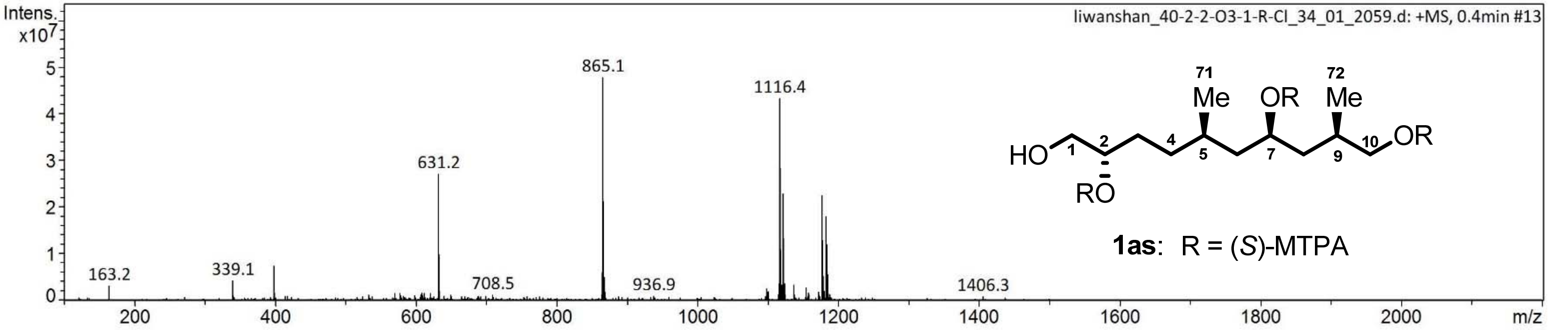

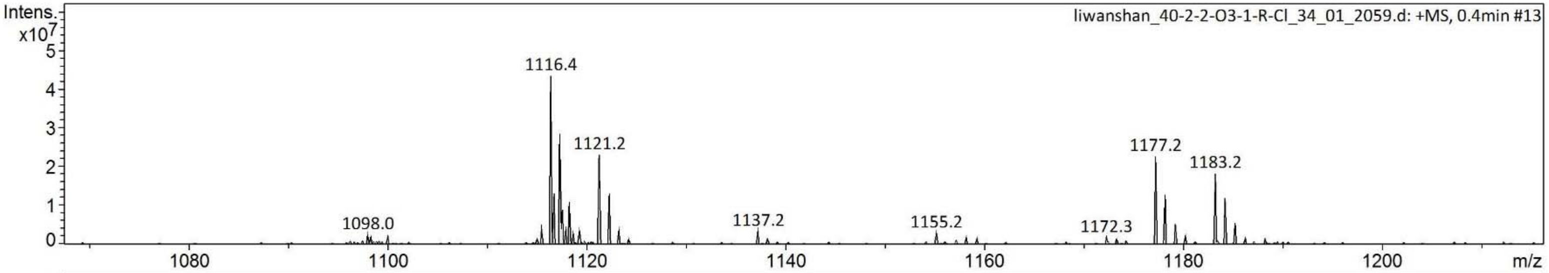

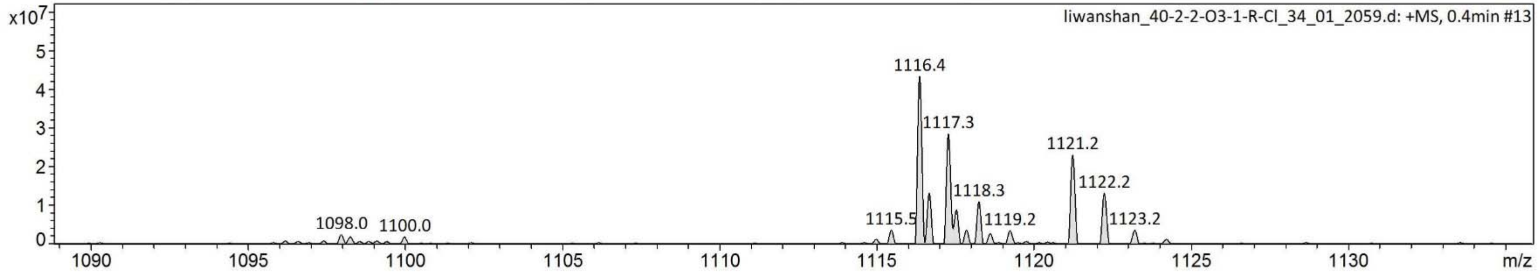

Bruker Compass DataAnalysis 4.4 printed: 2020-08-21 16:45:28 by: bruker Page 1 of 1

^{1}H (400 MHz) NMR spectrum of the fragment **1as** in CD_3OD

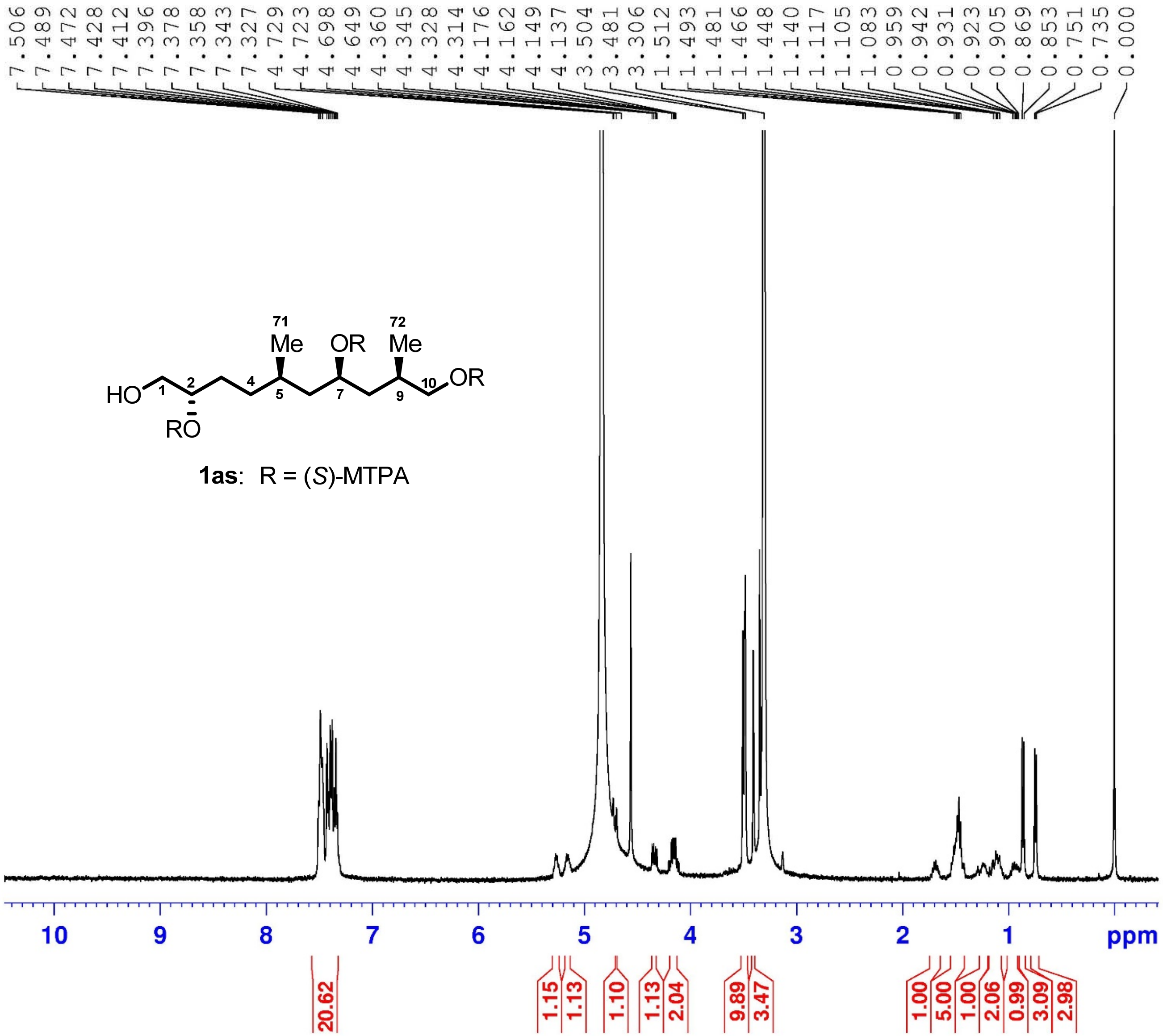

1as: R = (*S*)-MTPA

NAME	40-2-2-O3-1-R-Cl
EXPNO	1
PROCNO	1
Date_	20200425
Time	10.26
INSTRUM	spect
PROBHD	5 mm PABBO BB/
PULPROG	zg30
TD	65536
SOLVENT	MeOD
NS	32
DS	2
SWH	8012.820 Hz
FIDRES	0.122266 Hz
AQ	4.0894966 sec
RG	177.07
DW	62.400 use
DE	6.50 use
TE	300.0 K
D1	1.00000000 sec
TD0	1

======== CHANNEL f1 ========

SFO1	400.1324710 MHz
NUC1	1H
P1	9.73 use
SI	65536
SF	400.1300091 MHz
WDW	EM
SSB	0
LB	0.30 Hz
GB	0
PC	1.00

^{1}H (400 MHz) NMR spectrum of the fragment **1as** in CD_3OD

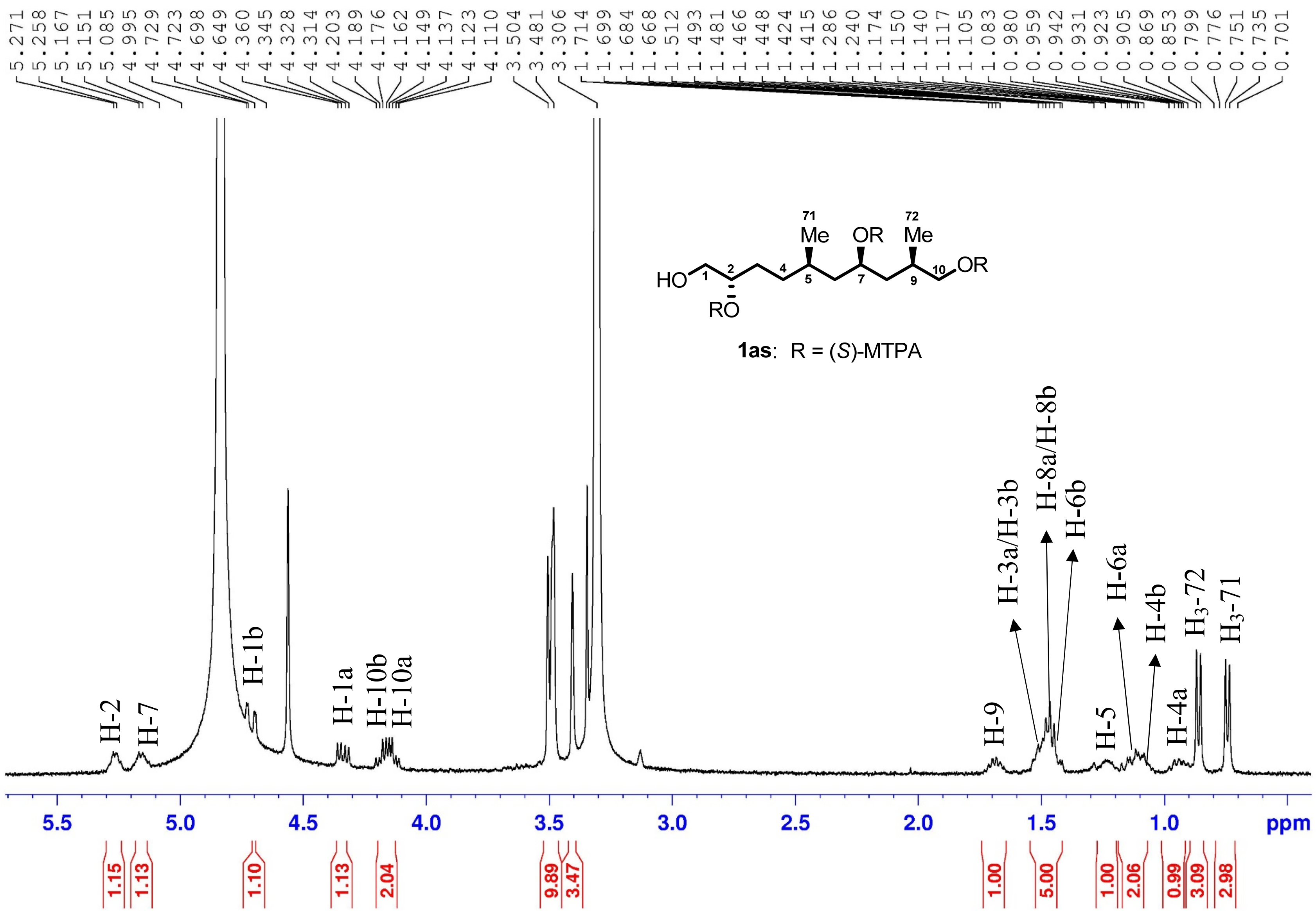

^{1}H (400 MHz) NMR spectrum of the fragment **1as** in CD_3OD

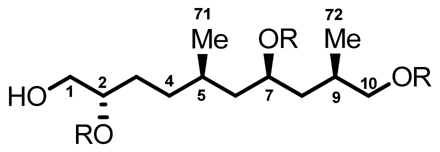

1as: R = (*S*)-MTPA

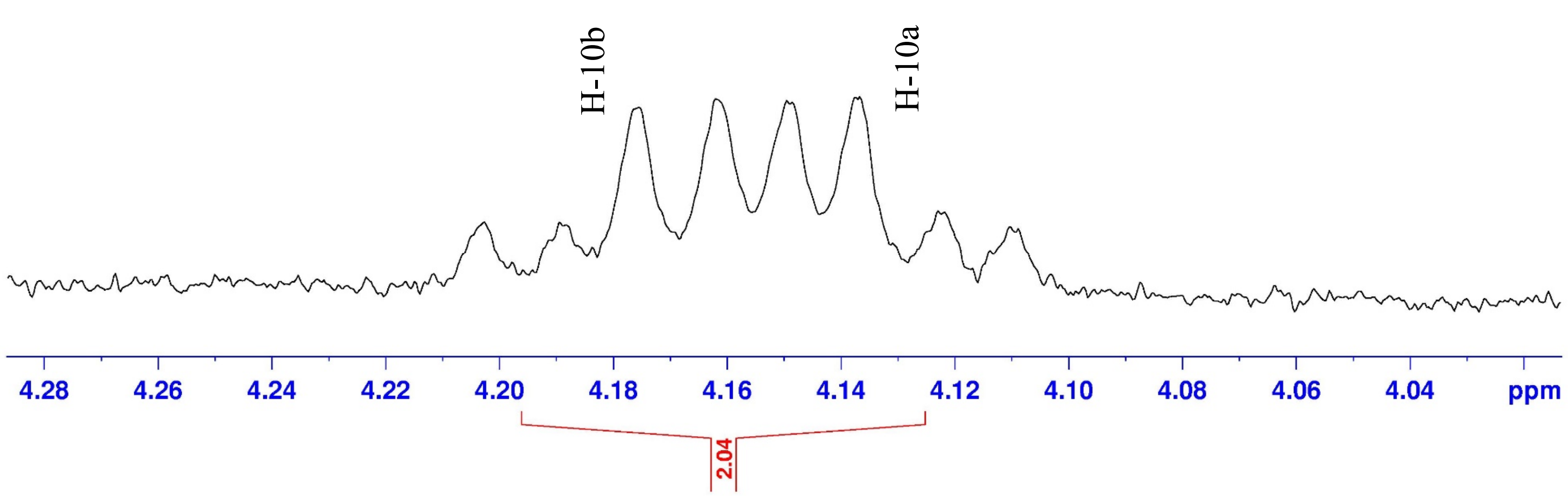

^{1}H–^{1}H COSY (400 MHz) spectrum of the fragment **1as** in CD_3OD

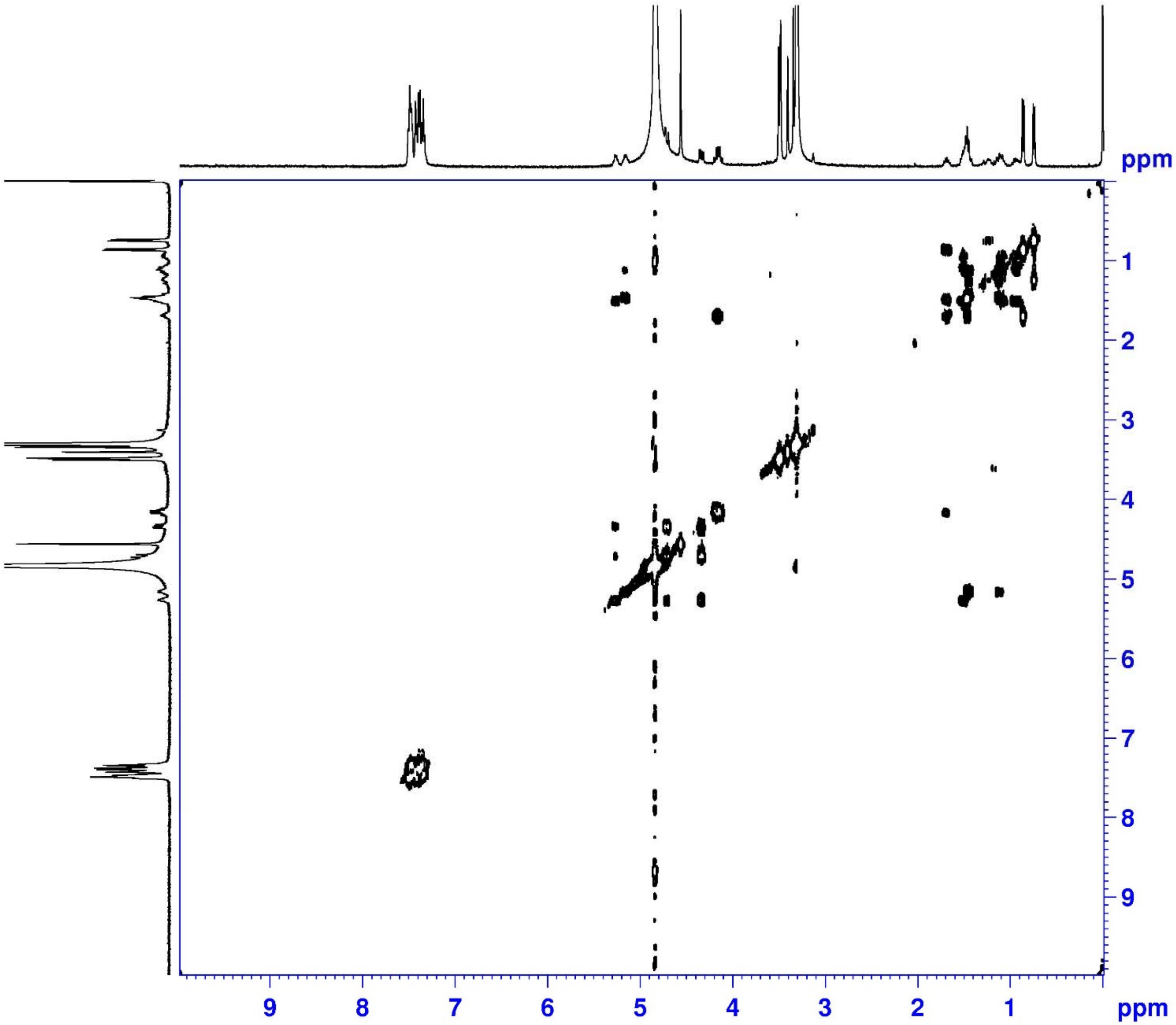


```
NAME       40-2-2-O3-1-R-Cl
EXPNO                     2
PROCNO                    1
Date_              20200425
Time                  10.34
INSTRUM               spect
PROBHD   5 mm PABBO BB/
PULPROG            cosygpqf
TD                     2048
SOLVENT                MeOD
NS                      100
DS                        8
SWH                4000.000 Hz
FIDRES             1.953125 Hz
AQ                0.2560500 sec
RG                    202.1
DW                  125.000 usec
DE                     6.50 usec
TE                    300.0 K
D0               0.00000300 sec
D1               2.00000000 sec
D13              0.00000400 sec
D16              0.00020000 sec
IN0              0.00025000 sec

======== CHANNEL f1 ========
SFO1          400.1320007 MHz
NUC1                     1H
P0                     9.73 usec
P1                     9.73 usec
ND0                       1
TD                      128
SFO1                400.132 MHz
FIDRES            31.250000 Hz
SW                    9.997 ppm
FnMODE                   QF
SI                     1024
SF            400.1300091 MHz
WDW                   QSINE
SSB                       0
LB                     0.00 Hz
GB                        0
PC                     1.40
SI                     1024
MC2                      QF
SF            400.1300091 MHz
WDW                   QSINE
SSB                       0
LB                     0.00 Hz
```

^{1}H–^{1}H COSY (400 MHz) spectrum of the fragment **1as** in CD_3OD

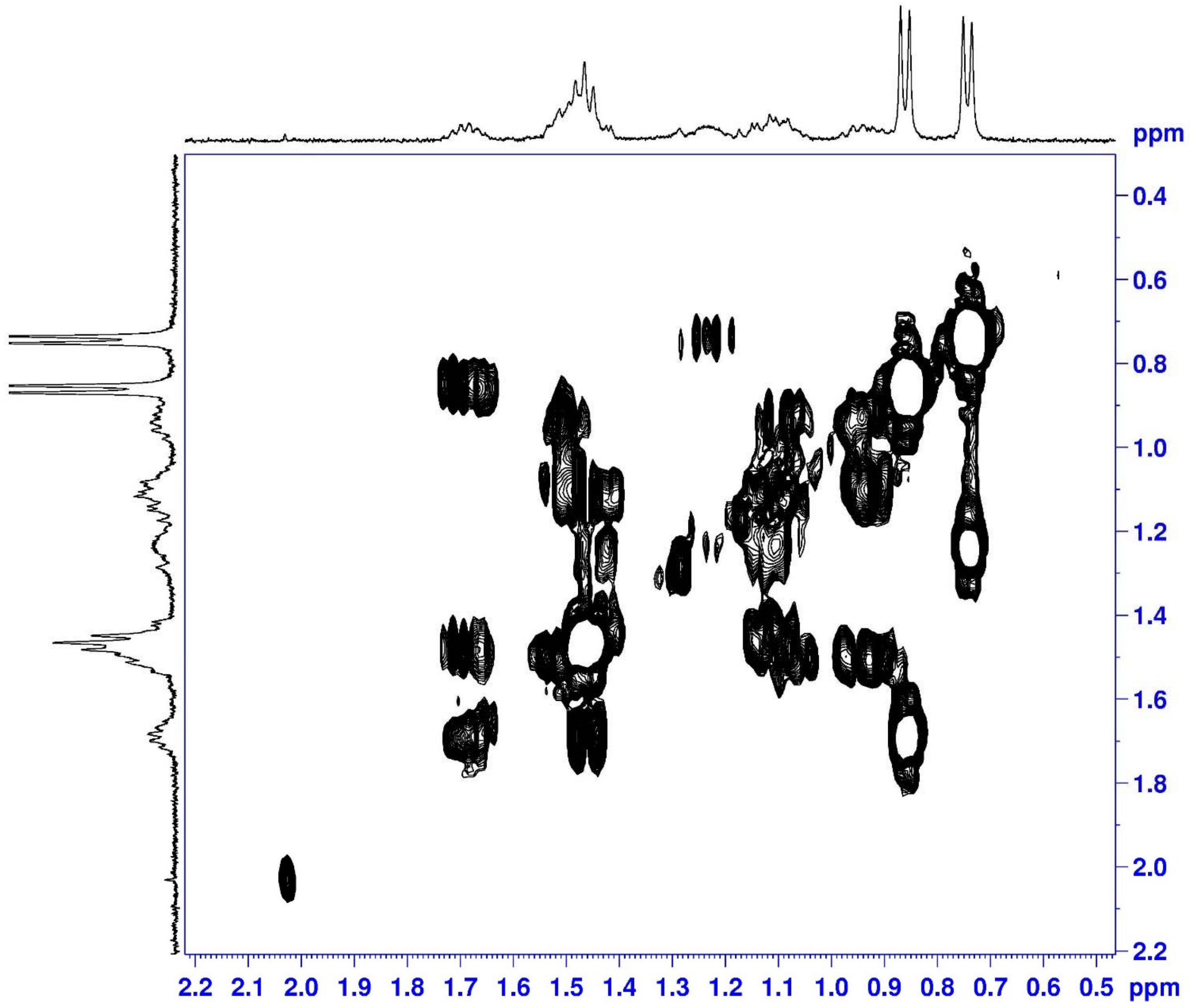

^1H–^1H COSY (400 MHz) spectrum of the fragment **1as** in CD_3OD

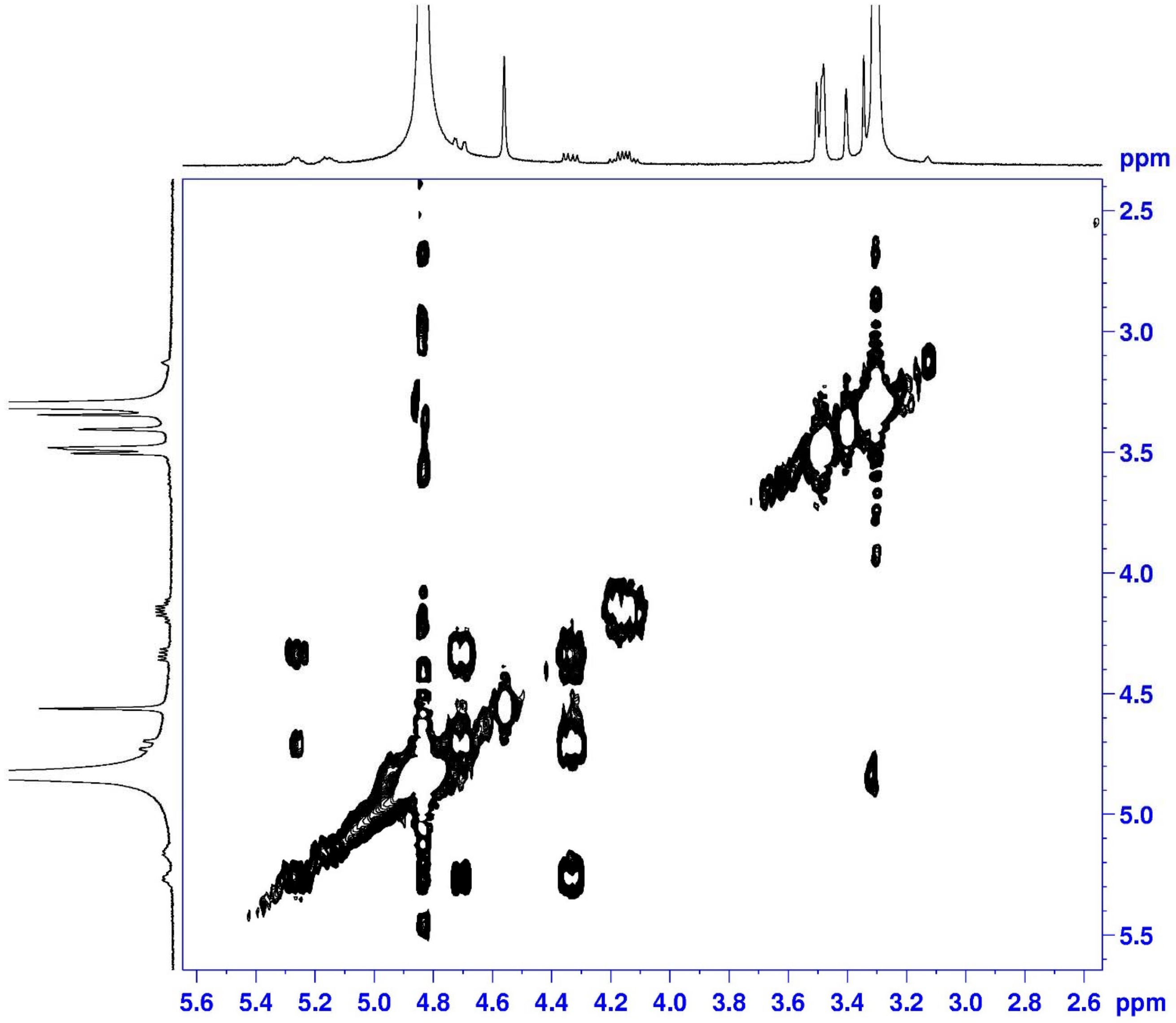

^{1}H–^{1}H COSY (400 MHz) spectrum of the fragment **1as** in CD_3OD

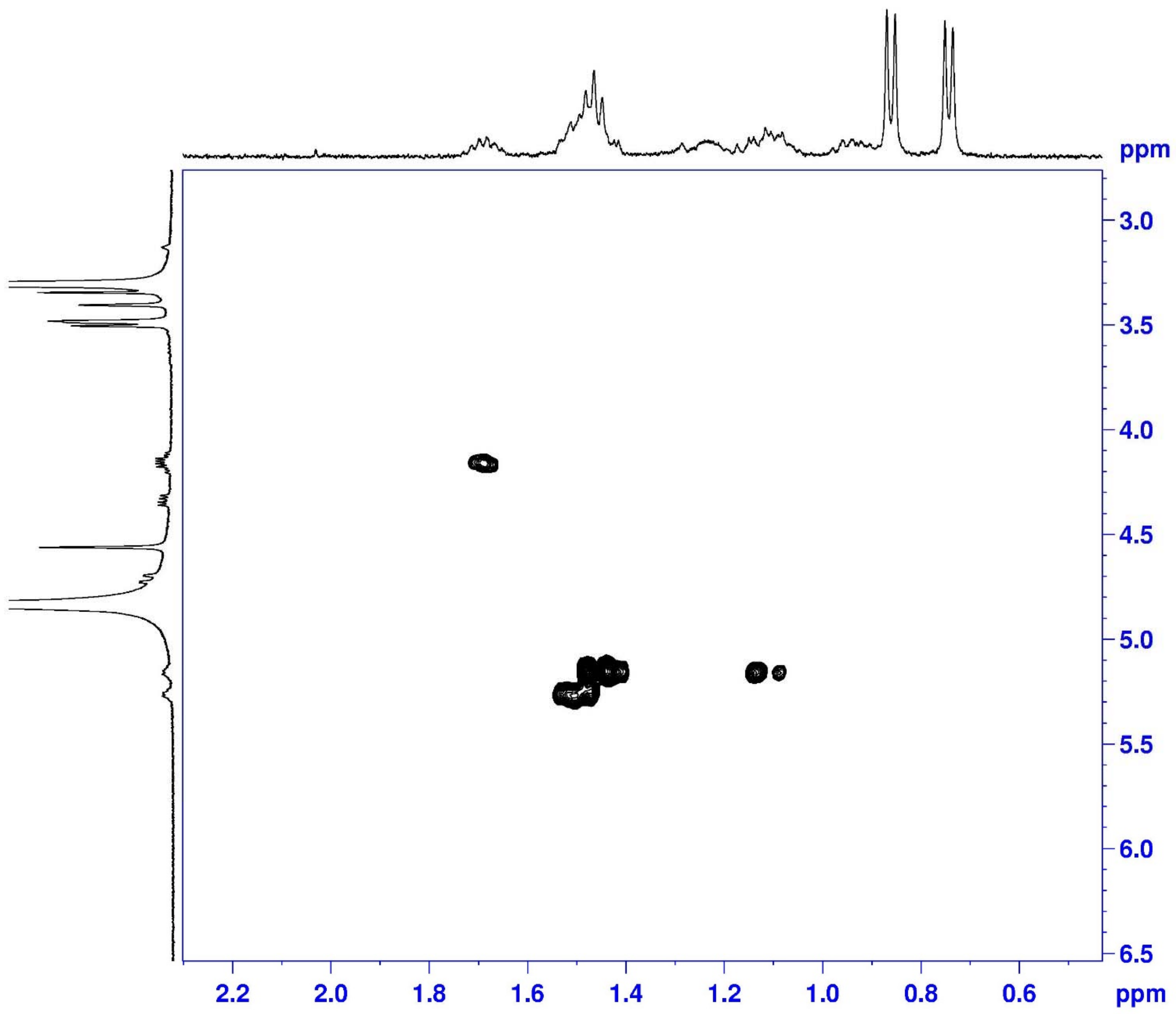

LR-ESI-MS for the fragment **1ar**

Generic Display Report

Analysis Info

Analysis Name	D:\Data\amaZon SL\MS\data\202008\liwanshan_40-2-2-O3-1-S-Cl_33_01_2060.d	Acquisition Date	2020-08-21 16:38:24
Method	2060.m	Operator	bruker
Sample Name	liwanshan_40-2-2-O3-1-S-Cl	Instrument	amaZon SL
Comment			

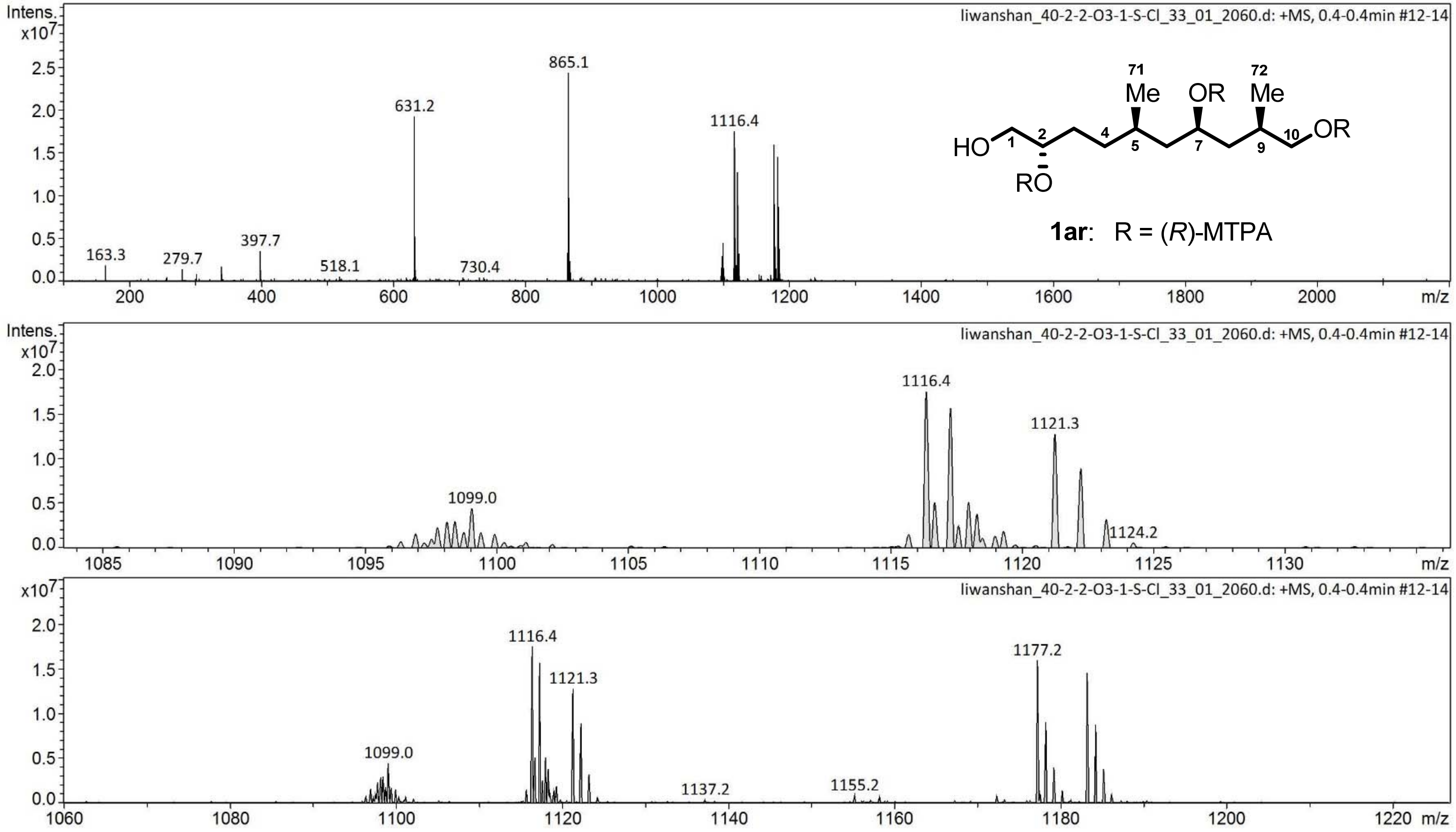

^{1}H (400 MHz) NMR spectrum of the fragment **1ar** in CD_3OD

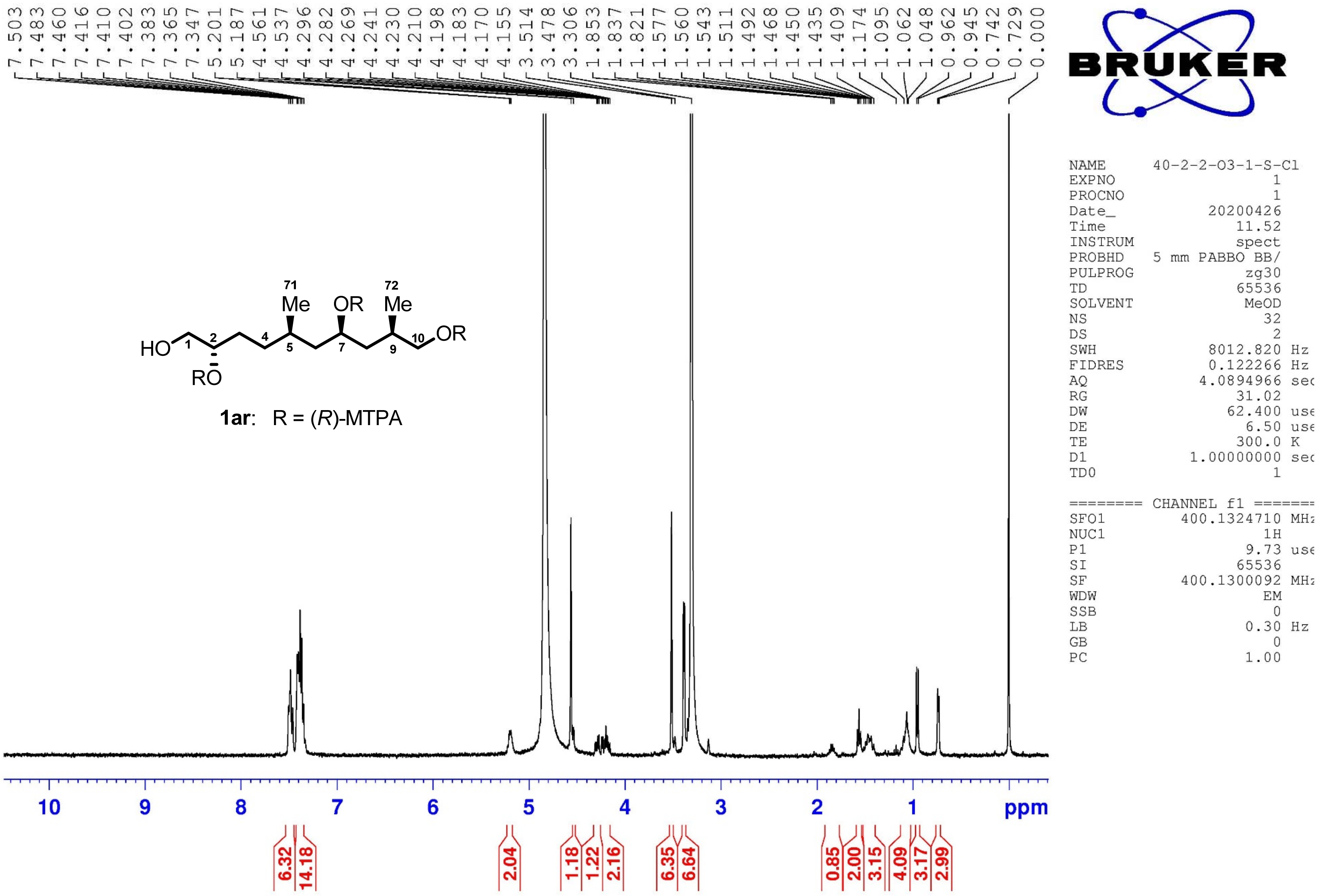

^{1}H (400 MHz) NMR spectrum of the fragment **1ar** in CD_3OD

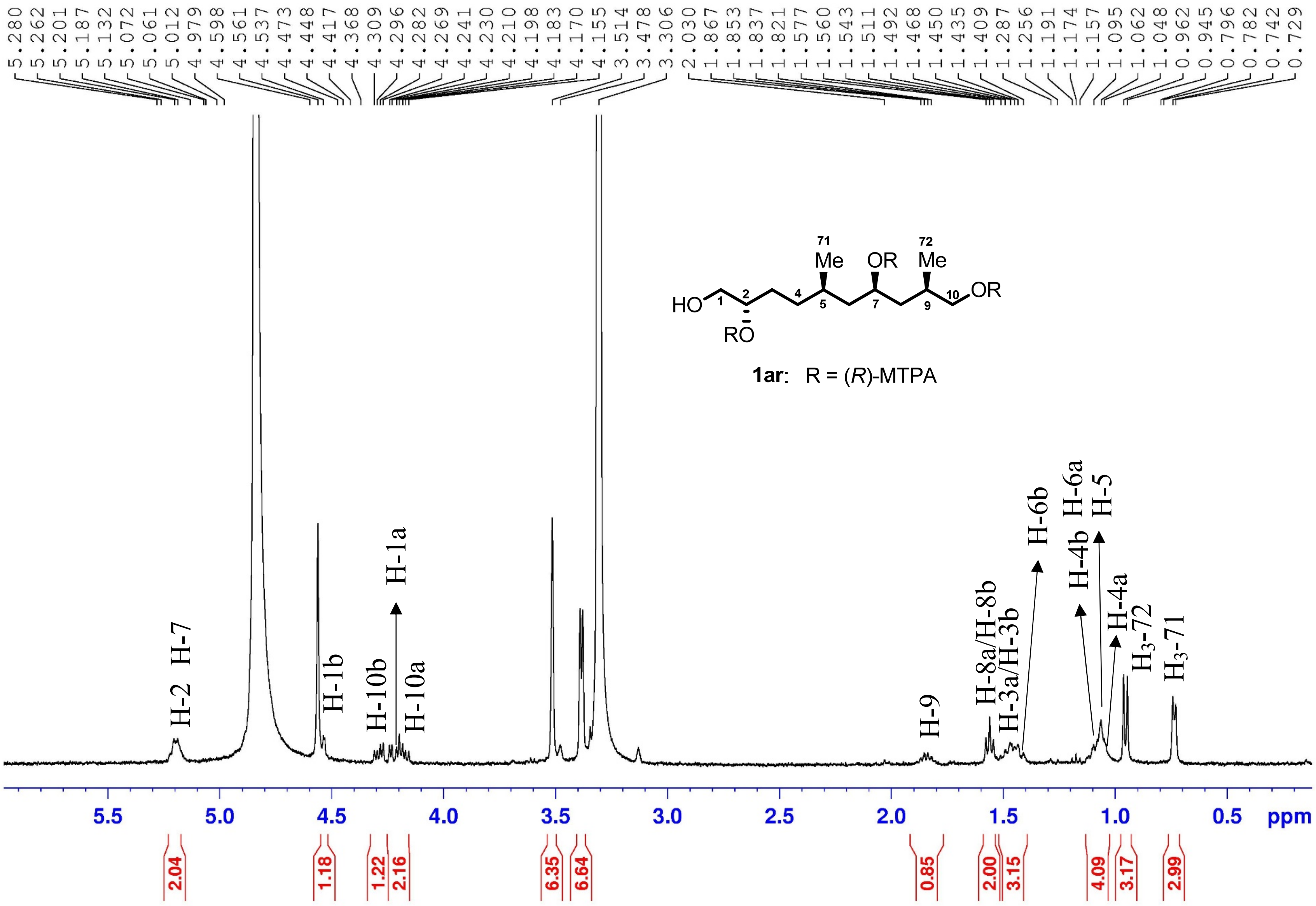

^{1}H (400 MHz) NMR spectrum of the fragment **1ar** in CD_3OD

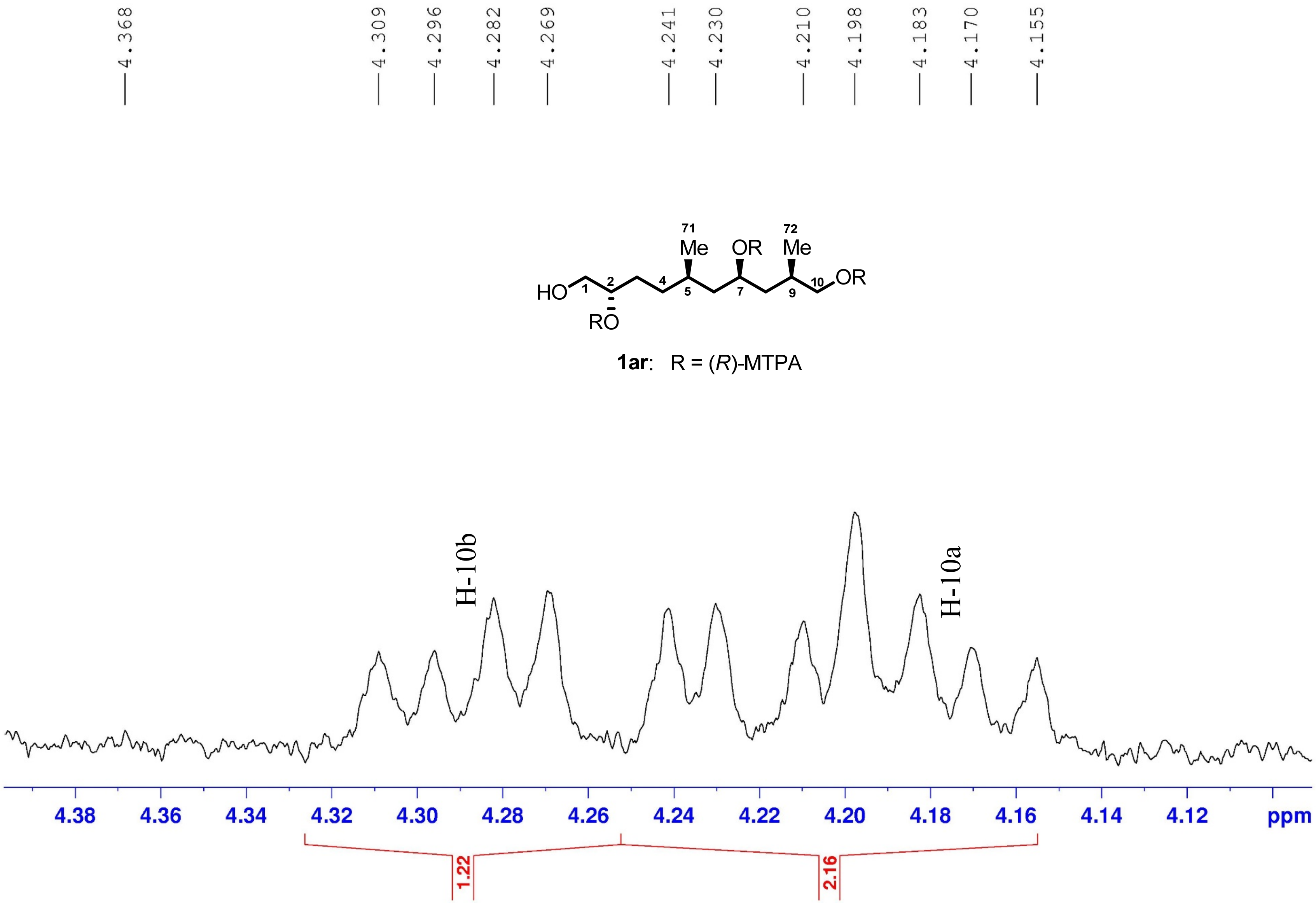

^1H–^1H COSY (400 MHz) spectrum of the fragment **1ar** in CD_3OD

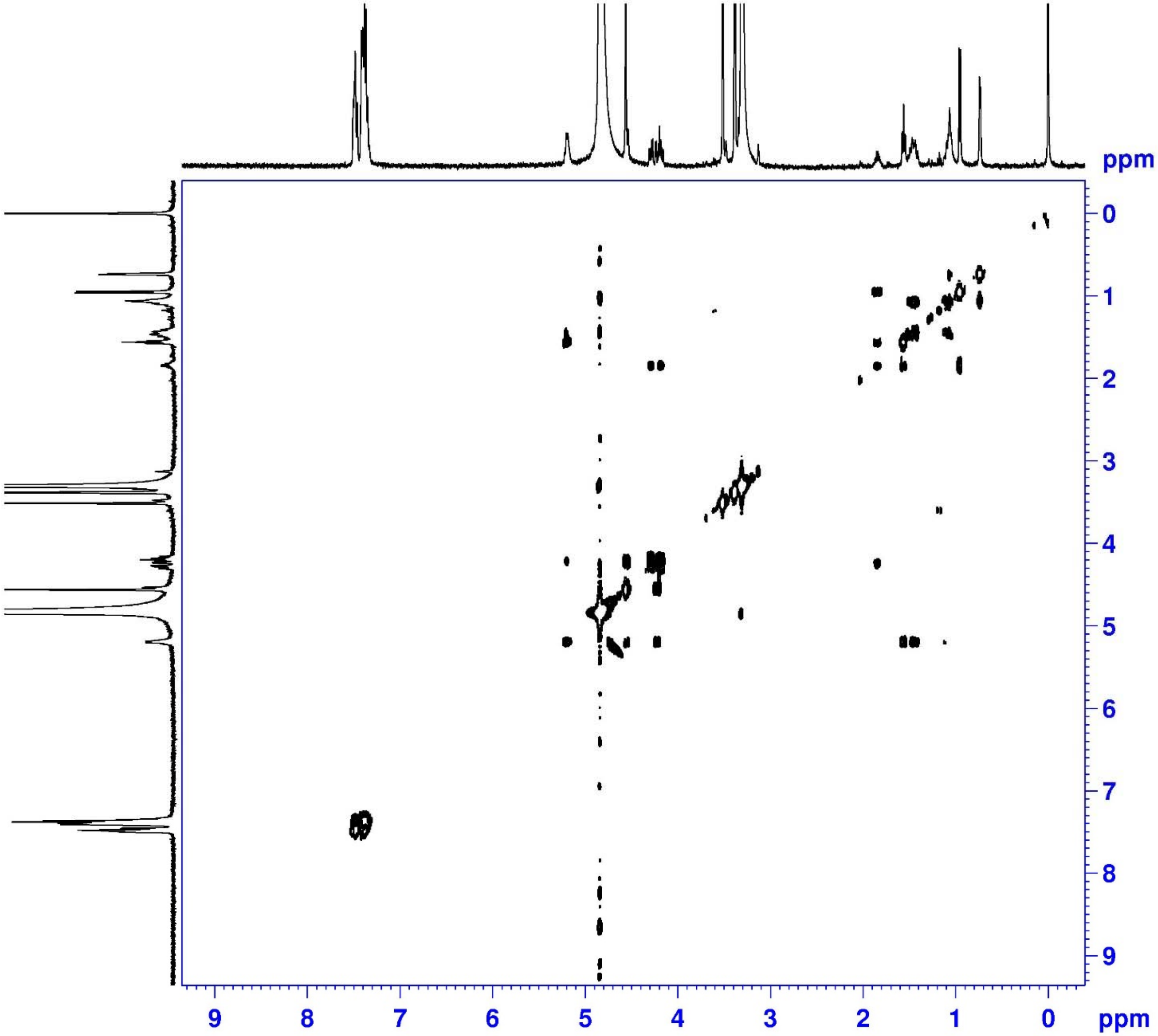


```
NAME        40-2-2-O3-1-S-Cl
EXPNO                      2
PROCNO                     1
Date_               20200426
Time                   12.00
INSTRUM                spect
PROBHD     5 mm PABBO BB/
PULPROG             cosygpqf
TD                      2048
SOLVENT                 MeOD
NS                       100
DS                         8
SWH                 4000.000 Hz
FIDRES              1.953125 Hz
AQ                 0.2560500 sec
RG                     202.1
DW                   125.000 usec
DE                      6.50 usec
TE                     300.0 K
D0                0.00000300 sec
D1                2.00000000 sec
D13               0.00000400 sec
D16               0.00020000 sec
IN0               0.00025000 sec

======== CHANNEL f1 ========
SFO1             400.1320007 MHz
NUC1                      1H
P0                      9.73 usec
P1                      9.73 usec
ND0                        1
TD                       128
SFO1                 400.132 MHz
FIDRES             31.250000 Hz
SW                     9.997 ppm
FnMODE                    QF
SI                      1024
SF               400.1300092 MHz
WDW                    QSINE
SSB                        0
LB                      0.00 Hz
GB                         0
PC                      1.40
SI                      1024
MC2                       QF
SF               400.1300092 MHz
WDW                    QSINE
SSB                        0
LB                      0.00 Hz
```

^1H–^1H COSY (400 MHz) spectrum of the fragment **1ar** in CD_3OD

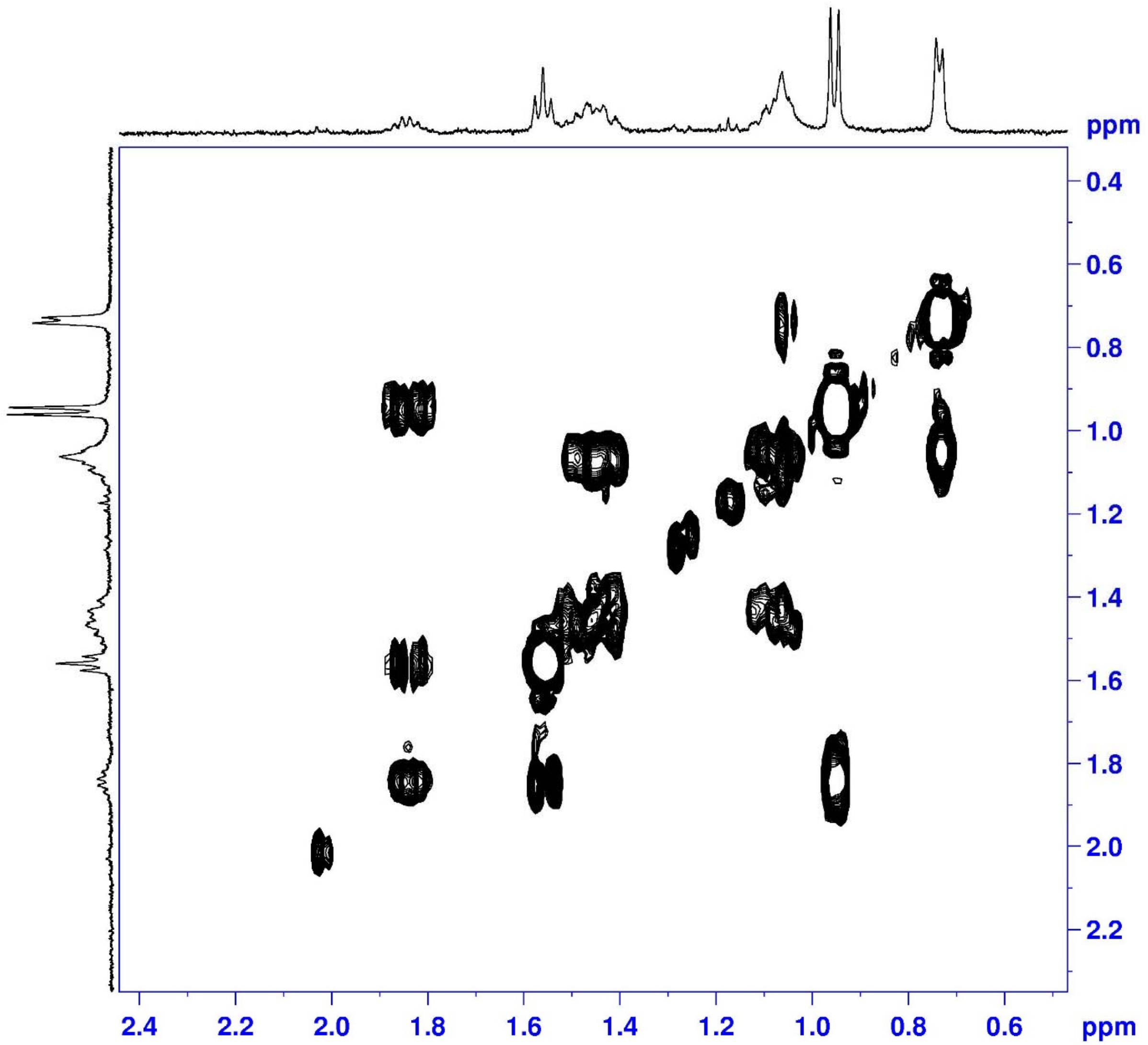

^{1}H–^{1}H COSY (400 MHz) spectrum of the fragment **1ar** in CD_3OD

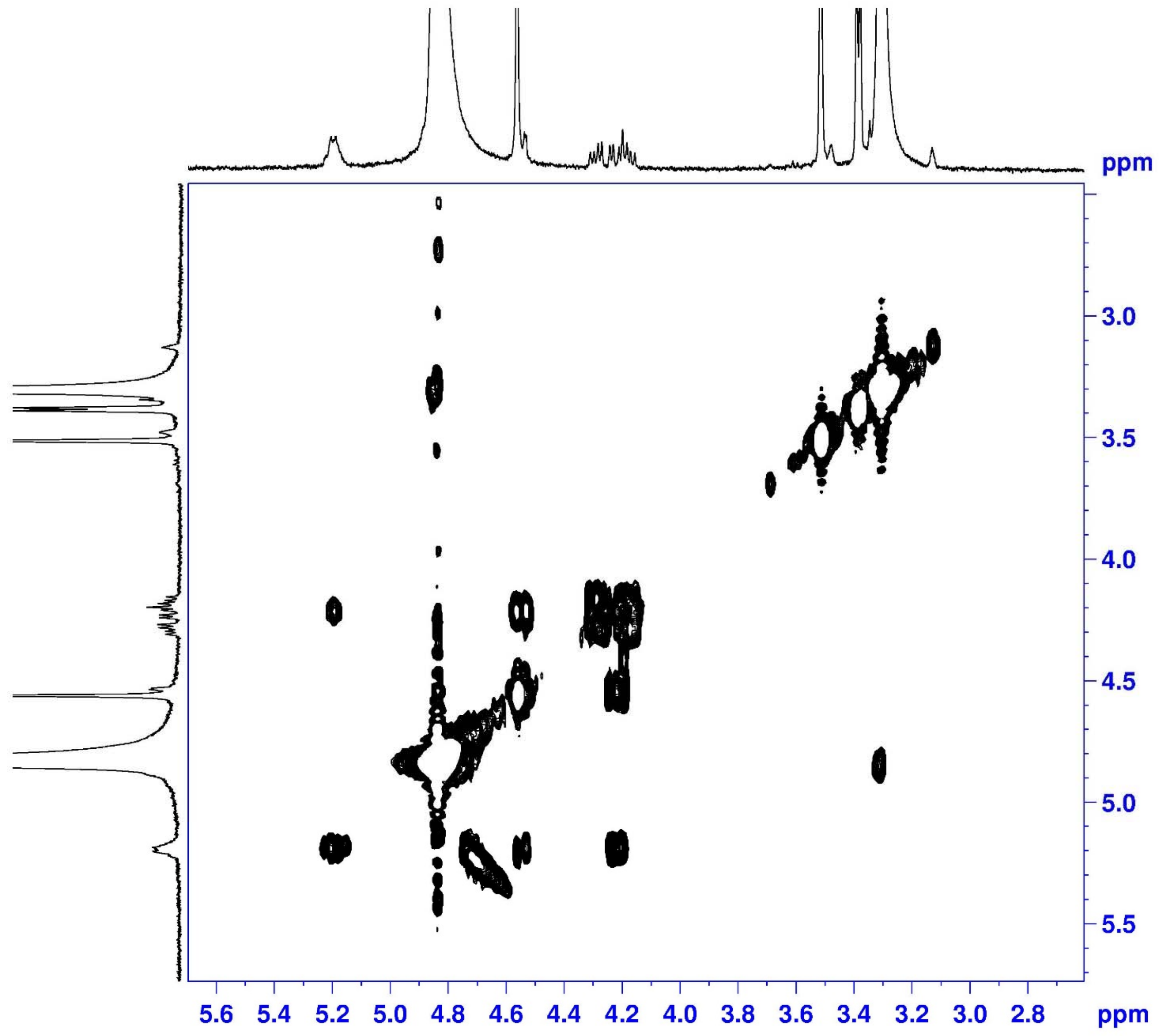

^{1}H–^{1}H COSY (400 MHz) spectrum of the fragment **1ar** in CD_3OD

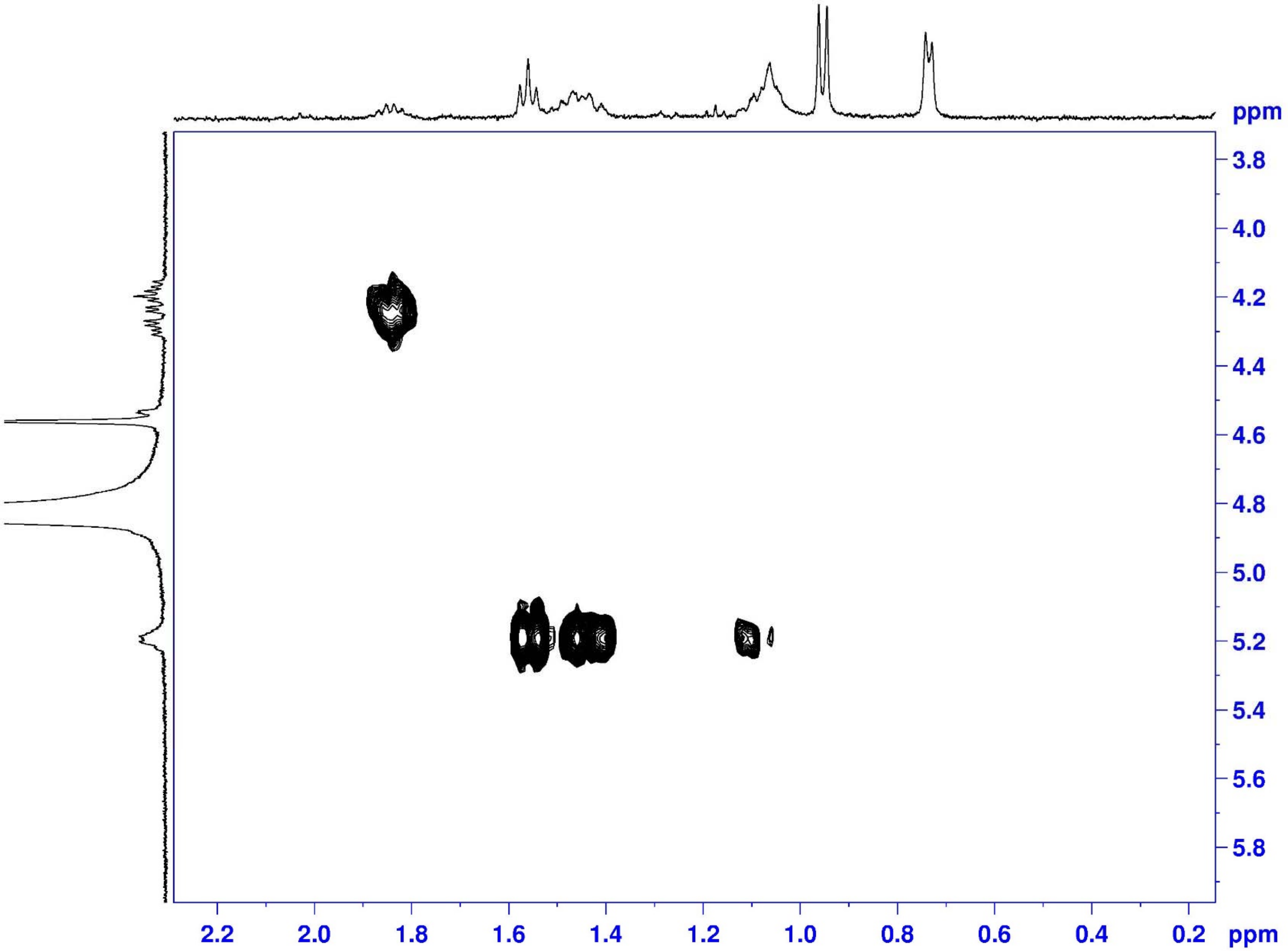

Supplement: Supplementary file 1 [file marinedrugs-18-00590-s001.pdf]
